# Impact of Genomics on Clarifying the Evolutionary Relationships amongst Mycobacteria: Identification of Molecular Signatures Specific for the Tuberculosis-Complex of Bacteria with Potential Applications for Novel Diagnostics and Therapeutics

**DOI:** 10.3390/ht7040031

**Published:** 2018-10-02

**Authors:** Radhey S. Gupta

**Affiliations:** Department of Biochemistry and Biomedical Sciences, McMaster University, Hamilton, ON L8N 3Z5, Canada; gupta@mcmaster.ca; Tel.: +1-905-525-9140

**Keywords:** mycobacterial genomes, comparative genomics, phylogenomics, tuberculosis-complex, novel drug targets, conserved signature indels, protein structures and surface loops, mycobacterial classification

## Abstract

An alarming increase in tuberculosis (TB) caused by drug-resistant strains of *Mycobacterium tuberculosis* has created an urgent need for new antituberculosis drugs acting via novel mechanisms. Phylogenomic and comparative genomic analyses reviewed here reveal that the TB causing bacteria comprise a small group of organisms differing from all other mycobacteria in numerous regards. Comprehensive analyses of protein sequences from mycobacterial genomes have identified 63 conserved signature inserts and deletions (indels) (CSIs) in important proteins that are distinctive characteristics of the TB-complex of bacteria. The identified CSIs provide potential means for development of novel diagnostics as well as therapeutics for the TB-complex of bacteria based on four key observations: (i) The CSIs exhibit a high degree of exclusivity towards the TB-complex of bacteria; (ii) Earlier work on CSIs provide evidence that they play important/essential functions in the organisms for which they exhibit specificity; (iii) CSIs are located in surface-exposed loops of the proteins implicated in mediating novel interactions; (iv) Homologs of the CSIs containing proteins, or the CSIs in such homologs, are generally not found in humans. Based on these characteristics, it is hypothesized that the high-throughput virtual screening for compounds binding specifically to the CSIs (or CSI containing regions) and thereby inhibiting the cellular functions of the CSIs could lead to the discovery of a novel class of drugs specifically targeting the TB-complex of organisms.

## 1. Introduction

Tuberculosis (TB), caused by the bacterium *Mycobacterium tuberculosis*, is the leading cause of death from an infectious agent worldwide [1]. In 2016, an estimated 10.4 million people were infected with TB, leading to the deaths of 1.7 million people [1]. Additionally, a third of the global population is latently infected with *M. tuberculosis* and is at risk of developing the active disease. These factors have led to TB being declared as a global health emergency by the World Health Organization [1]. Alarmingly, an increasing number of new cases of TB are due to multidrug-resistant (MDR), or extensively drug-resistant (XDR), strains of *M. tuberculosis*, which are not responsive to different first-line TB drugs as well as many other drugs used for the treatment of TB [1,2,3]. This is leading towards a crisis situation in the treatment and/or management of TB, where unless new drugs that are effective against the resistant strains of TB are developed [4,5,6,7], it will become very difficult to treat and control the spread of drug-resistant TB in the population. Thus, there is an urgent need for the development of new antimycobacterial drugs, acting via novel mechanisms, which are effective in killing both sensitive as well as resistant strains of *M. tuberculosis*. The availability of genome sequences for *M. tuberculosis* and other mycobacteria has provided a great impetus in the search for new drug targets for development of novel therapeutics for treatment of TB [5,6,7,8,9]. However, thus far the use of genomics for the identification of novel drug targets and treatment of TB has been explored to only a limited extent [5,6,7,8,9] and innovative comparative genomic approaches hold much promise of identifying many novel drug targets that can be exploited for the development of a new arsenal of antituberculosis drugs. 

For identification of potential drug targets for treatment of tuberculosis, it is important to first understand how the TB causing bacteria are related to, or differ from, other bacteria. In this context, it should be noted that *M. tuberculosis* is a member of the genus *Mycobacterium*, which until recently constituted the sole genus within the family *Mycobacteriaceae*, containing >188 different named species [10,11]. While a limited number of species from the genus *Mycobacterium* are important human and animal pathogens (e.g., *M. tuberculosis*, *Mycobacterium leprae* and *Mycobacterium bovis*) [10], a vast majority of the species within this genus are nonpathogenic and are found in diverse environments including water bodies, soil, and metalworking fluids [12,13,14,15]. Until recently, members of the genus *Mycobacterium* (family *Mycobacteriaceae*), which are a part of the phylum Actinobacteria [16], were distinguished from other Actinobacteria and other phyla of bacteria primarily on the basis of their distinct branching in phylogenetic trees based on 16S rRNA or other genes/proteins sequences [10,13,17]. Apart from their branching pattern in phylogenetic trees, no reliable characteristic was known that was specific for either all mycobacteria, or different main groups present within this genus/family, which can reliably differentiate the important groups of pathogenic species from nonpathogenic mycobacteria [10,13,17].

Genome sequences are now available for >150 of the 188 known mycobacterial species providing an excellent coverage of the genetic diversity existing within the genus *Mycobacterium* and providing a comprehensive resource for robustly elucidating the evolutionary relationships among mycobacterial species using different genome-scale approaches [18,19,20]. Additionally, comparative analyses of genome sequences by innovative approaches provide a rich resource for the identification of novel molecular characteristics that are specifically shared by either all mycobacteria or different major groups within mycobacteria that can now be reliably delineated [16,21,22,23]. In this review, I will first present an overview of the overall evolutionary relationships and the new classification scheme for mycobacteria that has emerged from comprehensive phylogenomic and comparative genomic approaches. The remainder of this review focuses on the results from this comparative genomic analysis which has identified >60 novel and highly-specific molecular characteristics, in the forms of conserved signature inserts and deletions (CSIs) in important proteins, that are exclusive to the *M. tuberculosis* complex of bacteria. The importance and usefulness of the identified CSIs as a new class of potential drug targets for development of novel drugs/compounds that will specifically target the TB-group of bacteria is discussed in the review.

## 2. Impact of Genomics on Clarifying the Evolutionary Relationships amongst Mycobacteria

As noted earlier, the genus *Mycobacterium* up to the beginning of this year contained 188 named species, which included several major human pathogens (viz. *M. tuberculosis* and *M. leprae*) as well as a large number of nonpathogenic species [10,24]. In our recent work, using available genome sequences for 150 mycobacterial species, comprehensive phylogenomics and comparative genome analyses were carried out using multiple independent approaches with the aim of understanding the evolutionary relationships among mycobacterial species [25]. Genome sequences permitted construction of phylogenetic trees for mycobacterial species based on multiple large datasets of protein sequences including 1941 core proteins representing the genus *Mycobacterium*, 136 core proteins specific for the phylum Actinobacteria, and 8 other highly conserved proteins that are found in all/most bacteria [25]. Phylogenetic trees based on large datasets of protein sequences are known to more accurately portray the evolutionary relationships within any given group of organisms than those based on single gene/protein sequences [19,26,27,28,29]. In all of the constructed trees, *Mycobacterium* species consistently grouped into five strongly supported clades at the highest level, designated as the Tuberculosis-Simiae, Terrae, Triviale, Fortuitum-Vaccae and Abscessus-Chelonae” clades. Some of these clades have also been observed in earlier phylogenetic studies [10,13,17,25]. Of these clades, the Tuberculosis-Simiae, Terrae, Triviale clades are largely comprised of slow-growing bacteria (i.e., requiring more than seven days to form colonies), while the other two clades mainly contain rapidly-growing species requiring less than seven days to form colonies [10,24]. Based on the core genome proteins of mycobacterial species, a pairwise amino acid (aa) identity between these species, which provides a measure of the overall genetic relatedness of the species, was also determined for the *Mycobacterium* species. The results of these analyses also confirmed that the members from each of these five clades are more similar to each other than to the members of the other respective clades [25]. 

**Figure 1:**
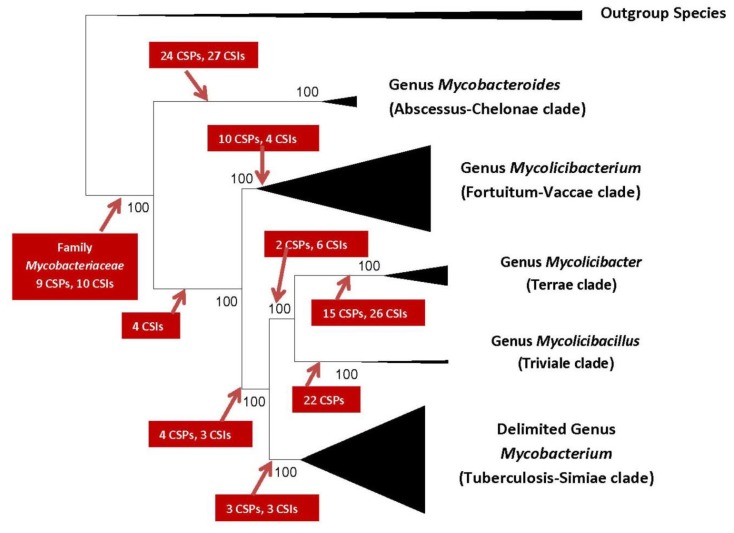
A compressed tree showing the main clades of mycobacteria observed in phylogenomic trees and molecular markers that have been identified for different clades. The tree shown is based on 1941 core proteins from the genomes of 150 *Mycobacteriaceae* species [25]. The terms CSIs and CSPs refer to conserved signature indels and conserved signature proteins, respectively, which are specific for the species from the observed clades. Comprehensive analyses of genome sequences have led to division of the family *Mycobacteriaceae* (genus *Mycobacterium*) into five different genera as indicated here [25].

In addition to these comprehensive phylogenomic studies, exhaustive comparative genomic analyses were also carried out on protein sequences from mycobacterial genomes to identify molecular signatures in the forms of CSIs and conserved signature proteins (CSPs) that are uniquely shared by either all members of the genus *Mycobacterium* or those exhibiting specificity for members of each of the five identified clades [16,30,31]. The importance of these molecular markers as useful tools for genetic and biochemical studies and for development of novel diagnostics and therapeutics will be discussed later. The results from these comparative genomic analyses have identified 172 molecular signatures consisting of CSIs and CSPs, which are distinctive characteristics of either all of the species from the family *Mycobacteriaceae* or which are specifically shared by different members of the five main clades of mycobacteria delineated by phylogenomic analyses. These molecular signatures provide strong independent evidence that the species from the five main observed clades of mycobacteria are genetically distinct from each other while also offering a reliable means for the demarcation of these groups in molecular terms. A summary diagram depicting the five main clades of mycobacteria and their interrelationships, which has emerged based on genomic analyses is presented in Figure 1 [25]. The numbers of identified molecular markers which are specific for different main clades as well various intermediate branch-points are also shown in this figure. 

Based on the compelling evidence amassed from different genomic scale analyses, all of which strongly supported the existence of the five main clades within the genus *Mycobacterium* as depicted in Figure 1, the genus *Mycobacterium* has now been divided into five different genera. In the new classification scheme for the family *Mycobacteriaceae*, the genus name *Mycobacterium* is limited to only members of the Tuberculosis-Simiae clade [25]. The delimited genus *Mycobacterium* continues to retain all of the major human and animal pathogenic species including *M. tuberculosis*, *M. leprae*, *M. bovis*, etc. The species belonging to the Fortuitum-Vaccae clade, which are primarily environmental species, are now placed into a new genus *Mycolicibacterium*, whereas the species from the Terrae and Triviale clades, which are also nonpathogenic, except occasional association with animal hosts or human patients, are now assigned to two new genera *Mycolicibacter* and *Mycolicibacillus*, respectively [25]. Lastly, the species from the Abscessus-Chelonae, some of which are associated with lung, skin and soft tissue infections are transferred into a new genus *Mycobacteroides* [25]. 

In the new classification scheme for mycobacteria, all of the major human and animal pathogenic species are retained in the delimited genus *Mycobacterium* and they are separated from other genera of mycobacterial species, which are comprised of species that are either non-pathogenic or are of lesser clinical significance [25]. With the explicit division of the mycobacterial species into these distinct groups or genera, attention can now be focused on the unique genetic and molecular characteristics that differentiate the members of these different groups of microbes. Although the new classification scheme represents a significant advancement in terms of clarifying the genetic diversity that exist within the family *Mycobacteriaceae*, two of the main genera comprising this family i.e., *Mycobacterium* and *Mycolicibacterium*, consisting respectively of the slow-growing and fast-growing mycobacterial species, are still very large and genetically diverse. Of these, the new genus *Mycobacterium* contains >70 species and within it a number of different species groups viz. “Tuberculosis complex”, “Avium complex”, “Gordonae clade”, “Kansasii clade”, “Simiae clade”, and a clade of mycolactone-producing mycobacteria, are informally recognized [10,17,19,24]. Additionally, a large number of species from this genus including *M. leprae*, are not part of any of these clades. As the genus *Mycobacterium* contains some of the most significant human and animal pathogens, it is necessary to obtain a more reliable understanding of the evolutionary relationships among this group of bacteria in order to identify characteristics that differentiate the tuberculosis causing bacteria from other members of this genus.

**Figure 2:**
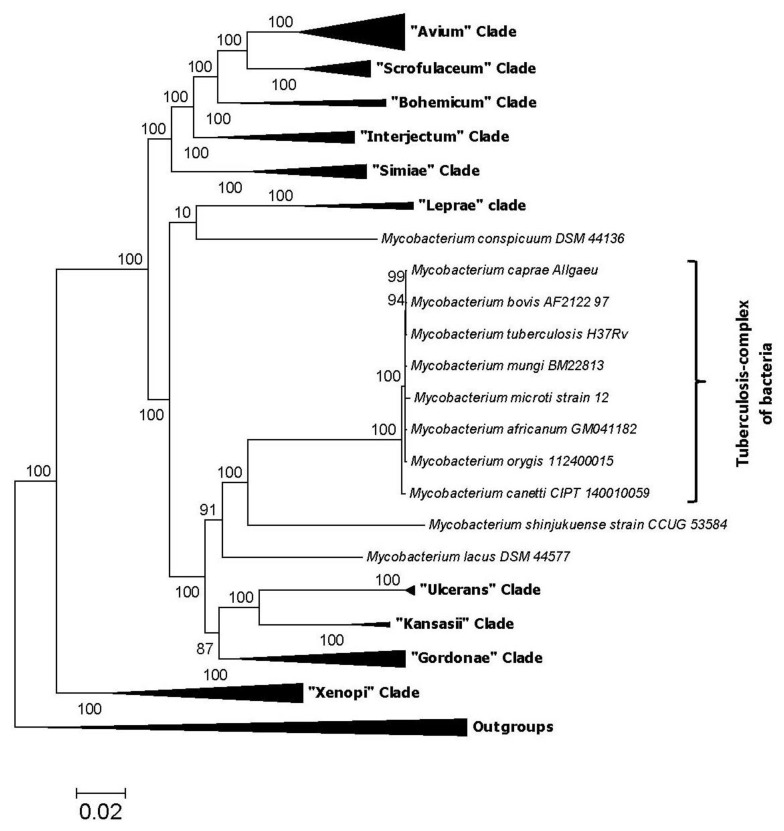
A compressed phylogenetic tree showing the main clades observed within the delimited genus *Mycobacterium* in a phylogenetic tree. The tree shown is based on 136 proteins commonly shared by members of the phylum Actinobacteria. The tree was constructed as described in earlier work [25] and the main species groupings observed are collapsed, except those from the *M. tuberculosis*-related group of bacteria. The group of species that is commonly referred to as the tuberculosis-complex is marked. All of the CSIs described in this work are specific for the tuberculosis-complex of bacteria.

Based on the branching patterns of species in different phylogenetic trees constructed in our earlier work [25], as well as additional phylogenomics and comparative genomics studies that we have carried out on these bacteria (to be described later), a good understanding of the evolutionary relationships among different species that comprise the new (delimited) genus *Mycobacterium* can now be acquired. Based on the results of these studies, at least 11 distinct species clades can be distinguished within the genus *Mycobacterium* based on their branching in phylogenetic trees and identified molecular signatures (Figure 2). A number of these species’ clades are similar to those indicated in earlier studies [10,24]. Of particular interest in the present context is the clade consisting of the tuberculosis-complex of species. The tuberculosis-complex consists of a tightly-clustered group of ten species (viz. *M. tuberculosis*, *Mycobacterium africanum*, *Mycobacterium bovis*, *Mycobacterium canettii*, *Mycobacterium caprae*, *Mycobacterium microti*, *Mycobacterium mungi*, *Mycobacterium orygis*, *Mycobacterium pinnipedii* and *Mycobacterium suricattae*), all of which are human or animal pathogens. Genome sequences are now available for most of the species from this group. In phylogenetic trees (see Figure 2), the tuberculosis-complex of species are separated from all other mycobacteria by a long branch and the species *Mycobacterium shinjukuense* and *Mycobacterium lacus*, which are rarely pathogenic [32,33], are indicated to be their closest relatives. In view of the long-branch (i.e., genetic distance) that separates the tuberculosis-complex of species from all other bacteria, it is expected that this group of bacteria should differ significantly from all other mycobacteria in terms of their genetic and biochemical characteristics. Hence, we have carried out detailed comparisons of the sequences for different proteins from the genomes of tuberculosis-complex of species and other mycobacteria to identify novel molecular signatures such as CSIs, which are specific for the *M. tuberculosis* complex of species. The genetic and biochemical significance of the CSIs and a summary of the results obtained from our comparative genomics studies are described below.

## 3. Genetic and Biological Significance of the Conserved Signature Indels

Conserved signature indels represent an important class of molecular markers, whose discovery has been enabled by the growing availability of genome sequences [25,28,31,34,35]. Although the inserts and deletions are commonly present in gene/protein sequences, only a small subset of these indels represent CSIs that are found to be useful for the different applications indicated here [29,31]. The indels that constitute CSIs are generally of fixed lengths, present at specific positions in particular genes/proteins, and are flanked on both sides by conserved regions to ensure that they constitute reliable characteristics [25,28,31,34,35]. As the CSIs in genes/proteins sequences result from rare genetic changes, when a particular CSI is specifically shared by a phylogenetically-related group of organisms, its presence is most parsimoniously accounted by the genetic change leading to the CSI occurring in a common ancestor of the group followed by vertical inheritance of this genetic change by other group members [25,28,31,34,35]. Based upon the presence or absence of a CSI in outgroup (i.e., ancestral) species, it is also possible to infer whether a given CSI represents an insertion or a deletion. Extensive earlier work on CSIs provides evidence that both large as well as small CSIs (even a one aa insert/deletion in protein sequence) are reliable molecular markers and they both exhibit a high degree of predictive ability to be present in other members of the indicated groups for which sequence information may be lacking [16,21,28,29,35,36,37]. In view of the reliability and exclusive presence of specific CSIs in a particular group of organisms, the CSIs now provide a dependable means for the demarcation of prokaryotic taxa of different ranks (ranging from genus to phylum) in molecular terms [25,28,29,38]. Furthermore, due to the specificity of the CSIs for a given group of organisms, the genetic/molecular changes introduced by them are predicted to be important for the CSI-containing organisms and this prediction has been confirmed experimentally for several studied CSIs [39,40,41,42]. As the genotype determines and controls the phenotype, the identified CSIs also provide important genetic/biochemical tools for discovering novel properties that are important to and uniquely shared by different groups of organisms for which these CSIs are specific. 

## 4. Conserved Signature Indels Specific for the *M. tuberculosis* Complex of Organisms

**Figure 3:**
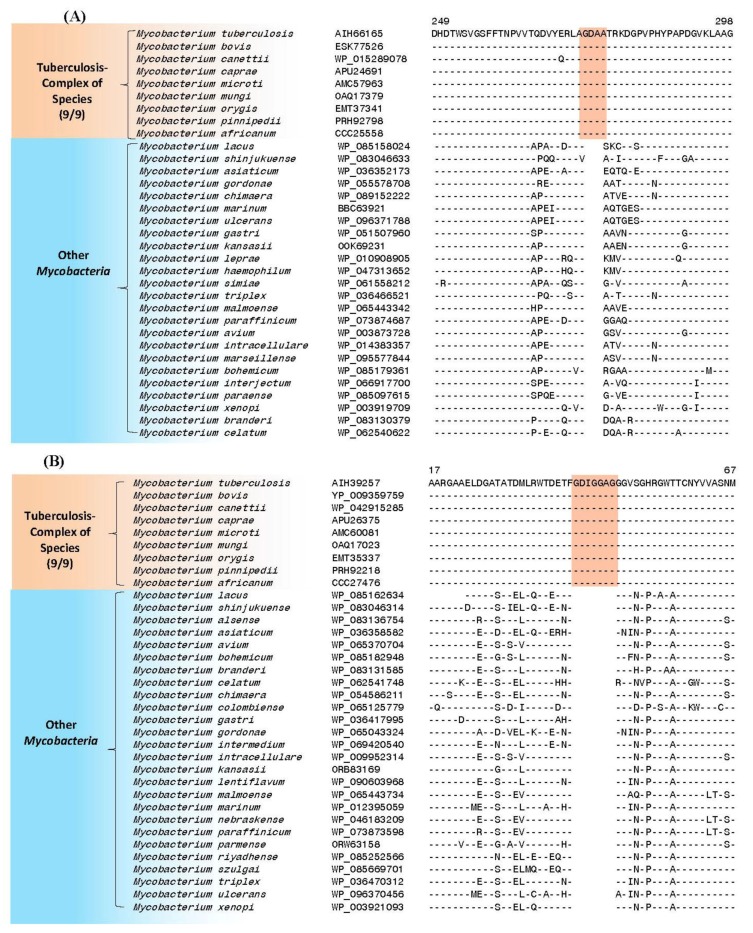
Partial sequence alignments of the proteins (**A**) UDP-*N*-acetylenolpyruvoyl-glucosamine reductase (MurB) and (**B**) 3′-phosphoadenosine 5′-phosphosulfate reductase (CysH), containing conserved inserts of four amino acid (aa) and seven aa (boxed), respectively, which are uniquely found in the tuberculosis-complex of bacteria. The numbers 9/9 indicate that there are 9 sequences available from the Tuberculosis-complex of bacteria and all 9 of them contain these CSIs. However, these CSIs are lacking in the homologs from all other mycobacteria as well as other examined bacteria. The homologs of these proteins, or the CSI-containing regions of these proteins, are not found in human. The dashes (-) in different sequence alignments show identity with the aa present on the top line. Mutational studies indicate that both these proteins are essential for the growth of *M. tuberculosis* [45,46].

**Figure 4:**
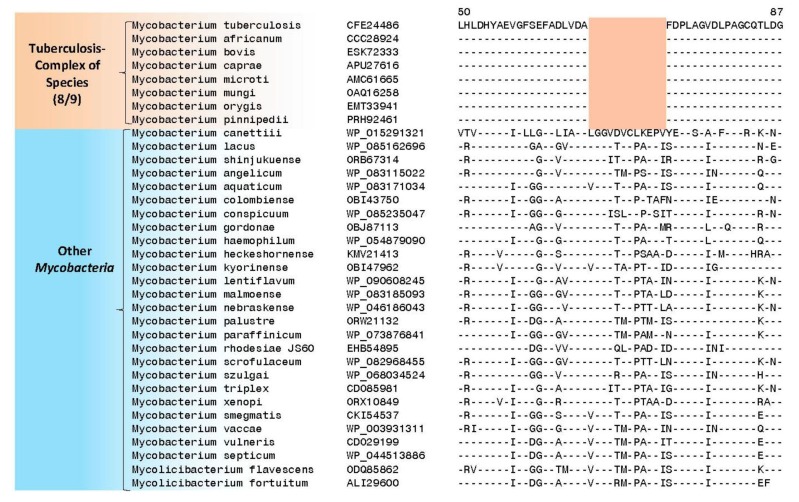
Partial sequence alignment of a LytR family transcriptional regulatory protein showing a 12 aa long deletion in a conserved region. This deletion is uniquely present in all other *M. tuberculosis* complex of organisms except *Mycobacterium canettii*, which branches earlier in comparison to the other species from this group (Figure 2). The dashes (-) indicate identity with the aa present on the top line.

The results of our comparative genomic studies on mycobacterial genomes have identified 63 CSIs in important proteins that are distinctive characteristics of the tuberculosis-complex of species. Sequence information for three of these CSIs are presented in Figure 3 and Figure 4. Figure 3A shows partial sequence alignment of the protein UDP-*N*-acetylenolpyruvoylglucosamine reductase (MurB), which plays an essential role in the biosynthesis of peptidoglycan in both Gram-positive and Gram-negative bacteria by catalyzing the formation of UDP-*N*-acetyl muramic acid from UDP- *N*-acetyl glucosamine [43,44]. In MurB protein, a four aa insert in a highly-conserved region is uniquely found in all nine sequenced species from the tuberculosis-complex of organisms, but it is not present in any other mycobacteria or actinobacteria. The results of Himar1-based transposon mutagenesis indicate that the *murB* gene is essential for in vitro growth of *M. tuberculosis* H37Rv [45,46]. As MurB has no known eukaryotic homologues, it provides a unique antibacterial target [44]. 

In Figure 3B, partial sequence alignment is presented of a protein annotated as putative 3′-phosphoadenosine 5′-phosphosulfate reductase (CysH). In this protein, a seven aa insertion in a conserved region is uniquely found in all nine sequenced *M. tuberculosis* complex of organisms, but not in any other mycobacteria. The CysH protein catalyzes the reduction of activated sulfate into sulfite and plays an important role in the sulfate activation pathway [47]. This protein is actively expressed in macrophages during the latent phase of infection with *M. tuberculosis* and appears to be required for the survival of tuberculosis bacteria in the macrophages. The gene for the CysH protein is also found to be essential for in vitro growth of H37Rv strain of *M. tuberculosis* [45,46]. Disruption of *cysH* gene in *M. tuberculosis* has been reported to cause auxotrophy for cysteine and methionine and attenuated virulence [48]. Although a homolog of the CysH protein is present in human, it lacks the N-terminal region of the protein, where this large insertion is found. Thus, the region of the protein shown in Figure 3B is not found in humans and the large insert present in this region is a specific characteristic of the *M. tuberculosis* complex of organisms. We also show in Figure 4 an example a protein where a 12 aa long deletion is present in a LytR family transcriptional regulatory protein in the *M. tuberculosis* related organisms. Interestingly, while this large CSIs is present in all other sequenced species from the *M. tuberculosis* complex (eight out of nine sequenced species), it is not present in *M. canettii*, which branches earlier in comparison to the other species from this complex in phylogenetic trees (Figure 2). Thus, this large deletion while providing a highly-specific molecular marker for the *M. tuberculosis* and its close relatives serves to differentiate them from *M. canettii*, which is also a part of the tuberculosis-complex of species.

In addition to the CSIs shown in Figure 3 and Figure 4, our comparative genomic analyses have identified 60 other CSIs in proteins involved in many diverse functions. A summary of some characteristics of these CSIs and the proteins in which they are found is shown in Table 1. Detailed sequence information for the three CSIs shown in Figure 3 and Figure 4 and the other 60 identified CSIs are provided in Figures S1–S63. As seen from Table 1, the identified CSIs are of different lengths and they are found in proteins involved in a broad range of cellular functions including cell wall synthesis, drug and ion transport, DNA replication, transcription and repair, protein translation, biosynthesis of cofactors and coenzymes such as ubiquinone and menaquinone, and proteins playing important roles in a variety of metabolic and regulatory pathways. In addition to the proteins with annotated cellular functions, a significant proportion of proteins harboring these CSIs are conserved proteins, with no information available regarding their cellular functions. The last column in Table 1 provides information based on Himar1-transposon mutagenesis studies indicating whether the genes for these proteins were found to be essential or nonessential for the in vitro growth of *M. tuberculosis* [45,46]. 

As seen from Table 1, the genes for a significant proportion (~20%) of the proteins, where these CSIs are found have been found to be essential for the in vitro growth/function of *M. tuberculosis* strain H37Rv [45,46]. Besides MurB and CysH, some of the other genes/proteins containing CSIs that are essential for the growth of *M. tuberculosis* include, MenE protein (an acetyl coenzyme A (acyl-CoA) synthetase (ligase) playing an essential role in menaquinone biosynthesis pathway [49,50], a putative adenosine triphosphate (ATP) dependent DNA ligase (Mt-Lig) [51], polyketide synthase protein (Pks8) involved in the biosynthesis of mycolic acid [52,53], NadE (nicotinamide adenine dinucleotide (NAD+) synthetase) protein catalyzing the last step of NAD biosynthesis [54], ribonuclease E playing an important role in RNA processing and decay [55] and folylpolyglutamate synthase (FPGS or FolC) protein, involved in the conversion of folates into polyglutamates derivatives [56,57]. Folate pathway is an established target for development of antimicrobials [58] and it is of interest to note that missense mutations within the dihydropteroate binding pocket of FolC confer resistance to *para*-aminosalicylic acid (PAS) in clinical isolates of *M. tuberculosis* and confer resistance to PAS, which is an important agent in the treatment of multidrug-resistant tuberculosis [56,57,58]. Some of the other proteins containing CSIs that are essential for the growth of *M. tuberculosis* include propionyl-CoA carboxylase subunit beta chain playing an essential role in the catabolic pathway of odd-chain fatty acids, isoleucine, threonine, methionine, and valine [59,60], putative phosphor-sugar (glucosamine) mutase involved in glycolysis and sugar metabolism [61], DNA topoisomerase I, TOPA (omega-protein) involved in relaxation of DNA [62], indolylacetylinositol arabinosyltransferase (EmbB), involved in the biosynthesis of cell wall arabinogalactan and lipoarabinomannan and a well-established target for the drug ethambutol [63,64], and guanosine triphosphatase (GTPase) Era which has intrinsic GTPase activity and is a regulator of cell growth [65].

**Figure 5:**
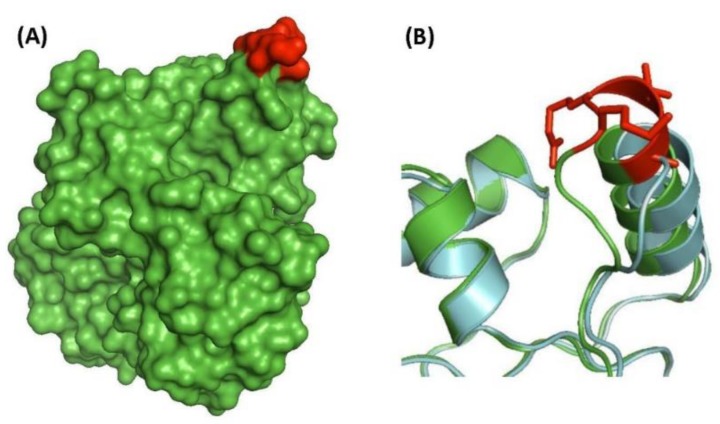
Structural localization of the CSI in the MurB protein. (**A**) Resolved structure of the UDP-*N*-acetylenolpyruvoylglucosamine reductase (MurB) protein from *M. tuberculosis* (PDB ID: 5JZX) [72]. The four aa insertion is highlighted in red. (**B**) A close up of the CSI region from *M. tuberculosis* proteins colored in green, and a homology model of the same protein from *Mycobacterium angelicum*, shown in cyan.

The CSIs in protein structures are generally located in surface-exposed loops of the proteins [39,40,66,67,68]. Structures of several proteins in which the CSIs have been identified in this work are now available for either *M. tuberculosis* or other organisms [69,70]. Based on the latter structures, structures of the corresponding *M. tuberculosis* proteins can be deduced using the homology modelling technique [71]. We have examined the structural locations of the two CSIs shown in Figure 2 in the corresponding proteins structures. For the MurB protein, its structure has been solved from *M. tuberculosis* (PDB ID: 5JZX) [72]. The four aa insertion in this protein which is specific for the *M. tuberculosis* complex of organisms (shown in red) forms a distinct lobe on the surface of the protein (Figure 5A). Using the structure of MurB protein from *M. tuberculosis* as a template, a homology model of this protein was also created for the *Mycobacterium angelicum homolog*, which lacks this CSI. The homology modelling was carried out as in our earlier work [39,66,73]. A close up of the structural comparison of the CSI-containing region for MurB from *M. tuberculosis* (colored in green) and *M. angelicum* (shown in cyan) is presented in Figure 5B. As seen, while the rest of the structures show nearly perfect overlap, the CSI in the *M. tuberculosis* protein (highlighted in red), extends an α-helix and forms part of a surface-exposed loop. 

**Figure 6:**
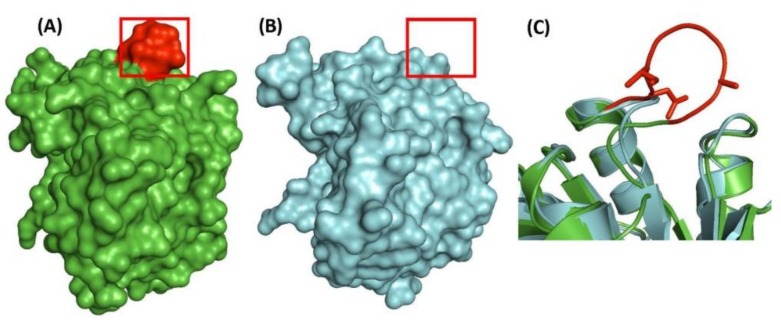
Structural location of the CSI in the CysH protein. (**A**) Homology model of the 3′-phosphoadenosine 5′-phosphosulfate reductase CysH protein from *M. tuberculosis* (based on PDB ID: 2GOY). The seven aa insertion is highlighted in red and boxed. (**B**) Resolved structure of the CysH protein from *Pseudomonas aeruginosa* (PDB ID: 2GOY). The region homologous to the insert is boxed. (**C**) A close-up of the CSI region in the aligned structures of the two proteins, with CSIs marked in red.

The structure of the CysH (3′-phosphoadenosine 5′-phosphosulfate reductase) protein from *M. tuberculosis* is not known. However, the structure of this protein has been solved from *Pseudomonas aeruginosa* (PDB ID: 2GOY) [74]. Based on the latter structure, a homology model of the CysH protein was created from *M. tuberculosis*. As the seven aa insertion is not present in the *P. aeruginosa* protein, it was modelled [39,66,73]. The structures of the modelled CysH protein from *M. tuberculosis* (Figure 6A), its solved structure from *P. aeruginosa* (Figure 6B), and a close up of the aligned structures for the CSI region from the two proteins, are shown in Figure 6. As seen from Figure 6C, the 7 aa CSI in the *M. tuberculosis* protein (shown in red) forms a surface-exposed loop/lobe in the protein structure (Figure 6A,C), that is lacking in the organisms not containing this CSI (Figure 6B).

## 5. Significance and Applications of the Tuberculosis-Complex Specific Conserved Signature Indels for Development of Novel Diagnostics and Therapeutics

Most studied CSIs exhibit a number of unique characteristics, which makes them potentially useful means for development of novel and specific diagnostic tests as well as potential targets for development of new classes of therapeutics. The usefulness of the CSIs for development of novel diagnostic tests is based on the observation that the CSIs are present in conserved regions of the genes/proteins and they exhibit a high degree of specificity for a given group of organisms [21,23,37,75,76]. Earlier work on CSIs provides compelling evidence that these molecular markers are highly specific characteristics of a given group of organisms [21,23,37,75,76]. Further, these molecular characteristics are not only specifically found in the available sequences from a given group of organisms, but that they also exhibit a high degree of predictive ability to be found in other group members for which sequence information is lacking at present [16,31,35,38]. In view of these characteristics, novel diagnostic tests for the detection of TB, based on the sequence regions harboring these CSIs, can be developed by means of different commonly used techniques e.g., polymerase chain reaction (PCR-based), real-time quantitative PCR (q-PCR-based), pyrosequencing, immunological or antibody-based methods, matrix-assisted laser desorption/ionization-time-of-flight (MALDI-TOF), aptamer-based methods, as well as in silico identification of the CSI-containing organisms in genomic and metagenomic sequences. As examples of the utility of CSIs for development of novel diagnostic tests, the CSIs specific for *Bacillus anthracis* and *Escherichia coli* O157/H7 have been successfully used for the development of highly-specific diagnostic tests for these important pathogens [75,76]. Although great advancements have been made in the detection of TB and multiple diagnostic tests are available for this purpose [2,3,77,78], most of these tests are either slow, lack specificity or are costly. Given that TB is most prevalent in low to middle income countries, there is a need for developing sensitive, specific and inexpensive tests for detection of TB [9,78].

Besides their presence in conserved regions and specificity for a particular group of organisms, most studied CSIs exhibit a number of other characteristics that make them potentially useful means for development of novel therapeutics. The first of these characteristics is that the CSIs in protein sequences are predicted to play important functional roles in the organisms in which they are found [41,42,66]. It has been experimentally shown for a number of CSIs that the removal of the CSIs from studied genes/proteins, or any significant changes in their sequences by genetic means affected/inhibited the growth of the CSI-containing organisms [35,41]. These studies also demonstrated that both large as well as small CSIs (even one aa insert or deletion) play important roles in the protein’s function [35,41]. Another important characteristic of the CSIs is that nearly all studied CSIs (including the examples described here) are located in the surface-exposed loops of the proteins, located away from the protein’s active or substrate binding sites [39,40,66]. Extensive earlier work indicates that the surface loops in protein sequences play important roles in facilitating novel protein-protein or protein-ligand interactions [39,66,67,68,79]. Based on the above observations, the most plausible explanation to account for the presence and functional roles played by different CSIs that are specific for the MTB-complex of organisms is that they are involved in mediating novel interactions (viz. protein-protein or protein-ligand) of the CSI-containing proteins in the MTB-complex of organisms [39,41,66,67,68,79]. It is postulated that these interactions, which are predicted to be specific for the MTB-complex of organisms, serve to differentiate these organisms from other mycobacteria which do not contain the CSIs. Further, it is suggested that the interactions mediated by the CSIs confer selective advantage to the MTB-complex of organisms in their natural habitat [41]. The selective advantages conferred by these interactions could include the ability to obtain nutrients in a nutrient-deficient environment, protection against host defense mechanisms, or breaching of the host defenses to enable its propagation (i.e. virulence). The blocking or inhibition of these interactions is thus expected to affect the growth or virulence of the MTB-complex organisms.

The above observations indicate that the CSI-containing regions of the *M. tuberculosis* proteins can also serve as potential means for the screening of novel compounds, which are specifically targeted towards this group of organisms. Since the CSIs are located in surface loops of the proteins, they provide readily accessible sites for binding of small molecules. Further as noted earlier, for most of the proteins which contain the *M. tuberculosis*-specific CSIs, their homologs are either not found in humans, or if they are present they generally do not contain the indicated CSIs. Based on these observations, it is hypothesized that screening for small molecules, which bind specifically to the CSI-containing regions of the *M. tuberculosis* proteins should lead to identification of a class of compounds which will interfere with the cellular functions of the CSIs, and such compounds would likely specifically affect the growth of the MTB-complex of organisms. Mutational studies indicate that only about 20–25% of the genes/proteins containing the CSIs specific for the MTB group of organisms are essential for the in vitro growth of *M. tuberculosis* (Table 1) [45,46]. However, growth of the cells *in vitro* does not provide an accurate measure of the growth of the organism in its natural habitat, which is impacted upon by many factors [80]. As the CSIs in all of the proteins described here are specific for the MTB group of organisms, it is likely that they all play specific roles in the functioning/survival of *M. tuberculosis* in the host environment by enabling functional interactions with other essential proteins/components. Thus, blocking the functions of CSIs in these other “non-essential” proteins by compounds that binds specifically to them is also expected to affect the growth or virulence of *M. tuberculosis* and related organisms within their hosts. 

To test the feasibility of using CSIs as possible drug targets, it is important to have the structural information for different *M. tuberculosis* proteins in which the identified CSIs are found. Although the structures of some *M. tuberculosis* proteins containing the CSIs are now available in the RCSB Protein Data Bank [69,70,81,82], further work on solving the structures of the proteins in which the large CSIs specific for the MTB-complex of organisms are found is necessary. Based on the structures of the proteins containing the CSIs as well as lacking the CSIs, the location of the CSI-containing regions in the protein structures can be reliably determined. High-throughput virtual screening of small molecule libraries can then be carried out to identify lead compounds [47,83,84] which bind with high affinity to only the CSI-containing regions in the proteins. Most of the work on drug discovery in the past, including structure-based drug design, has focused heavily on identification of compounds which inhibit growth by binding to the active sites of the proteins [7,8,9,50,85,86]. In contrast, identification of small molecules which inhibit cell growth or cellular function by interfering with the functions of the CSIs could represent a potentially new class of compounds that are active against the MTB-complex of organisms. Although the concept of using CSIs as possible drug-target has been suggested previously [87], the potential of these novel genetic/biochemical characteristics for new drug development remains unexplored.

## 6. Conclusions

Phylogenomic and comparative analyses of mycobacterial genomes sequences have provided important insights into the evolutionary relationships among this large and important group of microorganisms. The results reviewed here reveal that the TB-causing bacteria constitute a small group differing from all other mycobacteria in numerous regards. Comparative analyses of protein sequences from mycobacteria have identified 63 conserved signature indels in important proteins that are uniquely found in different members of the TB-complex of bacteria. Several characteristics of the CSIs reviewed here suggest that these novel genetic features could serve as an unexplored means for development of novel diagnostics and also as potential means for development of a new class of therapeutics specifically targeting the MTB-group of organisms. 

## Figures and Tables

**Table 1:** Summary of conserved signature indels (CSIs) that are specific for the tuberculosis complex.

Name	Gene Number (*M. tuberculosis* H37Rv)	Figure Number	Ins/Del	Location	Mutational Results #
putative UDP-*N*-acetylenolpyruvoyl-glucosamine reductase (MurB)	Rv0482	Figure 3A, Figure S1	4aa Ins	249–298	Essential
putative 3′-phosphoadenosine 5′-phosphosulfate reductase (CysH) (PAPS reductase, thioredoxin dep)	Rv2392	Figure 3B, Figure S2	7aa Ins	17–71	Essential (growth defect)
transcriptional regulator, LytR family	Rv3840	Figure 4, Figure S3	12aa Del	50–87	Non-essential
putative propionyl-CoA carboxylase beta chain 5 ACCD5 (PCCASE)	Rv3280	Figure S4	1aa Del	172–220	Essential
O-succinylbenzoic acid-CoA ligase MenE *	Rv0542c	Figure S5	2aa Ins	41–95	Essential
ligase *	Rv3712	Figure S6	4aa Ins	180–234	Essential
arabinosyltransferase EmbB *	Rv3795	Figure S7	3aa Ins	747–795	Essential
GTPase Era	Rv2364c	Figure S8	1aa Ins	225–283	Essential (growth defect)
primosome assembly protein PriA	Rv1402	Figure S9	3aa Ins	609–655	Essential
putative phospho-sugar mutase/MRSA homolog *	Rv3441c	Figure S10	3aa Ins	43–102	Essential
polyketide synthase Pks8	Rv1662	Figure S11	1aa Del	539–586	Non-essential
Glutamine-dependent NAD(+) synthetase	Rv2438c	Figure S12	1aa Del	584–641	Essential
ribonuclease E	Rv2444c	Figure S13	3aa Ins	219–269	Essential
putative folylpolyglutamate synthase protein (FolC)	Rv2447c	Figure S14	3aa Ins	111–170	Essential
DNA topoisomerase I TOPA (omega-protein)	Rv3646c	Figure S15	3aa Ins	392–440	Essential
metal cation transporting ATPase H	Rv0425c	Figure S16	1aa Del	963–1014	Non-essential
Acyltransferase *	Rv1565c	Figure S17	4aa Ins	162–220	Non-essential
α-amylase	Rv2471	Figure S18	1aa Del	428–477	Non-essential
hypothetical protein IQ48_14915, partial	Rv0897c	Figure S19	3aa Ins	257–306	Non-essential
hypothetical protein CAB90_01059 *	Rv0938	Figure S20	3aa Ins	422–469	Non-essential
transcriptional regulator *	Rv1186c	Figure S21	1aa Del	406–457	Non-essential
hypothetical protein IU12_21070	Rv0008c	Figure S22	4aa Ins	10–59	Non-essential
hypothetical protein IU14_19860	Rv0029	Figure S23	2aa Del	194–250	Non-essential
membrane protein	Rv0051	Figure S24	8aa Ins	470–522	Non-essential
hypothetical protein RN11_1864 *	Rv0094c	Figure S25	8aa Ins	18–67	Non-essential
transmembrane protein	Rv0188	Figure S26	3aa Del	18–55	Non-essential
hypothetical protein ERS181347_00724	Rv0209	Figure S27	3aa Ins	195–242	Non-essential
conserved membrane protein	Rv0210	Figure S28	3aa Ins	10–59	Non-essential
fructose-bisphosphate aldolase *	Rv0365c	Figure S29	4aa Ins	144–193	Non-essential
anti-sigma K factor	Rv0444c	Figure S30	1aa Ins	147–206	Non-essential
conserved protein of uncharacterised function % 2C possibly exported	Rv0518	Figure S31	3aa Ins	36–84	Non-essential
exonuclease V subunit α	Rv0629c	Figure S32	2aa Del	109–159	Non-essential
multidrug resistance protein EmrB	Rv0783c	Figure S33	3aa Del	320–366	Non-essential
Hypothetical protein ERS024213_05484	Rv0789c	Figure S34	1aa Del	39–91	Non-essential
LuxR family transcriptional regulator	RVBD_0890c	Figure S35	1aa Del	290–328	Non-essential
polyprenyl-diphosphate synthase GrcC	Rv0989c	Figure S36	3aa Del	205–251	Non-essential
polyprenyl-diphosphate synthase GrcC	Rv0989c	Figure S37	1aa Del	94–141	Non-essential
cold-shock protein	Rv1253	Figure S38	2aa Del	220–275	Non-essential
transcriptional regulator	Rv1358	Figure S39	1aa Ins	94–152	Non-essential
hypothetical protein IQ40_04435	Rv1359	Figure S40	4aa Ins	150–208	Non-essential
esterase	Rv1497	Figure S41	1aa Ins	296–344	Non-essential
hypothetical protein RN11_1864 *	Rv0094c	Figure S42	8aa Ins	165–213	Non-essential
DEAD/DEAH box helicase	Rv2092c	Figure S43	1aa Del	579–624	Non-essential
phosphoglycerate mutase	Rv2135c	Figure S44	1aa Del	01–48	Non-essential
hypothetical protein CAB90_02390	Rv2137c	Figure S45	2aa Ins	10–54	Non-essential
putative glycerol-3-phosphate dehydrogenase *	Rv2249c	Figure S46	4aa Ins	333–380	Non-essential
GTP-binding protein LepA	Rv2404c	Figure S47	3aa Ins	298–355	Non-essential
type I restriction/modification system specificity determinant HsdS	Rv2761c	Figure S48	4aa Ins	10–58	Non-essential
hypothetical protein IQ38_12515, partial	Rv2762c	Figure S49	2aa Del	42–81	Non-essential
polyketide synthase *	Rv2940c	Figure S50	3aa Ins	1311–1359	Non-essential
lipase	Rv2970c	Figure S51	1aa Ins	170–225	Non-essential
secreted protein	Rv3054c	Figure S52	1aa Del	13–61	Non-essential
DNA polymerase IV *	Rv3056	Figure S53	1aa Del	208–254	Non-essential
ATP-dependent DNA helicase *	Rv3202c	Figure S54	1aa Del	378–426	Non-essential
membrane protein	Rv3207c	Figure S55	1aa Ins	207–256	Non-essential
ATPase	Rv3220c	Figure S56	1aa Ins	215–269	Non-essential
DNA glycosylase *	Rv3297	Figure S57	4aa Ins	80–132	Non-essential
hypothetical protein IQ47_16905, partial *	Rv3394c	Figure S58	3aa Ins	78–123	Non-essential
hydrolase *	Rv3400	Figure S59	3aa Ins	141–186	Non-essential
hypothetical protein RN11_1864 *	Rv0094c	Figure S60	8aa Ins	19–68	Non-essential
acyl-CoA dehydrogenase FadE27	Rv3505	Figure S61	8aa Ins	162–211	Non-essential
oxidoreductase *	Rv3742c	Figure S62	11aa Del	25–65	Non-essential
hypothetical protein IQ42_20035 *	Rv3912	Figure S63	2aa Del	113–159	Non-essential

# Inferences whether the genes encoding different proteins are essential or not required for in vitro growth of *M. tuberculosis* H37Rv are based on the results from Himar1 based transposon mutagenesis reported in literature [45,46], * Some exceptions are seen for these CSIs.

## Supplementary Materials

The following are available online at http://www.mdpi.com/2571-5135/7/4/31/s1, Figure S1: Partial sequence alignment of a conserved region of the UDP-*N*-acetylenolpyruvoyl-glucosamine reductase (MurB) protein showing a four amino acid insertion that is specific for members of the “Tuberculosis” clade; Figure S2: Partial sequence alignment of a conserved region of the 3′-phosphoadenosine 5′-phosphosulfate reductase CYSH protein showing a seven amino acid insertion that is specific for members of the “Tuberculosis” clade; Figure S3: Partial sequence alignment of a conserved region of the transcriptional regulator protein showing a twelve amino acid deletion that is specific for members of the “Tuberculosis” clade; Figure S4: Partial sequence alignment of a conserved region of the propionyl-CoA carboxylase betachain 5 protein showing a one amino acid deletion that is specific for most members of the “Tuberculosis” clade and is absent form most other *Mycobacteriaceae*; Figure S5: Partial sequence alignment of a conserved region of the O-succinylbenzoic acid-CoA ligase MenE protein showing a two amino acid insertion that is specific for members of the “Tuberculosis” clade and absent in most other *Mycobacteriaceae*; Figure S6: Partial sequence alignment of a conserved region of a ligase protein showing a four aminoacid insertion that is specific for members of the “Tuberculosis” clade and absent from most other *Mycobacteriaceae*; Figure S7: Partial sequence alignment of a conserved region of the arabinosyltransferase protein showinga three amino acid insertion that is specific for members of the “Tuberculosis” clade and absent from most other *Mycobacteriaceae*; Figure S8: Partial sequence alignment of a conserved region of the GTPase Era protein showing a oneamino acid insertion that is specific for members of the “Tuberculosis” clade; Figure S9: Partial sequence alignment of a conserved region of the primosome assembly protein PriA showing a three amino acid insertion that is specific for members of the “Tuberculosis” clade; Figure S10: Partial sequence alignment of a conserved region of the phospho-sugar mutase/MRSAprotein showing a three amino acid insertion that is specific for members of the “Tuberculosis” clade and absent from most other *Mycobacteriaceae*; Figure S11: Partial sequence alignment of a conserved region of the polyketide synthase Pks8 protein showing a one amino acid deletion that is specific for members of the “Tuberculosis” clade; Figure S12: Partial sequence alignment of a conserved region of the Glutamine-dependent NAD(+) synthetase protein showing a one amino acid deletion that is specific for members of the “Tuberculosis” clade; Figure S13: Partial sequence alignment of a conserved region of the ribonuclease E protein showing a three amino acid insertion that is specific for members of the “Tuberculosis” clade; Figure S14: Partial sequence alignment of a conserved region of the ribonuclease E protein showing a three amino acid insertion that is specific for members of the “Tuberculosis” clade; Figure S15: Partial sequence alignment of a conserved region of the DNA topoisomerase I protein showing a three amino acid insertion that is specific for members of the “Tuberculosis” clade; Figure S16: Partial sequence alignment of a conserved region of the metal cation transporting ATPaseH protein showing a one amino acid deletion that is specific for members of the “Tuberculosis” clade; Figure S17: Partial sequence alignment of a conserved region of an acyltransferase protein showing afour amino acid insertion that is specific for members of the “Tuberculosis” clade and absent from most other *Mycobacteriaceae*; Figure S18: Partial sequence alignment of a conserved region of an alpha-amylase protein showing aone amino acid deletion that is specific for members of the “Tuberculosis” clade; Figure S19: Partial sequence alignment of a conserved region of the hypothetical proteinIQ48_14915 showing a three amino acid insertion that is specific for members of the “Tuberculosis” clade; Figure S20: Partial sequence alignment of a conserved region of the hypothetical proteinCAB90_01059 showing a three amino acid insertion that is specific for members of the “Tuberculosis” clade and is absent from most other *Mycobacteriaceae*; Figure S21: Partial sequence alignment of a conserved region of the transcriptional regulator proteinshowing a one amino acid deletion that is specific for members of the “Tuberculosis” clade and is absent from most other *Mycobacteriaceae*; Figure S22: Partial sequence alignment of a conserved region of the hypothetical protein IU12_21070 showing a four amino acid insertion that is specific for members of the “Tuberculosis” clade; Figure S23: Partial sequence alignment of a conserved region of the hypothetical protein IU14_19860 showing a two amino acid deletion that is specific for members of the “Tuberculosis” clade; Figure S24: Partial sequence alignment of a conserved region of amembrane protein showing an eight amino acid insertion that is specific for members of the “Tuberculosis” clade; Figure S25: Partial sequence alignment of a conserved region of the hypothetical protein RN11_1864 showing an eight amino acid insertion that is specific for most members of the “Tuberculosis” clade and is absent from most other *Mycobacteriaceae*; Figure S26: Partial sequence alignment of a conserved region of a transmembrane protein showing a three amino acid deletion that is specific for members of the “Tuberculosis” clade; Figure S27: Partial sequence alignment of the hypothetical protein ERS181347_00724 showing a three amino acid insertion that is specific for members of the “Tuberculosis” clade; Figure S28: Partial sequence alignment of a conserved membrane protein showing a three amino acid insertion that is specific for members of the “Tuberculosis” clade; Figure S29: Partial sequence alignment of the fructose-bisphosphate aldolase protein showing a four amino acid insertion that is specific for members of the “Tuberculosis” clade and is absent from most other *Mycobacteriaceae*; Figure S30: Partial sequence alignment of the fructose-bisphosphate aldolase protein showing a four amino acid insertion that is specific for members of the “Tuberculosis” clade and is absent from most other *Mycobacteriaceae*; Figure S31: Partial sequence alignment of a conserved protein showing a three amino acid insertion that is specific for members of the “Tuberculosis” clade; Figure S32: Partial sequence alignment of the exonuclease V subunit alpha protein showing a two amino acid deletion that is specific for members of the “Tuberculosis” clade; Figure S33: Partial sequence alignment of the multidrug resistance protein EmrB showing a three amino acid deletion that is specific for members of the “Tuberculosis” clade; Figure S34: Partial sequence alignment of the Hypothetical protein ERS024213_05484 showing a one amino acid deletion that is specific for members of the “Tuberculosis” clade; Figure S35: Partial sequence alignment of the LuxR family transcriptional regulator protein showing a one amino acid deletion that is specific for members of the “Tuberculosis” clade; Figure S36: Partial sequence alignment of the polyprenyl-diphosphate synthase GrcC protein showing a three amino acid deletion that is specific for members of the “Tuberculosis” clade; Figure S37: Partial sequence alignment of the polyprenyl-diphosphate synthase GrcC protein showing a one amino acid deletion that is specific for members of the “Tuberculosis” clade and is absent from most other *Mycobacteriaceae*; Figure S38: Partial sequence alignment of a cold-shock protein showing a two amino acid deletion that is specific for members of the “Tuberculosis” clade; Figure S39: Partial sequence alignment of a transcriptional regulator protein showing a one amino acid insertion that is specific for members of the “Tuberculosis” clade; Figure S40: Partial sequence alignment of the hypothetical protein IQ40_04435 showing a four amino acid insertion that is specific for members of the “Tuberculosis” clade; Figure S41: Partial sequence alignment of an esterase protein showing a one amino acid insertion that is specific for members of the “Tuberculosis” clade; Figure S42: Partial sequence alignment of the hypothetical protein RN11_1864 showing an eight amino acid insertion that is specific for most members of the “Tuberculosis” clade and is absent from most other *Mycobacteriaceae*; Figure S43: Partial sequence alignment of a DEAD/DEAH box helicase protein showing a one amino acid deletion that is specific for members of the “Tuberculosis” clade; Figure S44: Partial sequence alignment of a phosphoglycerate mutase protein showing a one amino acid deletion that is specific for members of the “Tuberculosis” clade; Figure S45: Partial sequence alignment of a hypothetical protein CAB90_02390 showing a two amino acid insertion that is specific for members of the “Tuberculosis” clade; Figure S46: Partial sequence alignment of the glycerol-3-phosphate dehydrogenase protein showing a four amino acid insertion that is specific for members of the “Tuberculosis” clade and is absent from most other *Mycobacteriaceae*; Figure S47: Partial sequence alignment of the GTP-binding protein LepA showing a three amino acid insertion that is specific for members of the “Tuberculosis” clade; Figure S48: Partial sequence alignment of the type I restriction/modification system specificity determinant HsdS protein showing a four amino acid insertion that is specific for members of the “Tuberculosis” clade; Figure S49: Partial sequence alignment of the hypothetical protein IQ38_12515 showing a two amino acid deletion that is specific for members of the “Tuberculosis” clade; Figure S50: Partial sequence alignment of the polyketide synthase protein showing a three amino acid insertion that is specific for most members of the “Tuberculosis” clade; Figure S51: Partial sequence alignment of a lipase protein showing a one amino acid insertion that is specific for members of the “Tuberculosis” clade; Figure S52: Partial sequence alignment of a secreted protein showing a one amino acid deletion that is specific for members of the “Tuberculosis” clade; Figure S53: Partial sequence alignment of the DNA polymerase IV protein showing a one amino acid deletion that is specific for most members of the “Tuberculosis” clade and is absent from most other *Mycobacteriaceae*; Figure S54: Partial sequence alignment of an ATP-dependent DNA helicase protein showing a one amino acid deletion that is specific for members of the “Tuberculosis” clade and is absent from most other *Mycobacteriaceae*; Figure S55: Partial sequence alignment of an ATP-dependent DNA helicase protein showing a one amino acid deletion that is specific for members of the “Tuberculosis” clade and is absent from most other *Mycobacteriaceae*; Figure S56: Partial sequence alignment of an ATPase protein showing a one amino acid insertion that is specific for members of the “Tuberculosis” clade; Figure S57: Partial sequence alignment of a DNA glycosylase protein showing a four amino acid insertion that is specific for members of the “Tuberculosis” clade and is absent from most other *Mycobacteriaceae*; Figure S58: Partial sequence alignment of the hypothetical protein IQ47_16905 showing a three amino acid insertion that is specific for most members of the “Tuberculosis” clade and is absent from most other *Mycobacteriaceae*; Figure S59: Partial sequence alignment of a hydrolase protein showing a three amino acid insertion that is specific for members of the “Tuberculosis” clade and is absent from most other *Mycobacteriaceae*; Figure S60: Partial sequence alignment of the hypothetical protein RN11_1864 showing an eight amino acid insertion that is specific for most members of the “Tuberculosis” clade and is absent from most other *Mycobacteriaceae*; Figure S61: Partial sequence alignment of the acyl-CoA dehydrogenase FadE27 protein showing an eight amino acid insertion that is specific for members of the “Tuberculosis” clade; Figure S62: Partial sequence alignment of an oxidoreductase protein showing an eleven amino acid deletion that is specific for most members of the “Tuberculosis” clade; Figure S63: Partial sequence alignment of the hypothetical protein IQ42_20035 showing a two amino acid deletion that is specific for most members of the “Tuberculosis” clade.
