# Supplementary material for: Impact of Genomics on Clarifying the Evolutionary Relationships amongst Mycobacteria: Identification of Molecular Signatures Specific for the Tuberculosis-Complex of Bacteria with Potential Applications for Novel Diagnostics and Therapeutics
Source: High Throughput. 2018 Oct 2;7(4):31. doi: 10.3390/ht7040031 (PMC6306742; doi:10.3390/ht7040031)
Supplement: Supplementary file 1 [file high-throughput-07-00031-s001.pdf]

			249		298
**"Tuberculosis" clade (9/9)**	*Mycobacterium tuberculosis*	AIH66165	DHDTWSVGSFFTNPVVTQDVYERLA	GDAA	TRKDGPVPHYPAPDGVKLAAG
	Mycobacterium bovis	ESK77526	-------------------------	----	---------------------
	Mycobacterium canettii	WP_015289078	---------------------Q---	----	---------------------
	Mycobacterium caprae	APU24691	-------------------------	----	---------------------
	Mycobacterium microti	AMC57963	-------------------------	----	---------------------
	Mycobacterium mungi	OAQ17379	-------------------------	----	---------------------
	Mycobacterium orygis	EMT37341	-------------------------	----	---------------------
	Mycobacterium pinnipedii	PRH92798	-------------------------	----	---------------------
	Mycobacterium africanum	CCC25558	-------------------------	----	---------------------
**Other *Mycobacteriaceae* (0/62)**	*Mycobacterium angelicum*	WP_083113123	----------------APE------		S-M-----N------I-----
	Mycobacterium arosiense	WP_083066530	---------------IAP-A-----		AAV-----N------------
	Mycobacterium asiaticum	WP_036352173	----------------APE--A---		EQTQ-E---------------
	Mycobacterium avium	WP_003873728	----------------AP-------		GSV----------G-------
	Mycobacterium avium subsp. avium	EUA28749	----------------APE------		ATV-----N------------
	Mycobacterium avium subsp. paratuberculosis	ETB42591	----------------AP-------		GSV----------G-------
	Mycobacterium avium subsp. silvaticum	ETB04819	----------------AP-------		GSV----------G-------
	Mycobacterium bohemicum	WP_085179361	----------------AP-----V-		RGAA-------------M---
	Mycobacterium branderi	WP_083130379	----------------P----Q---		DQA-R----------------
	Mycobacterium celatum	WP_062540622	----------------P-E--Q---		DQA-R-------A--------
	Mycobacterium chimaera	WP_089152222	----------------AP-------		ATVE----N------------
	Mycobacterium colombiense	WP_007768283	----------------AP---Q---		ASV-----N------------
	Mycobacterium conspicuum	WP_085235610	----------------AAE------		G-V--Q---------------
	Mycobacterium europaeum	WP_085240816	----------------APE------		AGFE-----------------
	Mycobacterium florentinum	WP_085222774	----------------APE---S--		G-T-----N------------
	Mycobacterium fragae	WP_085197663	----------------APEF-----		Q-AER--------A-------
	Mycobacterium gastri	WP_051507960	----------------SP-------		AAVN---------G-------
	Mycobacterium gastri 'Wayne'	ETW24530	----------------SP-------		AAVN---------G-------
	Mycobacterium genavense	WP_025737032	-----------------PQ---S--		A-T-----N------------
	Mycobacterium gordonae	WP_055578708	-----------------RE------		AAT-----N------------
	Mycobacterium haemophilum	WP_047313652	----------------AP---HQ--		KMV------------------
	Mycobacterium heckeshornense	WP_048889567	---------------------Q-V-		D-A------W-T-G-------
	Mycobacterium heidelbergense	WP_083074492	----------------PPG------		DSVE-----------------
	Mycobacterium interjectum	WP_066917700	----------------SPE------		A-VQ-----------I-----
	Mycobacterium intermedium	WP_069421516	----------------PPE-F----		A-V------------------
	Mycobacterium intracellulare	WP_014383357	----------------APE------		ATV-----N------------
	Mycobacterium kansasii	OOK69231	----------------AP-------		AAEN---------G-------
	Mycobacterium kyorinense	WP_065014685	----------------APE--Q---		DEA-R----------------
	Mycobacterium lacus	WP_085158024	----------------APA--D---		SKC--S---------------
	Mycobacterium lentiflavum	WP_090599765	----------------APE---S--		G-T-----N------------
	Mycobacterium leprae	WP_010908905	----------------AP---RQ--		KMV---------Q--------
	Mycobacterium lepraemurium	ATA29694	----------------AP-------		GSV--------T-G-------
	Mycobacterium liflandii	WP_015354460	----------------APEI-----		AQTGES---------------
	Mycobacterium malmoense	WP_065443342	----------------HP-------		AAVE-----------------
	Mycobacterium mantenii	WP_083095025	----------------AP-------		AAV------------------
	Mycobacterium marinum	BBC63921	----------------APEI-----		AQTGES---------------
	Mycobacterium marseillense	WP_095577844	----------------AP-------		ASV-----N------------
	Mycobacterium montefiorense	WP_108926083	----------------APG---S--		A-T-----N------------
	Mycobacterium palustre	WP_085077552	----------------SP-A-----		G-L-----------------A
	Mycobacterium paraense	WP_085097615	----------------SPQE-----		G-VE-----------I-----
	Mycobacterium paraffinicum	WP_073874687	----------------APE--D---		GGAQ-----------------
	Mycobacterium paraseoulense	WP_083172373	----------------APE------		GAA------------------
	Mycobacterium parmense	WP_085271789	----------------AP--C----		DTI------------------
	Mycobacterium persicum	WP_083154566	------A---------AP-------		AAV----------G-------
	Mycobacterium pseudoshottsii	WP_086085513	----------------APEI-----		AQTGES---------------
	Mycobacterium riyadhense	WP_085248323	----------------GP---Q---		-DM-----N----N-------
	Mycobacterium saskatchewanense	WP_085258717	----------------SAEA-----		GAVE-----------------
	Mycobacterium scrofulaceum	WP_067282226	----------------APE--D---		GGV------------------
	Mycobacterium shigaense	WP_096437122	-----------------PEA-----		A-A------W---G-------
	Mycobacterium shimoidei	WP_069397557	----------------PGE--Q---		Q- ----------------
	Mycobacterium shinjukuense	WP_083046633	-----------------PQQ----V		A-I------F---GA------
	Mycobacterium simiae	WP_061558212	-R--------------APA--QS--		G-V----------A-------
	Mycobacterium sphagni	WP_094482039	----------------PAERF-D-K		S-R----------G-------
	Mycobacterium szulgai	ORX12783	----------------APE------		S-M-----N----G-I-----
	Mycobacterium triplex	WP_036466521	-----------------PQ---S--		A-T-----N------------
	Mycobacterium ulcerans	WP_096371788	----------------APEI-----		AQTGES---------------
	Mycobacterium vulneris	WP_085288096	----------------PP-------		ASV-E---N------------
	Mycobacterium xenopi	WP_003919709	---------------------Q-V-		D-A------W---G-I-----
	Mycolicibacterium aurum	WP_048632310	-----------------RAEFD---		AEV--T---------------

Figure S1. Partial sequence alignment of a conserved region of the UDP-N-acetylenolpyruvoyl-glucosamine reductase (MurB) protein showing a four amino acid insertion that is specific for members of the "Tuberculosis" clade. The alignment shown here is the same as the one in Figure 3 (A), but it contains information for more outgroup species.

Group	Species	Accession	17	Insertion	71
"Tuberculosis" clade (9/9)	*Mycobacterium tuberculosis*	AIH39257	AARGAAELDGATATDMLRWTDETF	GDIGGAG	GGVSGHRGWTTCNYVVASNMADAV
	Mycobacterium bovis	YP_009359759	------------------------	-------	------------------------
	Mycobacterium canettii	WP_042915285	------------------------	-------	------------------------
	Mycobacterium caprae	APU26375	------------------------	-------	------------------------
	Mycobacterium microti	AMC60081	------------------------	-------	------------------------
	Mycobacterium mungi	OAQ17023	------------------------	-------	------------------------
	Mycobacterium orygis	EMT35337	------------------------	-------	------------------------
	Mycobacterium pinnipedii	PRH92218	------------------------	-------	------------------------
	Mycobacterium africanum	CCC27476	------------------------	-------	------------------------
Other *Mycobacteriaceae* (0/62)	*Mycobacterium alsense*	WP_083136754	--------R--S---L------N-		---N-P---A--------S-QE--
	Mycobacterium angelicum	WP_083114401	-----------S--EL-Q--E---		---N-P---A----------Q-G-
	Mycobacterium arosiense	WP_083062943	--------E--S-A-V--------		--IN-P---A--------S-QE--
	Mycobacterium asiaticum	WP_036358582	--------E--D--EL-Q--ERH-		-NIN-P---A----------Q---
	Mycobacterium avium	WP_065370704	--------E--S-S-V--------		---N-P---A--------S-QE--
	Mycobacterium bohemicum	WP_085182948	--------E--G-S-L------N-		--FN-P---A--------S-QE--
	Mycobacterium branderi	WP_083131585	--------E--S---L------N-		---H-P--AA----------Q---
	Mycobacterium celatum	WP_062541748	-----K--E--S--EL-----HH-		R--NVP---A--GW----S-Q---
	Mycobacterium chimaera	WP_054586211	---S----E--S--EL------N-		---N-P---A--------S-Q---
	Mycobacterium colombiense	WP_065125779	-Q---------S-D-I-----D--		---D-P-S-A--KW---C--QE--
	Mycobacterium conspicuum	WP_085235420	-----------D--EL----E-N-		---N-P---A----------Q---
	Mycobacterium europaeum	WP_085240287	--------E--S--EV--------		--LN-P---A-----LT-S-QE--
	Mycobacterium florentinum	WP_085220670	--------E--S--EL------N-		--IN-P---A----I-----Q---
	Mycobacterium fragae	WP_085199625	-E------E--S-I-L------N-		---R-P-ASA----------Q---
	Mycobacterium gastri	WP_036417995	-----D-----S---L-----AH-		---N-P---A----------QE--
	Mycobacterium genavense	WP_025736051	-T------E--S--EL------N-		--IN-P---A----------Q---
	Mycobacterium gordonae	WP_065043324	--------A--D-VEL-K--E-N-		-NIN-P---A----------Q---
	Mycobacterium heckeshornense	WP_048893314	-----------S--EL-Q------		---N-P---A----------Q---
	Mycobacterium heidelbergense	WP_083072076	--------V--S---L----E-N-		---N-P---A--------------
	Mycobacterium interjectum	WP_066913646	-E------E--G---I---A-DH-		A--G-P-P-AN-R--I----Q---
	Mycobacterium intermedium	WP_069420540	--------E--N---L----E-N-		---N-P---A----------Q---
	Mycobacterium intracellulare	WP_009952314	--------E--S-S-V--------		---N-P---A--------S-QE--
	Mycobacterium kansasii	ORB83169	-----------G---L--------		---N-P---A--------------
	Mycobacterium kyorinense	WP_065015558	--------A--S-S-L------N-		---N-P--AA----------Q---
	Mycobacterium lacus	WP_085162634	-----S--EL-Q--E---		---N-P-A-A--------------
	Mycobacterium lentiflavum	WP_090603968	--------E--S---L------N-		--IN-P---A----------Q---
	Mycobacterium lepraemurium	ATA28936	--------E--S-S-V--------		---N-P---A--------S-QE--
	Mycobacterium liflandii	WP_015356308	-------ME--S---L---A--H-		--IN-P---A----------Q---
	Mycobacterium malmoense	WP_065443734	--------E--S--EV--------		--AQ-P---A-----LT-S-QE--
	Mycobacterium marinum	WP_012395059	-------ME--S---L---A--H-		--IN-P---A----------Q---
	Mycobacterium montefiorense	WP_108925415	-T------E--S-DEL----E-H-		---N-P---A----------Q---
	Mycobacterium nebraskense	WP_046183209	--------E--S--EV--------		---N-P---A-----LT-S-QE--
	Mycobacterium noviomagense	WP_083087538	-----------S---L------H-		---G-P---A--KW------Q---
	Mycobacterium palustre	WP_085079959	--------E--S--EL----E-N-		---N-P---A----------Q---
	Mycobacterium paraense	WP_085104226	--------E--S---L-----DH-		--IN-P---A--------S-QE--
	Mycobacterium paraffinicum	WP_073873598	--------R--S--EV--------		---N-P---A-----LT-S-QE--
	Mycobacterium paraintracellulare	WP_014384197	--------E--S-S-V--------		---N-P---A--------S-QE--
	Mycobacterium paraseoulense	WP_083168877	-----D--E--S--EV-----RI-		---N-P---A-----LT-S-QE--
	Mycobacterium parmense	ORW63158	-----V--E--G-A-V------H-		---N-P---A--------S-QE--
	Mycobacterium riyadhense	WP_085252566	-----------N--EL-E--EQ--		---N-P---A----------Q---
	Mycobacterium saskatchewanense	WP_085254789	--E-----E--S-S-L------H-		---N-P---A--------S-QE--
	Mycobacterium scrofulaceum	WP_067282947	--------E--S--EV--------		---N-P---A-----LT-S-QE--
	Mycobacterium sherrisii	WP_069398943	-----------N-EEL----E-N-		---N-P---A----------Q---
	Mycobacterium shigaense	WP_096441322	-----V--E--S-DEL-----RN-		---N-P---A--Q-------Q---
	Mycobacterium shimoidei	WP_069394613	-------M---S-S-L------N-		---H-P--AA----------Q---
	Mycobacterium shinjukuense	WP_083046314	------D----S-IEL-Q--E-N-		---N-P---A--------------
	Mycobacterium szulgai	WP_085669701	-----------S--ELMQ--EQ--		---N-P---A----------Q---
	Mycobacterium talmoniae	WP_071027060	-----------N--EL------H-		---H-P--AA----L-----Q---
	Mycobacterium triplex	WP_036470312	--------E--S--EL------N-		--IN-P---A----------Q---
	Mycobacterium ulcerans	WP_096370456	-------ME--S---L-C-A--H-		A-IN-P---A----------Q---
	Mycobacterium xenopi	WP_003921093	-----------S--EL-Q------		---N-P---A----------Q---
	Mycolicibacillus trivialis	WP_085108897	-----------D--EL------H-		---N-P--GA--------------
	Mycolicibacter algericus	WP_083036313	--K-----------EL-E--EAN-		---N-P--GA--------------
	Mycolicibacter heraklionensis	WP_065040279	--K--------S--EL-E---AN-		---N-P--GA--------------
	Mycolicibacter hiberniae	WP_085134780	--K--------S-REL-E---TH-		GGPGGSAAD-------------
	Mycolicibacter icosiumassiliensis	WP_067971646	--K--------S--EL-E---AN-		---N-P--GA--------------
	Mycolicibacter longobardus	WP_085263510	--K--------S--EL-E---TH-		---N-P--GA--------------
	Mycolicibacter senuensis	WP_085083598	--K-----------EL-E--EAN-		---N-P--GA--------------
	Mycolicibacter sinensis	WP_013828454	--K-----------EL-E--EAN-		---N-P--GA--------------
	Mycolicibacter terrae	WP_085259646	-----------S--EL-E---AN-		---R-P--GA--------------

Figure S2. Partial sequence alignment of a conserved region of the 3'-phosphoadenosine 5'-phosphosulfate reductase CYSH protein showing a seven amino acid insertion that is specific for members of the "Tuberculosis" clade. The alignment shown here is the same as the one in Figure 3 (B), but it contains information for more outgroup species.

			50		87
"Tuberculosis" clade (8/8)	*Mycobacterium tuberculosis*	CFE24486	LHLDHYAEVGFSEFADLVDA		FDPLAGVDLPAGCQTLDG
	Mycobacterium bovis	ESK72333	--------------------		------------------
	Mycobacterium caprae	APU27616	--------------------		------------------
	Mycobacterium microti	AMC61665	--------------------		------------------
	Mycobacterium mungi	OAQ16258	--------------------		------------------
	Mycobacterium orygis	EMT33941	--------------------		------------------
	Mycobacterium pinnipedii	PRH92461	--------------------		------------------
	Mycobacterium africanum	CCC28924	--------------------		------------------
Other *Mycobacteriaceae* (0/70)	*Mycobacterium angelicum*	WP_083115022	-R----------G--V----	LGGVTMCPSEPI	S-----IN------Q---
	Mycobacterium aquaticum	WP_083171034	--------I--GG-------	VGGVTVCPAEPI	S-----I-------Q---
	Mycobacterium asiaticum	OBI87779	-R------I--AG--V----	LGGVTVCPTEPV	R-----L-------K---
	Mycobacterium colombiense	OBI43750	-R------I--GG--A----	LGGVTVCPKTAF	N-----IE--------N-
	Mycobacterium conceptionense	CQD02314	--------I--DG--G----	VGGVRMCPAEPI	S-----IE------E---
	Mycobacterium conspicuum	WP_085235047	-R------I--GG--G----	LGGISLCLPESI	T-----I-------R-N-
	Mycobacterium dioxanotrophicus	WP_087081487	--------I--GG-------	VGGVTVCPAEPI	S-----I-------Q---
	Mycobacterium florentinum	ORV49668	-R------I--AG--V----	LGGITVCPTAPI	D-----I-------K-N-
	Mycobacterium gordonae	OBJ87113	-----------AG--V----	LGGVTVCPAEPM	R-----L--Q----R---
	Mycobacterium grossiae	WP_070356433	-R------I--DG--E----	VGGVTMCPAEPI	S-----I-------E---
	Mycobacterium haemophilum	WP_054879090	--------I---G--G----	LGGVTVCPAEPV	T-----L-------Q---
	Mycobacterium heckeshornense	KMV21413	-R----V-----G--S----	LGGVTVCPSAAV	D-----I-M----HRA--
	Mycobacterium heidelbergense	ORA67140	-R------I--GG-------	LGGVTMCPTMPI	E-----I-------E---
	Mycobacterium houstonense	WP_066902093	-R------I--DG--A----	VGGVTMCPAEPI	N-----I-------E---
	Mycobacterium iranicum	WP_064282900	MR------I--GG-------	VGGVTMCPTEPI	N-----I-------E---
	Mycobacterium kyorinense	OBI47962	-R----V-----G--V----	VGGVTACPTEPI	D-----IG----------
	Mycobacterium lacus	WP_085162696	-R---------GA--GV---	LGGVTVCPAEPI	S-----I-------N-E-
	Mycobacterium lentiflavum	WP_090608245	-R------I---G--AV---	LGGVTVCPTAPI	N-----I-------K-N-
	Mycobacterium mageritense	WP_081489044	--------I--DG--G----	VGGVRMCPAEPI	S-----IE------E---
	Mycobacterium malmesburyense	CRL78841	-RM-----I--GG--TM---	VGGVQMCPAEPI	S-----IE------E---
	Mycobacterium malmoense	WP_083185093	-R------I--GG--GV---	LGGVTVCPTAPL	D-----I-------K---
	Mycobacterium montefiorense	GBG40913	-R------I---G--V----	LGGVTACPTEPI	N-----I-------Q-N-
	Mycobacterium moriokaense	WP_083157242	--M-----I--DG--VM---	VGGVTMCPDQPI	D-----IN------K---
	Mycobacterium nebraskense	WP_046186043	-R------I--GG---V---	LGGVTVCPTTPL	A-----I-------K-N-
	Mycobacterium neoaurum	WP_082700300	IR---------GG--Q----	VGGVTMCPAEPI	T-----I-------E---
	Mycobacterium neworleansense	CRZ17520	--------I--DG--A----	VGGVRMCPAEPI	S-----I-------E---
	Mycobacterium novocastrense	GAT08746	-RI-----I--DG--TM---	VGGVTMCPAEPI	S-----I-------K---
	Mycobacterium palustre	ORW21132	-R------I--DG--A----	LGGVTMCPTMPI	S-------------K---
	Mycobacterium paraffinicum	WP_073876841	--------I--GG--GV---	LGGVTMCPAMPV	N-----I-------K---
	Mycobacterium parafortuitum	WP_083146940	-RI--------GG--GV---	VGGVTVCPDAPI	D-----IN------E---
	Mycobacterium parascrofulaceum	EFG79865	-R------I--GG--GV---	LGGVTVCPTAPL	D-----I-------K---
	Mycobacterium parmense	WP_085270797	-R------I--GG--G----	LGGVTMCPAEPI	D-----I-----------
	Mycobacterium peregrinum	WP_064884548	-R------I--DG--G----	VGGVTMCPAEPI	S-----I-------EF--
	Mycobacterium porcinum	WP_075924576	--------I--DG--AM---	VGGVTMCPAEPI	S-----I-V-----E---
	Mycobacterium rhodesiae JS60	EHB54895	-----------DG--VV---	LGGVQLCPADPI	D-----INI---------
	Mycobacterium rutilum	SEH57226	-RM-----I--DG--TM---	VGGVTMCPAEPI	S-----I-------E---
	Mycobacterium scrofulaceum	WP_082968455	-R------I--GG--GV---	LGGVTVCPTTPL	N-----I-------K-N-
	Mycobacterium septicum	WP_044513886	--------I--DG--A----	VGGVTMCPAEPI	S-----I-------E---
	Mycobacterium setense	KHO20500	--------I--DG--A----	VGGVTMCPAEPI	S-----I-------E---
	Mycobacterium shimoidei	WP_084226280	-R----V-I---G--AV---	LGGVTACPTASI	Q-----I-------SF--
	Mycobacterium shinjukuense	ORB67314	-R----------G--V----	LGGITVCPAEPI	R-----I-------R-G-
	Mycobacterium smegmatis	CKI54537	-R------I--GG--S----	VGGVTVCPAEPI	S-----I-------E---
	Mycobacterium sphagni	WP_094484614	-----------DG--V----	LGGVHLCPADPM	D-----IN----------
	Mycobacterium szulgai	WP_068034524	-R---------GG--V----	LGGVRVCPAEPI	S-----IN------H---
	Mycobacterium vanbaalenii PYR-	ABM16430	-RI-----I--GG--E----	VGGVTMCPEAPI	S-----I-------R---
	Mycobacterium vulneris	CDO29199	--------I--DG--A----	VGGVTMCPAEPI	T-----I-------E---
	Mycobacterium wolinskyi	WP_085144790	--------I--DG--A----	VGGVTMCPAEPI	S-----I-------V---
	Mycobacterium xenopi	ORX10849	-R----V-I---G--R----	LGGVTVCPTAAV	D-----I-------RA--
	Mycolicibacterium aromaticivorans	WP_109751209	-----------DG--V----	LGGVDVCPANPI	D-----IT----------
	Mycolicibacterium austroafricanum	WP_105386657	-RI-----I--GG--E----	VGGVTMCPEAPI	S-----I-------R---
	Mycolicibacterium boenickei	WP_077741759	--------I--DG--AM---	VGGVTMCPAEPI	S-----I-------E---
	Mycolicibacterium brisbanense	GAS90911	--------I--GG-------	VGGVTVCPAEPI	S-----I--A----Q---
	Mycolicibacterium canariasense	WP_109762698	-R---------DG--E----	IGGVTMCPAEPI	T-----I-------E---
	Mycolicibacterium chubuense NB	AFM19703	-RI-----I--GG--N----	VGGVRVCPTEPI	S-----I-------HI--
	Mycolicibacterium conceptionense	WP_065064251	--------I--DG--G----	VGGVRMCPAEPI	S-----IE------E---
	Mycolicibacterium confluentis	ORV24951	-R------I--GG---V---	LGGVTMCPAEPI	S-----I--A----K---
	Mycolicibacterium doricum	WP_085188709	-R---------DG--SV---	VGGVTMCPAEPM	S-----I-------E---
	Mycolicibacterium elephantis	WP_046754423	-R------I--DG--VM---	VGGVTMCLDEPV	D------N------K---
	Mycolicibacterium farcinogenes	WP_036392636	--------I--DG--G----	VGGVRMCPADPI	S-----IE------E---
	Mycolicibacterium flavescens	ODQ85862	-RV-----I--GG--TM---	VGGVTMCPAEPI	S-----I-------K---
	Mycolicibacterium fortuitum	ALI29600	--------I--DG--A----	VGGVRMCPAEPI	S-----I-------EF--
	Mycolicibacterium goodii	WP_049743667	--------I--GG--A---S	VGGVTVCPAEPI	N-----I-------E---

Figure S3. Partial sequence alignment of a conserved region of the transcriptional regulator protein showing a twelve amino acid deletion that is specific for members of the "Tuberculosis" clade. The alignment shown here is the same as the one in Figure 4, but it contains information for more outgroup species.

Group	Species	Accession	172		220
“Tuberculosis” clade (8/9)	*Mycobacterium tuberculosis*	AUS52500	LELDTLIPDSPNQPYDMHEVITRLLDD		EFLEIQAGYAQNIVVGFGRIDG
	Mycobacterium bovis	WP_081002200	---------------------------		----------------------
	Mycobacterium bovis BCG str. K	AGE69320	---------------------------		----------------------
	Mycobacterium canettii	WP_014001661	---------------------------	D	----------------------
	Mycobacterium microti	AMC61046	---------------------------		----------------------
	Mycobacterium mungi	WP_064319911	---------------------------		----------------------
	Mycobacterium orygis	EMT34506	---------------------------		----------------------
	Mycobacterium pinnipedii	PSY99039	---------------------------		----------------------
	Mycobacterium africanum	CCC28361	---------------------------		----------------------
Other *Mycobacteriaceae* (2/57)	*Mycobacterium setense*	WP_039378393	I----------------------I---		----V-----G--------V--
	Mycobacteroides abscessus	WP_074332858	-----------------------I---		----V--------I--------
	Mycobacterium alsense	WP_083139388	-----------------------I---	D	------G-------------E-
	Mycobacterium angelicum	WP_083111158	-----------------------I--E	D	------------V-------E-
	Mycobacterium asiaticum	WP_036353162	-----------------------I---	D	----------------------
	Mycobacterium avium	WP_009978782	-----------------------I---	D	------G---------------
	Mycobacterium avium subsp. paratuberculosis	ETB07843	-----------------------I---	D	------G---------------
	Mycobacterium branderi	WP_083129958	----------A------------I---	D	----V-------V-I-----E-
	Mycobacterium celatum	WP_062539186	----------A------------I---	D	----V-------V-I-------
	Mycobacterium florentinum	WP_085219997	-----------------------I---	D	----V-G------------V--
	Mycobacterium fragae	WP_085198622	-----------------------I--E	D	----V-----N---------E-
	Mycobacterium gastri	WP_036410109	--------------------V--I--E	D	-------------I------E-
	Mycobacterium genavense	WP_025737716	-----------------------I---	D	-------------------V--
	Mycobacterium gilvum	WP_041800347	-----------------------I---	D	----V-----G--------V--
	Mycobacterium gilvum PYR-GCK	ABP47242	-----------------------I---	D	----V-----G--------V--
	Mycobacterium gordonae	WP_065133672	----------------I------I---	D	-------------------V--
	Mycobacterium heidelbergense	WP_083073217	-----------------------I--E	D	----------------------
	Mycobacterium interjectum	WP_066908111	-----------------------I---	D	------G-------------E-
	Mycobacterium intermedium	WP_069420459	-----------------------V--E	D	--------------------E-
	Mycobacterium kansasii	OOK67361	--------------------V--I--E	D	-------------I------E-
	Mycobacterium komaniense	WP_090281645	-----------------------I--E	D	----V--------I------E-
	Mycobacterium kubicae	WP_085072784	-----------------------I--E	D	------G---------------
	Mycobacterium kyorinense	WP_057003322	----------A------------I---	D	----V-------V-I-----E-
	Mycobacterium lacus	WP_085160413	-----------------------I--E	D	--------------------E-
	Mycobacterium lentiflavum	WP_090600703	-----------------------I---	D	------G------------V--
	Mycobacterium lepraemurium	ATA29411	-----------------------I---	D	------G---------------
	Mycobacterium liflandii	WP_015354827	-----------------------I--E	D	--------------------E-
	Mycobacterium malmesburyense	WP_090342159	-----------------------I--E	D	----V---------------E-
	Mycobacterium malmoense	WP_071513250	-----------------------I--E	D	----------------------
	Mycobacterium marinum	WP_094358611	-----------------------I--E	D	--------------------E-
	Mycobacterium montefiorense	WP_108921247	-----------------------I---	D	----V-G---------------
	Mycobacterium obuense	WP_046362531	-----------------------I---	D	----V--------I--------
	Mycobacterium paraense	WP_085096103	-----------------------I---	D	--------------------E-
	Mycobacterium parmense	WP_085270655	I----------------------I--E	D	------N---------------
	Mycobacterium rhodesiae	WP_005140398	-----------------------I---	D	----V-----------------
	Mycobacterium riyadhense	WP_085251016	-----------------------I--E	D	------------V------VE-
	Mycobacterium rufum	KGI67295	-----------------------I---	D	-------------I-----V--
	Mycobacterium rutilum	WP_083409949	-----------------------I--E	D	----V--------I------E-
	Mycobacterium saskatchewanense	WP_085255948	-----------------------I---	D	------G---------------
	Mycobacterium sherrisii	WP_069399840	-----------------------I---	D	------G---------------
	Mycobacterium shimoidei	WP_069395236	----------A------------I--E	D	----V-----E---------E-
	Mycobacterium shinjukuense	WP_083047445	--------------------V--I---	D	-------------------V--
	Mycobacterium simiae	WP_061558485	-----------------------I---	D	------G---------------
	Mycobacterium sphagni	WP_094479281	-----------------------I--E	D	----V--------I--------
	Mycobacterium szulgai	WP_068028968	-----------------------I--E	D	------G---------------
	Mycobacterium thermoresistibile	WP_040548634	-----------------------I---	D	----V--------I-----V--
	Mycobacterium triplex	WP_036467188	-----------------------I---	D	-------------------V--
	Mycobacterium ulcerans	WP_011740575	-----------------------I--E	D	--------------------E-
	Mycobacterium ulcerans str. Harvey	EUA89375	-----------------------I--E	D	--------------------E-
	Mycobacterium xenopi	WP_003919131	-----------------------I--E	D	--------------------E-
	Mycobacterium xenopi 3993	EUA19391	-----------------------I--E	D	--------------------E-
	Mycobacterium xenopi 4042	EUA07160	-----------------------I--E	D	--------------------E-
	Mycolicibacterium aromaticivorans	WP_036340345	-----------------------I---	D	----V-----------------
	Mycolicibacterium aurum	WP_048630289	-----------------------I---	D	----V--------I--------
	Mycolicibacterium chlorophenolicum	WP_048471966	-----------------------I---	D	----V--------I-----V--
	Mycolicibacterium chubuense	WP_014814579	-----------------------I---	D	----------G--I--------
	Mycolicibacterium duvalii	WP_098003836	-----------------------I---	D	----------G--I--------

Figure S4. Partial sequence alignment of a conserved region of the propionyl-CoA carboxylase beta chain 5 protein showing a one amino acid deletion that is specific for most members of the “Tuberculosis” clade and is absent form most other *Mycobacteriaceae*.

Group	Species	Accession	41		95
"Tuberculosis" clade (10/10)	*Mycobacterium tuberculosis*	CFA97332	LAKGYRNPVSPDPFAEPGWFHTDDLGA	LE	SGDSGVLTVLGRADEAISTGGFTVLP
	Mycobacterium bovis	ALE42439	---------------------------	--	--------------------------
	Mycobacterium canettii	WP_080605235	---------------------------	--	--------------------------
	Mycobacterium canettii CIPT 14	CCK62624	---------------------------	--	--------------------------
	Mycobacterium caprae	PRH93712	---------------------------	--	--------------------------
	Mycobacterium microti	AMC58035	---------------------------	--	--------------------------
	Mycobacterium mungi	OAQ16175	---------------------------	--	--------------------------
	Mycobacterium orygis	EMT37084	---------------------------	--	--------------------------
	Mycobacterium pinnipedii	PRH91737	---------------------------	--	--------------------------
	Mycobacterium africanum Mal010	KBG65899	---------------------------	--	--------------------------
Other *Mycobacteriaceae* (1/32)	*Mycobacterium alsense*	WP_083136129	---------D----------R-E----	--	L-EA----------D------L----
	Mycobacterium avium	WP_020425424	---------D----------L------		VD-D-----F----D------L----
	Mycobacterium avium subsp. avium	EUA39555	---------D-----D----L------		VD-D-----F----D------L----
	Mycobacterium avium subsp. hominissuis	PBJ31865	---------D-----D----L------		VD-D-----F----D------L----
	Mycobacterium bohemicum	ORV02881	---------D----------R------		ID-A----------D------L----
	Mycobacterium bohemicum DSM 44	CPR11783	---------D----------R------		ID-A----------D------L----
	Mycobacterium branderi	WP_083130287	---------D----------R-E-I-V		LD-------------------L----
	Mycobacterium colombiense	OBB81775	---------D----------A------		VS-A----------D------L----
	Mycobacterium colombiense CECT	EJO90684	---------D----------V------		VS-A----------D------L----
	Mycobacterium conspicuum	ORV35326	---------I----------N------		LD------------D------L----
	Mycobacterium florentinum	WP_085222834	---------D----------R-----S		I------------D------L----
	Mycobacterium heidelbergense	ORA72990	---------D----------R------		VD------------D------L----
	Mycobacterium intermedium	WP_079220231	---------H----------Y------		ID-A---S----V--------L----
	Mycobacterium kyorinense	ORV98107	---------E----------R-E-I-V		LD-------------------L----
	Mycobacterium lacus	ORW04190	---------H------S-------V--		VN-----R----T--------L----
	Mycobacterium marinum	WP_103671004	---------Q----------R------		LD-----S------D------L----
	Mycobacterium marinum M	ACC39345	---------Q----------R------		LD-----S------D------L----
	Mycobacterium marinum str. Europe	EPQ70072	---------Q----------R------		LD-----S------D------L----
	Mycobacterium nebraskense	KKB96964	---------D----------R------		VDEA----------D------L----
	Mycobacterium paraense	ORW29234	-------R-D----------Y------		VSE-----------D------L----
	Mycobacterium parascrofulaceum	EFG76430	---------D----------R------		VDEA----------D------L----
	Mycobacterium pseudoshottsii J	GAQ32705	---------Q----------R------		LD-----S------D------L----
	Mycobacterium shigaense	BAX90930	---------D----------------S		ID-A----------D---S--L----
	Mycobacterium shinjukuense	WP_083047938	------S--D-----------------		VD----------V--------L----
	Mycobacterium ulcerans	WP_083421690	---------Q----------R------		LD-----S------D------L----
	Mycobacterium ulcerans Agy99	ABL03306	---------Q----------R------		LD-----S------D------L----
	Mycobacterium ulcerans str. Harvey	EUA92415	---------Q----------R------		LD-----S------D------L----
	Mycobacterium vulneris	WP_085288042	---------D----------A------		VS-A----------D------L----
	Mycobacterium xenopi	ORX20358	--R------D----------Y------		LD---R-S-------------L--V-
	Mycobacterium xenopi 3993	EUA22430	--R------D----------Y------		LD---R-S-------------L--V-
	Mycobacterium xenopi 4042	EUA52406	--R------D----------Y------		LD---R-S-------------L--V-
	Mycobacterium xenopi RIVM70036	EID16345	--R------D----------Y------		LD---R-S-------------L--V-

Figure S5. Partial sequence alignment of a conserved region of the O-succinylbenzoic acid-CoA ligase MenE protein showing a two amino acid insertion that is specific for members of the "Tuberculosis" clade and absent in most other *Mycobacteriaceae*.

Group	Species	Accession	180		234
“Tuberculosis” clade (9/9)	*Mycobacterium tuberculosis*	CKP03712	VVWVAAGGAWANDSVSCPRSGEVIVR	KAPS	QEDHWYSTGADFKRPAPHWWFDDAT
	Mycobacterium bovis	WP_044798715	----------S---------S-----	----	-------------------------
	Mycobacterium canettii	WP_014001908	----------S---------------	N---	-------------------------
	Mycobacterium caprae	APU27511	----------S---------------	----	-------------------------
	Mycobacterium microti	AMC61521	----------S---------------	----	-------------------------
	Mycobacterium mungi	OAQ18994	----------S---------------	----	-------------------------
	Mycobacterium orygis	EMT33996	----------S---------------	----	-------------------------
	Mycobacterium pinnipedii	PRH91614	----------S---------------	----	-------------------------
	Mycobacterium africanum	KBG14050	----------S---------------	----	-------------------------
Other *Mycobacteriaceae* (1/45)	*Mycobacterium europaeum*	CQD05411	--------S-----------------	DPSI	KGG--F-S-------S-Q-----EK
	Mycobacterium alsense	WP_083140810	--------------------------		DRG------------S-Q-----H-
	Mycobacterium angelicum	WP_083115754	--------S-S---------------		EQG------------S-------D-
	Mycobacterium arosiense	WP_083063912	--------S-S---------------		DRG------V-----S-Q-----D-
	Mycobacterium asiaticum	WP_065153828	--------S-----------------		EQQ------------S-Q-----T-
	Mycobacterium avium complex	WP_038534838	--------S-S---------------		ERG--F---------S-Q-----Q-
	Mycobacterium bohemicum	WP_085183286	--------S-----------------		EQG------------S-Q-----E-
	Mycobacterium bohemicum DSM 44	CPR10993	--------S-----------------		EQG------------S-Q-----E-
	Mycobacterium chimaera	WP_094080860	--------S-S---------------		ERG--F---------S-Q-----H-
	Mycobacterium colombiense	WP_007772129	----------S----------I---		EPG------------S-------DA
	Mycobacterium conspicuum	WP_085232645	--------S-----------------		EQG------------S-------D-
	Mycobacterium florentinum	WP_085224958	--------------------------		DQG------------S-Q-----D-
	Mycobacterium genavense	WP_025736394	--------S-----------------		EKG------------S-R-----E-
	Mycobacterium gordonae	WP_055580281	--------S-S---------------		EQG------------S-Q----EQ-
	Mycobacterium haemophilum	WP_047316203	--------T-S---------------		DNG------------S------NN-
	Mycobacterium heidelbergense	WP_083074886	--------------------------		DKG------------S-Q-----D-
	Mycobacterium interjectum	WP_085202284	--------------------------		DKE------------S-Q-----E-
	Mycobacterium intracellulare	WP_064935016	--------S-S---------------		ESG--F---------S-Q-----H-
	Mycobacterium kansasii	WP_023368714	--------S-S---------------		EQG------------S-------D-
	Mycobacterium kansasii 824	EUA05190	--------S-S---------------		EQG------------S-------D-
	Mycobacterium lacus	WP_085158918	----------SG--------------		DQG------------S---L--SD-
	Mycobacterium leprae	WP_010908833	--------T-S---------------		KNR------------S-------N-
	Mycobacterium liflandii	WP_041299754	--------S-S---------------		EPG------------R-Q-----EA
	Mycobacterium liflandii 128FXT	AGC64927	--------S-S---------------		EPG------------R-Q-----EA
	Mycobacterium malmoense	WP_065445100	--------S-----------------		DKG------------S-Q-----E-
	Mycobacterium mantenii	WP_083095562	--------S-S---I-----------		DKG------------S-Q-----D-
	Mycobacterium marinum	WP_036450657	--------S-S---------------		EPG------------S-Q-----EA
	Mycobacterium marinum M	ACC43634	--------S-S---------------		EPG------------S-Q-----EA
	Mycobacterium marinum MB2	EPQ78914	--------S-S---------------		EPG------------S-Q-----EA
	Mycobacterium marinum str. Europe	EPQ72001	--------S-S---------------		EPG------------S-Q-----EA
	Mycobacterium nebraskense	KKB97163	--------S-----------------		DKN------------S-Q-----E-
	Mycobacterium paraense	WP_085171272	--------------------------		DQG------------S-Q-----E-
	Mycobacterium paraffinicum	WP_073871693	--------S-----------------		DKG------------S-Q-----E-
	Mycobacterium paraintracellulare	WP_014383685	--------S-S---------------		ERG--F---------S-Q-----H-
	Mycobacterium paraseoulense	WP_083176607	--------S-----------------		EKG------------S-Q-----E-
	Mycobacterium parmense	WP_085271229	--------S-----------------		ERG------------S-Q-----D-
	Mycobacterium riyadhense	WP_085252809	----------SS--------------		DQG------------S------AD-
	Mycobacterium saskatchewanense	WP_085258266	--------S-----------------		DTH------------S-Q-----E-
	Mycobacterium scrofulaceum	WP_067270952	-L------S-----------------		EKG------------S-Q-----H-
	Mycobacterium shinjukuense	WP_083048882	----------S---------------		DRG------------S------EN-
	Mycobacterium szulgai	WP_085670713	--------S-S---------------		EQG------------S------A--
	Mycobacterium talmoniae	WP_071029146	--------G--H--------------		ERA------------S-------D-
	Mycobacterium triplex	CDO85758	--------S-----------------		EKG------------S-Q-----D-
	Mycobacterium vulneris	WP_085290696	--------S-S----------I---		DRG------------S-Q-----DA

Figure S6. Partial sequence alignment of a conserved region of a ligase protein showing a four amino acid insertion that is specific for members of the “Tuberculosis” clade and absent from most other *Mycobacteriaceae*.

	Species	Accession	747		795
"Tuberculosis" clade (14/14)	*Mycobacterium tuberculosis*	AHZ31631	CGLADDVLVEPDTNAGFMKPLDGD	SGS	WGPLGPLGGVNPVGFTPNGVPE
	Mycobacterium africanum K85	EFD41456	------------------------	---	----------------------
	Mycobacterium africanum MAL020	KBI26945	------------------------	---	----------------------
	Mycobacterium bovis	WP_079293517	------------------------	---	----------------------
	Mycobacterium bovis AF2122/97	YP_009361185	------------------------	---	----------------------
	Mycobacterium bovis BCG str. A	AHM09574	------------------------	---	----------------------
	Mycobacterium canettii	WP_080624417	------------------------	---	----------------------
	Mycobacterium canettii CIPT 14	CCK66029	------------------------	---	----------------------
	Mycobacterium caprae	CEJ50030	------------------------	---	----------------------
	Mycobacterium microti	AMC61613	------------------------	---	----------------------
	Mycobacterium mungi	OAQ19140	------------------------	---	----------------------
	Mycobacterium orygis	EMT33993	------------------------	---	----------------------
	Mycobacterium pinnipedii	WP_107131691	------------------------	---	----------------------
	Mycobacterium africanum	KBF93339	------------------------	---	----------------------
Other ***Mycobacteriaceae*** (5/>100)	*Mycobacterium bohemicum*	ORU95584	-A----------S-----T--P--	--P	------------T--S------
	Mycobacterium heidelbergense	WP_083075946	------------S-----A--PDN	--P	------------T--------D
	Mycobacterium saskatchewanense	WP_085258360	-A----------S-----T--PPE	--G	Y--------------------D
	Mycobacterium shigaense	WP_096443647	------------S-----P--L-N	P-P	-S----------T--S---I-D
	Mycolicibacillus koreensis	OSC33534	-----Y------A-D--LH--PDG	A-A	-----A-A--D----SAD----
	Mycobacterium alsense	WP_083138471	------------S-V---A--P--		Y----A------T---------
	Mycobacterium angelicum	WP_083114039	------------P-----T-VP--		Y----------------D----
	Mycobacterium aquaticum	WP_083168783	--------------N--LT--P--		Y--------TA-T--------D
	Mycobacterium arosiense	WP_083063195	------I-----S-----AA-S--		Y-----------T--------D
	Mycobacterium asiaticum	WP_065033877	------------------T--P--		Y--------TD----S------
	Mycobacterium avium	WP_062888024	------------S-V-Y-T--G--		Y--------QH----S------
	Mycobacterium branderi	WP_083133537	------------------PA-P--		Y-S--S---------S-D----
	Mycobacterium celatum	WP_062539468	------------------AA-P--		Y-S------PS------D----
	Mycobacterium chimaera	WP_008263053	------------S-V-Y-A--P-N		Y--------EH----S------
	Mycobacterium colombiense	WP_064950914	------------S-----V--PDN		Y-----------T--------D
	Mycobacterium conceptionense	CQD02212	--------------D--LA--P--		Y--------TS-T--------D
	Mycobacterium dioxanotrophicus	WP_087083166	------------P-V--LT-EA-A		Y--------DA----S-D-L--
	Mycobacterium europaeum	WP_085242490	------I-----S-----AA-P-N		------------T--------D
	Mycobacterium florentinum	WP_085224771	------------S-V---T-YP-S		YTA--A---T--T--S---I--
	Mycobacterium fragae	WP_085196768	------------------TSSP--		Y--------------A-----D
	Mycobacterium gastri	WP_036411168	------------P-E---TA-P--		Y--------I--T---------
	Mycobacterium genavense	WP_036468502	------------S-----T-YP-S		YS---A---I--T--SA-----
	Mycobacterium gordonae	WP_065048494	------------------T--P-N		Y--------SD----S------
	Mycobacterium grossiae	WP_099045888	------------S----LRA-P-N		Y--------TS-A--S-----D
	Mycobacterium haemophilum	WP_047314801	------------S---Y-T--P-N		Y---------SAI---------
	Mycobacterium hassiacum	WP_005630998	------------P-E--LT--P--		Y---------A-----A----D
	Mycobacterium heckeshornense	WP_099869408	------------A-----AA-P--		Y-A--------------D----
	Mycobacterium houstonense	WP_066902118	--------------D--LT-AP--		Y--------TA-T--------D
	Mycobacterium interjectum	WP_066916973	-A----------S-----AA-P-N		Y--------AD----------D
	Mycobacterium intermedium	WP_069420083	------------P-E---T--P--		Y--------TD----S------
	Mycobacterium intracellulare	WP_064935347	------------S-V-Y-A--P-N		Y--------EH----S------
	Mycobacterium iranicum	WP_036463580	------------S-D--LT-AP-R		Y--------TD-T--S-----D
	Mycobacterium kansasii	WP_063468152	------------P-N---TA-P--		Y--------T--T---------
	Mycobacterium kubicae	WP_085074903	------------P-----T--P--		Y--------T-----S-D----
	Mycobacterium kyorinense	WP_065012532	--------------D---V--P--		Y-S-------D------S----
	Mycobacterium lacus	WP_085162437	------------------T-QP--		Y----------------D---D
	Mycobacterium leprae	WP_010907526	------------S---Y-TA-PSN		Y----------AI---A-----
	Mycobacterium lepraemurium	ATA27262	----N-------S-V-Y-T--G--		Y--------QH----S------
	Mycobacterium lepromatosis	KJX75859	------------S---Y-TA-PSN		Y----------AI---------
	Mycobacterium liflandii	WP_015357628	------------------T--P--		Y--------------S-----D
	Mycobacterium litorale	WP_078021379	------------A----LT--P--		Y--------T----Y-------
	Mycobacterium llatzerense	WP_083420577	------------A----LT-EA-R		Y--------AD-I--D------
	Mycobacterium mageritense	WP_081812450	-----------------LT--PAQ		-G-A-K-----S------
	Mycobacterium malmoense	WP_065442742	------I-----S-----PA-PDS		F---------K-T--------D
	Mycobacterium mantenii	WP_083097257	------------S-V---A--PDN		F-----------T--------D
	Mycobacterium marinum	WP_094361570	------------------T--P--		Y--------------S-----D
	Mycobacterium montefiorense	WP_108926393	------------S-----T-FP--		Y----A------T---------
	Mycobacterium mucogenicum	WP_064861216	-----N------S----LT-QA--		Y----A----G----------N
	Mycobacterium nebraskense	WP_047322860	------------S-----A--P-N		Y-S---------T--------D
	Mycobacterium neoaurum	CDQ43577	--M--------NS-D--LRA-P--		Y--------TD-A--S-D---D
	Mycobacterium neworleansense	WP_090517035	--------------D--LT-VP-E		Y--------TA-T--------D
	Mycobacterium noviomagense	WP_083088458	----A-------------PA-P-N		Y-A--------AI--------D
	Mycobacterium novocastrense	WP_067388628	------------A-Q--LTA-P-Q		Y--------DR-----AD----
	Mycobacterium obuense	WP_046366460	------------S----LS--P--		Y--------AT-T--S------
	Mycobacterium palustre	WP_085079060	-A----------S-----A--PDR		Y-----------M--S---I--
	Mycobacterium paraense	WP_085095310	-A----------S-----A--PDN		F-----------M--S---I--
	Mycobacterium paraffinicum	WP_073874976	------------S-----A--P--		Y----A---T--T--S-----D
	Mycobacterium paraseoulense	WP_083171803	------I-----S-----AA-P--		Y-----------T--------D
	Mycobacterium parmense	WP_085270832	------------S-----P-FP-N		Y----A------T----D----

Other *Mycobacteriaceae* (5/>100)

Mycobacterium peregrinum	OBB24714	------------A----LA--P-P	E----A---TE-I--D---L--
Mycobacterium persicum	WP_083153541	------------P-E---TA-P--	Y--------I--T---------
Mycobacterium phlei	WP_061481003	-A----------P-E---T--P--	Y---------D-I--A-D----
Mycobacterium porcinum	WP_069427548	------------P-T--LT-AA-R	Y-S------SE-I---AD----
Mycobacterium pseudoshottsii	WP_086084843	-----------------T--P--	Y-----------A--S-----D
Mycobacterium rhodesiae	WP_083121838	------------A----LT--P--	Y--------T-------S----
Mycobacterium riyadhense	WP_085252439	------------P-S---T--P--	Y---------------------
Mycobacterium rufum	KGI71208	------------S----LQ-MP-R	Y--------AT-T--S------
Mycobacterium rutilum	WP_083406724	-A----------A-D---PVVP--	F---------D-A---AD----
Mycobacterium scrofulaceum	WP_067274222	------------S-----AA-P--	Y-----------T--------D
Mycobacterium septicum	WP_044513836	--------------D--LT-AP--	Y--------TA-T--------D
Mycobacterium setense	WP_064876605	--------------D--LT-AP--	Y--------TA-T--------D
Mycobacterium shimoidei	WP_069394283	--------------E---AA-P--	Y----------------D----
Mycobacterium shinjukuense	ORB70603	------------P-----TA-P--	Y--------SD-----------
Mycobacterium smegmatis	AAC45273	------------S----LT--P-A	Y--------ED-Q--S-D---D
Mycobacterium sphagni	WP_094482448	------------A----LT--P--	Y--------T-----A-S----
Mycobacterium szulgai	WP_068034133	------------P-----T--P--	Y--------T-----S-D----
Mycobacterium talmoniae	WP_071026500	------------A-D---P--P-E	Y-A------TGA---S-D----
Mycobacterium triplex	CDO85942	------------S-V---T-YP-S	YS---A---I--T---A-----
Mycobacterium ulcerans	WP_011742448	------L-----------T--P--	Y--------E-----S-----D
Mycobacterium vanbaalenii	WP_011782749	------------S----LQ-IP-R	Y--------TD-A--S-----D
Mycobacterium vulneris	OCB08205	------------P-T--LT-AA-R	Y--------SE-I---AD----
Mycobacterium wolinskyi	WP_085144786	------------S----LQAMP-N	Y--------E--A--S-----D
Mycobacterium xenopi	WP_085195664	------------A-----AA-P-N	Y-A--------------D----
Mycobacteroides abscessus	WP_074254211	------------A---ALQ-MALA	T-----------T--SAD---D
Mycolicibacillus trivialis	WP_085109233	------------A-D--LT-----	Y------A-AD----SAD----
Mycolicibacter engbaekii	WP_085126478	-----N------P-D-ALT--P-Q	Y-E-------D-----AS----
Mycolicibacter heraklionensis	WP_047319107	-----N------P-D-ALT--P-E	Y-E-------D-----AS----
Mycolicibacter hiberniae	WP_085134390	-----N------P-D-ALT--P-Q	Y-E-------D-----AS----
Mycolicibacter icosiumassiliensis	WP_067977023	------------P-D-ALT--P-E	Y-D-------D-----AS----
Mycolicibacter kumamotonensis	WP_065287143	--Q--N------A-D-ALT--P-E	Y-E------AD----SA-----
Mycolicibacter minnesotensis	WP_083027061	-----N------P-D-ALT--P-A	Y-D-------D-----AS----
Mycolicibacter nonchromogenicus	WP_085137219	-----N------P-D-ALT--P-E	Y-E-------------A-----
Mycolicibacter senuensis	WP_085085361	--Q--N------P-D-ALT--P--	Y-E-------D-----A-----
Mycolicibacter sinensis	WP_064854835	------------A-D-ALT--P-R	Y-E------SD-----AS----
Mycolicibacter terrae	WP_085260015	--Q--N------A-D-ALT--P-E	Y-E-------D-I--SA-----
Mycolicibacterium agri	WP_097940589	------------P-D--LNA-P--	Y--------E-----------N
Mycolicibacterium aromaticivorans	WP_036341988	------------A-T--LT--P--	Y--------TS------S----
Mycolicibacterium aurum	WP_048630679	------------S-D--LQA-P--	Y-R------TD---S-----D
Mycolicibacterium austroafricanum	WP_105389140	------------S----LQ-IP-R	Y--------TD-A--S-----D
Mycolicibacterium bacteremicum	WP_109750215	--M---------S-D--LRA-P--	Y--------TD-S----D----
Mycolicibacterium boenickei	WP_077740669	--------------D--LT-AP--	Y--------TA-T--------D
Mycolicibacterium brisbanense	WP_062827211	------------P-V--LT-EP-S	Y-Q------DA----S-D-L--
Mycolicibacterium canariasense	GAS99419	------------P-Q--LTA-P--	Y---------K----------D
Mycolicibacterium chlorophenolicum	WP_048472898	------------S----LQ-MP--	Y--------AT-T--S------
Mycolicibacterium chubuense	ORA44304	------------S----LQ-MP--	Y--------AT-T--S------
Mycolicibacterium conceptionen	WP_085141702	--------------D--LA--P--	Y--------TS-T--------D
Mycolicibacterium confluentis	WP_085154488	--M---------P-D--LT--GTG	F----A---EE----S------
Mycolicibacterium cosmeticum	WP_036399014	------------P-Q--LTA-P--	Y---------K----------D
Mycolicibacterium doricum	WP_085192104	------------S-D--LRA-P-E	Y-Q------TGT---S-D---D
Mycolicibacterium elephantis	WP_083043239	------------P-E--LTA-P--	Y---------D------D----
Mycolicibacterium farcinogenes	WP_036391028	--------------D--LT--P--	Y--------TS-T--------D
Mycolicibacterium flavescens	WP_069416402	-M----------A-D---T--P--	Y---------D-----------
Mycolicibacterium fortuitum	WP_064897738	--------------D--LT-VP-E	Y--------TA-T--------D
Mycolicibacterium goodii	WP_049743636	------------S----LT--P-A	Y--------EH-Q--S-----D

Figure S7. Partial sequence alignment of a conserved region of the arabinosyltransferase protein showing a three amino acid insertion that is specific for members of the “Tuberculosis” clade and absent from most other *Mycobacteriaceae*.

Group	Species	Accession	225	Insertion	283
**"Tuberculosis" clade (9/9)**	*Mycobacterium tuberculosis*	AMC68818	DVIGLCIPADEAIGPGDRWIVEQLRSTGP	A	NTTLVVIVTKIDKVPKEKVVAQLVAVSEL
	Mycobacterium bovis	WP_099179445	-----------------------------	-	-----------------------------
	Mycobacterium canettii	WP_015290613	--------------------------P--	-	-----------------------------
	Mycobacterium caprae	APU27937	-----------------------------	-	-----------------------------
	Mycobacterium microti	AMC60048	-----------E-----------------	-	-----------------------------
	Mycobacterium mungi	OAQ17029	-----------------------------	-	-----------------------------
	Mycobacterium orygis	EMT35309	-----------------------------	-	-----------------------------
	Mycobacterium pinnipedii	PRH92260	-----------E-----------------	-	-----------------------------
	Mycobacterium africanum	AMC64655	-----------------------------	-	-----------------------------
**Other *Mycobacteriaceae* (0/54)**	*Mycobacterium alsense*	WP_083140944	---------------------D-I-AVA-		K---LG----------DR-A---------
	Mycobacterium arosiense	WP_083062979	-----------------------I-A-A-		K----A-------T--DR-A---------
	Mycobacterium asiaticum	WP_036358699	--------------------AD-I--VA-		K----A----------DR-A---------
	Mycobacterium avium	WP_003872521	-----------------------I-A-A-		K----A----------DR-A---------
	Mycobacterium bohemicum	WP_085182922	-----------------------I---A-		K----A----------DR-A---------
	Mycobacterium colombiense	WP_007770275	-----------------------I-A-A-		K----A-------T-RDR-A---------
	Mycobacterium europaeum	WP_085240324	---------------------D-I---A-		K-K--A---------RDR-A---------
	Mycobacterium florentinum	WP_085220630	--------------------LD-I--VA-		R---------------DR-A---------
	Mycobacterium gastri	WP_036417304	--------------------L--I--VA-		G-A-------------DR-AV--S--G--
	Mycobacterium genavense	WP_025735550	--------------------LD-I-AVA-		K--------------RDR-AS-----N--
	Mycobacterium gordonae	WP_055581787	-----------P-----------V-ALA-		K----A----------DR-A---------
	Mycobacterium haemophilum	WP_047315094	---------------------D-I--VA-		K---------L-----DRLP-------D-
	Mycobacterium heidelbergense	WP_083072097	-----------------------V-AIA-		R-K---V---------DR-A---M-----
	Mycobacterium interjectum	WP_066914039	---------------------D-I-AVA-		R-A--AV---------DR-A---------
	Mycobacterium intermedium	WP_069420569	-I---------------------ISAVA-		K-KV------------DR-AG--------
	Mycobacterium intracellulare	WP_009953083	-----------------------I-A-A-		KA---A-------A--DR-A-------A-
	Mycobacterium intracellulare A	AFC43243	-----------------------I-A-A-		KA---A-------A-RDR-A-------A-
	Mycobacterium intracellulare s	AFS13947	-----------------------I-A-A-		KA---A-------A--DR-A-------A-
	Mycobacterium kansasii	WP_023365635	--------------------L--I--VA-		G---------------DR-A------G--
	Mycobacterium lacus	WP_085161066	--------------------INEV--IA-		K----IV--------R-RLA-----A---
	Mycobacterium lentiflavum	WP_090603912	--------------------LD-I--VA-		K---------------DR-A---L-----
	Mycobacterium leprae	WP_010907847	-L----------T--------N-I--VA-		K-I-------------DRLS-------D-
	Mycobacterium leprae 3125609	Q49768	-L----------T--------N-I--VA-		K-I-------------DRLS-------D-
	Mycobacterium lepraemurium	ATA28905	-----------------------I-A-A-		K----A----------DR-----------
	Mycobacterium lepromatosis	KJX75569	-----------SP--------N-I--VAS		K---------------DRLP--M----D-
	Mycobacterium liflandii	WP_015356470	-----------S--------I--IAA-A-		KVK-------------DQ-A---------
	Mycobacterium malmoense	WP_065473899	---------------------D-I---A-		K----A---------RDR-A---------
	Mycobacterium mantenii	WP_083095749	-----------T-----------I-A-A-		K-A--A-------T--DR-A-----A---
	Mycobacterium marinum	WP_020730334	-----------S--------I--IAA-A-		RVK-------------DQ-A---------
	Mycobacterium marseillense	WP_083020480	-----------------------I-A-A-		KA---A-------M--DR-A---------
	Mycobacterium montefiorense	WP_108925349	-----------------------I--VA-		K-A-------------DR-A---------
	Mycobacterium nebraskense	WP_046186167	---------------------D-I---A-		K-A--A---------RDR-A---------
	Mycobacterium palustre	WP_085079994	-----------------------V-AVA-		-----AV--------RDR-A---------
	Mycobacterium paraense	WP_085104172	-----------P---------D-I-AVA-		K-A--AV---------DR-A---------
	Mycobacterium paraffinicum	WP_073873813	---------------------D-I---A-		K----A----------DR-A---------
	Mycobacterium parascrofulaceum	EFG77888	---------------------D-I---A-		K----A---------RDW-A---------
	Mycobacterium paraseoulense	WP_083169180	---------------------D-I---A-		K----A---------RDR-A-------D-
	Mycobacterium parmense	WP_085267524	-----------S-----------I---A-		K----AV---------DR-A---------
	Mycobacterium pseudoshottsii J	GAQ32367	-----------S--------I--IAA-A-		KVK-------------DQ-A---------
	Mycobacterium riyadhense	WP_085252570	---------------------D-I--IA-		K-A-I---------G-DRLA---A-----
	Mycobacterium saskatchewanense	WP_085254797	-----------------------I---A-		K----A---------RDR-A---------
	Mycobacterium scrofulaceum	WP_067276355	-I-------------------D-I---A-		R----A-----------R-A---------
	Mycobacterium sherrisii	WP_069398900	---------------------D-I--VA-		K----A----------DR-A---------
	Mycobacterium shigaense	BAX93495	-----------------------I--VA-		K----A----------DR-A------A--
	Mycobacterium shinjukuense	WP_083046336	---------------------D-I--SA-		--A-------------DRLA--------A
	Mycobacterium simiae	WP_061556276	-------------------V-D-I--VA-		-----A----------DR-A---L-----
	Mycobacterium szulgai	WP_068156932	-----------------------I-AIA-		K----A---------RD--A---------
	Mycobacterium triplex	WP_036470251	--------------------LD-I--VA-		K------------A-RDR-AG--------
	Mycobacterium ulcerans	WP_011741358	-----------S--------I--IAA-A-		KVK-------------DQ-A---------
	Mycobacterium ulcerans str. Harvey	EUA87706	-----------S--------I--IAA-A-		KVK-------------DQ-A---------
	Mycobacterium vulneris	WP_085291629	-----------------------I-A-A-		R----A-------T--DR-A---M-----
	Mycobacterium xenopi	WP_003921918	---------------------D-I--IA-		K-----V-------S-DQLA---A--G--
	Mycolicibacterium doricum	ORV42540	--------------------H--I-AVA-		R----A---------RDR-A---------
	Mycolicibacterium elephantis	WP_083043342	--------V--K--------C--I-AVA-		-----A----------DR-A---------

Figure S8. Partial sequence alignment of a conserved region of the GTPase Era protein showing a one amino acid insertion that is specific for members of the "Tuberculosis" clade.

Group	Species	Accession	609	Insertion	655
"Tuberculosis" clade (9/9)	*Mycobacterium tuberculosis*	AIH87751	RMLLRVCREQGLELAASLRRGIGVLSARQTRQT	RSL	VRVQIDPLHIG
	Mycobacterium bovis	AMC54764	---------------------------------	---	-----------
	Mycobacterium canettii	WP_015288055	---------------------------------	---	-----------
	Mycobacterium caprae	APU27833	---------------------------------	---	-----------
	Mycobacterium microti	AMC58995	---------------------------------	---	-----------
	Mycobacterium mungi	WP_064319848	---------------------------------	---	-----------
	Mycobacterium orygis	EMT36187	---------------------------------	---	-----------
	Mycobacterium pinnipedii	PRH90273	---------------------------------	---	-----------
	Mycobacterium africanum	WP_031670458	---------------------------------	---	-----------
Other *Mycobacteriaceae* (0/36)	*Mycobacterium angelicum*	WP_083114027	---V--G-RH-----------V--------H-S		L-------Q--
	Mycobacterium avium subsp. avium	EUA39328	---V--P-RD--A---A----V--------HEP		A----------
	Mycobacterium avium subsp. hominissuis	KDP07584	---V--P-RD--A---A----V--------HEP		A----------
	Mycobacterium chimaera	ASL10267	---V--P-R---A---A----V--------HEP		A----------
	Mycobacterium colombiense	WP_076053333	---V--P-Q---A---A----V-------AHEP		A----------
	Mycobacterium conspicuum	WP_085232593	---V--R--H--A---A----V---------DP		A--------V-
	Mycobacterium haemophilum	WP_047316300	---V--P-----Q-------------V---H-P		-----------
	Mycobacterium heidelbergense	WP_083074369	---V--R-----A--------V-------AHEP		-----------
	Mycobacterium interjectum	WP_085202017	---V--P-----A--G-----V-------DHEP		---------V-
	Mycobacterium intracellulare	WP_036390158	---V--P-R---A---A----V--------HEP		A----------
	Mycobacterium intracellulare 1	EUA55210	---V--P-R---A---A----V--------HEP		A----------
	Mycobacterium intracellulare A	AFC44433	---V--P-R---A---A----V--------HEP		A----------
	Mycobacterium intracellulare M	AFC49588	---V--P-R---A---A----V--------HEP		A----------
	Mycobacterium intracellulare s	ARR78847	---V--P-R---A---A----V--------HEP		A----------
	Mycobacterium kansasii	ORB87881	---I--R--H----------AVS-------HEP		-----------
	Mycobacterium kansasii 732	EUA11905	---V--R--H------C---AVS---T---HEP		-----------
	Mycobacterium kansasii 824	ETZ99787	---V--R--H------C---AVS-------HEP		-----------
	Mycobacterium lacus	WP_085158836	---V--R-----Q----------------AH-P		----V------
	Mycobacterium lentiflavum	CQD12657	---V--P--------------V-------SHEP		-------PQ--
	Mycobacterium leprae	WP_010907788	-L-V--P----WQ-------------V---H-L		-----------
	Mycobacterium lepromatosis	WP_045842568	-L-V--P----WQ-------------V---H-L		-----------
	Mycobacterium liflandii	WP_015355812	---V--R--------GC----V--------HEP		-----------
	Mycobacterium mantenii	WP_083094939	---V--P-Q---A---A----V--------HEP		A----------
	Mycobacterium marinum	WP_012393975	---V--R--------GC----V--------HEP		-----------
	Mycobacterium marseillense	WP_083019558	---V--P-R---A---A----V-I------HEP		A----------
	Mycobacterium montefiorense	GBG38383	---V--R--R----S------V--------HEP		-----------
	Mycobacterium paraense	WP_085244929	---A--P-----A---A----V-------NHEP		A----------
	Mycobacterium paraintracellulare	AFC54848	---V--P-R---A---A----V--------HEP		A----------
	Mycobacterium paraseoulense	WP_083174014	---V-AH-----A---A----VS------SKEP		-----------
	Mycobacterium riyadhense	WP_085250307	---V--R--------------V--------HDP		--------Q--
	Mycobacterium sherrisii	WP_069402179	---V--P--------T-----V--V-----NEP		--------Q--
	Mycobacterium shinjukuense	WP_083046476	---V--RHQ---R--------VS-------QEP		-----------
	Mycobacterium triplex	CDO88266	---V--R--------------V--------HEP		A------PQ--
	Mycobacterium ulcerans	WP_096371059	---V--R--------GC----V--------HEP		-----------
	Mycobacterium ulcerans str. Harvey	EUA90691	---V--R--------GC----V---G----HEP		-----------
	Mycobacterium vulneris	WP_085289529	---V--P-Q---A---A----V--------HEP		A----------

Figure S9. Partial sequence alignment of a conserved region of the primosome assembly protein PriA showing a three amino acid insertion that is specific for members of the "Tuberculosis" clade.

Group	Species	Accession	43		102
“Tuberculosis” clade (9/9)	*Mycobacterium tuberculosis*	CFR41125	NPMPDNGIKIFGPGGHKLDDDTEDQIEDL	VLG	VSRGPGLRPAGAGIGRVIDAEDATERYL
	Mycobacterium bovis	ESK73327	-----------------------------	---	----------------------------
	Mycobacterium canettii	WP_015303892	-----------------------------	---	----------------------------
	Mycobacterium caprae	APU27283	-----------------------------	---	----------------------------
	Mycobacterium microti	AMC61230	-----------------------------	---	----------------------------
	Mycobacterium mungi	OAQ18663	-----------------------------	---	----------------------------
	Mycobacterium orygis	EMT34273	-----------------------------	---	----------------------------
	Mycobacterium pinnipedii	PRH93428	-----------------------------	---	----------------------------
	Mycobacterium africanum	CCC28525	-----------------------------	---	----------------------------
Other *Mycobacteriaceae* (1/71)	*Mycobacterium colombiense*	WP_076052044	--------------------G------A-	LAD	-PVD-----V-------------DD---
	Mycobacterium alsense	WP_083137310	---------------------------A-		-AA------V------R-E----AD---
	Mycobacterium angelicum	WP_083112995	-----------------------------		-AA------I--D----V-----A----
	Mycobacterium asiaticum	WP_036352867	------------------------R----		-AA------V-------L--A--AD---
	Mycobacterium bohemicum	WP_085180101	--------------------G------E-		-AA------V-------LE----AD---
	Mycobacterium branderi	WP_083130078	--------------------A------E-		-AA------V--A----V-----LD---
	Mycobacterium conceptionense	CQD06408	--------------------AA--R--E-		-NG------T-------L--D--LD---
	Mycobacterium conspicuum	WP_085233522	-------------R-------------E-		-AA------V---L---------A----
	Mycobacterium dioxanotrophicus	WP_087079202	--------------------A---R--E-		--S---M--T-------Q-----LD---
	Mycobacterium florentinum	WP_085224476	---------------R-----------E-		-AA------V-SA----------AD---
	Mycobacterium fragae	WP_085198651	--------------------A--------		-AA------V-T-L---------L----
	Mycobacterium gastri	WP_036419852	------------A--Q-----------N-		-AS------V-------------A----
	Mycobacterium genavense	WP_025737525	---------------------------Q-		-AA------I---L---------AD---
	Mycobacterium gordonae	WP_055579854	---------------------------N-		-AT------V-------V-----A----
	Mycobacterium haemophilum	WP_047315553	-----------------------------		-AG--------VA-----E----A----
	Mycobacterium heckeshornense	WP_048890208	--------------------A------E-		-AA-----AI-------V--D--L----
	Mycobacterium heidelbergense	WP_083075039	------------------------R--E-		-AA------V------R------DD---
	Mycobacterium holsaticum	WP_069407983	--------------------A---R--E-		-HQ---D--V-------V-----LD---
	Mycobacterium houstonense	WP_066901224	--------------------AA--R----		-NT------T-------L-----LD---
	Mycobacterium interjectum	WP_066907616	---------------------------E-		-AA------V------RV-----AD---
	Mycobacterium intermedium	WP_069417542	---------------------------N-		-AA------V--------E----AD---
	Mycobacterium iranicum	WP_024446272	------------------------R--E-		-HQ---N--T-------VS-D--L----
	Mycobacterium kansasii	KEP43684	------------A--Q-----------N-		-AS------V-------V-----A----
	Mycobacterium kubicae	WP_085073709	------------------------E--N-		-AA------I-D-L---V--V--AD---
	Mycobacterium kyorinense	WP_045382687	--------------------A------E-		-AA------V--A----V-----LD---
	Mycobacterium lacus	WP_085160716	------------------A--------A-		-AA------V-V-----V--G--AD---
	Mycobacterium lentiflavum	WP_090600555	---------------------------Q-		-AA------V--A----------AD---
	Mycobacterium leprae	WP_010907686	---V-------------------------		-TG--------VA---------------
	Mycobacterium lepromatosis	WP_045842426	---L-------------------------		-AG--R-----VA---------------
	Mycobacterium malmoense	WP_083009449	------------------------R--E-		-AA------V------R------DD---
	Mycobacterium marinum	WP_012392995	--------------------A---R--S-		-AA------V-SE----------AD---
	Mycobacterium montefiorense	WP_108921076	---------------R-----------Q-		-AA------V---L---------AD---
	Mycobacterium noviomagense	WP_083088220	--------------------P---E--E-		-AA-----AV-------V--D--L----
	Mycobacterium palustre	WP_085079305	---------------------------E-		-AA------V-------V-----AD---
	Mycobacterium paraense	WP_085096403	---------------------------E-		-AA------V------R------AD---
	Mycobacterium parafortuitum	WP_083145002	--------------------A---R--E-		-HQ---A--T-------------L----
	Mycobacterium porcinum	WP_069424878	--------------------TA--R--E-		-NG------T-------L-----LD---
	Mycobacterium pseudoshottsii J	GAQ32243	--------------------A---R--S-		-AA------V-SE----------AD---
	Mycobacterium rhodesiae	WP_005140080	------------------------R--E-		-AE------V-------V--S--L----
	Mycobacterium riyadhense	WP_085249885	-----------------------------		-AA------V-------V-----A----
	Mycobacterium rutilum	WP_083410136	--------------------A---R--E-		--Q---G--T-------V-----LD---
	Mycobacterium simiae	WP_061558386	---------------R-----------Q-		-AA------V--AL---L-----VD---
	Mycobacterium szulgai	WP_085672406	-----------------------------		-AA------I-TD----V-----A----
	Mycobacterium triplex	WP_036466857	---------------------------Q-		-AA---Q--V-V-L---------AD---
	Mycobacterium ulcerans	WP_096371626	--------------------A---R--S-		-AA------V-SE----------AD---
	Mycobacterium vaccae	WP_003929226	--------------------A---R--E-		-QQ---A--T-------VA----L----
	Mycobacterium vanbaalenii	WP_011778730	--------------------A---R--E-		-HQ---S--T-------V-----L----
	Mycobacterium vulneris	WP_065459559	--------------------TA--R--E-		-NG------T-------L-----LD---
	Mycobacterium wolinskyi	WP_085146662	--------------------A---R--E-		-NA---Q--T---L---V-----LD---
	Mycobacterium xenopi	WP_003919269	--------------------A------E-		-AA------V------AV--G--L----
	Mycolicibacterium aurum	WP_087019590	--------------------A---R--E-		LNS---T--V-------V-----LD---
	Mycolicibacterium austroafricanum	WP_036369935	--------------------A---R--E-		-HQ---S--T-------V-----L----
	Mycolicibacterium bacteremicum	WP_083055630	--------------------A---R-AE-		--S---E--T-------V-----LD---
	Mycolicibacterium boenickei	WP_077742107	--------------------TA--R--E-		-NG------T-------L-----LD---
	Mycolicibacterium brisbanense	WP_062830038	--------------------A---R--E-		-NS---M--T-------Q-----LD---
	Mycolicibacterium chubuense	WP_014814359	------------------------R--E-		LHQ---S--T-S-----V-----L----
	Mycolicibacterium conceptionense	WP_076217425	--------------------AA--R--E-		-NG------T-------L--D--LD---
	Mycolicibacterium confluentis	WP_085149452	------------------------R-TE-		-AA---E--V------IV-----L----
	Mycolicibacterium diernhoferi	WP_073856555	--------------------A---R--E-		LNS---T--V-------V-----LD---
	Mycolicibacterium duvalii	WP_098004183	------------------------R--E-		-HQ---Q--T-------VT-P--L----
	Mycolicibacterium farcinogenes	CDP84891	--------------------AA--R--E-		-NG------T-------L--D--LD---
	Mycolicibacterium flavescens	WP_069416967	--------------------A---R--E-		--Q---G--T-------V-----LD---

Figure S10. Partial sequence alignment of a conserved region of the phospho-sugar mutase / MRSA protein showing a three amino acid insertion that is specific for members of the “Tuberculosis” clade and absent from most other *Mycobacteriaceae*.

Group	Species	Accession	539		586
"Tuberculosis" clade (13/13)	*Mycobacterium tuberculosis*	AIH28258	DCGVVEELTVVTPLVLPTVGGVQLQVVVGVG		EMGQRPVSIYSRNAESD
	Mycobacterium africanum MAL010	KBG21913	-------------------------------		-----------------
	Mycobacterium bovis	WP_069523279	-------------------------------		-----------------
	Mycobacterium bovis AF2122/97	YP_009359027	-------------------------------		-----------------
	Mycobacterium bovis BCG	AMC50496	-------------------------------		-----------------
	Mycobacterium bovis BCG str. P	CAL71688	-------------------------------		-----------------
	Mycobacterium canettii	WP_014000874	-------------------------------		-----------------
	Mycobacterium caprae	WP_075744516	-------------------------------		-----------------
	Mycobacterium microti	AMC59270	-------------------------------		-----------------
	Mycobacterium mungi	WP_064319984	-------------------------------		-----------------
	Mycobacterium orygis	WP_003408182	-------------------------------		-----------------
	Mycobacterium pinnipedii	WP_105826451	-------------------------------		-----------------
	Mycobacterium africanum	KBF86563	-------------------------------		-----------------
Other *Mycobacteriaceae* (0/36)	*Mycobacterium angelicum*	WP_083114726	GY-T-------A--A--M---L-V--L--GA	D	-L-E-S--V---ADAQ-
	Mycobacterium avium	WP_062886498	G---ID---LSA--P--AS---R--L---AP	D	-A-R--L-V--AA-HQ-
	Mycobacterium avium subsp. avium	EUA39396	G---ID---LSA--P--AS---R--L---AP	D	-A-R--L-V--AA-HQ-
	Mycobacterium avium subsp. hominissuis	PBJ40791	G---ID---LSA--P--AS---R--L---AP	D	-A-R--L-V--AA-HQ-
	Mycobacterium avium subsp. paratuberculosis	ANH28144	G---ID---LSA--P--AS---R--L---AP	D	-A-R--L-V--AAVHQ-
	Mycobacterium avium subsp. silvaticum	ETB09015	G---ID---LSA--P--AS---R--L---AP	D	-A-R--L-V--AA-HQ-
	Mycobacterium colombiense	WP_065051421	G---ID---LSA--L--AA----V-L---AP	D	-S-S-RLTV--AG--P-
	Mycobacterium colombiense CECT	EJO86797	G-S------LAA---V-AA-V-H-----AGA	G	DA-T-T-TV---A-Q--
	Mycobacterium conspicuum	WP_085234253	G-S------LLA-M---SA--ARV-I--DPA	G	-S-S-G-WV---GTGP-
	Mycobacterium haemophilum	WP_082607575	G-P-I----LLA------A---PVR-L--AA	G	NS-L-S--V--AD-Q
	Mycobacterium haemophilum DSM	ALL56345	G-P-I----LLA------A---PVR-L--AA	G	NS-L-S--V--AD-Q
	Mycobacterium heidelbergense	ORA66842	G--AI----LSA-----AS-------L--AP	S	-S---E--V--LGPQ-
	Mycobacterium interjectum	WP_085200572	G-S------LSA--I--AP-T-RV-----AA	G	-A-R-A--V--SGVQP-
	Mycobacterium kansasii	WP_063477072	G-AT-A----SA--L---A-AL-V-----AA	D	TS---TA-V---A-QP-
	Mycobacterium kubicae	ORW03439	G-DA------LA-----PTSR-----T--AA	D	-SA----AV---V--P-
	Mycobacterium lacus	WP_085157733	-A-I---VLAA----HPGVA--V-----AA	V	DS-L-A--V---AGQ-
	Mycobacterium lentiflavum	WP_090601745	G-S------LSA--L--ASD---I-----PP	G	SS-R-A--V--LGM-P-
	Mycobacterium lepraemurium	ATA28778	G---ID---LSA--P--AS---R--L---AP	D	-A-R-LL-V--AA-HQ-
	Mycobacterium malmoense	OCB32301	G--AL----LSA-----AAD--RV-----AA	A	-S---E--V--AG-Q--
	Mycobacterium marinum	WP_103759350	G--------LA-----NAGTA--V-----SA	G	QS---L--M---ADQP-
	Mycobacterium palustre	WP_085080838	G-PAI----LSA-----AS--TR------ES	G	-S-R-S-----AGSQP-
	Mycobacterium parascrofulaceum	EFG79593	G--AL----LSA-----AAD--RV-----AA	V	-S---E--V--AG-Q--
	Mycobacterium pseudoshottsii	WP_106407457	G--------LA-----NAGTA--V-----SA	G	QS---L--M---ADQP-
	Mycobacterium pseudoshottsii J	GAQ40085	G--------LA-----NAGTA--V-----SA	G	QS---L--M---ADQP-
	Mycobacterium riyadhense	WP_085249718	G----------A--A--A----R------AL	D	DS---A--V---A----
	Mycobacterium riyadhense	WP_085249719	G--------LLA-----PA--MRV--L--DA	G	-S-R-A--V--HGMAA-
	Mycobacterium saskatchewanense	WP_085256883	G-A-I----LSA-----AS-A-RV----DGA	G	-DQS----V--SA-Q--
	Mycobacterium scrofulaceum	WP_083175478	G--AL----LSA-----AAD--RV-----AA	T	-S---E--V--AG-Q-E
	Mycobacterium shigaense	WP_096439716	G-T------LSA--L--AAD--HV-----AA	A	DS-R-A--V---GTQP-
	Mycobacterium shinjukuense	WP_083052204	G-RL------LA------T---R-R----TV	D	-LQK-AL-----T--A-
	Mycobacterium szulgai	OBF21827	G-DA------LA-----PTSR-----T--AA	D	-SA----AV---V--P-
	Mycobacterium timonense	WP_083187138	G---ID---LSA--P--ES---R--L---AP	D	-S-R--L-V--AA-HQ-
	Mycobacterium triplex	WP_036468893	G-S--D---LLA-----PG-AARV----DAA	G	-S-S-A-WV---G-A--
	Mycobacterium xenopi	WP_085196881	G-S--D---LR---IV--S-S-TV-----AA	A	-S-E-G--V---S
	Mycobacterium xenopi 3993	EUA44472	G-S--D---LR---IV--S-S-TV-----AA	A	-S-E-G--V---S
	Mycobacterium xenopi 4042	EUA42231	G-S--D---LR---IV--S-S-TV-----AA	A	-S-E-G--V---S

Figure S11. Partial sequence alignment of a conserved region of the polyketide synthase Pks8 protein showing a one amino acid deletion that is specific for members of the "Tuberculosis" clade.

```
                                                      584                                                         641
Mycobacterium tuberculosis            AUS51591        EFGEKVGEVLQSVLDTEITPELIPTGEEE   LQSSEAKVGPFALQDFSLFQVLRYGFRPS
Mycobacterium bovis                   WP_044798297    -----------------------------   -----------------------------
Mycobacterium canettii                WP_015290669    D----------------------------   -----------------------------
Mycobacterium caprae                  APU27946        -----------------------------   -----------T-----------------
Mycobacterium microti                 PRI03803        -----------------------------   -----------------------------
Mycobacterium mungi                   OAQ17596        -----------------------------   -----------------------------
Mycobacterium orygis                  WP_003412565    -----------------------------   -----------------------------
Mycobacterium pinnipedii              PRH91190        -----------------------------   -----------------------------
Mycobacterium africanum               WP_031669376    -----------------------------   -----------------------------
Mycobacterium angelicum               WP_083114318    --EDE-S---------------V-S---- E -------I---------------F-----
Mycobacterium avium                   WP_009975982    --APE--A--------------V-S---- E -------------------H---------
Mycobacterium fragae                  WP_085199556    Q-D-Q-CD--------------V-S---- E -----------------------F-----
Mycobacterium kansasii                ORB83221        --D-H-----------------V-S---- E -------------------H---F-----
Mycobacterium kansasii 732            EUA13039        --D-Q-C---------------V-S---- E -------------------H---F-----
Mycobacterium lacus                   WP_085162935    Q-DDE-SAT-------------V---AD- E I-R--ST---YV-------C---------
Mycobacterium lentiflavum             WP_090604586    --ESE-DD--------------V------ E -------I--Y--------H---------
Mycobacterium lepraemurium            ATA28970        --APE--A--------------V-S---- E -------------------H---------
Mycobacterium litorale                WP_078018699    --SGE---I-------------V----D- E I---------YS-----------------
Mycobacterium mageritense             WP_036443341    Q--PE-N-I-------------V----D- E I---------YV-----------------
Mycobacterium mageritense DSM         CDO26891        Q--PE-N-I-------------V----D- E I---------YV-----------------
Mycobacterium malmoense               WP_071513343    Q-ESR-D-----------------S---- E -------I-----------H---------
Mycobacterium microti                 WP_101528156    --SSD-A---------------V-S---- E -------------------H---F-----
Mycobacterium montefiorense           WP_108925505    Q-ESD-D---------------------- E ----------Y--------Y---------
Mycobacterium neworleansense          CRZ13529        Q--GE-N---------------V----D- E I---------Y------------------
Mycobacterium parmense                WP_085267314    Q-EPE-C---------------V-S---- E -------------------H---------
Mycobacterium peregrinum              WP_064931113    Q--AE-ND--------------V----D- E I---------Y------------------
Mycobacterium persicum                WP_083153202    --DQQ-C---------------V-S---- E -----------------------F-----
Mycobacterium rhodesiae               WP_005144992    Q-EDE---I-------------V----D- E I---------YS-----------------
Mycobacterium riyadhense              WP_085250756    --D-N-S---------------V------ E ----------YV-----------------
Mycobacterium septicum                WP_044522879    Q--VE-N---------------V----D- E I---------Y------------H-----
Mycobacterium setense                 WP_039322195    ---PD-N---------------V----D- E I---------YV-----------------
Mycobacterium sherrisii               WP_085167533    Q-SSD-A---------------V-S---- E -------------------H---F-----
Mycobacterium shinjukuense            WP_083046283    --D-Q------------------------ E -------I-----------W---------
Mycobacterium simiae                  WP_044507059    --SSD-A---------------V-S---- E -------------------H---F-----
Mycobacterium smegmatis               WP_058126631    --DDE-N---------------V----D- E I---------Y------------F-----
Mycobacterium sphagni                 WP_094481328    Q-EDE---I-----E-------V----D- E I---------YS-----------------
Mycobacterium szulgai                 WP_085670293    --EDE-S---------------V-S---- E -------I---------------F-----
Mycobacterium xenopi 3993             EUA18238        Q-DDE-CAT-------------V------ E I---------YV-------H---------
Mycolicibacterium aromaticivorans     WP_036344405    --SDE--DI-------------V----D- E I---------YS-----------------
Mycolicibacterium aurum               WP_048633195    --DAA-ND--------------V----D- E I---------Y------------F-----
Mycolicibacterium boenickei           WP_077742040    Q--AE-N---------------V----D- E I---------Y------------H-----
Mycolicibacterium goodii              WP_049745814    D-EDE-N---------------V----D- E I---------Y------------F-----
```

"Tuberculosis" clade (9/9) — *Mycobacterium tuberculosis* to *Mycobacterium africanum*

Other *Mycobacteriaceae* (0/33) — *Mycobacterium angelicum* to *Mycolicibacterium goodii*

Figure S12. Partial sequence alignment of a conserved region of the Glutamine-dependent NAD(+) synthetase protein showing a one amino acid deletion that is specific for members of the "Tuberculosis" clade.

Group	Species	Accession	219		269
"Tuberculosis" clade (9/9)	*Mycobacterium tuberculosis*	AMC73876	EDENGSLEAGNRRRRRRRRRKSASGDDND	AAL	EGPLPDDPPNTVVHERVPR
	Mycobacterium bovis	WP_046026288	-----------------------------	---	-------------------
	Mycobacterium canettii	CCC44814	-----------------------------	---	-------------------
	Mycobacterium caprae	APU26422	-----------------------------	---	-------------------
	Mycobacterium microti	AMC60139	-----------------------------	---	-------------------
	Mycobacterium mungi	OAQ17568	-----------------------------	---	-------------------
	Mycobacterium orygis	EMT35239	-----------------------------	---	-------------------
	Mycobacterium pinnipedii	PRH91194	-----------------------------	---	-------------------
	Mycobacterium africanum	AMC64751	-----------------------------	---	-------------------
Other *Mycobacteriaceae* (0/41)	*Mycobacterium angelicum*	WP_083115582	--D---A---------------GA-----		---A------------A--
	Mycobacterium arosiense	WP_083062904	-D--ADG-------------G-A----		-SSS------------P--
	Mycobacterium avium	WP_009975970	DG-------------G-----E		D--S------------P--
	Mycobacterium avium 09-5983	ETB26381	DG-------------G-----E		D--S------------P--
	Mycobacterium avium 11-0986	ETB48540	DG-------------G-----E		D--S------------P--
	Mycobacterium avium MAV_120709	ETZ41629	DG-------------G-----E		D--S------------P--
	Mycobacterium avium MAV_120809	ETZ43215	DG-------------G-----E		D--S------------P--
	Mycobacterium avium subsp. avium	AIV27363	DG-------------G-----E		D--S------------P--
	Mycobacterium avium subsp. hominissuis	BAN30595	DG-------------G-----E		D--S------------P--
	Mycobacterium chimaera	WP_085081460	--G------		---S------------P--
	Mycobacterium colombiense	WP_064952991	-------------G------		--SS------------S--
	Mycobacterium conspicuum	WP_085232598	D-----G--ATK----------G------		---S------------A--
	Mycobacterium fragae	WP_085199549	---A-TAT----------AG----GE		D--S------------A--
	Mycobacterium gastri	WP_084293324	---A-GAS----------PG--E-G-		---A------------A--
	Mycobacterium gastri 'Wayne'	ETW26284	---A-GAS----------PG--E-G-		---A------------A--
	Mycobacterium heckeshornense	WP_048892718	--P-I-T---------R-G----G-		N--A---------------
	Mycobacterium heidelbergense	WP_083072030	D-----SD-AT-----------G------		--LS------------P--
	Mycobacterium intermedium	WP_069417392	DE----T---S----------AG-S----		---A------------A--
	Mycobacterium intracellulare	WP_026071538	--G------		---S------------P--
	Mycobacterium intracellulare 1	EUA58335	--G------		---S------------P--
	Mycobacterium intracellulare A	AFC43027	--G------		---S------------P--
	Mycobacterium intracellulare M	AFC48154	--G------		---S------------P--
	Mycobacterium intracellulare s	AFS13725	--G------		---S------------P--
	Mycobacterium kansasii	OOK80033	---A-GAS-----------G--E-G-		---A------------A--
	Mycobacterium kansasii ATCC 12	AGZ49771	---A-GAS-----------G--E-G-		---A------------A--
	Mycobacterium kubicae	ORW02422	----AD--S----------TG-A---E		D--A------------P--
	Mycobacterium lacus	WP_085162941	-ED--PA-V-S-----------G-----E		D--A------------A--
	Mycobacterium lepraemurium	ATA28976	DG--ADG-------------G-----E		D--S------------P--
	Mycobacterium marinum	BBC67138	D-D---ADGA------------GA---G-		---A------------A--
	Mycobacterium paraintracellulare	AFC53063	--G------		---S------------P--
	Mycobacterium persicum	WP_083153200	---A-GAS-----------G--E-G-		---A------------A--
	Mycobacterium pseudoshottsii	WP_086085553	D-D---ADGAS-----------GA---G-		---A------------A--
	Mycobacterium pseudoshottsii J	GAQ38324	D-D---ADGAS-----------GA---G-		---A------------A--
	Mycobacterium shigaense	WP_096441419	---ADG-------------G-A----		-S-S------------A--
	Mycobacterium shinjukuense	WP_083046278	-AD---P---T-----------GA-E-S-		---A------------A--
	Mycobacterium szulgai	WP_085669807	--D---A---------------GA-----		---A------------A--
	Mycobacterium ulcerans	WP_011741442	D-D---ADGAS-----------GA---G-		---A------------A--
	Mycobacterium ulcerans str. Harvey	EUA87319	D-D---ADGAS-----------GA---G-		---A------------A-
	Mycobacterium xenopi	WP_085193537	--TA-S-I-T---------R-G----G-		N--A------------E--
	Mycobacterium xenopi 4042	EUA33215	--TA-S-I-T---------R-G----G-		N--A------------E--
	Mycobacterium xenopi RIVM70036	EID13549	--TA-S-I-T---------R-G----G-		N--A------------E--

Figure S13. Partial sequence alignment of a conserved region of the ribonuclease E protein showing a three amino acid insertion that is specific for members of the "Tuberculosis" clade.

Clade	Species	Accession	111		170
"Tuberculosis" clade (9/9)	*Mycobacterium tuberculosis*	AUS51603	DGKPISPAQYVATYREIEPLVALIDQQSQAS	AGK	GGPAMSKFEVLTAMAFAAFXDAPVDV
	Mycobacterium bovis	WP_047709789	------------A------------------	---	-------------------A------
	Mycobacterium canettii	WP_014001276	--------------------Q----------	---	-------------------A------
	Mycobacterium caprae	APU26424	-------------------------------	---	-------------------A------
	Mycobacterium microti	WP_105799763	-------------------------------	---	-------------------A------
	Mycobacterium mungi	OAQ17566	-------------------------------	---	-------------------A------
	Mycobacterium orygis	WP_003412601	-------------------------------	---	-------------------A------
	Mycobacterium pinnipedii	PRH91196	-------------------------------	---	-------------------A------
	Mycobacterium africanum	CCC27534	-------------------------------	---	-------------------A------
Other *Mycobacteriaceae* (0/53)	*Mycobacterium angelicum*	WP_083115584	-------------------F-QM--E----A		A------------------A---I--
	Mycobacterium asiaticum	WP_036358460	--R--T-----E-------YIQI-------A		-------------------A------
	Mycobacterium avium	WP_019729012	-A------R--D-------F-QMV------A		-------------------A------
	Mycobacterium avium 11-0986	ETB48559	-A------R--D-------F-QMV------A		-------------------A------
	Mycobacterium avium subsp. avium	EUA38950	-A------R--D-------F-QMV------A		-------------------A------
	Mycobacterium chimaera	KPN59055	-A-----------------F-QMV-A----A		-------------------A------
	Mycobacterium colombiense	WP_064878455	-S-----------------F-QMV-A----E		-------------------A------
	Mycobacterium haemophilum	WP_054879689	-A-----------------F-QM--A---S-		----L-----V--------A-T-I-I
	Mycobacterium holsaticum	WP_069403881	-D-----------------F-Q-V----E-A		----------V--------A---I--
	Mycobacterium intermedium	WP_069417390	----V-------A--D---F-Q-V------A		-------------------A------
	Mycobacterium intracellulare	WP_095579945	-A-----------------F-QMV-A----A		-------------------A------
	Mycobacterium intracellulare M	ETZ32811	-A-----------------F-QMV-A----A		-------------------A------
	Mycobacterium kansasii	OOK78672	-------------------F-QM--E---NA		-------------------A------
	Mycobacterium kubicae	WP_085073382	-------G-----------F-HMV-E----A		-------------------A------
	Mycobacterium lacus	WP_085162944	----------------L----QMV-R----G		------------
	Mycobacterium liflandii	WP_015356543	-----T-----Q-------F-QM-------A		-------------------A------
	Mycobacterium mageritense	WP_036442620	-----T-----E-------F-E-V----E-A		---K---------------A---I--
	Mycobacterium mantenii	WP_083096959	-S-----------------F-QMV-A----D		-------------------A------
	Mycobacterium marinum	WP_012395379	-----T-----Q-------F-QM-------A		-------------------A------
	Mycobacterium nebraskense	WP_046185970	--R--T-------------F-HM--A----A		-------------------A------
	Mycobacterium neworleansense	WP_090515339	-----T--H--E-------F-Q-V----E-A		---K---------------A-V-I--
	Mycobacterium paraffinicum	WP_073880171	--R----------------F-QM--A--E-A		-------------------A------
	Mycobacterium peregrinum	WP_055120406	----VT--R--E-------F-E-V----E-A		---K---------------A-V-I--
	Mycobacterium persicum	WP_083153199	-------------------F-QM--E---NA		-------------------A------
	Mycobacterium porcinum	WP_069424579	-----T-----N-------F-E-V-R--E-A		---K---------------A-I-I--
	Mycobacterium riyadhense	WP_085249494	-------------------F-QM-------A		D------------------A------
	Mycobacterium saskatchewanense	WP_085258011	-S-----------------F-QMV-A----E		------------G------A------
	Mycobacterium scrofulaceum	WP_067275374	--R--T-------------F-QM--A----A		-------------------A------
	Mycobacterium septicum	WP_044522831	--M--T-----N--T----F-E-V-R--E-A		---K---------------A-V-I--
	Mycobacterium shinjukuense	WP_083046275	--L----------------F-QM-----S-A		----------V--------A------
	Mycobacterium smegmatis	WP_003896006	--------------T----F-Q-V----E-A		---K---------------A---I--
	Mycobacterium smegmatis str. M	AFP40966	--------------T----F-Q-V----E-A		---K---------------A---I--
	Mycobacterium sphagni	WP_094481304	-NE-----R--EV------F-H-V-S----G		------------G------A------
	Mycobacterium szulgai	WP_068033555	-------G-----------F-HMV-E----T		-------------------A------
	Mycobacterium talmoniae	WP_071028613	--Q--T--------A----F-QMV-A----D		------------G------A----E-
	Mycobacterium tusciae	WP_006241188	--L-LT--K--E-------F-H-V----EEA		----------V-G------A---I--
	Mycobacterium ulcerans	WP_096371379	-----T-----Q-------F-QM-------A		-------------------A------
	Mycobacterium ulcerans str. Harvey	EUA87333	-----T-----Q-------F-QM-------A		-------------------A------
	Mycobacterium vulneris	WP_065461843	-----T-----N-------F-E-V-R--E-A		---K---------------A-V-I--
	Mycobacterium wolinskyi	WP_085148578	--------H--Q--T----F-Q-V----E-A		----------------S--A---I--
	Mycobacterium xenopi	WP_003921045	--------D------D---Y-QM------S-		-------------------A------
	Mycobacteroides abscessus 1948	EUA60091	--Q-----K--EI-S----F-E-V-K---EQ		-----------V----V--A-----I
	Mycolicibacter arupensis	KKC01386	--R----GR--E--A----F-QMV-AS---D		------------G------A------
	Mycolicibacter engbaekii	WP_085129677	--E-----R--Q--T----F-QMV-AS---D		------------G------A------
	Mycolicibacter heraklionensis	OBG37122	--Q-----R--E--T----F-QMV-AS---D		------------G------A------
	Mycolicibacter icosiumassiliensis	WP_096867715	--Q-----R--E--A----F-QMV-AS---D		------------G------A------
	Mycolicibacter longobardus	WP_085264279	--E-----R--E--A----F-QMV-AS---D		------------G------A------
	Mycolicibacter nonchromogenicus	WP_085139620	--Q-----R--E--T----F-QMV-AS---D		------------G------A------
	Mycolicibacterium boenickei	WP_077741364	-----T-----D-------F-E-V-R--E-A		---K---------------A-V-I--
	Mycolicibacterium canariasense	WP_062657473	-------G-----------F-E-V-A--E-A		---K--------V------A------
	Mycolicibacterium conceptionense	WP_064899406	-----T--R--E-------F-H-V----E-A		---K---------------A-V-I--
	Mycolicibacterium farcinogenes	WP_036393540	-----T--R--E-------F-H-V----E-A		---K---------------A-V-I--
	Mycolicibacterium fortuitum	WP_054602728	--M--T--R--E-------F-H-V----E-A		---K---------------A-V-I--

Figure S14. Partial sequence alignment of a conserved region of the folylpolyglutamate synthase protein FOLC showing a three amino acid insertion that is specific for members of the "Tuberculosis" clade.

Group	Species	Accession	392		440
"Tuberculosis" clade (12/12)	*Mycobacterium tuberculosis*	AAC44599	EAHEAIRPAGETFATPDAVRRELDG	PNI	DDFRLYELIWQRTVASQMADA
	Mycobacterium bovis	WP_080659562	-------------------------	---	---------------------
	Mycobacterium bovis AF2122/97	YP_009361030	-------------------------	---	---------------------
	Mycobacterium bovis BCG	CUI13679	-------------------------	---	---------------------
	Mycobacterium bovis BCG str. P	CAL73693	-------------------------	---	---------------------
	Mycobacterium canettii	WP_015291611	-------------------------	---	---------------------
	Mycobacterium caprae	APU27451	-------------------------	---	---------------------
	Mycobacterium microti	PRI06602	-------------------------	---	---------------------
	Mycobacterium mungi	OAQ17167	-------------------------	---	---------------------
	Mycobacterium orygis	WP_003419640	-------------------------	---	---------------------
	Mycobacterium pinnipedii	PRH91560	-------------------------	---	---------------------
	Mycobacterium africanum	CCC28722	-------------------------	---	---------------------
Other *Mycobacteriaceae* (0/>100)	*Mycobacterium alsense*	WP_083138393	-------------------------		---------------------
	Mycobacterium angelicum	WP_083115815	-------------------------		-E------V------------
	Mycobacterium arosiense	WP_083066595	-------------------------		---------------------
	Mycobacterium asiaticum	WP_065035927	-------------------------		-E-------------------
	Mycobacterium avium	WP_084024827	-------------------------		-E-------------------
	Mycobacterium avium subsp. avium	EUA25067	-------------------------		-E-------------------
	Mycobacterium avium subsp. paratuberculosis	ETA98054	-------------------------		-E-------------------
	Mycobacterium bohemicum	WP_085182349	----------D------------A-		---------------------
	Mycobacterium branderi	WP_083131194	-------------------------		---------------------
	Mycobacterium celatum	WP_062541537	-------------------------		---------------------
	Mycobacterium chimaera	WP_095662509	-------------------------		-E-------------------
	Mycobacterium colombiense	WP_064885253	-------------------------		-E--I----------------
	Mycobacterium colombiense CECT	EJO89075	-------------------------		-E--I----------------
	Mycobacterium conspicuum	WP_085232731	--------------------H--G-		-E-------------------
	Mycobacterium europaeum	WP_085241416	-----------A-------------		-E-------------------
	Mycobacterium florentinum	WP_085223181	-------------------------		-E-------------------
	Mycobacterium fragae	WP_085200167	-------------------------		---------------------
	Mycobacterium gastri	WP_036411215	-------------------------		-E-------------------
	Mycobacterium gastri 'Wayne'	ETW25367	-------------------------		-E-------------------
	Mycobacterium genavense	WP_025736321	-------------------C-----		-E-------------------
	Mycobacterium gordonae	WP_055576781	-------------------------		-E-------------------
	Mycobacterium haemophilum	WP_047316417	-------------------------		-E------V------------
	Mycobacterium hassiacum	WP_018354201	----------DV----GQLHA---A		-E-------------------
	Mycobacterium hassiacum DSM 44	EKF22101	----------DV----GQLHA---A		-E-------------------
	Mycobacterium heckeshornense	WP_071700308	-------------------------		---------------------
	Mycobacterium heidelbergense	WP_083073758	-------------------------		-E-------------------
	Mycobacterium holsaticum	WP_069404806	----------DV-S--GQLHSQ--T		-E-------------------
	Mycobacterium insubricum	WP_083029258	----------DV------------S		-E-------------------
	Mycobacterium interjectum	WP_066916583	-------------------------		-E-------------------
	Mycobacterium intermedium	WP_069419406	--------------------H----		-E-------------------
	Mycobacterium intracellulare	WP_064938671	-------------------------		-E-------------------
	Mycobacterium intracellulare 1	EUA57053	-------------------------		-E-------------------
	Mycobacterium kansasii	WP_063468661	-----------A------------N		-E------V------------
	Mycobacterium kansasii 732	EUA05983	-----------A------------N		-E------V------------
	Mycobacterium kansasii 824	ETZ96908	------------------------S		-E------V------------
	Mycobacterium kubicae	WP_085074662	-------------------------		-E------V------------
	Mycobacterium kyorinense	WP_065012742	-------------------------		-E-------------------
	Mycobacterium lacus	WP_085158498	-------------------------		-E-------------------
	Mycobacterium lentiflavum	WP_090607446	-------------------------		-E-------------------
	Mycobacterium leprae	WP_010907592	-------------------CN----		-E--I---V-R----------
	Mycobacterium leprae 3125609	OAR19947	-------------------CN----		-E--I---V-R----------
	Mycobacterium lepraemurium	ATA27570	-------------------------		-E-------------------
	Mycobacterium lepromatosis	KJX75809	--------------------G---S		-E--I---V-R----------
	Mycobacterium liflandii	WP_015357483	-------------------------		-E-------------------
	Mycobacterium malmoense	WP_065445873	-----------A-------------		-E-------------------
	Mycobacterium mantenii	WP_083098967	-------------------------		-E--I----------------
	Mycobacterium marinum	WP_094360861	-------------------------		-E-------------------
	Mycobacterium marinum str. Europe	EPQ71907	-------------------------		-E-------------------
	Mycobacterium marseillense	WP_083019068	-------------------------		-E-------------------
	Mycobacterium montefiorense	WP_108920271	-------------------------		-E-------------------
	Mycobacterium nebraskense	WP_046186104	-------------------------		-E-------------------
	Mycobacterium noviomagense	WP_083088597	-----------------------N-		---------------------
	Mycobacterium obuense	WP_046364506	----------DV----GQLHSA--T		-E-------------------
	Mycobacterium palustre	WP_085079430	-------------------------		-E-------------------
	Mycobacterium paraense	WP_085103368	-------------------------		-E-------------------
	Mycobacterium paraffinicum	WP_073871461	-----------A-------------		-E-------------------
	Mycobacterium paraintracellulare	WP_014383732	-------------------------		-E-------------------
	Mycobacterium paraseoulense	WP_083175956	-------------------------		-E-------------------
	Mycobacterium parmense	WP_085267343	----------D--------------		-E-------------------
	Mycobacterium persicum	WP_083156050	-------------------------		-E-------------------
	Mycobacterium pseudoshottsii	WP_086084796	-------------------------		-E-------------------

	Mycobacterium pseudoshottsii J	GAQ32284	-------------------------	-E-------------------
	Mycobacterium rhodesiae	WP_083120803	----------DV----G-LH----T	-E-------------------
	Mycobacterium riyadhense	WP_085252094	-----------------------N-	-E------V------------
	Mycobacterium saskatchewanense	WP_085255252	----------DA-------------	-E-------------------
	Mycobacterium scrofulaceum	WP_067270659	-----------A-------------	-E-------------------
	Mycobacterium sherrisii	WP_069400778	-------------------------	-E-------------------
	Mycobacterium shigaense	BAX94748	-------------------------	-E-------------------
	Mycobacterium shimoidei	WP_069394709	-----------------------N-	---------------------
	Mycobacterium shinjukuense	WP_083051286	-------------------------	-E-------------------
	Mycobacterium simiae	WP_061557414	-------------------------	-E-------------------
	Mycobacterium sphagni	WP_094479679	----------D-----G-LH----N	-E-------------------
	Mycobacterium szulgai	WP_068023088	-------------------------	-E------V------------
	Mycobacterium talmoniae	WP_105368780	-------------------------	---------------------
	Mycobacterium triplex	WP_036471856	-------------------------	-E-------------------
	Mycobacterium tusciae	WP_006243846	----------DV-S--GQLHAQ--T	-E-------------------
	Mycobacterium ulcerans	WP_096369565	-------------------------	-E-------------------
	Mycobacterium ulcerans str. Harvey	EUA86576	-------------------------	-E-------------------
	Mycobacterium vulneris	WP_085290802	-------------------------	-E--I----------------
	Mycobacterium xenopi	WP_039890612	-------------------------	---------------------
	Mycobacterium xenopi 3993	EUA24298	-------------------------	---------------------
	Mycobacterium xenopi 4042	EUA23439	-------------------------	---------------------
	Mycobacterium xenopi RIVM70036	EID12255	-------------------------	---------------------
	Mycobacteroides abscessus	WP_062878555	-------------K--GQLHSS--N	-E-------------------
	Mycobacteroides abscessus 1948	EUA64294	-------------K--GQLHSS--N	-E-------------------
	*Mycobacteroides abscessus MAB_*	ETZ87592	-------------K--GQLHSS--N	-E-------------------
**Other *Mycobacteriaceae* (0/>100)**	*Mycobacteroides abscessus MAB_*	ETZ94722	-------------K--GQLHSS--N	-E-------------------
	Mycobacteroides abscessus subsp. abscessus	SIL00731	-------------K--GQLHSS--N	-E-------------------
	Mycobacteroides immunogenum	WP_064630663	-------------K--GQLHSS--N	-E-------------------
	Mycobacteroides saopaulense	WP_088413098	-------------K--GQLHSS--N	-E-------------------
	Mycolicibacillus koreensis	OSC33707	----------D---------G---S	---------------------
	Mycolicibacillus trivialis	WP_085110452	----------D---------N----	-E-------------------
	Mycolicibacter algericus	WP_083040190	--------------------N--GS	-E-------------------
	Mycolicibacter arupensis	WP_046189722	--------------------N--GS	-E-------------------
	Mycolicibacter engbaekii	WP_085128725	--------------------N--GS	-E-------------------
	Mycolicibacter heraklionensis	OBK84822	--------------------N--GS	-E-------------------
	Mycolicibacter hiberniae	WP_085134075	--------------------N--GS	-E-------------------
	Mycolicibacter icosiumassiliensis	WP_067976690	--------------------N--GS	-E-------------------
	Mycolicibacter kumamotonensis	WP_065289399	--------------------N--GS	-E-------------------
	Mycolicibacter longobardus	WP_085265466	--------------------N--GS	-E-------------------
	Mycolicibacter minnesotensis	WP_083026078	--------------------N--GS	-E-------------------
	Mycolicibacter nonchromogenicus	WP_085138940	--------------------N--GS	-E-------------------
	Mycolicibacter senuensis	ORW66399	--------------------N--GS	-E-------------------
	Mycolicibacter sinensis	WP_049792954	--------------------N--GS	-E-------------------
	Mycolicibacter terrae	WP_085260311	--------------------N--GS	-E-------------------
	Mycolicibacterium agri	WP_097937617	----------DV----GQLHS---T	-E-------------------
	Mycolicibacterium aromaticivorans	WP_036341814	----------DV----G-LH----T	-E-------------------
	Mycolicibacterium brumae	WP_090588990	--------S-D-------------S	-EH------------------
	Mycolicibacterium celeriflavum	WP_083000270	----------DV-S--GQLHGQ--T	-E-------------------
	Mycolicibacterium confluentis	WP_085152370	----------D-------------N	-E-------------------
	Mycolicibacterium duvalii	WP_098006267	----------DV-S--GQLHSQ--N	-E-------------------
	Mycolicibacterium fallax	WP_085094967	----------D------------EN	-E-------------------

Figure S15. Partial sequence alignment of a conserved region of the DNA topoisomerase I protein showing a three amino acid insertion that is specific for members of the “Tuberculosis” clade.

Group	Species	Accession	963		1014
"Tuberculosis" clade (16/16)	*Mycobacterium tuberculosis*	AMC67037	CFDKTGTLSENRLRVAQVRPVAGHSREEVLRCAAHAAPASNG		PQVHATDVAI
	Mycobacterium africanum MAL010	KBG16644	--		----------
	Mycobacterium africanum MAL020	KBH72480	--		----------
	Mycobacterium bovis	WP_080698901	--		----------
	Mycobacterium bovis AF2122/97	YP_009357762	--		----------
	Mycobacterium bovis B2 7505	KAO03132	--		----------
	Mycobacterium bovis BCG	AMC48981	--		----------
	Mycobacterium bovis Bz 31150	KAN87274	--		----------
	Mycobacterium canettii	WP_014000222	------------------------P---------R-------		----------
	Mycobacterium canettii CIPT 14	CCK62494	--		----------
	Mycobacterium caprae	CEJ50837	--		----------
	Mycobacterium microti	AMC57903	--		----------
	Mycobacterium mungi	WP_064319831	--		----------
	Mycobacterium orygis 112400015	EMT37281	--		----------
	Mycobacterium pinnipedii	WP_105826288	--		----------
	Mycobacterium africanum	WP_031666816	--		----------
Other *Mycobacteriaceae* (0/75)	*Mycobacterium angelicum*	WP_083116278	--------------------AP----D------V----P---	G	--I-------
	Mycobacterium arosiense	WP_083064249	---------------S--H-A------------V----VT--	G	T---------
	Mycobacterium avium	WP_052312205	---------------TA-H-MPKYTDDD------N----PE-	A	LHA----Q-V
	Mycobacterium bohemicum	WP_085179422	------------------H-AP------------Q-------	D	-H-----A--
	Mycobacterium branderi	ORA32068	---------------S--H-----------H-------TA--	N	-H-----R--
	Mycobacterium celatum	WP_085167383	---------------S--H----R------H-------TA--	N	-H-----R--
	Mycobacterium chimaera	WP_072501446	---------------S--H-APRY---------V----VT--	G	T---------
	Mycobacterium conspicuum	WP_085235678	---------------SR-H-AP-G-EPD----------VTD-	G	--L----
	Mycobacterium europaeum	ORV46990	----------------E---M-----DD------R----DD-	N	-H-----E--
	Mycobacterium fragae	ORV59894	------------------H--GSY---D----------TA--	G	-H-----R--
	Mycobacterium gastri	WP_036418706	----------------K-H-AP-Y-GD------V-----T--	G	--L-------
	Mycobacterium grossiae	WP_070353581	---------------TS-DALN-ED-DG---A-VR-T-RA--	D	GHE----A-V
	Mycobacterium haemophilum	WP_082129398	------------------H-AP-VT--------------T-C	G	--I----
	Mycobacterium heckeshornense	WP_082169443	----------------R-H--S-----------------A--	G	--I-------
	Mycobacterium interjectum	WP_085202860	--------------------AP---P----HD-------A--	N	----------
	Mycobacterium intracellulare	WP_064934959	---------------SH-H-AP-Y---------V----VT--	G	T---------
	Mycobacterium kansasii	KZS66907	----------------K---AP-Y-AD------V-----T--	G	--L-------
	Mycobacterium kubicae	WP_085075078	---------------TR-Q-AP-QPS------------P---	G	--I-------
	Mycobacterium kyorinense	WP_065014205	------------------H--G--------GY----T-P---	T	--I----R--
	Mycobacterium lacus	WP_085157902	---------------S--H-AP-Y--A-----------VT--	G	R-A----
	Mycobacterium liflandii	WP_051045858	------------------H-GP-F-DHD-----V-----T--	G	----------
	Mycobacterium malmoense	WP_065445701	---------Q------E-VAAP-T--D-----------PA--	N	-H--------
	Mycobacterium marinum	WP_094361511	------------------H-GP-F-DHD-----V-----T--	G	----------
	Mycobacterium marseillense	WP_095577869	---------------S--H-A------------V----MT--	G	T---------
	Mycobacterium nebraskense	WP_082123071	----------------K-H---E---DD------Q----DD-	N	-H-----E--
	Mycobacterium noviomagense	ORB14502	------------------HT-D-Y-Q------------TV--	G	--I-------
	Mycobacterium palustre	WP_085077492	--------------------AP-L---------------A--	G	--A-------
	Mycobacterium paraense	WP_085092928	--------------------AP-R-P----HD-------A--	N	----------
	Mycobacterium paraffinicum	WP_073877923	----------------E-Q---E----D------Q----DD-	N	-H-----E--
	Mycobacterium parascrofulaceum	WP_083802020	----------------E-L-LV------------Q----DD-	N	-H-----E--
	Mycobacterium paraseoulense	WP_083175570	---------Q------E-VAAP-T------------------	G	-H--------
	Mycobacterium parmense	WP_085271735	---------Q------E---A-D---------------PT--	N	-HI----A--
	Mycobacterium pseudoshottsii	WP_086085303	------------------H-GP-F-DHD-----V-----A--	G	----------
	Mycobacterium riyadhense	WP_085248205	---------------SR-H-AP-Y--A-----------VT--	G	------
	Mycobacterium szulgai	WP_068022576	---------------TR-Q-AP-QPS------------P---	G	--I-------
	Mycobacterium talmoniae	OHV03555	---------------T--H-----TDDD-----V-----PE-	T	AHA----Q--
	Mycobacterium ulcerans	WP_083421612	-----E------------H-GP-F-DHD-----V-----T--	G	----------
	Mycobacterium wolinskyi	WP_084356640	---------------SE-Q-AGKS---Q--D---R-T-PK--	D	HHE----A-V
	Mycobacterium xenopi	WP_085193134	----------------R-H--S-Y--------------TA--	G	--I-------
	Mycolicibacillus koreensis	WP_109561785	---------------TG-H--T-Y-DD---AD-V----SPD-	A	AHA----Q--
	Mycolicibacillus trivialis	WP_085110447	---------------TA-Q-LP--ASDD--VA-MQ----PEE	A	QHA----Q--
	Mycolicibacter algericus	WP_083036713	---------Q-----T-LT-AS-Y-TD---AF--Q----T--	R	Q -----
	Mycolicibacter arupensis	WP_083070937	----------------T---L--Y-NDD--GT--Q----PD-	A	AHA----Q--
	Mycolicibacter engbaekii	WP_109560965	---------------TT---LT-YTDDD--GT--Q----PD-	A	AHA----Q--
	Mycolicibacter heraklionensis	OBI05291	---------------TR-Y-----TH-D------N---SPE-	D	-HT----Q-V
	Mycolicibacter hiberniae	WP_109559887	---------R-----S--H-AS-F-D-D---F--S---VP--	A	R------T--
	Mycolicibacter icosiumassiliense	WP_067966546	---------K-----S--H-A--FAEHD---F--S---V---	V	R------T--
	Mycolicibacter kumamotonensis	WP_109560674	---------K-----T--H-AD-FTDDD---Y--T----A--	N	R-T----T--
	Mycolicibacter longobardus	WP_085263416	---------------TR-Y---S--EDD------N----PE-	D	-HT----Q-V
	Mycolicibacter minnesotensis	ORB03792	---------K-----S--H-A--FTEHD--HF--S---VP--	T	R------N--
	Mycolicibacter nonchromogenicus	WP_085138891	----------------T---L--Y-DDD--GT--Q----PD-	A	AHA----Q--
	Mycolicibacter senuensis	WP_085085262	---------------TR-H----NTDDD------N---TAE-	A	LHA----Q-V
	Mycolicibacter sinensis	WP_064856040	---------------TT---LR---ADD--GT--Q----PE-	A	AHA----Q--
	Mycolicibacter terrae	WP_085261407	---------------TT---LR-Y-DDD--ST--Q----PD-	A	AHA----Q--
	Mycolicibacter virginiensis	WP_105294465	---------------TR-Y----Y-DDD---S--N----PE-	D	-HA----Q-V
	Mycolicibacterium doricum	ORV44322	---------------S--HA-D-YT-D---T---R-T-PK--	E	RHE----A-V

Figure S16. Partial sequence alignment of a conserved region of the metal cation transporting ATPase H protein showing a one amino acid deletion that is specific for members of the "Tuberculosis" clade.

```
                                                                 162                                                          220
"Tuberculosis" clade (10/10)
Mycobacterium tuberculosis           AIH35166       IWSMSVQGQFYLAFLLLVAGCAYLLRRLF RGPR APYLRTMFVVLLSTLTLASFIYAIVA
Mycobacterium bovis                  PHO33000       ----------------------------- ---- --------------------------
Mycobacterium bovis BCG              AMC50386       ----------------------------- ---- --------------------------
Mycobacterium canettii               WP_015289972   --------------------------G-- ---- --------------------------
Mycobacterium caprae                 APU25655       ----------------------------- ---- --------------------------
Mycobacterium microti                AMC59181       ----------------------------- ---- --------------------------
Mycobacterium mungi                  OAQ18780       ----------------------------- ---- --------------------------
Mycobacterium orygis                 EMT36350       ----------------------------- ---- --------------------------
Mycobacterium pinnipedii             PRH92610       ----------------------------- ---- --------------------------
Mycobacterium africanum              WP_031670464   ----------------------------- ---- ---------A----------------
Other Mycobacteriaceae (2/30)
Mycobacterium shigaense              WP_096439561   -----------IG---M-------SQ--- -SRK --H---G-I---GA--V---T---A-
Mycobacterium triplex                CDO88416       -----------V-----I----V--QG-- ---- G-----L-----GV--V---V---F-
Mycobacterium asiaticum              WP_065034935   ---------------F----S-----AVL      GKH---F-----T---V---V---I-
Mycobacterium asiaticum              WP_065144193   -----------------IG-------P-G       KH---V------V--V---G---I-
Mycobacterium conceptionense         CQD14527       -----------------IS-F-F-G----      GRH---A-I----A--I---V---I-
Mycobacterium europaeum              WP_085238532   -----------V-----I------F---L      GAR---L-L---TA--V---V--V--
Mycobacterium florentinum            WP_085225617   -----------V---------TF-F--PL      KAH---L----I-A--V---V---F-
Mycobacterium genavense              WP_025737656   -----------V-----I----V-F--PL      KAH---L-----GA--V---V---F-
Mycobacterium gordonae               WP_055576247   ----------------VI-AV-----KPL      GRH---V------A--V---V---I-
Mycobacterium haemophilum            WP_047313904   -----------I------------F--PL      GTQ------A--GA--I---G---F-
Mycobacterium heidelbergense         WP_083074796   -----------IG------A----F--PL      GAH---L-L----A--V---V----V
Mycobacterium interjectum            WP_085204075   -----------I---V-------AW-GRR      -AH---V-L----A--V---V-----
Mycobacterium kansasii               WP_075542856   ---------------F---A---VF--PL      G-R---L------A--I---G---A-
Mycobacterium lacus                  WP_085160830   ------------------------F-VSL      -SH---T------A--T---G--VF-
Mycobacterium lentiflavum            CQD13241       -----------V----------V-F---L      KTR---L------A--I---V---F-
Mycobacterium liflandii              WP_015356016   -----------------IT--TF-F---L      GTH----------A--I---V---I-
Mycobacterium malmoense              WP_071509519   -----------VG----------AF--RL      STH-----M----A--V---V-----
Mycobacterium malmoense              WP_083011239   -----------VG----------AF--RL      STH-----M----A--V---V-----
Mycobacterium marinum                BBC65536       -----------------I---TF-F---L      GTH----------A--I---V---I-
Mycobacterium paraffinicum           WP_073877114   -----------VG----I-A----FG-PP      G-R---L-L---TA--V---V-----
Mycobacterium paraseoulense          WP_083171883   -----------VG----I------F--PL      GAR---L-L---TA--V---V-----
Mycobacterium pseudoshottsii J       GAQ31936       -----------------IT--TF-F---L      GTH----------A--I---V---I-
Mycobacterium scrofulaceum           WP_067277933   -----------VG---VI-A----FG-PL      G-R---L-L---TA--V---V-----
Mycobacterium shinjukuense           WP_083046018   ----------A-------TA-----K-PL      GDH---F------A--V--LC--V--
Mycobacterium szulgai                WP_085673397   -----------V---F-I------FK-SL      GKH---L------G------V--VI-
Mycobacterium ulcerans               WP_011739687   -----------------I---TF-F---L      GTH----------A--I---V---I-
Mycobacterium ulcerans str. Harvey   EUA90980       -----------------I---TF-F---L      GTH----------A--I---V---I-
Mycolicibacter longobardus           WP_085264210   ------------S----IF---F-F--PL      RAH---A-----TS--V---W-----
Mycolicibacterium conceptionense     WP_064899707   -----------------IS-F-F-G----      GRH---A-I----A--I---V---I-
Mycolicibacterium farcinogenes       WP_036387553   -----------------IS-F-F-G----      GRH---A-I----A--I---V---I-
```

Figure S17. Partial sequence alignment of a conserved region of an acyltransferase protein showing a four amino acid insertion that is specific for members of the "Tuberculosis" clade and absent from most other *Mycobacteriaceae*.

Group	Species	Accession	428		477
	Mycobacterium tuberculosis	AIH42673	WAALTAEKQRADAGSTLSFFRLALRLRRERNEFDG		DVDWLAAPDDALIFR
	Mycobacterium bovis	ANG88229	-----------------------------------		---------------
	Mycobacterium bovis BCG	AMC51469	-----------------------------------		---------------
	Mycobacterium bovis BCG str. A	AHM08225	-----------------------------------		---------------
	Mycobacterium canettii	WP_014001285	-----------------------------------		---------------
**"Tuberculosis" clade (11/11)**	*Mycobacterium caprae*	CEJ52987	-----------------------------------		---------------
	Mycobacterium microti	WP_105799835	-----------------------------------		---------------
	Mycobacterium mungi	WP_003899336	-----------------------------------		---------------
	Mycobacterium orygis	WP_003412692	-----------------------------------		---------------
	Mycobacterium pinnipedii	PRH91215	-----------------------------------		---------------
	Mycobacterium africanum	WP_080702412	-----------------------------------		---------------
	Mycobacterium asiaticum	OBI87239	-----V------T--A-A---EVI----A-A----	T	EI---SS-TGV----
	Mycobacterium avium complex	WP_042911199	-----VD--S--PD-------R--E--KR-V----	D	G----D-SG--V---
	Mycobacterium bohemicum	WP_085183024	-----V------P----H---RLIE--KA-S--A-	T	QIE--P--G------
	Mycobacterium branderi	WP_083131512	-----V---Q-------A---R--E---Q-E--G-	S	EIE--P--RG--M-K
	Mycobacterium celatum	WP_062538962	-T---V---L-----------R--E---Q-S--A-	G	EIE-----RG--M-K
	Mycobacterium chimaera	WP_087139676	-----VD--S--PD-------R--E--KR-V----	D	G----D-SG--V---
	Mycobacterium europaeum	WP_090420747	-----V-R-L--PE-------HVIK--K--T--E-	D	QI---D--R---V--
	Mycobacterium florentinum	WP_085220761	--P--V---D--PD---A---T--K-----A----	L	ELE--T-T--G-V--
	Mycobacterium gastri	WP_036418303	-----V------PD-------R--Q----HAQ---	S	QI---P--G--VV--
	Mycobacterium gordonae	WP_065133528	-----V------P--------T--E--GS-A----	E	GI---P---R--V--
	Mycobacterium haemophilum	WP_054879680	-----V----T--D------HQM-E-----A----	S	GIE--T--R------
	Mycobacterium heckeshornense	WP_048893034	---V-V---LR-PA-------R-VE---T-A----	D	RI---P--RE-----
	Mycobacterium intermedium	WP_069417363	-----V-R--K-PA-M-----R-IEI-KQ-A--Q-	G	-----S--E---L--
	Mycobacterium intracellulare M	ETZ32786	-----VD--S--PD-------R--E--KR-V----	D	G----D-SG--V---
	Mycobacterium intracellulare s	ARR82350	-----VD--S--PD-------R--E--KR-V----	D	G----D-SG--V---
	Mycobacterium kansasii	WP_023365920	-----V------PD-------R--Q----HDQ---	S	QI---P-TG---V--
	Mycobacterium kansasii 662	EUA19775	-----V------PD-------R--Q----HDQ---	S	QI---P-TG---V--
	Mycobacterium kansasii 732	EUA12856	-----V------PD--W----R--Q--K-HAQ---	N	QI---P--G--VV--
	Mycobacterium kansasii 824	EUA01255	-----V------PD-------R--Q----HDQ---	S	QI---P-TG---V--
**Other *Mycobacteriaceae* (0/39)**	*Mycobacterium kubicae*	WP_085074736	--G--V------PE-------RMIEI-SD------	T	QI------A-V----
	Mycobacterium lacus	WP_085162961	---W-V------PD-------R--E-----D--E-	T	EIE--G--RNT-V--
	Mycobacterium lentiflavum	WP_090604669	--S--V---D--PD---T---TV-K-----A----	V	ELE--T-T----V--
	Mycobacterium liflandii	WP_041299454	-----V---LE------A---R--E---K-R----	N	EIR--S--RG-----
	Mycobacterium marinum	WP_020726017	-----V---LEN-----A---R--E---K-R----	N	EIR--S--RG-----
	Mycobacterium microti	WP_101528565	--S--V---D--P----A---V--G---Q-S----	D	ELQ--S-SA-G-A--
	Mycobacterium nebraskense	WP_046184455	--T--V---I--PE--F----RVIK--K--T----	E	EI---D--R------
	Mycobacterium palustre	WP_085081031	-----VD-----PD-------R--Q-----T--E-	G	ELE-----R--V--
	Mycobacterium paraseoulense	WP_083174986	-----V-R-I--PE--F---QRVIK--K--T--E-	D	EI---D--R------
	Mycobacterium pseudoshottsii	WP_086085200	-----V---LE------A---R--E---K-R----	N	EIR--S--RG-----
	Mycobacterium saskatchewanense	WP_085254987	-----I---D-----------RIIQ-H---P----	G	EI------AG--V--
	Mycobacterium scrofulaceum	WP_067307130	-----V---V--PE--F---QHII---K--A----	E	ELE--D--R------
	Mycobacterium shinjukuense	WP_083046163	--G--V------G--------R--Q--SG-A----	D	-IE--T--RT-V---
	Mycobacterium simiae	WP_044507112	--S--V---D--P----A---V--G---Q-S----	D	ELQ--S-SA-G-A--
	Mycobacterium szulgai	WP_068032528	--G--V------PE------HRMIEI-SG-S----	T	QI------A-V----
	Mycobacterium ulcerans	WP_096371399	-----V---LEN-----A---R--E---K-R----	N	EIR--S--RG-----
	Mycobacterium ulcerans str. Harvey	EUA87399	-----V---LEN-----A---R--E---K-R----	N	EIR--S--RG-----
	Mycobacterium ulcerans subsp. shinshuense	BAV42573	-----V---LEN-----A---R--E---K-R----	N	EIR--S--RG-----
	Mycobacterium xenopi 3993	EUA18174	-----V---LS-PD----L--R-IE---T-V----	S	RIE---T-R------
	Mycobacterium xenopi 4042	EUA33162	-----V---LS-PD----L--R-IE---T-V----	S	RIE---T-R------

Figure S18. Partial sequence alignment of a conserved region of an alpha-amylase protein showing a one amino acid deletion that is specific for members of the "Tuberculosis" clade.

			257		306
	Mycobacterium tuberculosis	AIH43349	IVTGADVFALDPDGAVRYHSDGS	DGA	EHLVRGRFVLVGVTPAVLASLLGE
	Mycobacterium africanum	WP_105814941	--------------T--------	---	------------------------
	Mycobacterium africanum MAL020	KBI64038	--------------T--------	---	------------------------
	Mycobacterium bovis	WP_024457939	--------------T--------	---	------------------------
**"Tuberculosis" clade (9/9)**	*Mycobacterium bovis AF2122/97*	YP_009358254	--------------T--------	---	------------------------
	Mycobacterium bovis BCG	AMC49570	--------------T--------	---	------------------------
	Mycobacterium canettii	WP_014000490	--------------T--------	---	------------------------
	Mycobacterium microti	AMC58426	--------------T--------	---	------------------------
	Mycobacterium orygis	WP_003404667	--------------T--------	---	------------------------
	Mycobacterium alsense	WP_083139541	---A-----VN---Q---R-GDD		--V-------A--S-----G----
	Mycobacterium angelicum	WP_083114969	-T-D-----VS---E---RAAEA		--V---E---A--------G----
	Mycobacterium aquaticum	WP_083165026	-----E-Y-V----E---RK--A		AQR-EADV--AN---T---G----
	Mycobacterium arosiense	WP_083064178	-A---E-Y-V--T-E--FRAG-E		D-RITT----A----T---E----
	Mycobacterium asiaticum	OBI95472	---D----GIA---E-H-RNG-D		----------A--G-----N----
	Mycobacterium avium	WP_076220893	-L---E---V--S-E---R-GDD		--V--A----A----T---G----
	Mycobacterium bohemicum	ORV03997	-T-----Y-IN---V-H-RTGDA		-RTL-AG---A----T---D----
	Mycobacterium branderi	WP_083131341	-------Y-I----E---RCGDD		-CV-H-----A--A-----D----
	Mycobacterium celatum	WP_062541155	-------YVV----E---RRGDD		-CV-H--V-----A-----D----
	Mycobacterium chimaera	WP_089151362	-IS--E-Y----S-E-H-R-G-Q		-RR--A----A----T---G---D
	Mycobacterium colombiense	WP_064954377	-T-----C-V----E---R-G-E		D--I-A----A----T---G----
	Mycobacterium colombiense CECT	EJO86358	-T---E-Y-V--T-E---R-RDE		D--I-A----A----T---G----
	Mycobacterium europaeum	WP_085243260	-S---E-Y-IE---L---RTGET		-R-I-A----A--------Q----
	Mycobacterium florentinum	WP_085221906	-I---E-YGVE---E---RK--E		--PIQ---I-A----T---E----
	Mycobacterium fragae	WP_085194791	-T-------V--N-E---RHRDD		-FT-H-----A----S---E----
	Mycobacterium gastri	WP_036420249	-T---E---VE-T-E---RAG-D		--AI------S--------D----
	Mycobacterium gordonae	WP_065049841	-T-D-E-L----G-E---R-N-D		--R-------S--G-----G----
	Mycobacterium haemophilum	WP_054879403	-T-----Y-VN---S-H-RT-DE		---------I-A--------N----
	Mycobacterium hassiacum	WP_026213395	--CN-E-Y-VS---T-H-RRH-E		-RT-HADW--AN-------R---A
	Mycobacterium heckeshornense	WP_048890162	-L-----Y-I--E-E---RRG-E		-CR----VI-A----V---D---Q
	Mycobacterium heidelbergense	ORA70482	-I------GVE---L---R-G-D		---I------A-------SG----
	Mycobacterium houstonense	WP_066899489	-------Y-V----E-C-RTH-A		TRR--AGH--AN-------E---D
	Mycobacterium insubricum	WP_083031151	L----E-TGI------Y-RLGDD		-QR---DR----CA--E--R----
	Mycobacterium interjectum	WP_066915554	-II------VE---G---RCGEK		--E-------A----T---G---T
	Mycobacterium intermedium	WP_069421064	---------V----V---R-G-D		--AI------A--G-----R---D
	Mycobacterium intracellulare	WP_064892922	-IS--E-Y----S-E---R-G-H		-RR--A----A----T---G---D
	Mycobacterium iranicum	WP_064283698	-----E-Y-IH---E-H-RHSDV		--RIGA-H--AN-------R----
	Mycobacterium kansasii	WP_063472394	-T-------VA-T-E-H-RA--D		--VI------A--------G----
	Mycobacterium komaniense	WP_090274484	-TC----Y-I--T-E--FNC--A		-QT-H-DV--AN---T---G-M--
	Mycobacterium kubicae	WP_085075677	-T---E---IT-S-E-AFRR--R		Q-RA-S----C--S-T---G----
	Mycobacterium kyorinense	WP_045374789	-------Y-V----E---RRGDD		-CV-Q-----A--A-----D----
**Other *Mycobacteriaceae* (0/>100)**	*Mycobacterium lacus*	WP_085156158	-I-----Y-VA---E----TG-A		D-VI------A--------G----
	Mycobacterium lehmannii	WP_094288494	-TC--E-Y-V--E-E---RCG-V		DGT-Q-DVI-AN---T---G----
	Mycobacterium lentiflavum	WP_090606299	-----E-YGVE---Q---RQ--E		-RR-----I-A----T---E----
	Mycobacterium mageritense	WP_036433689	-------Y-I--E-E---RQG-T		A-RA-AGHI-AN---V---G----
	Mycobacterium malmoense	WP_065446260	-T-R-E-Y-V----R---RVRDD		-RTI-A----A----SA--G----
	Mycobacterium marinum	WP_094358470	-----EAV-VT-N-D---RAGDD		----------A--------E---D
	Mycobacterium marseillense	WP_083017974	-IC--E-Y-V--S-E---RIG-Q		--R--S----A----T--TG----
	Mycobacterium microti OV254	PLV54775	-T---E--HIQ---L-CFRR-DE		-QRL-A----A--------G----
	Mycobacterium montefiorense	WP_108924645	-T---E-YSVA---C---RR--E		--RIQA----A--------E----
	Mycobacterium moriokaense	WP_083149220	-AC----YHI----Q-C-RR--R		--R-NATH--AN---T---A--D-
	Mycobacterium nebraskense	WP_046184838	-I---E-Y-VE---V---RTGDK		-R-I-A----A----T---G----
	Mycobacterium neoaurum	CDQ45349	-------Y-IS---E---RTGEV		DN-----H--A-----T--R----
	Mycobacterium noviomagense	WP_083087668	-T-----Y-I----E-C-RVGDD		-R--T--HI-A----V---D----
	Mycobacterium novocastrense	WP_084377695	-TC-TE-Y-I--A-E----C--I		D-T-H--V--AN---T---G----
	Mycobacterium palustre	WP_085078629	-T---E---IE-G-R-C-RKA-E		-RAI------A----T---G----
	Mycobacterium paraense	WP_085093410	-L-------V--E-L---RCGDK		--A-------A----T---G----
	Mycobacterium paraffinicum	WP_073870628	-T---E-YVVE---L--FRARDE		-RII-A----A----T---G----
	Mycobacterium parafortuitum	WP_083144670	-R-----Y-IT---E-S-RDGDG		DQR-AADR--AN-------R----
	Mycobacterium parascrofulaceum	EFG75349	-T-R-E-Y-V----G---RVRDD		-RTL-A----A----SA--G----
	Mycobacterium paraseoulense	WP_083167454	-H--T--Y-VE---V---RAGNE		---L-A----A----SA--G----
	Mycobacterium parmense	WP_085270143	-L-----YRI----R-LFRTGEQ		--V--A-Y--A--------A----
	Mycobacterium peregrinum	WP_064936132	-I-----Y-VS---E---RQG--		ARR-HAER--AN-------E----
	Mycobacterium phlei	WP_040635217	-TC------I----E--FRHN-D		--V-H-DV--AN-------E----
	Mycobacterium rhodesiae	WP_014211052	-LC----Y-I--G-Q---RRH-R		D---QAQH--AN---T---A----
	Mycobacterium rutilum	WP_083410474	--CN-E-Y-VN-------RK--A		--T-H-DV--AN---S---E-M--
	Mycobacterium saskatchewanense	WP_085256085	-T-D-E-YVVQ---L--FRLGDD		--A-------SA---T---A----
	Mycobacterium scrofulaceum	WP_067274067	-T---E-Y-VE---V--FRARDE		---I-A----A--------G----
	Mycobacterium septicum	WP_044520285	-I-----Y-I----E---RRG--		ARR-AAER--AN-------D----
	Mycobacterium setense	WP_039324873	-I-----Y-VN---E---RQG--		ARR-NADR---N----A--G---D
	Mycobacterium sherrisii	WP_069398415	-----E--RIE---S-CFRR--E		--RL-A----A--------A----
	Mycobacterium shigaense	WP_096442556	-I---E-Y-V----R-D-RC-DD		-RVS------A-----A--E----
	Mycobacterium shimoidei	WP_069396757	-I---A-Y-VN---E---RHGDD		-CV-------A----T---G----
	Mycobacterium shinjukuense	WP_083050187	-T-------VN---E---R-R-G		---I------A----T---G----

**Other *Mycobacteriaceae* (0/>100)**	*Mycobacterium simiae*	WP_061557016	-T---E--RIQ---L-CFRR-DE	-QRL-A----A--------G----
	Mycobacterium smegmatis	WP_058126994	-----E-Y-IT---E---RHG-T	A-RISAHR--AN---V---K----
	Mycobacterium szulgai	WP_085670572	-T-D-----VS-----H-RDV-A	--V---E---A--------G----
	Mycobacterium timonense	WP_083186799	-L---E---V--S-E---R--	--VA-A----A----T---G----
	Mycobacterium triplex	WP_051641489	-----E-Y-V--T-E---RR--D	--VS------S----T---R----
	Mycobacterium tusciae	WP_083127828	-ICD-E-Y-IN---E---RR--R	D-R-HAGHI-SN---T---T----
	Mycobacterium ulcerans	WP_011738617	-----EAV-VT-N-D---RAGDD	----------A--------K---D
	Mycobacterium vaccae	WP_003930174	-I---E-Y-IT---E---RHGDA	--V-A-GH--AN-------R----
	Mycobacterium vulneris	WP_065458396	-I-----Y-I----E---RQA-A	SRR-AADRI-AN-------E----
	Mycobacterium wolinskyi	WP_085144855	-I---E-HGI----Q---RHN-K	A-QLSADHI-AN-------D----
	Mycobacterium xenopi	WP_085194644	-------Y-V--E-E-W-RCG-E	-CRI---VI-A----V---D---Q
	Mycobacteroides abscessus	WP_062878657	LLS--E-LSIS--AE---RR--D	--T-A-DR--S--S-T---G----
	Mycobacteroides chelonae	WP_070918468	LR---E-LSIN-E-E---RAGDD	--TAI-ER--S----T---R----
	Mycobacteroides franklinii	WP_078334129	LR---E-LSIS---E---GTG-E	--T-A-GR--S--S-----N----
	Mycobacteroides immunogenum	WP_064628379	LR---E-LSVS---E---RTG-G	--T-A-AR--S--S-T---G----
	Mycobacteroides salmoniphilum	WP_078330740	LR---E-LTVS---E-C-RIGDD	--IAI-ER--S--------H----
	Mycobacteroides saopaulense	WP_070913575	LR---E-LSIN---E-K-RAV-E	--T-V-ER--S--S-T---R----
	Mycolicibacterium agri	WP_097942466	-T-N-E-YGI----E---RL--D	--R-HAGY-MA--------D-V-D
	Mycolicibacterium aurum	WP_087027099	-----E-Y-IS---D---RTRDG	--TMH-TH--A--S-----R----
	Mycolicibacterium austroafricanum	WP_036371272	-------Y-I--E-Q---RCADD	--V-AA-HI-AN-------R----
	Mycolicibacterium bacteremicum	ORA04928	-I---E-Y-IT---E---RTGDV	--V-H--H--A--------R----
	Mycolicibacterium boenickei	WP_077740490	-I-----Y-V----E---RQA--	ARC-AADRI-AN-------D----
	Mycolicibacterium brisbanense	WP_062827597	-I-----Y-VN---E---RK--A	AQR-E-DV--AN---T---G----
	Mycolicibacterium brumae	WP_090589335	L--E-E-LSI----S---LH--A	--Q---KKI-A-CS--E--R----
	Mycolicibacterium canariasense	WP_109762334	-S---E---IT---E-H-RHG-A	--V-S-AV--A----T---G----
	Mycolicibacterium confluentis	WP_085155349	-----E---IT---E---RQ--R	--V-AAGH--AN-------Q-V--
	Mycolicibacterium cosmeticum	WP_036397374	-S---E---IT---E-H-REG-T	-RV-S-AV--A----T---G----
	Mycolicibacterium diernhoferi	WP_073858974	-I---E-Y-IS---D---RTQDG	--T-H--H--A--------R----
	Mycolicibacterium elephantis	WP_084730306	-IC--E---I----Q---RHN-D	--R-HADV--AN-------R----
	Mycolicibacterium flavescens	WP_084294203	--C----Y-V----Q---RK--A	-QT-H-EV--AN---T---G----
	Mycolicibacterium fortuitum	WP_061265390	-I-----Y-VS---E---RHG--	TRR-TAER--AN-------D----
	Mycolicibacterium goodii	WP_100516424	-----E-Y-IT---E---RHG-T	ARRIGA-H--AN-------K----

Figure S19. Partial sequence alignment of a conserved region of the hypothetical protein IQ48_14915 showing a three amino acid insertion that is specific for members of the “Tuberculosis” clade.

Group	Species	Accession	422		469
“Tuberculosis” clade (9/9)	*Mycobacterium tuberculosis*	AUS50012	GGRISGRYALIRTNGDRWLAHRLK	NQK	DQKVFEFDNLAPMLATHGTVA
	Mycobacterium bovis	AMC54246	------------------------	---	---------------------
	Mycobacterium canettii	WP_044096082	------------------------	---	---------------------
	Mycobacterium caprae	WP_075744483	------------------------	---	---------------------
	Mycobacterium microti	PRI06705	------------------------	---	---------------------
	Mycobacterium mungi	OAQ18148	------------------------	---	---------------------
	Mycobacterium orygis	WP_003404798	------------------------	---	---------------------
	Mycobacterium pinnipedii	PRH89837	------------------------	---	---------------------
	Mycobacterium africanum	CCC26019	------------------------	---	---------------------
Other *Mycobacteriaceae* (2/37)	*Mycobacterium angelicum*	ORA06167	------------------------	---	---------------------
	Mycobacterium peregrinum	OWL92313	------------------------	---	---------------------
	Mycobacterium alsense	WP_083139897	-N---------Q----Q-----M-		--------E----F----S--
	Mycobacterium arosiense	WP_083064108	-SK--------Q----Q-----M-		--------AV--------S-T
	Mycobacterium asiaticum	WP_065141285	-S---------Q----Q-----M-		--N--D--A-T--F----S--
	Mycobacterium avium	WP_003872849	-RK--------Q-Q--Q-----M-		--N-----TI--------S-S
	Mycobacterium avium 10-5560	ETB50965	-RK--------Q-Q--Q-----M-		--N--D--TI--------S-S
	Mycobacterium avium MAV_061107	ETZ46540	-RK--------Q-Q--Q-----M-		--N--D--TI--------S-S
	Mycobacterium avium subsp. avium	EUA36129	-RK--------Q-E--Q-----M-		--N--D--TI--------S-S
	Mycobacterium avium subsp. hominissuis	BAN29981	-RK--------Q-Q--Q-----M-		--N--D--TI--------S-S
	Mycobacterium avium subsp. paratuberculosis	ETA99855	-RK--------Q-Q--Q-----M-		--N-----TI--------S-S
	Mycobacterium bohemicum	WP_085183503	-S--------------Q-----M-		--N--D--EIR----S--S-S
	Mycobacterium chimaera	WP_089151376	-SK--------Q----Q-----M-		--------TI--------S-T
	Mycobacterium colombiense	WP_007777035	-S---------Q----Q-----M-		-----D--TI--------S-T
	Mycobacterium florentinum	WP_085219845	-S---------Q----Q-----M-		--------EVT-------S--
	Mycobacterium fragae	WP_085194886	-K---------Q----Q-----M-		-----D--G-------E-S--
	Mycobacterium genavense	WP_036467825	-S---------Q----Q-----M-		--------EV--------S--
	Mycobacterium gordonae	WP_055581154	-N---------Q----Q-----M-		-----D--G-----T---S--
	Mycobacterium intracellulare	WP_014379267	-SK--------Q----Q-----M-		--------TI--------S-T
	Mycobacterium intracellulare 1	EUA57633	-SK--------Q----Q-----M-		--------TI--------S-T
	Mycobacterium kansasii	WP_063472356	-N---------Q-D--Q-----M-		--Q--D--S-----T---S--
	Mycobacterium kubicae	WP_085073036	-N---------Q-K-NQ-----M-		--------S----F-S--S--
	Mycobacterium lacus	WP_085161692	-S---------Q----H-----M-		-------GA-----C---S--
	Mycobacterium lentiflavum	WP_090606186	-S---------Q-K--Q-----M-		--------EV--------S--
	Mycobacterium mantenii	WP_083096821	-SKV-------Q----Q-----M-		--------TI--------S-T
	Mycobacterium marinum	WP_020729647	-S---------Q----Q-----M-		--T--D--T-----TS--S--
	Mycobacterium marinum MB2	EPQ78271	-S---------Q----Q-----M-		--T--D--T-----TS--S--
	Mycobacterium marseillense	WP_083018018	-AK--------Q----Q-----M-		--------TI--------S-T
	Mycobacterium montefiorense	WP_108925544	-S---------Q----Q---R-M-		--------EI--------S--
	Mycobacterium paraense	WP_085098847	-N---------Q----Q-----M-		-----D-GS----M--E---T
	Mycobacterium riyadhense	WP_085253074	-N---------Q-D--Q--V--M-		--------T---------S--
	Mycobacterium sherrisii	WP_069398486	-S--------------Q-----M-		--H--D--EI---F----S-T
	Mycobacterium shinjukuense	WP_083050335	-S-----------D--------M-		--Q-----S---------S--
	Mycobacterium szulgai	WP_068028769	-N---------Q-K-NQ-----M-		--------S----F-S--S--
	Mycobacterium triplex	WP_036473025	-S---------Q-S--Q-----M-		--------EV--------S--
	Mycobacterium ulcerans str. Harvey	EUA86423	-S---------Q----Q-----M-		--TA-D--T-----TS--S--
	Mycobacterium vulneris	WP_085289747	--K--------Q----Q-----M-		-----D--TI--------S-T

Figure S20. Partial sequence alignment of a conserved region of the hypothetical protein CAB90_01059 showing a three amino acid insertion that is specific for members of the “Tuberculosis” clade and is absent from most other *Mycobacteriaceae*.

Group	Species	Accession	406		457
"Tuberculosis" clade (9/9)	*Mycobacterium tuberculosis*	AIH41577	VAGLAEVNPARVEVDRVLESAERHPIL		GQVTSLAEARTTVLLDEIVTLVGTD
	Mycobacterium bovis	ESK76721	---------------------------		-------------------------
	Mycobacterium canettii	WP_014000651	---------------------------		-------------------------
	Mycobacterium caprae	CEJ50977	---------------------------		-------------------------
	Mycobacterium microti	WP_105799522	---------------------------		-------------------------
	Mycobacterium mungi	OAQ17758	---------------------------		-------------------------
	Mycobacterium orygis	EMT36547	---------------------------		-------------------------
	Mycobacterium pinnipedii	PRH92776	---------------------------		-------------------------
	Mycobacterium africanum	WP_080701602	---------------------------		-------------------------
Other *Mycobacteriaceae* (2/38)	*Mycobacterium alsense*	WP_083139834	-P-P-QFAAV-A------D------V-		-R-IT-D----------------A-
	Mycobacterium sphagni	WP_094478528	I---GA-AG--R-I---FD--Q----F		----T---------------M-AA-
	Mycobacterium angelicum	WP_083116116	I----G-GA--I------D--A----S	V	-------------------------
	Mycobacterium arosiense	WP_083065078	I----G-AA---------D--------	F	---------------------I-G-
	Mycobacterium asiaticum	WP_065144441	-T---A-AA--L------D--G---VA	I	-------------------A--SA-
	Mycobacterium avium	WP_003875481	-P--SA-AA--A------D------LS	F	-----------------------R-
	Mycobacterium avium subsp. hominissuis	PBJ38167	-P--SA-AA--A------D------LS	F	-----------------------R-
	Mycobacterium bohemicum	WP_085180361	-P---G-AT--A------D------LS	I	EP-----------------S---A-
	Mycobacterium chimaera	WP_089151443	----TGAAA--I------D-------S	F	-----------A--V------I-G-
	Mycobacterium colombiense	WP_044487032	-----GIAA--I------D-------S	F	-----------------------G-
	Mycobacterium florentinum	WP_085221266	-----GIAT--S------D-------S	I	--------------------TI---
	Mycobacterium genavense	WP_025735304	-----GIAE--S------D------VS	I	--------------------TI---
	Mycobacterium gordonae	WP_055577966	-H---A-AA--A------D------VS	F	-R-------------------I-A-
	Mycobacterium haemophilum	WP_054880891	I---SG-AA---------D------VS	I	D----V------------I--I---
	Mycobacterium interjectum	WP_066914857	-------AA--T------D------VS	I	----T---------------V--A-
	Mycobacterium kansasii	WP_036400803	----RGIAA--T---G--D--V----S	I	-----V-----A------------H
	Mycobacterium lacus	WP_085162108	-------AA--T------D------VS	I	-E---------------------A-
	Mycobacterium lentiflavum	CQD19472	-----G-AA--S------D------VS	I	--------------------TI-A-
	Mycobacterium lepraemurium	ATA27984	-P--SG-AA--A------D------LS	F	-----------------------R-
	Mycobacterium liflandii 128FXT	AGC63997	----DGLAS---------D-------S	I	-R---------------------N-
	Mycobacterium marinum	WP_103653964	----DGLAS--I------D-------S	I	-R---------------------N-
	Mycobacterium montefiorense	WP_108921694	-----G-AA--S------D-------S	I	-----------------------S-
	Mycobacterium palustre	WP_085076186	L---T--AA--S------D------LS	I	-------------------A--RA-
	Mycobacterium paraense	WP_085096875	-------AV--A------D------VA	I	-R--T------------------A-
	Mycobacterium paraffinicum	WP_073880537	-----GIA---A-L----D--A----S	I	P--------------------I-RE
	Mycobacterium parmense	WP_085269300	-----G-AD--A------D--G---VS	I	-E---------------------S-
	Mycobacterium pseudoshottsii	WP_086085855	----DGLAS---------D-------S	I	-R---------------------N-
	Mycobacterium sherrisii	WP_069399301	----TG-AA--A------D--A---VS	I	-R-----------------G-I-S-
	Mycobacterium shigaense	WP_096442011	-----GIAA--G------D-------S	L	------------------------H
	Mycobacterium shinjukuense	WP_083050757	----SA-ES--I------D------SS	F	-R----D-------------S-AA-
	Mycobacterium simiae	WP_061559784	AD---G-AA--A------D--A---VS	I	-R-------------------I-S-
	Mycobacterium szulgai	WP_085673230	I----G-AA--S------D--A---VS	I	-------------------A---S-
	Mycobacterium timonense	WP_083187408	-P--SG-AA--A------D------LS	F	-----------------------R-
	Mycobacterium triplex	CDO90089	-----GIAE--N------D--Q---VS	I	--------------------TI---
	Mycobacterium ulcerans	WP_011739179	----DGLAS---------D-------S	I	-R---------------------N-
	Mycobacterium ulcerans str. Harvey	EUA91996	----DGLAS---------D-------S	I	-R---------------------N-
	Mycobacterium vulneris	WP_085292389	-----GIAA--I------D-------S	F	-----------------------GH
	Mycobacterium xenopi	WP_003921962	----G-AAA--A------DT-----HV	V	-----V-------------G--SA-

Figure S21. Partial sequence alignment of a conserved region of the transcriptional regulator protein showing a one amino acid deletion that is specific for members of the "Tuberculosis" clade and is absent from most other *Mycobacteriaceae*.

Group	Species	Accession	10		59
"Tuberculosis" clade (9/9)	*Mycobacterium tuberculosis*	AIH59242	TPRERLTRGLAYSAVGPVDVTRGL	LELG	VGLGLQSARSTAAGLRRRYREG
	Mycobacterium bovis	PHP04760	------------------------	----	----------------------
	Mycobacterium canettii	WP_015288696	-----------T------------	----	----------------------
	Mycobacterium caprae	WP_003400307	------------------------	----	----------------------
	Mycobacterium microti	AMC57439	------------------------	----	----------------------
	Mycobacterium mungi	OAQ18847	------------------------	----	----------------------
	Mycobacterium orygis	EMT37776	------------------------	----	----------------------
	Mycobacterium pinnipedii	PRH91326	------------------------	----	----------------------
	Mycobacterium africanum	WP_031669836	----------------------S-	----	----------------------
Other *Mycobacteriaceae* (0/65)	*Mycobacterium alsense*	WP_083140941	--S---A---T--T----------		--------Q---S---Q-----
	Mycobacterium angelicum	WP_083112583	----------T------------V		---SV---H---SE--------
	Mycobacterium arosiense	WP_083064829	---Q--A---S---Q----L---V		---SV---Q----R-----Q--
	Mycobacterium asiaticum	WP_036362279	----------T------------V		----V---H---TE--------
	Mycobacterium avium	WP_003872095	------A---T--TL--L-L---V		A--SV-------EQ--------
	Mycobacterium bohemicum	WP_085179791	---Q--A---T--T---------V		----AK--Q-A--Q-----Q--
	Mycobacterium branderi	WP_083132206	---Q--------T----------V		----VH--QAG-SN-----Q--
	Mycobacterium celatum	WP_062539884	---Q--------T-A---------		----VH--QAG-SS-----Q--
	Mycobacterium chimaera	WP_089151285	------A---T--TR--------V		---SV---Q---Q---------
	Mycobacterium colombiense	WP_007771619	---Q--A---S---Q--------V		---SV---Q---SQ-----Q--
	Mycobacterium conspicuum	WP_085233853	------S---T------------V		----VH-VQ---SQ-----QQ-
	Mycobacterium europaeum	WP_085239298	------A---T--TL--------V		----V---QA--SQV--K-Q--
	Mycobacterium florentinum	WP_085219492	---Q--S---T---L--------V		I---AH-VQ---SE------Q-
	Mycobacterium fragae	WP_085199183	---Q--S-----T-----------		----VD--QAG--N--------
	Mycobacterium gastri	WP_036418468	----------T------------V		----V---H---SE--------
	Mycobacterium genavense	WP_025738159	---Q--S----------------V		I---ADTVQA---E------Q-
	Mycobacterium gordonae	WP_055578116	----------T------------V		----V---H----EV-------
	Mycobacterium grossiae	WP_070355117	---Q--A---T------L-----A		----V---G-S--W--D---S-
	Mycobacterium haemophilum	WP_047315666	----------T---L--------V		----VH------S-------H-
	Mycobacterium heckeshornense	WP_048891393	---Q--------T------I---V		----AH--QVG--N-L------
	Mycobacterium heidelbergense	WP_083075359	---Q--A---T------------V		----VHF-Q---SQ-H------
	Mycobacterium interjectum	WP_085201846	------A---T-T------L---V		---SVNG-Q-A-SE--------
	Mycobacterium intermedium	WP_069422155	----------T-T----------V		-----N--Q-A-SE---H----
	Mycobacterium intracellulare	WP_036457838	S-----A---T---R--------V		---SV---Q-S--Q-----Q--
	Mycobacterium kansasii	OOK75890	----------T------------V		----V---Q---SEV-------
	Mycobacterium kubicae	WP_085073260	----------T------------V		----VH--Q---SE-----Q--
	Mycobacterium kyorinense	WP_065014543	---Q--N---T-------------		-E--VH--QAG-SS-----Q--
	Mycobacterium lacus	WP_085163121	----------T-T----------V		----V---Q---SE--------
	Mycobacterium lentiflavum	WP_090597996	---Q--S-------I-----A--A		I---AH-VQ---SE-H------
	Mycobacterium lepraemurium	ATA27160	------A---T--TL--L-L---V		A--SV-------EQ--------
	Mycobacterium liflandii	WP_015353887	----------T-T----------V		----VH--Q---SE-----PQ-
	Mycobacterium malmoense	WP_065444773	------A---T--T---------V		----V---Q---SQI--K-Q--
	Mycobacterium mantenii	WP_083095116	---Q------T---Q--------V		----V---Q---SQ--S--E--
	Mycobacterium marinum	WP_012392018	----------THT----------V		----VH--Q---SE------Q-
	Mycobacterium marseillense	WP_083015749	------A---T---R----L---V		---SVE--Q----E-----Q--
	Mycobacterium montefiorense	WP_108926472	---Q--S---T-------------		I---VN-TQ---SE------Q-
	Mycobacterium nebraskense	KKC05547	------A---T--TL--------V		----V---Q---SQI--K-Q--
	Mycobacterium noviomagense	WP_083088021	---Q------T-T----------V		-----H--A-G--NV-------
	Mycobacterium palustre	WP_085081011	---A-------NTVW--------A		L---V---QA--SE--------
	Mycobacterium paraense	WP_085243879	------A---T-T----------V		---SVNG-Q---LE--------
	Mycobacterium paraffinicum	WP_073878918	S-----A---T--TL-----A--V		---SA---Q----QI--K-Q--
	Mycobacterium paraintracellulare	WP_041784728	------A---T---R--------V		---SV---Q---Q---------
	Mycobacterium paraseoulense	WP_083171200	----------T--TL--------A		----V---Q----QI--K-Q--
	Mycobacterium parmense	WP_085270406	S-----A----HT-A------W--		I---AN--Q-AGSQ------Q-
	Mycobacterium persicum	WP_083154539	----------T--M---------V		----V---Q---SE--------
	Mycobacterium riyadhense	WP_085252991	----------T--------I---V		----V---H---SQ--------
	Mycobacterium saskatchewanense	WP_085254176	---Q--V----------------M		----V---Q---SE-----Q--
	Mycobacterium scrofulaceum	WP_067279752	S-----A---T--T------A--V		----A---Q---SQI--K-Q--
	Mycobacterium sherrisii	WP_069401007	---Q--S---T---L---------		----VD--Q---SQ------Q-
	Mycobacterium shigaense	WP_096436023	------A---T--T---------V		-E--VN-------Q--D--ER-
	Mycobacterium shimoidei	WP_069394304	---Q--S-----T----------V		----V---QAG-SS--------
	Mycobacterium shinjukuense	WP_083049392	----------T------------V		----V----A--SQ--------
	Mycobacterium smegmatis	WP_036452144	--Q------K-T------I---V		L-I-ADT-QA---E-----ES-
	Mycobacterium szulgai	WP_085672027	----------T------------V		---SV---H---SE--------
	Mycobacterium talmoniae	WP_071028768	---Q--S---T-T-I--------V		----AH---AG--S-----K--
	Mycobacterium triplex	WP_036465571	---Q--S----------------V		I---ADTVQA---E------R-
	Mycobacterium ulcerans	WP_011738415	----------T-T----------V		----VH--Q---SE-----PQ-
	Mycobacterium vulneris	WP_085288762	---Q--A---T---Q--------V		---SV---Q---SK-----Q--
	Mycobacterium wolinskyi	WP_067849849	-HQ--S---K-T------I---V		-----N--QA--N------ES-
	Mycobacterium xenopi	WP_003919828	---Q------T-T----------V		-----HT-QAG--N----H---
	Mycolicibacillus koreensis	WP_085304210	---Q--------T-L--------V		-----HT--AG-T------Q--
	Mycolicibacillus trivialis	WP_069391643	---Q--------T-L--------V		-----HT--AG---V----QQ-
	Mycolicibacterium doricum	WP_085192523	-S-Q--A---K-T--------L-V		L-V-AD-TKA---E------S-

Figure S22. Partial sequence alignment of a conserved region of the hypothetical protein IU12_21070 showing a four amino acid insertion that is specific for members of the "Tuberculosis" clade.

Clade	Species	Accession	194		250
“Tuberculosis” clade (8/8)	Mycobacterium tuberculosis	AIH80201	IAGLSVELGIATQRHDGLPKIVHALATAA		GNGAAAEEVDLLRVHVDTALHHVLAQYP
	Mycobacterium africanum	AMC61790	------------------------M----		----------------------------
	Mycobacterium bovis	PRI08661	------------------------M----		----------------------------
	Mycobacterium canettii	WP_044095667	------Q-------R----R---------		----------------------------
	Mycobacterium microti	PRI02693	------------------------M----		----------------------------
	Mycobacterium mungi	OAQ18826	------------------------M----		----------------------------
	Mycobacterium orygis	EMT37799	------------------------M----		----------------------------
	Mycobacterium pinnipedii	PRH91345	------------------------M----		----------------------------
Other *Mycobacteriaceae* (0/36)	Mycobacterium alsense	WP_083136449	VDDIGW--SQ--HWR----RM--T--R--	AA	-T-V-Q--I------L---RYQL-I-H-
	Mycobacterium angelicum	WP_083112668	VED-GW--AT--HLR----NM---M-KV-	AA	-T-VVE---------L---RYQL-----
	Mycobacterium asiaticum	WP_051545711	VDD-DW---A--HWRE---RLA-T--K--	AG	-GEIVEQ-A------L--LRYQ------
	Mycobacterium avium	WP_042792140	-DD-ARV-SQ--RA-AE-----TR--D-T	AA	-TFVVDQ---V----L---RYQL-V---
	Mycobacterium avium subsp. avium	EUA29469	-DD-ARV-SQ--RA-AE-----TR--D-T	AA	-TFVVDQ---V----L---RYQL-V---
	Mycobacterium chimaera	ASL12517	-DD-ARV-SQ--RA-AE-----TR--D-T	AA	-TFVVDQ---V----L---RYQL-V---
	Mycobacterium colombiense	WP_064883179	-DD-AGV-SA--R-R-D-----PR--E--	AA	-TVVVDQ---V----L---RYQL-V---
	Mycobacterium gastri	WP_051508061	LDN-LS--SG--RWR----R---L--Q-S	AA	EG-VGED-I------L---R-E-V----
	Mycobacterium gastri 'Wayne'	ETW23557	LDN-LS--SG--RWR----R---L--Q-S	AA	EG-VGED-I------L---R-E-V----
	Mycobacterium gordonae	WP_065047705	VED-DW--RV--HGRE---RL-N-M-K-V	AS	-ADIVEQ--------L--VRYQ------
	Mycobacterium interjectum	WP_085201823	VDD-GW--SQ--HWR----RM--T--R--	AA	-T-V-E--I------L---RYQL-I---
	Mycobacterium intracellulare	WP_014378760	-DD-ARV-SQ--RA-AE-----TR--D-T	AA	-TFVVDQ---V----L---RYQL-V---
	Mycobacterium intracellulare 1	EUA54093	-DD-ARV-SQ--RA-AE-----TR--D-T	AA	-TFVVDQ---V----L---RYQL-V---
	Mycobacterium intracellulare s	AFS12022	-DD-ARV-SQ--RA-AE-----TR--D-T	AA	-TFVVDQ---V----L---RYQL-V---
	Mycobacterium kansasii	ORB90236	LDD-VS--SG--RWR----R---L-TQ--	AA	-G-VGED-I------L---R-K-V----
	Mycobacterium kansasii 732	EUA08047	LDH-LP--SG--RWR----R---L--Q--	AA	-G-VGED-I------L---R-E-V----
	Mycobacterium kansasii ATCC 12	AGZ51438	SDN-AS--SGV-RWR----R---L--Q--	AA	-G-VGED-I------L---R-A-V----
	Mycobacterium liflandii	WP_015353915	ADNVAR--CD--RSRV---RMA-V--K--	DA	-TAVVED-I------L---R-QIV-H--
	Mycobacterium litorale	WP_078021462	VED-GWK-AE--HWR----RM--T--K-G	AA	RT-VVDA---V----L---RYQL-----
	Mycobacterium mantenii	WP_083095136	-ED-ASV-SA--CGR-D-----PR--Q--	AA	-TFVVDQ---V----L---RYQL-V---
	Mycobacterium marinum	WP_020727579	ADNVAR--CD--RSRV---RMA-V--K--	DA	-TAVVED-I------L---R-E-M-H--
	Mycobacterium paraense	WP_085243871	VND-GW--SQ--HWR----RM--T--R--	AA	-T-V-E--I------L---RYQL-I---
	Mycobacterium paraffinicum	WP_073879003	-E--GEV-SQT-R-F----R--QR--D--	AT	EAVIIDQ---V----L---RYQL-V---
	Mycobacterium paraintracellulare	WP_085318484	-DD-ARV-SQ--RA-AE-----TR--D-T	AA	-TFVVDQ---V----L---RYQL-V---
	Mycobacterium parmense	WP_085270426	-DN-RSV--ET-HWR----R--YR--N--	AT	-ETVVDQ-I-V----L---RYQL-V---
	Mycobacterium persicum	WP_083154497	LDD-VS--SG--RWR----R---L-TQ--	AA	-G-VGED-I------L---R-E-V----
	Mycobacterium rhodesiae	WP_005142017	VDDMGWK-VE--HWR----RM--T--K-G	VA	RT-VLDA---V----S---RYQLM----
	Mycobacterium riyadhense	WP_085253014	VKD-GR---N--TTR----RM--T-----	AA	-TAVVD--LE-----LE--RYQ------
	Mycobacterium shinjukuense	WP_083052343	VDD-GGR-SR--H-R----RLA-TI-G--	TA	-AVVTGD-A------L---RSQ------
	Mycobacterium sphagni	WP_094483721	VDE-GWK-VE--HWR----RM--T--K-G	TA	RT-VLDA---V----S---RYQLM----
	Mycobacterium szulgai	ORW93167	VE--GQ---A--H-R-----M--TM-KV-	AA	-T-VVE---------L--SRYQL-----
	Mycobacterium vulneris	WP_085288741	-DD-ASV-SAT-R-R-D-----PR--E--	AA	-TVVVDQ---V----L---RYQL-V---
	Mycolicibacter arupensis	WP_046190023	VRD-GWQ-SE--HWRE---RM-NT--K-G	SA	-T-VVDA-I------L--LRYQL-----
	Mycolicibacter minnesotensis	WP_083026308	VRD-GWQ-SE--HWRE---RM-NT--K-G	SA	-T-VVDA-I------L--LRYQL-----
	Mycolicibacter sinensis	WP_064857054	-DD-GWQ-SE--HWR----R-ANT--K-G	SA	-T-VVDA-I------L--MRYQL-----
	Mycolicibacterium aromaticivorans	WP_036342046	VHD-GWK-VE--HWR----R---T--K-G	AA	RT-VLDA---V----S---RYQLM----

Figure S23. Partial sequence alignment of a conserved region of the hypothetical protein IU14_19860 showing a two amino acid deletion that is specific for members of the “Tuberculosis” clade.

Group	Species	Accession	470		522
“Tuberculosis” clade (10/10)	*Mycobacterium tuberculosis*	AIH76484	LLRDIAVMVLCGLVVWQIYRPGRDLVRTG	GPGALPAC	GGVDDPVGGVFANAAD
	Mycobacterium bovis	WP_031702925	-----------------------------	--------	----------------
	Mycobacterium bovis BCG	AMC48512	-----------------------------	--------	----------------
	Mycobacterium canettii	WP_015288736	-----------------------------	--E-P---	----------------
	Mycobacterium caprae	WP_075744433	-----------------------------	--------	----------------
	Mycobacterium microti	AMC57487	-----------------------------	--------	----------------
	Mycobacterium mungi	OAQ17309	-----------------------------	--------	----------------
	Mycobacterium orygis	EMT37820	-----------------------------	--------	----------------
	Mycobacterium pinnipedii	PRH91363	-----------------------------	--------	----------------
	Mycobacterium africanum	WP_031668153	-----------------------------	--------	----------------
Other *Mycobacteriaceae* (0/40)	*Mycobacterium aquaticum*	WP_083164280	--------G--A--IR-----EL----H-		-RI---T----DG-P-
	Mycobacterium aurum	WP_048631588	-------V----F-IR-----HL----Q-		-----A----DR-P-
	Mycobacterium boenickei	WP_077739677	----L--I---A--IR-----EL----H-		-RI---T----DR-P-
	Mycobacterium brisbanense	WP_062828868	--------G--A--IR-----EL----H-		-RI---I----DG-P-
	Mycobacterium colombiense	WP_007771712	-------I---A--IR------E----W-		-R----A--P-DR-P-
	Mycobacterium conceptionense	CQD03028	-I--L--I---A-IIR-----EL----H-		-RI---T----DG-P-
	Mycobacterium dioxanotrophicus	ART73306	--------A--A--IR-----EL----H-		-RI---T----DG-P-
	Mycobacterium doricum	WP_085192539	----V--IT--A--IR-----EL----Y-		-R----T----DR-S-
	Mycobacterium elephantis	WP_046752337	-------IA--A--IR-----EL----NR		-D----A----DR-P-
	Mycobacterium engbaekii	WP_085128976	-V--L--L---A--IR-----QQ----AH		-WA---A--S-DG---
	Mycobacterium fallax	WP_085093152	-------LG--V---R------Q----W-		-R----A----DR-P-
	Mycobacterium fortuitum	OBG53383	-------I---A-IIR-----EL----H-		-RI---T----DR-P-
	Mycobacterium fortuitum subsp. fortuitum	EJZ13948	-------I---A-IIR-----EL----H-		-RI---T----DR-P-
	Mycobacterium fragae	WP_085199226	-M--V--LA--A---R------E----W-		-R----A----DR-D-
	Mycobacterium haemophilum	WP_047316799	-------VA--A--IR-----DE----L-		-R----A--P-DR-P-
	Mycobacterium heraklionense	WP_076048367	-I--LM-----A--IR-----QE----AH		-W----A----DA-P-
	Mycobacterium houstonense	WP_066901874	----L--I---A--IR-----EL----H-		-R----T----DG-D-
	Mycobacterium intermedium	WP_069420699	----L---A--A---R-----DE----WM		-RI---S----DR-P-
	Mycobacterium kansasii	WP_099225671	-------I---A-IIR-----EE----WS		-R----A---YDR-P-
	Mycobacterium kubicae	WP_085074402	-------IA--A--IR-----DE----WQ		-R----A--IYDR---
	Mycobacterium liflandii	WP_015353934	--------A--A--IR-----EE----WD		-RI---A--T-DR---
	Mycobacterium mageritense	WP_051578581	--------A--A--IR-----EL----MR		-I---T----D--P-
	Mycobacterium marinum	WP_012392075	--------A--A--IR-----EE----WD		-RI---A--T-DR---
	Mycobacterium marinum MB2	EPQ70844	--------A--A--IR-----EE----WD		-RI---A--T-DR---
	Mycobacterium marinum str. Europe	EPQ72340	--------A--A--IR-----EE----WD		-RI---A--T-DR---
	Mycobacterium neworleansense	WP_090516801	----L--I---A--IH------L----H-		-RI---T----DR-P-
	Mycobacterium nonchromogenicum	WP_085139592	-I--LM-----A--IR-----QQ----AH		-W----A----DA-P-
	Mycobacterium peregrinum	WP_055119158	----L--IA--A--IR-----DL----H-		-R----A----DQ-P-
	Mycobacterium porcinum	WP_069427529	----L--I---A-IIR-----EL----H-		-RI---T----DR-P-
	Mycobacterium pseudoshottsii J	GAQ36616	--------A--A--IR-----EE----WD		-RI---A--T-DR---
	Mycobacterium riyadhense	WP_085253038	-------VL--A---R-----NE----WQ		-R----A---YDR-P-
	Mycobacterium rufum	KGI70526	-------V---A--IR-----EL----R-		-----A----DD-P-
	Mycobacterium setense	WP_039327319	----L--I---A--IR-----EL----H-		-RI---T----DG-P-
	Mycobacterium shinjukuense	WP_083048232	-V------M--A--IR-----DE----W-		-RI-------YDR-P-
	Mycobacterium szulgai	WP_068158297	-------IA--A--IR-----DE----WQ		-R----A--IYDR---
	Mycobacterium terrae	WP_085260161	-V--L--VG--A--IR-----E-----ER		-I---A----DR-P-
	Mycobacterium ulcerans	WP_011738467	--------A--A--IR-----EE----WD		-RI---A--T-DR---
	Mycobacterium ulcerans str. Harvey	EUA93387	--------A--A--IR-----EE----WD		-RI---A--T-DR---
	Mycobacterium vulneris	CDO29445	----L--I---A-IIR-----EL----H-		-RI---T----DR-P-
	Mycobacterium wolinskyi	WP_085143641	-------I---A--IR-----EL----W-		-RI---T----DG-P-

Figure S24. Partial sequence alignment of a conserved region of a membrane protein showing an eight amino acid insertion that is specific for members of the “Tuberculosis” clade.

Group	Species	Accession	18	Insertion	67
"Tuberculosis" clade (20/21)	*Mycobacterium tuberculosis*	AMC72874	TGGGTLLPMADVIRMTSHAHHY	SPASGRYP	QAIFDHGTPLALYHTKRLAS
	Mycobacterium africanum GM0411	CCC28551	----------------------	--------	--------------------
	Mycobacterium africanum K85	KBF47630	----------------------	--------	--------------------
	Mycobacterium africanum MAL010	KBH37364	----------------------	--------	--------------------
	Mycobacterium bovis	AKR01299	----------------------	--------	--------------------
	Mycobacterium bovis AF2122/97	YP_009360856	----------------------	--------	--------------------
	Mycobacterium bovis AN5	ESK75391	----------------------	--------	--------------------
	Mycobacterium bovis B2 7505	KAN87758	----------------------	--------	-------K------------
	Mycobacterium bovis BCG	AAB96960	----------------------	--------	--------------------
	Mycobacterium bovis BCG str. A	AHM09807	----------------------	--------	--------------------
	Mycobacterium bovis BCG str. P	CAL70112	----------------------	--------	--------------------
	Mycobacterium bovis Bz 31150	KAN92248	----------------------	--------	--------------------
	Mycobacterium bovis MAL010093	KBG51344	----------------------	--------	--------------------
	Mycobacterium canettii	WP_015292722	----SRV--S-L---A-N----		L-L--GAK------------
	Mycobacterium caprae	WP_083647839	----------------------	--------	--------------------
	Mycobacterium microti	AMC57538	----------------------	--------	--------------------
	Mycobacterium mungi	OAQ17315	----------------------	--------	--------------------
	Mycobacterium orygis	WP_081608392	----------------------	--------	--------------------
	Mycobacterium orygis 112400015	EMT33889	----------------------	--------	--------------------
	Mycobacterium pinnipedii	PRH91488	----------------------	--------	--------------------
	Mycobacterium africanum	AMC61867	----------------------	--------	--------------------
Other *Mycobacteriaceae* (1/51)	*Mycobacterium gordonae*	WP_065044389	-A-------S--L-LATS----	A-------	-T---QDKT-----G----T
	Mycobacterium alsense	WP_083141168	-A--S----T-----AG-----		L-----AK------------
	Mycobacterium angelicum	ORA12304	-----I---P----LGRQ-N--		L----Q-K------------
	Mycobacterium arosiense	WP_083066330	--A--I---S----LAR-----		L------K------------
	Mycobacterium asiaticum	OBI87458	-S--S------L--LAAQ----		L----DAK-----------N
	Mycobacterium avium	WP_040963252	---------S----LSR-----		L----Q-KA---LYHTKRLA
	Mycobacterium bohemicum	WP_085181641	-A----V--S----WAG-----		L------K------------
	Mycobacterium colombiense	WP_064877140	-----I---S----LAR-----		L------KA-----------
	Mycobacterium conspicuum	WP_085233296	-----I---S----LAR-----		L----K-KA-----------
	Mycobacterium europaeum	CQD22373	-A----V--S----WAT-----		L----KSRS---W-------
	Mycobacterium gastri	WP_036415427	----SR---S-L---A------		L-L--R-KA-----------
	Mycobacterium heckeshornense	WP_048890622	----S----SE---LAQ-----		L----N-RA--------I--
	Mycobacterium heidelbergense	ORA65721	----S---LS-----A------		L-L--NAK---------F--
	Mycobacterium interjectum	WP_066907899	-A----V--P----WAG-----		L----KAKS----S------
	Mycobacterium intracellulare	WP_064893654	----S-V--S-L---A------		L----R-RA-S-H-------
	Mycobacterium kansasii	KZS64453	----SQ---S-L--LA------		L-L--D-KA-----S-----
	Mycobacterium kubicae	WP_085072951	---------S----LSR-----		L----R-NA-----------
	Mycobacterium lacus	WP_085155832	----SRV--S-L---A-S----		L-L--G-K------A-----
	Mycobacterium malmoense	WP_065442061	-A----V--S----WAG-----		L----RAR------------
	Mycobacterium mantenii	WP_083099146	-----VV--S----WA------		L------KA---H-------
	Mycobacterium marseillense	ORA94115	-----I---S----LDR--N--		L----K-NA-----------
	Mycobacterium nebraskense	WP_085165693	-----R---S----LAR-----		L-V--K-KAV----G-----
	Mycobacterium palustre	WP_085079703	------V--S----WA---Y--		L----R-KA---H-------
	Mycobacterium paraense	ORW52145	-A----V--S----WAG-----		L----NARS--------I--
	Mycobacterium paraffinicum	OJZ62670	-A----V--S----WAG-----		L----R-RS-----AT-F--
	Mycobacterium parascrofulaceum	WP_007170112	-----I---S----LAR-----		L----NAK-V----------
	Mycobacterium paraseoulense	WP_083172732	-----I---S----LAR-----		L-V--R-E------G-----
	Mycobacterium parmense	WP_085269507	-----I---S----LAG--N--		L----K-KA-----------
	Mycobacterium scrofulaceum	ORB62829	-A----V--S----WAG-----		L-----AR------------
	Mycobacterium shinjukuense	WP_083046789	----S----SE---LAA-----		LR-----RE-----------
	Mycobacterium simiae	WP_061555588	----SVI--T-LL-LA------		L-L--SAK---------V--
	Mycobacterium szulgai	WP_085669083	-----I---S----LGRQ-N--		L----E-K--T---------
	Mycobacterium xenopi	WP_003918915	----S----SE---LAQ-----		L----K-RA--------I--
	Mycolicibacillus trivialis	ODR01013	---------S----LAR-----		L----R-KTIG-F-------

Figure S25. Partial sequence alignment of a conserved region of the hypothetical protein RN11_1864 showing an eight amino acid insertion that is specific for most members of the "Tuberculosis" clade and is absent from most other *Mycobacteriaceae*.

Group	Species	Accession	18		55
**“Tuberculosis” clade (9/9)**	*Mycobacterium tuberculosis*	CKT50978	VLPGVAVTAGMMWSHIIAGAVVAVLG		FYFGMRTRAAAN
	Mycobacterium bovis	WP_099180604	--------------------------		------------
	Mycobacterium canettii	WP_015287774	--------------------------		------------
	Mycobacterium caprae	APU24423	--------------------------		------------
	Mycobacterium microti	AMC57641	--------------------------		------------
	Mycobacterium mungi	OAQ17092	--------------------------		------------
	Mycobacterium orygis	EMT37533	--------------------------		------------
	Mycobacterium pinnipedii	PRH90401	--------------------------		------------
	Mycobacterium africanum	CCC25261	--------------------------		------------
**Other *Mycobacteriaceae* (0/63)**	*Mycobacterium alsense*	WP_083140657	-----TL----I--NVV---LMTF--	LNA	T-V------
	Mycobacterium aquaticum	WP_083166779	----I---VLS--L-FL--	LYA	T-L---V-
	Mycobacterium arosiense	WP_083066211	I----TL----T--NVV---LLTF--	LNA	T-------
	Mycobacterium avium	WP_023864782	I----VL----T--NVV---LLTF--	LNA	T--------S-
	Mycobacterium avium subsp. hominissuis	KDO92942	I----VL----T--NVV---LLTF--	LNA	T--------S-
	Mycobacterium branderi	WP_083133168	-SPHG--I--NV-----LT---	LNA	M-----A-S
	Mycobacterium celatum	WP_085168241	-INH-SPHG--I--NV-----LT---	LNA	M-----A-S
	Mycobacterium colombiense	WP_044483380	I----VM----T--NVV---LL-F--	FNA	T--------
	Mycobacterium dioxanotrophicus	WP_087081014	----I--NVVS-V--CL--	LYA	A-L---F-
	Mycobacterium europaeum	WP_085243205	------L---T---NVV---LLT---	LNA	A---
	Mycobacterium florentinum	WP_085222452	-V----L----I---VV---LMTF--	LNA	A----------D
	Mycobacterium fragae	WP_085199408	-IH--SPHG--I--NV-----LT---	LNA	M-----A-S-
	Mycobacterium gastri	WP_036412282	--Q-ITR----------------F--	LTA	T----------T
	Mycobacterium genavense	WP_025736437	-VS-----T--V---VV--VLLTF--	LNA	A--------T-D
	Mycobacterium haemophilum	WP_047314456	I-H-ISL----I--NV----LL-C--	LNA	T-------NT-S
	Mycobacterium heckeshornense	WP_048890442	--H--SP----I--NVV----IT---	LTA	V-----A-S
	Mycobacterium heidelbergense	WP_083075832	I---L-L----I--NVV-----TL--	VTA	T----------
	Mycobacterium houstonense	WP_066900890	----I---V-S-GL-ML--	FNA	M-----V-
	Mycobacterium interjectum	WP_085205038	------L----L--NVV---LL----	LNA	T---V------T
	Mycobacterium intracellulare A	AFC46122	I--D-TL-T--T--NVV---LLTF--	LNA	T--------
	Mycobacterium kansasii	WP_075512500	--Q-ITR----------------F--	LTA	T---I------T
	Mycobacterium kyorinense	WP_045382986	-SPHG--I--NV-----LTL--	LNA	M-----A-S
	Mycobacterium lacus	WP_085162762	I---M------A---V----L--FF-	LTA	T----------T
	Mycobacterium lentiflavum	WP_090598605	-VT---L-T-TI---VV---LLTF--	LNA	A----------D
	Mycobacterium mageritense	WP_036432474	----I---VVS-VI-T---	LDA	A-----L-NS
	Mycobacterium malmoense	WP_083010688	I---L-L---II--NVV-----TL--	MTA	T---------
	Mycobacterium mantenii	WP_083097635	I----TL----T--NVV---LLTF--	LNA	T---------
	Mycobacterium marseillense	WP_083020379	I--D--L-T--T--NVV---LLTF--	LNA	T--------
	Mycobacterium montefiorense	WP_108922082	-V----M-S--I--NVV---LMTF--	LNA	T---------TH
	Mycobacterium nebraskense	WP_046186670	I-----L-------N-V---LLTL--	LNA	T---R-----TT
	Mycobacterium neworleansense	WP_090516614	-T--I---VVS--L-ML--	FNA	M-----V-
	Mycobacterium noviomagense	WP_083089088	-MH--SP----I--NVV----IT-M-	LIA	V-----A-S
	Mycobacterium palustre	WP_085076323	----FTP----I--NVV---LLTF--	LNA	T-------E-
	Mycobacterium paraense	WP_085098591	----FTPS---I--NVV--GLL-F--	LNA	T-------EV-T
	Mycobacterium paraffinicum	WP_073877841	I---MTL---V---NVV---LLTL--	VNA	A---R------T
	Mycobacterium paraintracellulare	AFC56520	I--D-TL-T--T--NVV---LLTF--	LNA	T--------
	Mycobacterium paraseoulense	WP_083175724	----F-L-------NVV---LLT---	LNA	T---
	Mycobacterium peregrinum	WP_055119499	S---I---V-S--L-ML--	FTA	M----QV-
	Mycobacterium porcinum	WP_069427114	----I---V-S--L-M---	FTA	M-----V-
	Mycobacterium riyadhense	WP_085248947	I-Q-ITR---------L---L--F--	LTA	T-------ST-T
	Mycobacterium scrofulaceum	WP_083175612	I----HL---T---NLV---LLT---	LNA	T---R------T
	Mycobacterium setense	WP_039312628	-T--I---V-S--L-M---	FNA	M-----V-
	Mycobacterium sherrisii	WP_069401653	----LTL-G-VI---VV---LLTF--	LNA	T-------D-
	Mycobacterium shimoidei	WP_069394244	--ST-HP-I--NVV---LLTF--	LNA	A-----A-NS-T
	Mycobacterium shinjukuense	WP_083045791	I-R--T-------------L---GM-	LTA	T----------T
	Mycobacterium talmoniae	WP_071021800	--S--SP----I--NV---GLLMA--	LTA	V-----A-SE-T
	Mycobacterium triplex	WP_036466065	-VS-----T--A---VV---LLTF--	LNA	A----------D
	Mycobacterium vulneris	WP_085291183	I----VM-T--T--NVV---LL-F--	FNA	T--------
	Mycobacterium xenopi 3993	EUA43204	--H--SP----I--NVV----IT---	LTA	V-----A-S
	Mycolicibacter heraklionensis	WP_064888582	S-E-I--NV-T--LLTI--	LNA	A-----A-SES
	Mycolicibacter kumamotonensis	WP_109510999	S-E-I--NV-T--LLTI--	LNA	A-----A-GE-
	Mycolicibacter longobardus	WP_085267047	SSD-I--NV-T---LT---	MNA	A-----A-GE-
	Mycolicibacter nonchromogenicus	WP_085139040	I-TDGSPSTA-I--NV-T---MT---	LNA	A-----A-SES
	Mycolicibacter sinensis	WP_064856983	--RDMSP-P--I--NVV----LTA--	LTA	A---R-A-HD-T
	Mycolicibacter terrae	WP_095173667	S-E-I--NV-T--LLTI--	LNA	A-----A-GE-
	Mycolicibacter virginiensis	WP_105294441	S-E-I--NV-T--LMTI--	LNA	A-----A-SES
	Mycolicibacterium boenickei	WP_077739841	----I---V-S--L-M---	FNA	M-----V-
	Mycolicibacterium chubuense	WP_014814357	-ASP--S----NVVI----T---	LVA	TGL---A-TG-
	Mycolicibacterium farcinogenes	WP_036390558	ST--I---V-S--L-M---	FNA	M-----V-

Figure S26. Partial sequence alignment of a conserved region of a transmembrane protein showing a three amino acid deletion that is specific for members of the “Tuberculosis” clade.

Group	Species	Accession	195		242
"Tuberculosis" clade (9/9)	*Mycobacterium tuberculosis*	CFK13498	RLRLLDTLDLFGIALGMAAFRPG	RPS	RTPAQLRTLLRRVSGVDAVIDK
	Mycobacterium bovis	WP_024459288	-----------------------	---	----------------------
	Mycobacterium canettii	WP_044095737	-------------------L---	---	----------------------
	Mycobacterium caprae	APU24438	-----------------------	---	----------------------
	Mycobacterium microti	AMC57666	-----------------------	---	----------------------
	Mycobacterium mungi	WP_064319877	-----------------------	---	----------------------
	Mycobacterium orygis	EMT37554	-----------------------	---	----------------------
	Mycobacterium pinnipedii	PRH90419	-----------------------	---	----------------------
	Mycobacterium africanum	WP_031667806	-----------------------	---	----------------------
Other *Mycobacteriaceae* (0/36)	*Mycobacterium arosiense*	WP_083064547	-R---G------T--AV----Q-		-S---VL-----M----G-LAS
	Mycobacterium avium	WP_003874092	-E----------T--AI--I-R-		AG----------I------LAE
	Mycobacterium avium subsp. avium	ETB24418	-E----------T--AI--I-R-		AG------M---I------LAE
	Mycobacterium avium subsp. paratuberculosis	ETA97025	-E----------T--AI--I-R-		AG----------I------LAE
	Mycobacterium branderi	WP_083133152	-R--V-------V-AAV-GI-R-		ASK--V-AR---L-RI---LAE
	Mycobacterium colombiense	OBJ13571	-Q----------T--AV----Q-		-----VL---H-M------LA-
	Mycobacterium europaeum	WP_090419913	-T-------------AV--I-Q-		--AT-V-A----T------LSR
	Mycobacterium gastri	WP_036411872	-----ES---------V--V-R-		AP--RV-A----A----V-VAG
	Mycobacterium gordonae	WP_069433669	-RQ-MA-V-------AV--L-Q-		SG---V-A-W-------D-VER
	Mycobacterium haemophilum	WP_047314441	-R----------T--AV--L-K-		G-AR-V-A-----------V--
	Mycobacterium haemophilum DSM	ALL56205	-R----------T--AV--L-K-		G-AR-V-A-----------V--
	Mycobacterium intermedium	WP_069421458	-Q----S--V-----VV--L-R-		AGQ-EV-A---------G-TAR
	Mycobacterium intracellulare	WP_014381429	CQ----------T--AV--I-Q-		-----V-A----M------LA-
	Mycobacterium intracellulare 1	EUA53746	-Q----------T--AV--I-Q-		-----V-A----M------LA-
	Mycobacterium kansasii	KEP44552	-----EA---------V--V-R-		AG--EV-A--H-A------VAQ
	Mycobacterium lacus	WP_085162744	-M-V-AG-----T---I----QH		---E-V-A-----------V-R
	Mycobacterium lentiflavum	WP_090598680	-M----S--V--T--AV--I-RN		A----F-A------------AR
	Mycobacterium malmoense	WP_065517420	-----Q----------V----Q-		--A--V-A----T------LAQ
	Mycobacterium mantenii	WP_083097674	-Q----------A--AV--L-Q-		-----VL---H-M---E--VAR
	Mycobacterium marseillense	WP_083020360	-Q----------T--AV--I-Q-		--R--V----C-M------LA-
	Mycobacterium microti	WP_101528403	-Y----N--M--T--SI--V-RA		G----V-S---------S-VAA
	Mycobacterium nebraskense	KKC01828	----------------V--V-Q-		--A--V-----T------LSR
	Mycobacterium noviomagense	WP_083089352	-Q--VE-F-M---S-AV--A-Q-		-SFG-V-AW---A-C--V-V-R
	Mycobacterium palustre	WP_085078273	-S---A------T-VSI--L-R-		AA---V-A----I------LGQ
	Mycobacterium paraintracellulare	WP_014386008	-Q----------T--AV--I-Q-		-----VL------------LG-
	Mycobacterium parascrofulaceum	EFG76787	-----Q----------V----Q-		--A--V-A----T------LAQ
	Mycobacterium persicum	WP_083154782	-M---EA---------V--V-R-		VP--GV-A----A------VAG
	Mycobacterium riyadhense	WP_085248928	-E---Q------T---I--A-R-		KSA--I---W------------
	Mycobacterium saskatchewanense	WP_085258037	-V----R-----V--A-V-V-Q-		--SG-V-A--H-MG-A-D-LRA
	Mycobacterium scrofulaceum	WP_083178380	-----Q----------V----Q-		--A--V-A----T------LSQ
	Mycobacterium sherrisii	WP_069399968	-V----S--M--T--SV--V-R-		GS---V-S---------T-VAA
	Mycobacterium shigaense	WP_096436629	-R----------T---V--AHR-		--R--VAA----M------V-R
	Mycobacterium shinjukuense	WP_083045775	------V--VCR-		G-AE---A------
	Mycobacterium simiae	WP_044509427	-Y----N--M--T--SI--V-RA		G----V-S---------S-VAA
	Mycobacterium timonense	WP_083187691	-E----------T--AI--I-R-		AG------M---I------LAE
	Mycobacterium vulneris	WP_085291166	-Q----S-----T--AV----Q-		-----VL---H-M------LA-

Figure S27. Partial sequence alignment of the hypothetical protein ERS181347_00724 showing a three amino acid insertion that is specific for members of the "Tuberculosis" clade.

			10		59
**“Tuberculosis” clade (11/11)**	*Mycobacterium tuberculosis*	CEZ53357	AGVDELVAAIAPGL	AGL	GLPVINRREVVLVTGPWLAGVSGVRAALAERLP
	Mycobacterium bovis	WP_079367471	--------------	---	---------------------------------
	Mycobacterium bovis 04-303	ESK77813	--------------	---	---------------------------------
	Mycobacterium bovis B2 7505	KAN92334	--------------	---	---------------------------------
	Mycobacterium canettii	WP_014000118	--------------	---	---------------------------------
	Mycobacterium caprae	APU24439	--------------	---	---------------------------------
	Mycobacterium microti	AMC57667	--------------	---	---------------------------------
	Mycobacterium mungi	OAQ17110	--------------	---	---------------------------------
	Mycobacterium orygis	EMT37555	--------------	---	---------------------------------
	Mycobacterium pinnipedii	PRH90420	--------------	---	---------------------------------
	Mycobacterium africanum	WP_031670740	--------------	---	---------------------------------
**Other *Mycobacteriaceae* (0/45)**	*Mycobacterium alsense*	WP_083139375	-E--A-----G-H-		DP-A-H--D------------TA-AT--R----
	Mycobacterium angelicum	WP_083115428	-Q--A-----G-R-		DA-A---------A--------S-A---R----
	Mycobacterium asiaticum	OBK20320	-R--AM----G-N-		-P------D---------S--TS-ATV-RQQI-
	Mycobacterium branderi	WP_083133151	-D--A--G--G-R-		SA---A--DA--------------A-V-R----
	Mycobacterium celatum	WP_085168233	-D--A--G--G-R-		SA-G-Q--DA--------------VSV-R----
	Mycobacterium europaeum	CQD08726	-A--A---R-G-A-		DA-AVH--D--------M----A-A-V-T----
	Mycobacterium florentinum	WP_085222998	-Q--------G-R-		DS-AVS--D--------M---T--AT--G----
	Mycobacterium fragae	WP_085199132	----A--GS-G-R-		DA-I-Q--D------------T--LN--RD---
	Mycobacterium gastri 'Wayne'	ETW25013	--A-----G-KV		-P---C--D-------------S-V---RQ-A-
	Mycobacterium genavense	WP_025736453	-E--------G-R-		DP-A-T--D--------M---T--AT--S----
	Mycobacterium gordonae	WP_082658447	-R--AM---VG-N-		-P-A----D-------------S-A---RQ-I-
	Mycobacterium haemophilum	WP_047314440	-Q--A----VG---		SS-A---Q---------M--AT--A---R----
	Mycobacterium heckeshornense	KMV23493	-R--A-----G-R-		AA-RVQ--D---------------A---R----
	Mycobacterium intermedium	WP_069421459	-R--AM----G-R-		-V-T----G------------T--VI--R----
	Mycobacterium kansasii	KZS68912	-R--A-----G-KA		-P---Q--D-------------S-V---RQ-A-
	Mycobacterium komaniense	WP_090277595	-SA-AV----E---		AA-AVRA-D-----------TTS-V---R--M-
	Mycobacterium kyorinense	WP_052425665	-D--AM-G--G-R-		SA---Q--DA--------------VSV-RQ---
	Mycobacterium lacus	ORW00162	-R--A-I---G-Q-		DS-A-D--D---------------VGV-R----
	Mycobacterium lentiflavum	WP_090608336	--------G-R-		ES--VS--D--------M---T--ATV-G----
	Mycobacterium malmoense	WP_065442438	-A--A---GV--E-		EA-AVH----------------A-A---R----
	Mycobacterium marinum	BBC63542	-R-----L--G-K-		-A-S----D-----------T-S-IT--RR---
	Mycobacterium marinum E11	CDM74562	-R-----L--G-K-		-A-S----D-----------T-S-IT--RR---
	Mycobacterium marinum M	ACC38917	-R-----L--G-K-		-A-S----D-----------T-S-IT--RR---
	Mycobacterium montefiorense	GBG38016	-Q--------G-R-		ES-A-S--D--------M---T--ATV-G----
	Mycobacterium palustre	WP_085078342	-Q--A---G-G-R-		DA-AVT--D------------TA-V---R----
	Mycobacterium paraffinicum	WP_073877657	-E--A---SVG-E-		DA-AVH--D-------------A-A---R----
	Mycobacterium parascrofulaceum	EFG76788	-A--A---GV--E-		EA-AVH----------------A-A---RD---
	Mycobacterium paraseoulense	WP_083174838	-E--A---G-G-E-		DA-AVH--D--------M----A-A-V-R----
	Mycobacterium pseudoshottsii	WP_086085135	-R-----L--G-K-		-A-S----D-----------T-S-IT--RR---
	Mycobacterium pseudoshottsii J	BBA86270	-R-----L--G-K-		-A-S----D-----------T-S-IT--RR---
	Mycobacterium riyadhense	WP_085248927	-R--A-----GRR-		DS-A----D---------------V---RD---
	Mycobacterium saskatchewanense	WP_085258063	-Q--A-----G-D-		-P-AV---D----A--------S-A---R----
	Mycobacterium scrofulaceum	WP_067282778	-Q--A---S-G-A-		DP-AVH--D-------------A-A---R----
	Mycobacterium senuense	WP_085082032	-A--A---E-----		RP-AVR--N--M----------A-A-M-----
	Mycobacterium shimoidei	WP_069394259	-E--AMIG--S-R-		PA-TVQ--D---------------AG-------
	Mycobacterium shinjukuense	ORB72323	-R--A----VG-R-		-V-A--------------------H---R----
	Mycobacterium simiae	WP_061557986	-E--------G-E-		DA---I--D--------M---T--ATV-R----
	Mycobacterium szulgai	WP_085673213	-Q--A-----G-R-		DA-A---------A--------S-A---R----
	Mycobacterium talmoniae	OHV05957	-Q--A---G-G-RV		RP-AVQ--D---------------A---RD---
	Mycobacterium triplex	WP_036472013	-E--------G-R-		DP-A-T-----------M---T--AT--G----
	Mycobacterium ulcerans	WP_071497524	-R-----L--G-K-		-A-S----D-----------T-S-IT--RR---
	Mycobacterium ulcerans Agy99	ABL03689	-R-----L--G-K-		-A-S----D-----------T-S-IT--RR---
	Mycobacterium ulcerans str. Harvey	EUA91645	-R-----L--G-K-		-A-S----D-----------T-S-IT--RR---
	Mycobacterium ulcerans subsp. shinshuense	BAV43493	-R-----L--G-K-		-A-S----D-----------T-S-IT--RR---
	Mycobacterium xenopi	WP_004571681	-R--A--G--G-R-		SA-GVQ---------------T--A---R----

Figure S28. Partial sequence alignment of a conserved membrane protein showing a three amino acid insertion that is specific for members of the “Tuberculosis” clade.

"Tuberculosis" clade (10/10)

```
                                                      144                                              193
Mycobacterium tuberculosis            AIH34285       QDQFFNAPANGPAGLFLARYPD QYGK RLKRAEQMADWIDRTLIDPETHLV
Mycobacterium bovis                   WP_079293488   ---------------------- ---- ------------------------
Mycobacterium bovis BCG               AMC48903       ---------------------- ---- ------------------------
Mycobacterium canettii                WP_015288989   ---------------------- ---- ------------------------
Mycobacterium caprae                  APU24584       ---------------------- ---- ------------------------
Mycobacterium microti                 AMC57837       ---------------------- ---- ------------------------
Mycobacterium mungi                   OAQ17484       ---------------------- ---- ------------------------
Mycobacterium orygis                  EMT37383       ---------------------- ---- ------------------------
Mycobacterium pinnipedii              WP_105826294   ---------------------- ---- ------------------------
Mycobacterium africanum               CCC25439       ---------------------- ---- ------------------------
```

Other *Mycobacteriaceae* (4/65)

```
Mycobacterium litorale                WP_078017669   --------------I-----LT SQ-E -VR--Q-----L-E-----D----
Mycobacterium parmense                WP_085271685    -------------I-----S- DR-N -----G-------------D----
Mycobacterium vaccae                  WP_003932915   -------------AI-----LS SL-E P-R--Q-------E----------
Mycolicibacterium aurum               WP_087030337   --------------I-----LT SH-D -VR--Q-----M-E-----D----
Mycobacterium aquaticum               WP_083165348   --------------I-----D-      --R--Q-------E----------
Mycobacterium arosiense               WP_083064206    -------------I-----G-      H------------Q----------
Mycobacterium avium                   WP_003873851    -------------I-----G-      H--------N---E----------
Mycobacterium branderi                WP_083133853   --------------I-----G-      -----Q-------K-----D----
Mycobacterium celatum                 WP_062539905   --------------I-----G-      -----Q-------A----------
Mycobacterium colombiense             WP_064880705   --------------I-----GG      H-------S----Q----------
Mycobacterium conspicuum              WP_085236310   --------------I-----G-      --------G----Q----------
Mycobacterium dioxanotrophicus        WP_087080095   --------------I-----D-      --R--Q-------D----------
Mycobacterium fragae                  WP_085197892   --------------I-----G-      --R--Q-------A-----Q----
Mycobacterium genavense               WP_025736934   --------------I-----GE      H----A--G----Q----------
Mycobacterium haemophilum             WP_047313750   --------------I-----G-      --R---------------------
Mycobacterium heckeshornense          WP_048889442   -------------AI-----GN      -----Q-------A----------
Mycobacterium heidelbergense          WP_083074160   --------------I-----G-      --------G---------------
Mycobacterium holsaticum              WP_069404626   --------------I-----D-      --R--Q------EE----------
Mycobacterium kansasii                ORB85236       --------------I-----D-      W-G--Q------------------
Mycobacterium kyorinense              WP_045374037   --------------I-----G-      -----Q-------A----------
Mycobacterium lacus                   WP_085160062   --------------I----SGG      -MR--Q-------E-----D----
Mycobacterium lentiflavum             WP_090599306   --------------I-----GA      -----A--G----Q----------
Mycobacterium mageritense             WP_036433503   --------------I-----D-      --R--Q-------E----------
Mycobacterium malmoense               WP_065477819   --------------I-----SE      KMR--Q-------E----------
Mycobacterium marinum                 WP_103654799   --------------I-----D-      --A-----G---------------
Mycobacterium marseillense            WP_083019135   --K-----------I-----G-      H--------N---Q--V-------
Mycobacterium nebraskense             WP_046184827   --------------I-----GH      -MR--Q------------------
Mycobacterium neworleansense          WP_090516231   --------------I-----D-      --R--Q-------D----------
Mycobacterium noviomagense            WP_083088613   -------A------I-----G-      -----Q-------A----------
Mycobacterium palustre                WP_085078826   --------------I-----G-      ------------------------
Mycobacterium paraense                WP_085093052   --K-----------I-----GE      H-Q--D--S---------------
Mycobacterium paraffinicum            WP_073874446   --------------I-----GE      KMR--Q-------E----------
Mycobacterium peregrinum              WP_055110674   --------------I-----D-      --R--Q-------D----------
Mycobacterium porcinum                WP_069425223   --------------I-----D-      --R--Q-------D----------
Mycobacterium rhodesiae               WP_083117990   --------------I--S--D-      --R--Q-------E----------
Mycobacterium saskatchewanense        WP_085257856   --------------I-----G-      --------G----Q----------
Mycobacterium scrofulaceum            WP_067268722   --------------I-----GE      -MR--Q-------E----------
Mycobacterium septicum                WP_044515389   --------------I-----D-      --R--Q-------D----------
Mycobacterium shimoidei               WP_069395966   --------------I-----G-      --E--QK------A----------
Mycobacterium shinjukuense            WP_083052071   --R-----------I-----DG      W-P--AA-----E-----------
Mycobacterium simiae                  WP_061558126   E-------------I-----G-      H-------G----------Q----
Mycobacterium smegmatis               WP_058125219   --------------I-----D-      --R--Q-------E-----D----
Mycobacterium sphagni                 WP_094480727   --------------I-----G-      --R--Q-------E----------
Mycobacterium talmoniae               WP_071024725   --------------I-----G-      -----Q-------K----------
Mycobacterium timonense               WP_083187512    -------------I-----G-      H--------N---E----------
Mycobacterium ulcerans                WP_071497704   --------------I-----D-      --A-----G---------------
Mycobacterium ulcerans Agy99          ABL02869       --------------I-----D-      --A-----G---------------
Mycobacterium vulneris                WP_065462411   --------------I-----D-      --R--Q-------D----------
Mycobacterium wolinskyi               WP_085145447   --------------I-----D-      --R--Q-------E-----D----
Mycobacterium xenopi                  WP_003922468   --------------I-----G-      -----Q-------A----------
Mycolicibacterium aromaticivorans     WP_036342979   --------------I--T--E-      --R--Q-------E----------
Mycolicibacterium boenickei           WP_077740206   --------------I-----D-      --R--Q-------D----------
Mycolicibacterium brisbanense         WP_062829597   --------------I-----D-      --R--Q-------E----------
Mycolicibacterium chubuense           WP_014813742   --------------I-----E-      --R--Q-------D----------
Mycolicibacterium confluentis         WP_085151664   --------------I-----G-      --R--Q-------E----------
Mycolicibacterium fortuitum           WP_054601045   E-------------I-----D-      --R--Q-------D----------
Mycolicibacterium goodii              WP_049743204   --------------I-----D-      --R--Q-------E-----D----
```

Figure S29. Partial sequence alignment of the fructose-bisphosphate aldolase protein showing a four amino acid insertion that is specific for members of the "Tuberculosis" clade and is absent from most other *Mycobacteriaceae*.

```
                                                                   147                                                           206
                  Mycobacterium tuberculosis        AIH66129       SRDRNTGLLVMNNVAPPSRGTVYQMWLLG G AKGPRSAGTMGTAAVTPSTTATLTDLGAST
                  Mycobacterium bovis               WP_024458012   ----------------------------- - -------E----------------------
                  Mycobacterium bovis BCG           AMC49003       ----------------------------- - -------E----------------------
"Tuberculosis"    Mycobacterium canettii            WP_014000235   ----------------------------- - ------------------------------
clade             Mycobacterium caprae              APU24654       ----------------------------- - -------E----------------------
(10/10)           Mycobacterium microti             AMC57923       ----------------------------- - ------------------------------
                  Mycobacterium mungi               OAQ17415       ----------------------------- - ------------------------------
                  Mycobacterium orygis 112400015    EMT37301       ----------------------------- - ------------------------------
                  Mycobacterium pinnipedii          PRH92830       ----------------------------- - ------------------------------
                  Mycobacterium africanum           WP_031667293   ----------------------------- - ------------------------------
                  Mycobacterium asiaticum           WP_036352079   -----AAV------P--AP--------V-   -N--T----------S---K-M-D---SA-
                  Mycobacterium bohemicum           WP_085183500   -H---A-V------P---A--------I-   ----T-----D----A--------N--P--
                  Mycobacterium celatum             WP_062539160   --E--A-V------P---P---------S   N---V-----S----A-----V-SR--D-S
                  Mycobacterium chimaera            WP_089152267   -----A-V------P---P--------IS   -N--A------A---S---K---AN--S--
                  Mycobacterium conspicuum          WP_085235643   -----A-V------P--AP--------I-   SN--T---------------HMFN---H-K
                  Mycobacterium florentinum         WP_085222740   ----GA-V------P---P--------IN   -N--T-----S----S-------PN--K--
                  Mycobacterium fragae              WP_085197722   -----A-V------P---S---------S   D-A-Q-----NAT--R----EV-PN--S-S
                  Mycobacterium gastri              WP_036416484   -H---A-V------P---P----------   -N--T------P---A---K----N--N--
                  Mycobacterium genavense           WP_025737004   -----A-V------P---P--------ID   ----T-----S----S--------N--K--
                  Mycobacterium gordonae            WP_055578676   -----AAV------P---N----------   -D-----------T-Q-T----VNEI-SCR
                  Mycobacterium haemophilum         WP_047313689   -----A-V------P---P--------V-   SH--T-----S----A-----M-AN--TA-
                  Mycobacterium interjectum         WP_066917669   -----AAV-L----P---P--------I-   TN--T------A---L---KD---NI-S-R
                  Mycobacterium intermedium         WP_069422294   -----A-V------P---S--------I-   -N--K-----DAQQ-R-T----VG---TCR
                  Mycobacterium kansasii            ORB85313       -H---A-V----D-P---P----------   -N--T------P---A---K---P---N--
                  Mycobacterium kubicae             WP_085075064   -----A-V------P---Q----------   ----T------A-------K---D---S--
                  Mycobacterium kyorinense          WP_045375833   --EK-A-V------P---P-S--------   -Q--T-----D-K--A-----V-PH--D--
                  Mycobacterium lacus               WP_085157940   -----A-V------P---P----------   -Q--T----------S---K---DN---A-
                  Mycobacterium lentiflavum         WP_090599635   ----GA-V------P--TP--------ID   S---T----------L--------N--K--
                  Mycobacterium malmoense           WP_065444686   -----A-V------P--KP----------   DR-AT-----N----A--------N--S-K
                  Mycobacterium mantenii            WP_083099821   -H---A-V------P---P----------   ---AT-------------------VN--S--
                  Mycobacterium marinum             WP_020731728   -----A-V------P---P--------VD   ----T------PT------K-------D--
Other             Mycobacterium montefiorense       WP_108926050   ----GA-V------P--TP--------ID   ----T-------T--S--------N--K--
Mycobacteriaceae  Mycobacterium nebraskense         KKC05152       -H---A-V------P--KP---------S   D---T-----S----A--------N--S--
(0/46)            Mycobacterium obuense             WP_046364380   --E-DS-V------T--KA--------VA   -D--H-----DAG--------V-P---S-Q
                  Mycobacterium paraense            WP_085172765   -----AAV------P---P--------I-   SN--T------A---L---KDM--NI-T--
                  Mycobacterium parafortuitum       WP_083144646   --E--S-V------S--QP--------I-   -D--H-----DSES-A-----V-P---S-Q
                  Mycobacterium paraseoulense       WP_083172766   -H---A-V------P--KP---------S   D---T-----S----A--------N--S--
                  Mycobacterium pseudoshottsii      WP_086085295   -----A-V------P---P--------VD   ----T------PT------K----Y--D--
                  Mycobacterium riyadhense          WP_085248258   -----A-V------P---P--------I-   -N--T------A-------K-M-P---S--
                  Mycobacterium rufum               KGI69723       --E-DS-V------P--KP--------VA   -D--H-----DAK--A-----V-P---S-R
                  Mycobacterium scrofulaceum        WP_083179878   -H---A-V------P--KP----------   D--AT-----N----A--------N--S-K
                  Mycobacterium sherrisii           WP_069400680   -----A-V--L-------P-----L--ID   ----T------N---L----E------K--
                  Mycobacterium shigaense           WP_096437060   -H---A-V----D-P--AP--------I-   -N--K-V----P---------M-ANI-S--
                  Mycobacterium shinjukuense        WP_083046602   -----A-V------P---P--------I-   SS--T--------------K---PN--N--
                  Mycobacterium simiae              WP_061558188   -----A-V--L-------P-S---L--ID   ----T------N---S--------N--K--
                  Mycobacterium sphagni             WP_094484374   ---T-A-V---------PP-S-------D   GGT-------S----R-----V-----NTN
                  Mycobacterium szulgai             WP_068022496   -----A-V------P---Q----------   ----T------A-------K---D---S--
                  Mycobacterium triplex             WP_036466479   ----DA-V------P---P--------ID   ----T-----S----S--------N--K--
                  Mycobacterium tuberculosis        CNK55822       -----A-V------P--AP--------I-   SN--T---------------HMFN---H-K
                  Mycobacterium ulcerans            WP_096371800   -----A-V------P---P--------VD   ----T------PT------K----A--D--
                  Mycobacterium wolinskyi           WP_067843868   -----A-V----G-T--ET--------MR   DT--E-----DS---A-----V-P---DAS
                  Mycolicibacterium aurum           WP_087027015   --E--A-V------S--ES--------ID   EA--H---V-DAG--A-----V-P---D--
                  Mycolicibacterium canariasense    GAS98684       -KE--A-V---------AP---------R   ST--Q-----DQ---A-----V-P---DA-
                  Mycolicibacterium canariasense    WP_062659479   -KE--A-V---------AP---------R   ST--Q-----DQ---A-----V-P---DA-
                  Mycolicibacterium cosmeticum      WP_036397317   -KK--A-V---------AA---------R   DN--E-----DQ---A-----V-P---DA-
                  Mycolicibacterium diernhoferi     WP_073859462   --E--A-V---------ES--------ID   DA--H---V-DAG--A-----V-P---D--
```

Figure S30. Partial sequence alignment of the anti-sigma K factor protein showing a one amino acid insertion that is specific for members of the "Tuberculosis" clade.

			36		84
“Tuberculosis” clade (9/9)	*Mycobacterium tuberculosis*	CFR80796	ARAVQPDDALVVFFGSRNDQGM	DPE	DPEMLAEKVRDTFDLARHRAPSAS
	Mycobacterium bovis	WP_086449525	------G---------------	---	------------------------
	Mycobacterium canettii	WP_014000269	----------------------	---	------------------------
	Mycobacterium caprae	WP_075744462	----------------------	---	------------------------
	Mycobacterium microti	AMC58006	----------------------	---	------------------------
	Mycobacterium mungi	OAQ15855	----------------------	---	------------------------
	Mycobacterium orygis	EMT37058	----------------------	---	------------------------
	Mycobacterium pinnipedii	PRH91716	----------------------	---	------------------------
	Mycobacterium africanum	CCC25597	----------------------	---	------------------------
Other *Mycobacteriaceae* (0/73)	*Mycobacterium abscessus*	AMU19729	G-V-H---E------------V		---TY--VT-N-LES--RM--A-K
	Mycobacterium alsense	WP_083136232	D---KD-------------ESA		--QL--G------G---T---A-R
	Mycobacterium angelicum	WP_083113177	----K-G--------------V		--AL---R--N--G---RL----T
	Mycobacterium arosiense	WP_083066974	---------------------V		--AT-TV-THAAL----QL--A-R
	Mycobacterium asiaticum	OBK25681	----K---K-----------DV		--VL--QRAQA-LA---RL----R
	Mycobacterium avium	WP_099180031	-----S---------------V		--GL-I--THAAL----RL--A-R
	Mycobacterium bohemicum	WP_085179499	----K--------------E-V		--GV--QNI-K------RA----K
	Mycobacterium branderi	WP_083132158	--T-R-N--------------V		E--L-GQMAH--L---HRT--A-R
	Mycobacterium celatum	ORV18499	----H-N--------------V		E--I-GQMAH--L----RT--A-R
	Mycobacterium chelonae	WP_078323055	N-V-R---E------------V		--DVY--AT-S-LAS--RT--A-K
	Mycobacterium colombiense	WP_044483695	---------------------V		--AT-TV-TNAAL-V--QL--A-R
	Mycobacterium colombiense CECT	EJO90713	---------------------V		--AT-TV-TNAAL-V--QL--A-R
	Mycobacterium conspicuum	ORV35474	----KR---------------V		--GL--L-T-EA-L---RF--T-R
	Mycobacterium europaeum	CQD10342	-----G--V----------EPI		-ANVIGQ-A-S------RL----R
	Mycobacterium florentinum	WP_085222805	V-S-KN--V------------S		--G---GRA----EV--IL----R
	Mycobacterium gastri	WP_036415996	----K---V----------E-V		E-QL----AGG------RL----R
	Mycobacterium gastri 'Wayne'	ETW23170	----K---V----------E-V		E-QL----AGG------RL----R
	Mycobacterium genavense	WP_025738082	V-S-KA--V------------T		--GL--GRA----QI--ILT-T-R
	Mycobacterium gordonae	WP_055578894	-KV-K-E-V-----------DV		--VL--QR-HA--T---RL----R
	Mycobacterium heckeshornense	WP_099868994	----K---V------------V		--GL---MA-NA-----RIT-TGR
	Mycobacterium heidelbergense	WP_083074522	G---K----------------V		---Q---RAG-A-----I-----R
	Mycobacterium immunogenum	WP_081272313	G-V-H---E------------V		--DAY--AT-S-LES--RM--A-K
	Mycobacterium interjectum	WP_066917754	----K-A--------------V		--ALVG--A--A-N---RF----R
	Mycobacterium intermedium	WP_069421895	--V-K---------------DV		--VL-FARAH--YA---RF--A-K
	Mycobacterium intracellulare	OBH44904	----H----------------I		--LL-T--THAAL----RF--A-R
	Mycobacterium intracellulare M	ETZ27230	----H----------------I		--LL-T--THAAL----RF--A-R
	Mycobacterium kansasii	ORB85383	----K---V----------E-V		---L-S--A-G------RL--G-R
	Mycobacterium kansasii ATCC 12	AGZ52059	----K---V----------E-V		--QL----AGE------RL----R
	Mycobacterium kubicae	WP_085075091	----KG---------------V		-LGQ------AAYG--HDL----R
	Mycobacterium kyorinense	ORW05915	--T-R-N--------------V		E--L-GQMAH--L---HRT--A-R
	Mycobacterium lacus	WP_085157202	----KS--V-----------RA		---L---RA--------RL----R
	Mycobacterium lentiflavum	WP_090599874	V-S-KA--V------------T		--G---GRA----QV--IL--T-R
	Mycobacterium malmoense	OCB51307	-----G--V----------EPI		--GVIGQ-A-S------RL----R
	Mycobacterium marinum str. Europe	EPQ70038	-G--K----------------V		-LGL-GDRA-NA----HRV----R
	Mycobacterium microti	WP_101528209	--VAKSE-V------------S		-LGL--GR-----T---IL----R
	Mycobacterium montefiorense	GBG40606	S-VAK---V----Y-------S		-LGL--GRA--A-T---IL----R
	Mycobacterium nebraskense	WP_046185623	-----G--V----------ETI		--GVV-Q-A-S------RL--A-R
	Mycobacterium noviomagense	WP_083087044	----K---V------------V		--NQ--DTA-NA-----RIT---R
	Mycobacterium palustre	WP_085077586	----K----------------V		-LGL--A-AHE------RF--N-K
	Mycobacterium paraense	WP_085097520	----KQG--------------V		--VLVG--A-EA-S---RF----R
	Mycobacterium paraffinicum	WP_073871019	S----A--V----------EPI		--HVIGQ-A-S------RL----R
	Mycobacterium paraseoulense	WP_083172469	-----G-------------ETI		--GVI---A-S------RL----R
	Mycobacterium parmense	WP_085271815	----K-S--------------V		-LGL--Q---T-Y----RI----R
	Mycobacterium persicum	WP_075546960	----K---V----------A-V		E-QL----AGA------RL----R
	Mycobacterium pseudoshottsii J	BBA86660	-G--K----------------V		-LGL-GDRA-NA----HRV----R
	Mycobacterium riyadhense	WP_085250406	----H---V------------V		--GLV--RASA-----HRL----K
	Mycobacterium salmoniphilum	WP_078330540	G-V-H---E------------V		--DVY--VT-N-LAS--RM--A-K
	Mycobacterium saskatchewanense	WP_085258683	----K-E--------------F		-LGQ---RA--------RL----K
	Mycobacterium scrofulaceum	WP_067282346	S-------V----------EPI		-AGVIGQ-A-S------RL----R
	Mycobacterium sherrisii	WP_085168007	--VAKSE-V------------S		-LGL--GR-----T---IL--T-R
	Mycobacterium shigaense	WP_096437189	--V-K-Q-V------------V		--VL--DRA----A---RF--T-R
	Mycobacterium shinjukuense	WP_085157202	----KS--V-----------RA		---L---RA--------RL----
	Mycobacterium simiae	WP_044509708	--VAKSE-V------------S		-LGL--GR-----T---IL----R
	Mycobacterium szulgai	WP_085670065	----K-S--------------V		---L---R-HN--G---RL----T
	Mycobacterium triplex	WP_036472085	V-S-KA--V------------S		--GL--GRA----QV--IL--T-R
	Mycobacterium ulcerans	WP_071497893	-G--K----------------V		-LGL-GDRA-NA----HRV----R
	Mycobacterium vulneris	WP_085288067	---------------------V		--AT-TV-TNAAL-V--QL--A-R
	Mycobacterium xenopi	WP_081485287	----K---V------------V		-AGL---MA-NA-----RIT-A-R

Figure S31. Partial sequence alignment of a conserved protein showing a three amino acid insertion that is specific for members of the “Tuberculosis” clade.

Clade	Species	Accession	109		159
"Tuberculosis" clade (11/11)	*Mycobacterium tuberculosis*	CFR63098	LDATDRARLGDLHAVTLHRLLGAKPG		ARFRQDRQNRLPHNVIVVDETSMVS
	Mycobacterium africanum K85	EFD42240	--------------------------		-------------------------
	Mycobacterium bovis	WP_024457966	--------------------------		-------------------------
	Mycobacterium bovis AF2122/97	YP_009357978	--------------------------		-------------------------
	Mycobacterium canettii	WP_014000331	--------------------------		-------------------------
	Mycobacterium caprae	APU24819	--------------------------		-------------------------
	Mycobacterium microti	AMC58129	--------------------------		-------------------------
	Mycobacterium mungi	OAQ17243	--------------------------		-------------------------
	Mycobacterium orygis	WP_003403250	--------------------------		-------------------------
	Mycobacterium pinnipedii	PRH91813	--------------------------		-------------------------
	Mycobacterium africanum	WP_031669274	--------------------------		-------------------------
Other *Mycobacteriaceae* (0/>100)	*Mycobacterium alsense*	OQZ93823	---A------G-E-M-------RR-D	SS	S---H--G-----D-----------
	Mycobacterium angelicum	WP_083112899	-H-A-Q----P-P---------R--D	TS	----H--S-----D-----------
	Mycobacterium aquaticum	WP_083161823	--LV--R--AG-Q-T-------PR-D	TS	S---HH-G-----D-----------
	Mycobacterium asiaticum	OBK14580	--PV--Q-VSG-R-T-------SR-D	TS	S---HH-G-----D-----------
	Mycobacterium bohemicum	WP_085179261	--PG--EC-AG-Q---------SR-D	SS	V---HH-G-----D-----------
	Mycobacterium branderi	WP_083130215	-PDI--D--TG-Q---------SR-D	TS	V--KHN-G-----A-----------
	Mycobacterium conspicuum	WP_085233363	-APD-A--VAG-R-T-------PR-D	TS	V--KHN-G-----D-----------
	Mycobacterium dioxanotrophicus	WP_087074514	--LV--R--AG-Q-T-------PR-D	TS	S---HH-G-----D-----------
	Mycobacterium europaeum	WP_085240572	-EPP--R--AG-E---------SR-D	TS	V---HH-A-----D-----------
	Mycobacterium gastri	WP_036408766	---A--M---Q-Q---------SR-D	TS	S---HH-G-----D-----------
	Mycobacterium gordonae	OBK59743	--PV--Q-VSG-R-T-------SR-D	TS	S---HH-G-----D-----------
	Mycobacterium grossiae	WP_070351321	--PG-AQ-ISG-Q-T-------SR-D	SS	S---HH-G-----D-V---------
	Mycobacterium haemophilum	KLO33035	---G--E--S------------SR-D	TS	T---HH-G-----D-----------
	Mycobacterium haemophilum DSM	AKN18209	---G--E--S------------SR-D	TS	T---HH-G-----D-----------
	Mycobacterium hassiacum	WP_018354877	-P-A-QQA-AG-Q-T-------SR-D	TS	----HH-G-----E-----------
	Mycobacterium hassiacum DSM 44	EKF24987	-P-A-QQA-AG-Q-T-------SR-D	TS	----HH-G-----E-----------
	Mycobacterium heckeshornense	WP_048890320	---A------E-P-M-------PR--	TS	S--KH--G-----D-----------
	Mycobacterium heidelbergense	WP_083074006	-S-A--E---E-R---------P---	TS	----H--G-----------------
	Mycobacterium holsaticum	WP_084223007	-S-V---A-AG-Q-T-------SR-D	TS	----HN-A-----E-----------
	Mycobacterium insubricum	ORA65210	--DA--D--PE-R---------VR-D	SS	V---HH-G-----D-----------
	Mycobacterium intermedium	WP_069421482	--VA------P-E---------S---	TS	----H--A-----D-----------
	Mycobacterium iranicum	WP_064284762	-ADV-Q---SG-R-T-------SR-D	TS	S---HH-G-----D-V---------
	Mycobacterium kansasii	ORB85511	---A--L-V-Q-Q---------SR-D	TS	S---H--G-----D-----------
	Mycobacterium komaniense	WP_090276792	---A--EA-SG---T-------SR-D	TS	----HN-A-----D-----------
	Mycobacterium kubicae	WP_085072868	--QA--Q-VSG-R-T-------SR-D	TS	S---HH-G-----D-----------
	Mycobacterium kyorinense	WP_065013563	-NES--V-----Q-M-------SR-D	TS	S--KH--G-----D-----------
	Mycobacterium lacus	WP_085157428	---G--Q---E-----------SR-D	TS	----H--A-----D-----------
	Mycobacterium lehmannii	WP_094289790	---A--QA-SG---T-------SR-D	TS	----HN-Y-----D-----------
	Mycobacterium liflandii	WP_015354635	---I-QQ-VRG-Q-T-------SR-D	TS	S---HH-G-----D---I-------
	Mycobacterium litorale	AQT82457	-S-V--E--QG---T--------RHD	TS	S---HH-E-----D-----------
	Mycobacterium llatzerense	WP_052506286	---A-QE--SG-R---------GR-D	SA	S--KHH-G-----D-----------
	Mycobacterium mageritense	WP_036429256	--LI--R--AG-Q-T--------R-D	TS	S---HH-A-----D-----------
	Mycobacterium malmesburyense	WP_090343739	---A--EA-SG---T-------SR-D	TS	----HN-A-----D-----------
	Mycobacterium malmoense	WP_071513547	-S-A--E---E-R---------P---	TS	----H--G-----------------
	Mycobacterium marinum	WP_020731883	---V-QQ-VRG-Q-T-------SR-D	TS	S---HH-G-----D---I-------
	Mycobacterium monacense	WP_083044910	---V--D--PE-T-S------QRRRD	NA	S---HH-E-----D-----------
	Mycobacterium moriokaense	WP_083156994	--PV--D--PE-K-S------RSR-D	NS	S---HH-E-----D-----------
	Mycobacterium mucogenicum	WP_064857872	--SA--E--SG-R---------GR-D	SA	S--KHH-G-----D-V---------
	Mycobacterium neoaurum	CDQ46973	--PQ-----QG-T-T--------R-D	TS	S---HH-G-----D-----------
	Mycobacterium neumannii	WP_094293426	--S---QA-SG---T-------SR-D	TS	----HN-H-----D-----------
	Mycobacterium neworleansense	WP_090515180	---V--E-ISG-Q-T-------PR-D	TS	S---HH-A-----D---I-------
	Mycobacterium noviomagense	WP_083088299	--SA-QT---E-Q-M-V----HMR--	SS	S---HH-G-----D-----------
	Mycobacterium novocastrense	WP_067392028	-ETA--EA-SG---T-------SR-D	TS	----HN-A-----D-----------
	Mycobacterium obuense	WP_046361067	---Q--D--PA-T-S------RPR-D	SG	S---HH-E-----D-----------
	Mycobacterium palustre	WP_085081053	-EDV--Q--SG-Q---------SR-D	TS	V---HH-G-----D-----------
	Mycobacterium parafortuitum	WP_083145991	-TEV--G--SG-R-T-------SR--	TS	S---HH-G-----D-----------
	Mycobacterium paraseoulense	WP_083171635	-QPR--E--AG-E---------SR-D	TS	V---HH-A-----D-----------
	Mycobacterium peregrinum	WP_064886359	---V--E-ISG-Q-T-------SR-D	TS	S---HH-A-----D-----------
	Mycobacterium phlei	WP_061482629	-PEA---A-SGM--T-------SR-D	TS	----HH-G-----D-----------
	Mycobacterium porcinum	WP_069426551	---V--E-ISG-Q-T-------PR-D	TS	S---HH-A-----D-----------
	Mycobacterium rhodesiae	ORB56199	-TPA--D--TG-R-T-------PL-R	TS	----HH-E-----D-----------
	Mycobacterium riyadhense	WP_085249176	---V--V---P-Q-L-------RR-D	TS	----H--A-----D-----------
	Mycobacterium rufum	KGI66926	---Q--D--PE-T-S------RPR-D	NA	T---HH-E-----D-----------
	Mycobacterium rutilum	WP_083405530	---A--EA-SGM--T-------SR-D	TS	----HH-G-----D-----------
	Mycobacterium saskatchewanense	WP_085258140	-AQA--E---R-Q---------SR--	SS	T---H--A-----D-----------
	Mycobacterium septicum	WP_044515982	---V--E-ISG-Q-T-------SR-D	TS	S---HH-A-----D-----------
	Mycobacterium setense	WP_064871624	---V--E-ISG-Q-T-------PR-D	TS	S---HH-G-----D-----------
	Mycobacterium sherrisii	WP_069402775	-S-A-QQA-SG-Q-T-------SR-D	TS	----HN-A-----D-----------
	Mycobacterium shimoidei	WP_069395722	-G-A--T---E-R-M-------SR-D	TS	S---H--G-----D-----------
	Mycobacterium shinjukuense	WP_083050879	--DA------EP----------P---	TS	----H--R-----D-----------
	Mycobacterium smegmatis	WP_003892718	--LV--R--TG---T-------PR-D	TS	S---HH-A-----D-----------
	Mycobacterium sphagni	WP_094480564	--PV--D--QG-R-T-------PL-R	TS	----HH-G-----D-----------

Other *Mycobacteriaceae* (0/>100)

Mycobacterium szulgai	OBF24477	--QA--Q-VSG-R-T-------SR-D TS S---HH-G-----D----------
Mycobacterium thermoresistibile	WP_003925971	--LV--R--SG-Q-T-------PR-D SS S---HH-A-----D----------
Mycobacterium tusciae	WP_006246889	---V--D--PK-K-S------RTR-- NS S---HH-E-----D----------
Mycobacterium ulcerans	OIN17199	---I-QQ-VRG-Q-T-------SR-D TS S---HH-G-----D---I------
Mycobacterium vaccae	WP_003929667	--PV--T--AG-R-T-------SR-- TS S---HH-D-----D----------
Mycobacterium vanbaalenii	WP_011778477	-P-V-QG--AG-R-T-------SR-D TS S---HH-D-----D----------
Mycobacterium vulneris	WP_065461665	---V--E-ISG-Q-T-------PR-D TS S---HH-A-----D----------
Mycobacterium wolinskyi	WP_085146452	---V--S--SG-Q-T-------PR-D TS S---HH-A-----D----------
Mycobacterium xenopi	WP_003921185	---A------E-Q-M-------SR-- TS S--KH--G-----D----------
Mycobacteroides abscessus	WP_062878541	---V--S--AG-Q-T-------SR-D TS S--KH--G-----D----------
Mycobacteroides abscessus 6G-0	EIU51479	---V--S--AG-Q-T-------SR-D TS S--KH--G-----D----------
*Mycobacteroides abscessus MAB_*	ETZ95059	---V--S--AG-Q-T-------SR-D TS S--KHH-G-----D----------
Mycobacteroides abscessus subsp. bolletii	SIN42964	---V--S--AG-Q-T-------SR-D TS S--KHH-G-----D----------
Mycobacteroides franklinii	WP_070937364	--PV-QS--AG-Q-T-------SR-D TS S---HH-G-----D----------
Mycobacteroides immunogenum	WP_043078480	---V--S--AG-Q-T-------SR-D TS S--KH--G-----D----------
Mycolicibacillus koreensis	WP_085301948	-S-A--D--AG-Q-T-V-A---WR-- SS V---H--T----YD---I------
Mycolicibacillus trivialis	ODR08096	-S-A--D--AG-Q-T-V-A---WR-- SS V---H--T----YD---I------
Mycolicibacter algericus	WP_083037328	-EPK-Q---AG-Q-S---S---SR-- NS ---KHN-G-----D----------
Mycolicibacter arupensis	WP_046187853	--PV-----AG-G-T---T---SR-D TS V---H--A-----D----------
Mycolicibacter heraklionensis	OBG40431	-EPV-Q---AG-G-T---T---SR-D SS V---H--A-----D----------
Mycolicibacter kumamotonensis	WP_084014048	--PV-Q---AG-Q-S---S---SR-R NS S--KHN-A-----D-V--------
Mycolicibacter longobardus	ORW08855	--PV-Q---AG-Q-S---S---SR-- NS S--KHN-G-----D----------
Mycolicibacter minnesotensis	WP_083022762	-EPV-Q---AG-G-T---T---SR-D SS V---H--A-----D----------
Mycolicibacter senuensis	ORW66931	-EPV-Q---AG-Q-S---S---SR-- NS ---KHN-G-----D----------
Mycolicibacter sinensis	WP_013827584	-EPK-Q---AG-Q-S---S---SR-- NS ---KHN-G-----D----------
Mycolicibacter virginiensis	WP_105294735	-EPV-Q---AG-G-T---T---SR-D SS V---H--A-----D----------
Mycolicibacterium agri	WP_097939130	--EA-QRA-AE---M-------SR-D TS ----HN-G-----D----------
Mycolicibacterium aromaticivorans	WP_036342431	-T-A--D--QG---T-------PV-R TS ----HH-E-----D----------
Mycolicibacterium aurum	WP_087031251	--EQ-----TG-Q-T--------R-D TS S---HH-G-----D----------
Mycolicibacterium austroafricanum	WP_036370219	-P-V-QG--AG-R-T-------SR-D TS S---HH-D-----D----------
Mycolicibacterium bacteremicum	WP_083055450	-GP---G--TG-Q-T-------SR-D TS S---HH-G-----D----------
Mycolicibacterium boenickei	WP_097926231	---V--E-ISG-Q-T-------PR-D TS S---HH-A-----D----------
Mycolicibacterium brisbanense	WP_062829260	--LV--R--AG-Q-T-------PR-D TS S---HH-G-----D----------
Mycolicibacterium brumae	WP_090587927	--PV-----P-PQ---------FR-- SA T--AHH-G-----D-V--------
Mycolicibacterium canariasense	WP_062658133	--EV-----SG-S-T-V-----PR-D TS S---HH-G-----D------A---
Mycolicibacterium celeriflavum	ORA45241	-P-A--EA-AG---T-M-----SR-D TS ----HH-G-----D----------
Mycolicibacterium chlorophenolicum	WP_048471748	---Q--D--PE-T-S------RPR-D NA S---HH-E-----D----------
Mycolicibacterium chubuense	WP_014814166	---A-----PE-S-S-V----RPR-D NS S---HH-E-----D----------
Mycolicibacterium conceptionense	WP_085142680	---V--E-ISG-Q-T-------PR-D TS S---HH-A-----D----------
Mycolicibacterium confluentis	ORV31237	-GDS-----QG-Q-L-------S--D SS T---HH-G-----DLV--------
Mycolicibacterium cosmeticum	WP_036401504	--EV--T---G-S-T-------PR-D TS S---HH-G-----D------A---
Mycolicibacterium diernhoferi	WP_073856988	--PQ-----AG-R-T--------R-D TS S---HH-G-----D----------
Mycolicibacterium duvalii	WP_098002401	---E--D---E-T-S------QPR-- SS S---HH-E-----D-V--------
Mycolicibacterium elephantis	WP_046751223	-S-A--SA-EG-Q-T-------SR-D TS ----HN-A-----D----------
Mycolicibacterium fallax	ORV00089	-S-A--D--PE-----I-----FR-- SA T---HH-G-----D-V--------
Mycolicibacterium farcinogenes	WP_036389134	---V--E-ISG-Q-T-------PR-D TS S---HH-A-----D----------
Mycolicibacterium flavescens	WP_069413236	-G-A--EA-SGM--T-M-----SL-E TS ----HH-G-----D----------
Mycolicibacterium fortuitum	WP_061262392	---V--D-ISG-Q-T-------SR-D TS S---HH-G-----D----------
Mycolicibacterium goodii	WP_049748265	-ELV--R--TG-Q-T-------PR-D TS S---HH-A-----D----------

Figure S32. Partial sequence alignment of the exonuclease V subunit alpha protein showing a two amino acid deletion that is specific for members of the “Tuberculosis” clade.

Group	Species	Accession	320		366
"Tuberculosis" clade (9/9)	*Mycobacterium tuberculosis*	AMC71919	KVALTPESGAGRGAAVDPSSLPRQTN		FAAQLLHDLSHAYAVVFVIAT
	Mycobacterium bovis	WP_044798679	--------------------------		---------------------
	Mycobacterium canettii	WP_014000415	--------A----V-----------S		---------------------
	Mycobacterium caprae	APU24969	--------------------------		---------------------
	Mycobacterium microti	AMC58301	--------------------------		---------------------
	Mycobacterium mungi	OAQ17950	--------------------------		---------------------
	Mycobacterium orygis	EMT36996	--------------------------		---------------------
	Mycobacterium pinnipedii	WP_105826372	--------------------------		---------------------
	Mycobacterium africanum	EFD42411	--------------------------		---------------------
Other *Mycobacteriaceae* (0/35)	*Mycobacterium alsense*	WP_083136683	EL-TLQQKA-AT-VP----AV-PR-L	APG	-SGG---------TT----GV
	Mycobacterium angelicum	WP_083113736	-I-VLQ-DS-R--VP-----M-ARAL	DPE	-PGN-MR------TA--LV-V
	Mycobacterium asiaticum	OBI81546	EL-RLEQNA-R--VP----AI-QRAL	DPG	---S-A-------TLILA--V
	Mycobacterium avium	WP_033706144	-L-ALHQKA-AG-TPI-Q-AI---SL	APG	-WGNV--------TA-----V
	Mycobacterium avium 05-4293	ETA91338	-L-ALHQKA-AG-TPI-Q-AI---SL	APG	-WGNV--------TA-----V
	Mycobacterium avium 09-5983	ETB22503	-L-ALHQKA-AG-TPI-Q-AI---SL	APG	-WGNV--------TA-----V
	Mycobacterium avium 10-5560	ETB50414	-L-ALHQKA-AG-TPI-Q-AI---SL	APG	-WGNV--------TA-----V
	Mycobacterium avium 104	ABK65775	-L-ALHQKA-AG-TPI-Q-AI---SL	APG	-GGNV--------TA-----V
	Mycobacterium avium MAV_061107	ETZ46800	-L-ALHQKA-AG-TPI-Q-AI---SL	APG	-WGNV--------TA-----V
	Mycobacterium avium MAV_120809	ETZ34458	-L-ALHQKA-AG-TPI-Q-AI---SL	APG	-GGNV--------TA-----V
	Mycobacterium avium subsp. hominissuis	ETB27073	-L-ALHQKA-AG-TPI-Q-AI---SL	APG	-WGSV--------TA-----V
	Mycobacterium avium subsp. paratuberculosis	AAS02936	-L-ALHQKA-AG-TPI-Q-AI---SL	APG	-WGNV--------TA-----V
	Mycobacterium bohemicum	WP_085182632	-L-ALQHKA-VS-LPI--AAV--RSL	APG	-P-EV-----R--TT-----A
	Mycobacterium bohemicum DSM 44	CPR11316	-L-ALQHKA-VS-LPI--AAV--RSL	APG	-P-EV-----R--TT-----A
	Mycobacterium conspicuum	ORV38176	AL-TLQQNA-A--VP-N-AAI----L	SPD	--EH------R--TA---L-V
	Mycobacterium genavense	WP_036468039	-L-ALQQQA-MT-VPI---QI-H-SL	DPN	-S-D---------TS---V-V
	Mycobacterium gordonae	OBJ79192	EL-RRQQEA-R--VPL---AI-PR-L	GPD	-TTN---------T--ML--V
	Mycobacterium haemophilum	WP_082129340	-L--AEHNAVR--VP----AI-G-AL	TPD	--GSV---------T--AL-V
	Mycobacterium heidelbergense	WP_083072278	TLVSLQQKA-AS-LP--R-AI---SL	VPG	-SGNV--------TA-----V
	Mycobacterium interjectum	ORV86968	-L-TLQQKA-AT-MP----AV-P--L	APG	-SGG---------TL-----V
	Mycobacterium kansasii	OOK70496	-I-ALQQQAVRQ-V-----AI-Q-AL	AHD	--SRV--------T------V
	Mycobacterium kansasii 662	EUA10371	-I-ALQQQAVRQ-V-----AI-Q-AL	AHD	--SRV--------T------V
	Mycobacterium kansasii 824	ETZ97903	-I-ALQQQAVRQ-V-----AI-Q-AL	AHD	--SRV--------T------V
	Mycobacterium kansasii ATCC 12	AGZ53804	-I-DLQ-DA-R--VP-----I-P-AL	NPD	-KGNV--------S---AA-V
	Mycobacterium kubicae	ORV97272	-L--LQQGA-R--IPL--N-I-A--L	APD	-N-KV----C-S-T--L-LGC
	Mycobacterium lacus	WP_085159263	--GFLQQNA-RT-VP----A----AL	APD	--VHV-----------ML-V
	Mycobacterium malmoense	OIN80796	-L-SLQQKA-SS-VP--Q-AI---SL	APS	-W-DV--------TA--A--V
	Mycobacterium nebraskense	WP_082147484	---SLR-DADK--LP----AM---AL	SPD	-TSHVM-N---V-TL---L-V
	Mycobacterium paraense	ORW30813	-L-TLQQKA-AT-VP----AV-P--L	APG	-SGSV--------TL-----V
	Mycobacterium parmense	ORW59825	-I-ALKQRA-EGAVS--G-AM--ESM	APG	--GNVI-------TA--A--V
	Mycobacterium riyadhense	WP_085249617	Q--ALQHDA-R--VPI---AI--R-L	TPD	--GNV----A---T-----GV
	Mycobacterium sherrisii	ODR04013	-L-ALQQKA-IS-EP----QI-PE-L	APG	-S-N---------TA---VSA
	Mycobacterium shinjukuense	WP_083046396	-R--VP--V-QM-G-AR	TPD	--VN------R--
	Mycobacterium szulgai	OBF21900	-L--LQQGA-R--IP---N-I-A--L	APD	-N-KV----C-S-T--L-LGC
	Mycobacterium triplex	WP_036473104	-L-ALQQQA-IT-VP----QI-H-SL	DPN	-S-D---------TS---V-V

Figure S33. Partial sequence alignment of the multidrug resistance protein EmrB showing a three amino acid deletion that is specific for members of the "Tuberculosis" clade.

Clade	Organism	Accession	39		91
"Tuberculosis" clade (9/9)	*Mycobacterium tuberculosis*	CKR96409	NLQNHEVTAIVHGSSDPHRIGVAAERSL		IRDRIRFDDFATFVDNAVSAATELF
	Mycobacterium bovis	WP_099171347	----------------------------		-------------------------
	Mycobacterium canettii	WP_014000421	----------------------------		-------------------------
	Mycobacterium caprae	CEJ50081	----------------------------		-------------------------
	Mycobacterium microti	AMC58309	----------------------------		-------------------------
	Mycobacterium mungi	WP_003403998	----------------------------		-------------------------
	Mycobacterium orygis	EMT37003	----------------------------		-------------------------
	Mycobacterium pinnipedii	PRH91966	----------------------------		-------------------------
	Mycobacterium africanum	CCC25873	----------------------------		-------------------------
Other *Mycobacteriaceae* (0/53)	*Mycobacterium arosiense*	WP_083064922	-RLDRH-----------Q----V-RK-Q	W	V-NDVS-----S---RT-AD-----
	Mycobacterium asiaticum	WP_065159289	-E-SDD-----------R-------Q-R	W	-EA--T-AE----T-RVTA--S---
	Mycobacterium avium	WP_003875788	-RLGRR-----------Q----T-RK-Q	W	--NGVG-T---A-L-ETIAD--Q--
	Mycobacterium avium 104	ABK65322	-RLGRR-----------Q----T-RK-Q	W	--NGVG-T---A-L-ETIAD--Q--
	Mycobacterium avium XTB13-223	KBR64903	-RLGRR-----------Q----T-RK-Q	W	--NGVG-T---A-L-ETIAD--Q--
	Mycobacterium avium subsp. avium	ETB14289	-RLGRR-----------Q----T-RK-Q	W	--NGVG-T---A-L-ETIAD--Q--
	Mycobacterium avium subsp. hominissuis	BAN29719	-RLGRR-----------Q----T-RK-Q	W	--NGVG-T---A-L-ETIAD--Q--
	Mycobacterium avium subsp. silvaticum	ETB07290	-RLGRR-----------Q----T-RK-Q	W	--NGVG-T---A-L-ETIAD--Q--
	Mycobacterium bohemicum	WP_085182626	-RRDRA---V--D----R------RS-Q	S	-P-GLC-EG--S-A-GTIAD-IDF-
	Mycobacterium colombiense	WP_007772616	-RLDR------------Q----T-RK-Q	W	V--DVS-N---A---RT-AD-----
	Mycobacterium conspicuum	WP_085234712	-Q--RS-----------Q-V----RG-Q	W	--EGMSLE---A---RTLT--IDF-
	Mycobacterium europaeum	WP_085243013	-RDE----------C--Q----V-R--H	W	--RNTS-EE--A---RTIAD--D--
	Mycobacterium florentinum	WP_085223566	-SRSRD-LVT-R-----Q-V--V-RS-R	K	--EPVA------YI-HSIA--I-F-
	Mycobacterium gastri	WP_084293502	-TR--A-----------Q-V--T-CN-Q	R	-PGLV--NE--DAI-R-LD--VD--
	Mycobacterium gastri 'Wayne'	ETW24487	-TR--A-----------Q-V--T-CN-Q	R	-PGLV--NE--DAI-R-LD--VD--
	Mycobacterium genavense	WP_025736905	-RRSRD-VVT-R-----Q-V--V-RS-R	K	--EPVD--E---YI-HTIA--I-F-
	Mycobacterium gordonae	OBS00484	-E-HR----V-------Q-------Q-R	W	-GE-V----L-A-A-R-TA--ID--
	Mycobacterium haemophilum	WP_082129372	-QH-R------------Q----D-RS-Q	R	--A----E---S-L-R-LV--VD--
	Mycobacterium interjectum	WP_066910927	-QK-RG-----R--A-SE---LV-R--Q	W	-AEG-A-ER--S---RTIAE-VD--
	Mycobacterium intermedium	WP_069419786	-REHR-I--D--R----L-----GA--Q	R	MGG-V--E--TA---E-IG--V---
	Mycobacterium intracellulare	WP_014379089	-R-DRH--------C--Q----V-RK-Q	W	--SKVS-AE--SL--LTIAD-----
	Mycobacterium intracellulare M	ETZ39401	-R-DRH--------C--Q----V-RK-Q	W	--SKVS-AE--SL--LTIAD-----
	Mycobacterium kansasii	KZS63551	-AR--A-----------Q-V--T-CN-Q	W	-PGPVW-NE--DN--R-LD--VD--
	Mycobacterium kansasii 732	EUA00975	-TR--A----M------Q-V--T-CN-Q	W	-PGLV--NE--DT--R-LD--VD--
	Mycobacterium kansasii ATCC 12	AGZ50746	-TR--A-----------Q-V--T-CN-Q	W	-PSLV--NE--DT--R-LD--VD--
	Mycobacterium kubicae	WP_085074999	-KH-G-LA-V-------Q----TVSA-H	W	VGE-V--E--GE---R-LG--V---
	Mycobacterium lacus	WP_085159265	-RR-SA-S----E----Q----I-SK-Q	S	-GGLV------G---RTLA--ID--
	Mycobacterium lentiflavum	WP_090606894	-RRSRD-VVT-------Q-V--L-RS-R	K	--EPLE---L--YI-HTIA--I-F-
	Mycobacterium lepraemurium	ATA27729	-RLGRR-----------Q--D-T-RK-Q	W	--NGVG-T---A-L-ETIAD-----
	Mycobacterium malmoense	WP_083009922	-Q--R------------Q----V-R--Q	W	---DVTL----R---GTIAD-ID--
	Mycobacterium mantenii	WP_083093928	-RLDRQ-----------R----M-RK-Q	W	--RD-S-G---S---RTIAD-----
	Mycobacterium marinum	WP_020730751	ST-RRG-------N---Q----T-YK-Q	W	-AGPVD-SE--V---R-IG-SI---
	Mycobacterium marseillense	WP_083014715	-R-DRH-----------Q----V-RK-Q	W	--SAVS-A---S---L-IAD-----
	Mycobacterium montefiorense	GBG36281	-HA-RD-VVT-R-----Q----V-RS-R	K	V-EPVA-EE---YI-HSIA--I-F-
	Mycobacterium nebraskense	WP_046184303	-HRER---------C--Q----V-R--Q	W	--PNVS-E---A---RTIAD--D--
	Mycobacterium palustre	WP_085078568	-S--R--V----N-T--Q---MI-R--H	W	---GVS-E---S---G-IAD-I---
	Mycobacterium paraense	WP_085095629	-A-DRG-V---------Q----V-R--Q	W	--KGVS-E---S---A-IAD-ID--
	Mycobacterium paraffinicum	WP_073871061	-R-DR------------Q----I-R--H	W	--RSVS-EE--A---RTIAD-----
	Mycobacterium parascrofulaceum	EFG77795	-H-DR------------Q----V-R--H	W	--QNVS-EA--A---RTIAD-----
	Mycobacterium paraseoulense	WP_083168081	-REE-----VM---C--Q----V-R--H	W	--RNTS-EE--A---RTIDD--KF-
	Mycobacterium parmense	WP_085269545	-H--RG---L--VW---P---IS-R--R	W	VP-GLS--E--S-A-GSIAD-I-F-
	Mycobacterium riyadhense	ORW84112	-AR--A-----------R-VVIE-QQCQ	W	LSEP---E---DSA-RTITT-ID--
	Mycobacterium saskatchewanense	WP_085255725	-CG-R----L--D-T--Q---LL-R--Q	W	--GGGS-E---S---AVIAD-ID--
	Mycobacterium scrofulaceum	WP_067269440	-R-DR------------Q----V-R--H	W	--RNVS-----A---RTIAD--D--
	Mycobacterium sherrisii	WP_069401920	-HDHRD-I-T-------Q----V-RN-R	R	--EP-E--E---H--HSIAE-I-F-
	Mycobacterium shigaense	WP_096442961	-RH-RDLVVT-RR----K----L-RSTR	R	V-GQVD--E------HGIA--I-F-
	Mycobacterium shinjukuense	WP_083048132	-G--R--M------A---------HAYR	R	FGTHVC-----GR--R-IG--I---
	Mycobacterium simiae	WP_061557220	-HKSRA-V-T-------R-V--V-RT-R	R	V-EP-E--E---H--HSIA--I-F-
	Mycobacterium szulgai	ORX19146	-ERRRDI----------R----E-QQCR	W	FTQEVT-EE--E---RTLT--IG--
	Mycobacterium triplex	CDO90944	-RRSRD-VVT-R-----Q-V--V-RS-R	K	--EPVD-E----YI-HTIA--I-F-
	Mycobacterium ulcerans	WP_011738744	-T-RRG-------N---Q----T-YK-Q	W	-AGPVD-SE--V---R-IG-SI---
	Mycobacterium ulcerans str. Harvey	EUA92729	-T-RRG-------N---Q----T-YK-Q	W	-AGPVD-SE--V---R-IG-SI---
	Mycobacterium vulneris	WP_085288923	-RLDR------------Q----T-RK-Q	W	--NDVS-N---S---R--AD-----

Figure S34. Partial sequence alignment of the Hypothetical protein ERS024213_05484 showing a one amino acid deletion that is specific for members of the "Tuberculosis" clade.

Group	Species	Accession	290		328
“Tuberculosis” clade (10/10)	*Mycobacterium tuberculosis*	AIH29714	RYRLLETVRRYALEKLGDSGEAD		VHARHRDYYTALAASL
	Mycobacterium africanum MAL010	KBG16995	-----------------------		----------------
	Mycobacterium bovis	WP_079367612	-----------------------		----------------
	Mycobacterium bovis AF2122/97	YP_009358247	-----------------------		----------------
	Mycobacterium bovis BCG	AMC49560	-----------------------		----------------
	Mycobacterium canettii	WP_014000486	-----------------------		----------------
	Mycobacterium canettii CIPT 14	CCK50803	-----------------------		----------------
	Mycobacterium microti	AMC58417	-----------------------		----------------
	Mycobacterium orygis 112400015	EMT36789	-----------------------		----------------
	Mycobacterium africanum	WP_013988782	-----------------------		----------------
Other *Mycobacteriaceae* (0/64)	*Mycobacterium alsense*	WP_083137383	---------Q--Q----------	E	-RT------ASR-TH-
	Mycobacterium angelicum	WP_083114901	---------Q-------E-----	E	-RT--------T-VE-
	Mycobacterium aquaticum	WP_095533761	----M--I-Q--Q----E-----	Q	-RD-------GTVE--
	Mycobacterium asiaticum	OBI79507	---------Q-------E-R-G-	D	-RG----H---I-SR-
	Mycobacterium avium subsp. avium	EUA25771	---------Q--Q----E-----	D	-RT--------
	Mycobacterium bohemicum DSM 44	CPR13161	---------Q-------E-----	T	-R---S----LM-SQ-
	Mycobacterium branderi	WP_083132161	------M--Q-S----AE-----	A	-RI----H------T-
	Mycobacterium caprae	APU26456	---M-----Q------RE--D--	A	-R-----H-A-V--G-
	Mycobacterium celatum	ORV15423	---------Q--Q---LE-----	G	-RT----H---I--P-
	Mycobacterium chimaera	ARV84894	---M-----Q-------E-----	A	-R-----H---M--L-
	Mycobacterium colombiense	WP_081396250	---------Q-------E-----	V	-R-----H---M-TQ-
	Mycobacterium florentinum	ORV50168	-------I-Q--QS---A---G-	T	--G------L-M-TV-
	Mycobacterium fragae	WP_085194147	---------Q-------E-----	A	-RS-------SM--AV
	Mycobacterium gordonae	WP_055581268	---V-----Q-G-----E----G	T	-R-----F-ITT--A-
	Mycobacterium heidelbergense	WP_083073662	---------Q--Q----E-----	E	-RT--------T--E-
	Mycobacterium interjectum	ORV94581	---------Q-------E---S-	A	-RT----H--H--VV-
	Mycobacterium intermedium	ODQ97972	---------Q-------E-----	A	IR-------ESM--Q-
	Mycobacterium intracellulare	OBH38896	---------Q--Q----E-----	E	-RT-------TT-ID-
	Mycobacterium kansasii	KZS65551	---------Q------SE-D---	A	IR--------SM--L-
	Mycobacterium komaniense	WP_090274073	---------Q---D---E-----	T	-RG----H--SMT-Q-
	Mycobacterium kyorinense	WP_065015729	---------Q-------E-----	A	-RS--C----SM--L-
	Mycobacterium lacus	WP_085156368	---C--M-E------KQA--V-	L	-R-----H---M--L-
	Mycobacterium lentiflavum	CQD24068	---------Q-------E-----	A	-R-----H------L-
	Mycobacterium llatzerense	WP_082067987	-------M-Q-------E----A	A	-R-----H---M-MR-
	Mycobacterium malmoense	WP_083212447	---------Q-G----AE----E	A	-RI----H---V--R-
	Mycobacterium marinum	BBC68212	---I-----Q------AE-----	S	-RT----H--TM--R-
	Mycobacterium marseillense	WP_095576485	---M-----Q------SE-S---	S	-R-----H---M--L-
	Mycobacterium microti	AMC60182	---------Q------RE--D--	A	-R-----H-A-V--G-
	Mycobacterium montefiorense	GBG35888	---------Q--Q----E-----	A	-RS-------SI-VAV
	Mycobacterium mucogenicum	OBJ39938	---------Q-------E-----	D	-RV--------I-MR-
	Mycobacterium neumannii	WP_094293510	---------Q---D---E-----	T	-RS----H--SMT-R-
	Mycobacterium noviomagense	WP_083088937	-FL------Q--Q---VE-----	T	-RT--------M--P-
	Mycobacterium novocastrense	WP_067389187	---------Q---D---E-----	T	-RG----H-NSMT-Q-
	Mycobacterium paraense	ORW40175	---------Q-------E-----	T	-R------F-LM-SQ-
	Mycobacterium parascrofulaceum	WP_085981628	---------Q--Q----E---S-	E	-RT------V-K--E-
	Mycobacterium paraseoulense	WP_083166763	---------Q--Q----E---G-	N	-RT--------TI-E-
	Mycobacterium parmense	WP_085270575	---------Q-------E-----	A	-RS----H------
	Mycobacterium scrofulaceum	OBH94271	---------Q-------E---S-	E	-RT--------T-VL-
	Mycobacterium shigaense	BAX93575	----M----Q-------E-----	H	-RD----H--G-V-D-
	Mycobacterium shimoidei	ODR13073	------M--Q------SE-----	A	-R-----H---MG-L-
	Mycobacterium shinjukuense	WP_083046925	------M-E----R-DEA--V-	G	-R-----H------L-
	Mycobacterium szulgai	OBF15989	---M--M--Q------AE-----	A	-RT----H------A-
	Mycobacterium triplex	WP_084163278	---------Q-------------	T	-R---------M-TR-
	Mycobacterium tusciae	WP_006246964	---------Q-------E-----	S	TRD-------SM--L-
	Mycobacterium vanbaalenii	WP_041307284	---------Q--Q----E-----	T	LR-----H---R--E-
	Mycobacteroides abscessus subsp. abscessus	SHW23433	-------M-Q-------E-----	T	IRS------A-MVSQ-
	Mycobacteroides franklinii	WP_070936487	----P----Q-------E----E	T	-RS----H---V-TL-
	Mycolicibacter arupensis	WP_046189249	---M--M--Q-------E-----	T	IR-----H--LM-TA-
	Mycolicibacter heraklionensis	OBG31410	---M--M--Q--M----E-----	A	IR-----H---M--A-
	Mycolicibacter icosiumassiliensis	WP_078058768	---M--M--Q-------E-----	A	IR-----H---M--A-
	Mycolicibacter sinensis	OBG04837	---M--M--Q-------E-----	D	-RG----H------I-
	Mycolicibacter terrae	WP_085261241	------M--Q---G--AE-----	A	-RT----H---I--G-
	Mycolicibacter virginiensis	WP_105295293	---M--M--Q--M----E-----	A	IR-----H---M--A-
	Mycolicibacterium austroafricanum	WP_105387600	---------Q--Q----E-----	T	LR-----H---R--E-
	Mycolicibacterium chlorophenolicum	WP_082168977	---M---M-Q-------E--HG-	D	-R-----HF-G---V-
	Mycolicibacterium flavescens	WP_069416459	---------Q-------E-----	D	-RT----F--VT---

Figure S35. Partial sequence alignment of the LuxR family transcriptional regulator protein showing a one amino acid deletion that is specific for members of the “Tuberculosis” clade.

Group	Species	Accession	205		251
	Mycobacterium tuberculosis	AUS50074	IRRVARLGRMIGAAFEISRDIIAI		SGDSATLSGADLGQAVHTLPMLY
	Mycobacterium bovis	WP_046026275	------------------------		-----------------------
	Mycobacterium canettii	WP_044096121	------------------------		-----------------------
	Mycobacterium caprae	APU25148	------------------------		-----------------------
**“Tuberculosis” clade (9/9)**	*Mycobacterium microti*	AMC58531	------------------------		-----------------------
	Mycobacterium mungi	OAQ18283	------------------------		-----------------------
	Mycobacterium orygis	EMT36698	------------------------		-----------------------
	Mycobacterium pinnipedii	WP_105826514	------------------------		-----------------------
	Mycobacterium africanum	WP_031669304	------------------------		-----------------------
	Mycobacterium angelicum	WP_083115663	VE-LS---NIV-T--Q-AD---D-	DSE	-QE-GKVP-T-IREG--------
	Mycobacterium avium	WP_019736098	-E-L----GVV-T--Q--D---D-	DSD	PDE-GK-P-T--REG-----V--
	Mycobacterium branderi	WP_083130528	-E-LD---GIV-T--Q--D---D-	DSD	PDE-GK-P-T--REG-----V--
	Mycobacterium chimaera	ASL11505	VE-LS---GIV-T--Q--D---D-	DSD	-HE-GK-P-T-VREG------V-
	Mycobacterium conspicuum	WP_085235531	VE-LD---DLV-T--Q--D---D-	GSE	-EE-GKVP-T--REG--------
	Mycobacterium fragae	WP_085197489	VQ-LS---GIV-T--Q--D---D-	DSD	PDE-GK-P-T--REG-----V--
	Mycobacterium gilvum PYR-GCK	ABP47717	-E-L-K--GIV-T--Q--D---D-	DSD	ADE-GKVP-T--REG-----V--
	Mycobacterium hassiacum DSM 44	EKF25182	VE-LS---GIV-T--Q--D---D-	DSE	ADE-GKQP-T--REG-----V--
	Mycobacterium holsaticum	WP_069404996	-E-LS---GIV-T--Q--D---D-	DSD	PEE-GKVP-T--REG-----V--
	Mycobacterium houstonense	WP_066898138	VE-LS---GIV-T--Q--D---D-	DSD	PDE-GKVP-T--REG-----V--
	Mycobacterium insubricum	WP_083033321	-E-L-T--GIV-T--Q--D---D-	ESE	ADE-GK-P-T--REG-----V--
	Mycobacterium intracellulare	WP_064939190	VE-LS---GIV-T--Q--D---D-	DSD	-HE-GK-P-T-VREG--------
	Mycobacterium iranicum	WP_064284223	VE-L----GIV-T--Q--D---D-	DSD	PDE-GKVP-T--REG-----V--
	Mycobacterium komaniense	CRL69876	-E-LS---GIV-T--Q--D---D-	DSD	PEE-GKVP-T--REG-----V--
	Mycobacterium kubicae	WP_085072853	-E-L----GIV-T--Q-AD---D-	GSE	-EE-GK-P-T-IREG-R------
	Mycobacterium litorale	WP_078017894	-A-LS---GIV-T--Q--D---D-	DSD	PDE-GK-P-T--REG-----V--
	Mycobacterium mageritense DSM	CDO20753	-E-LS---GIV-T--Q--D---D-	DSD	PDE-GK-P-T--REG-----V--
	Mycobacterium malmesburyense	WP_090345003	-E-LS---GIV-T--Q--D---D-	DSD	PEE-GKVP-T--REG-----V--
	Mycobacterium malmoense	WP_065443247	VE-LS---GIV-T--Q--D---D-	DSD	-HE-GK-P-T-IREG--------
	Mycobacterium montefiorense	GBG40653	VA-LS---GIV-T--Q--D---D-	DSD	-HE-GK-P-T-IREG------V-
	Mycobacterium moriokaense	WP_083150488	-E-LS---GIV-T--Q--D---D-	DSD	PDE-GK-P-T--REG-----V--
	Mycobacterium neumannii	WP_094294468	-E-LS---GIV-T--Q--D---D-	DSD	PDE-GKVP-T--REG-----V--
	Mycobacterium neworleansense	WP_090517351	-D-LH---GIV-T--Q--D---D-	DSD	PDE-GKVP-T--REG-----V--
	Mycobacterium obuense	WP_046363006	-E-L----GIV-T--Q--D---D-	DSD	PDE-GKVP-T--REG-----V--
	Mycobacterium paraffinicum	WP_073871002	VE-LS---GIV-T--Q--D---D-	DSD	-HE-GK-P-T-VREG------V-
	Mycobacterium parafortuitum	WP_083144752	-E-L-K--GIV-T--Q--D---D-	DSD	ADE-GK-P-T--REG-----V--
	Mycobacterium peregrinum	OBB26054	VE-LH---GIV-T--Q--D---D-	DSD	PDE-GKVP-T--REG-----V--
	Mycobacterium phlei	WP_040632604	-E-LS---GIV-T--Q--D---D-	ASD	PEE-GKVP-T--REG-----V--
	Mycobacterium phlei RIVM601174	EID18229	-E-LS---GIV-T--Q--D---D-	ASD	PEE-GKVP-T--REG-----V--
**Other *Mycobacteriaceae* (0/64)**	*Mycobacterium porcinum*	WP_069424818	-D-LH---GIV-T--Q--D---D-	DSD	PDE-GKVP-T--REG-----V--
	Mycobacterium rhodesiae	WP_014209449	-E-LS---GIV-T--Q--D---D-	DSD	PDE-GK-P-T--REG-----V--
	Mycobacterium rhodesiae JS60	EHB56065	VE-LS---GIV-T--Q--D---D-	DSD	PDE-GK-P-T--REG-----V--
	Mycobacterium rufum	KGI66788	-E-L----GIV-T--Q--D---D-	DSD	PDE-GKVP-T--REG-----V--
	Mycobacterium rutilum	WP_083405724	-E-LS---GIV-T--Q--D---D-	DSD	PEE-GKVP-T--REG-----V--
	Mycobacterium scrofulaceum	WP_067275280	VE-LS---GIV-T--Q--D---D-	DSD	-HE-GK-P-T-VREG------V-
	Mycobacterium septicum	WP_044515828	-D-LH---GIV-T--Q--D---D-	DSD	PDE-GKVP-T--REG-----V--
	Mycobacterium setense	WP_039314873	-D-LH---GIV-T--Q--D---D-	DSD	PDE-GKVP-T--REG-----V--
	Mycobacterium shimoidei	WP_069396889	VE-L----NIV-T--Q--D---D-	DSD	PDE-GK-P-T--REG-----V--
	Mycobacterium smegmatis	WP_011727438	-E-LS---GIV-T--Q-AD---D-	DSD	PDE-GKTP-T--REG-----V--
	Mycobacterium szulgai	ORX12858	VE-LS---NIV-T--Q-AD---D-	DSE	-QE-GKVP-T-IREG--------
	Mycobacterium thermoresistibile	EHI12202	VE-L----GIV-T--Q--D---D-	ESE	-TE-GKTP-T--REG-----V--
	Mycobacterium tusciae	WP_006244394	-E-LS---GIV-T--Q--D---D-	DSD	PDE-GK-P-T--REG-----V--
	Mycobacterium vaccae	WP_040542647	-E-L----GIV-T--Q--D---D-	DSD	PDE-GKVP-T--REG-----V--
	Mycobacterium wolinskyi	WP_067859049	-E-LS---GIV-T--Q--D---D-	DSD	PDE-GKVP-T--REG-----V--
	Mycolicibacillus trivialis	WP_085109403	-E-LS---GIV-T--Q--D---D-	ASD	PDE-GK-P-T--REG-----V--
	Mycolicibacter kumamotonensis	OBY31914	-E-L----GVV-T--Q--D---D-	DSD	PDE-GK-P-T--REG-----V--
	Mycolicibacter longobardus	WP_085266048	-E-L----GIV-T--Q--D---D-	DSD	PDE-GK-P-T--REG-----V--
	Mycolicibacter terrae	ORW96121	-E-L----GVV-T--Q--D---D-	DSD	PDE-GK-P-T--REG-----V--
	Mycolicibacterium agri	WP_097939858	-E-LS---GIV-TV-Q--D---D-	DSD	PDE-GKVP-T--REG-----V--
	Mycolicibacterium aurum	WP_048632214	TE-LS---GIV-T--Q--D---D-	DSD	ADE-GK-P-T--REG-----V--
	Mycolicibacterium austroafricanum	WP_105389455	-E-L----GIV-T--Q--D---D-	DSD	PDE-GKVP-T--REG-----V--
	Mycolicibacterium boenickei	WP_077742410	-D-LH---GIV-T--Q--D---D-	DSD	PDE-GKVP-T--REG-----V--
	Mycolicibacterium brumae	WP_090588480	VD-L----GIV-T--Q--D---D-	ESD	ADE-GK-P-T--REG-----V--
	Mycolicibacterium chubuense	WP_014814017	-E-LS---G-V-TV-Q-CD---D-	DSD	ADE-GKVP-T--REG-----V--
	Mycolicibacterium confluentis	WP_085149832	-E-L----GIV-MV-Q--D---D-	DSD	PDE-GK-P-T--REG-----V--
	Mycolicibacterium cosmeticum	CDO09603	-E-L----GIV-T--Q--D---D-	DSD	PDE-GK-P-T--REG-----V--
	Mycolicibacterium diernhoferi	WP_097933624	-E-L----GIV-T--Q--D---D-	DSD	PDE-GKVP-T--REG-----V--
	Mycolicibacterium duvalii	WP_098000666	-E-L--I-AIV-T--Q--D---D-	DSD	ADE-GKVP-T--REG-----V--
	Mycolicibacterium flavescens	WP_069415848	-E-LS---GIV-T--Q--D---D-	DSD	PDE-GKVP-T--REG-----V--
	Mycolicibacterium fortuitum	WP_064848155	VE-LH---GIV-T--Q--D---D-	DSD	PDE-GKVP-T--REG-----V--

Figure S36. Partial sequence alignment of the polyprenyl-diphosphate synthase GrcC protein showing a three amino acid deletion that is specific for members of the “Tuberculosis” clade.

	Organism	Accession	94		141
	Mycobacterium tuberculosis	AMC72161	ALREQTPDTSRLRELLAGPIHDDH		VAEALTLLRCSPGIGKAKNVVAAY
	Mycobacterium bovis	WP_046026275	------------------------		------------------------
	Mycobacterium canettii	WP_014000545	------------------------		------------------------
	Mycobacterium caprae	APU25148	------------------------		------------------------
**"Tuberculosis" clade (9/9)**	*Mycobacterium microti*	AMC58531	------------------------		------------------------
	Mycobacterium mungi	OAQ18283	------------------------		------------------------
	Mycobacterium orygis	EMT36698	------------------------		------------------------
	Mycobacterium pinnipedii	WP_105826514	------------------------		------------------------
	Mycobacterium africanum	AMC62964	----------------G-------		------------------------
	Mycobacterium heckeshornense	WP_099868739	---DSG-QAD---------VD-AV		L----Q---S---MA---DT--R-
	Mycobacterium alsense	WP_083136113	----PA-EAA------S--VE--A	Q	---------A---MA---HFLTQ-
	Mycobacterium angelicum	WP_083115663	----TG--SV---------VR--A	E	-L-------A---MA---D-L-Q-
	Mycobacterium avium	WP_042906693	----PG--AA------V---D--E	V	---------A---MA---QSLRE-
	Mycobacterium avium	WP_062895358	----PG--AA------V---D--E	V	---------A---MA---HAL-D-
	Mycobacterium avium subsp. hominissuis	KDP03581	----PG--AA------V---D--E	A	---------A---MA---QSLRE-
	Mycobacterium avium subsp. paratuberculosis	EGO39428	----PG--AA------V---D--E	V	---------A---MA---QSLRE-
	Mycobacterium bohemicum	WP_085179490	----PG-EAA------------ED	A	-T-------A---MT---QFL-E-
	Mycobacterium bohemicum DSM 44	CPR11809	----PG-EAA------------ED	A	-T-------A---MT---QFL-E-
	Mycobacterium branderi	WP_083130528	----TG--AD---Q-----VT--A	V	L--------A-A-MAQ--DT--R-
	Mycobacterium canettii	WP_015289146	----SG--CA---A--N--VD--A	E	-R-------A---MAR--D-L-Q-
	Mycobacterium celatum	WP_062540037	----TG--AD---K-----VS--A	V	L--------R---MAQ---T--R-
	Mycobacterium conceptionense	CQD08494	----SG--SD---------VER-E	D	---------R-A-MAR--ET--E-
	Mycobacterium conspicuum	WP_085235531	--A-PG--GA----------T--A	E	-L-------A---MA---D-L-Q-
	Mycobacterium flavescens	WP_069415848	---DTG--AD---------VER-D	D	-----R---A----AM--ET--Q-
	Mycobacterium goodii	WP_049748383	----TGA-AD---D-----VER-E	D	-----A---R---LT---QT--S-
	Mycobacterium heidelbergense	WP_083074558	----PG-EAA---------VD--G	A	-T---A---A---MA---HFL-Q-
	Mycobacterium interjectum	WP_066906997	----PGAEAA------S--VT--E	E	---------A---MA---DFL-Q-
	Mycobacterium intermedium	WP_069421126	---DTG--GD---------V---A	E	-H-------A-E-MA---D-L-G-
	Mycobacterium kansasii	OOK67047	---DTG--GA---A---------D	A	-L---A---A---MA---D-L-K-
	Mycobacterium kansasii 662	AIR19888	---DTG--GA---A---------D	A	-L---A---A---MA---D-L-K-
**Other *Mycobacteriaceae* (1/43)**	*Mycobacterium komaniense*	CRL69876	---DDG--AD---------VEN-D	D	-----R---A---MA---ET--Q-
	Mycobacterium kubicae	WP_085072853	----SG--GE---T-----V---A	Q	-----A---V---MD---E-L-Q-
	Mycobacterium kyorinense	WP_045376639	----TGR-AD---------VT--A	V	L--------A-A-MAQ--DT--R-
	Mycobacterium malmesburyense	WP_090345003	---DGG-EAD---------VEN-D	D	-----S---A---MA---ET--Q-
	Mycobacterium malmoense	WP_071512141	----PG-EAA------S--VD--E	A	---------A---MA---EFLGQ-
	Mycobacterium mantenii	WP_083093173	----PG-EAA------V---D--E	A	---------A---MV---HSLQE-
	Mycobacterium moriokaense	WP_083150488	---DSG--AD----------EN-D	D	L--------A-Q-MVR--QT--E-
	Mycobacterium noviomagense	WP_083089911	--QDRG-EAD---------V---A	V	L----R---A---MA---QT--R-
	Mycobacterium palustre	WP_085080870	----PG-EAA---------VG--A	A	-----S---S---MA---EFL-Q-
	Mycobacterium paraense	WP_085097394	----PG-EAA------S--VT--E	A	---------A---MAR--DFL-Q-
	Mycobacterium paraseoulense	WP_083171678	----SGANAA----------D--D	A	-----A---A---MA---QTL-E-
	Mycobacterium phlei	WP_040632604	---DTG--AD---------VT--A	D	---------R-A-MAR--ET--Q-
	Mycobacterium phlei RIVM601174	EID18229	---DTG--AD---------VT--A	D	---------R-A-MAR--ET--Q-
	Mycobacterium pinnipedii	WP_107131609	----SG--CA---A--N--VD--A	E	-R-------A---MAR--D-L-Q-
	Mycobacterium rhodesiae	WP_014209449	---DTG--AD---------VEN-D	D	L----R---A---MVR--QT--E-
	Mycobacterium riyadhense	WP_085251554	----TG-EAA---------V---A	E	-L-------A---MAT--D-L-R-
	Mycobacterium scrofulaceum	WP_067275280	----PG--AA----------D--E	A	---------A---MAR--QTL-E-
	Mycobacterium shinjukuense	WP_083047968	---DTGSEGA---T-----V---T	E	---------A---MA---D-L-Q-
	Mycobacterium smegmatis	WP_011727438	----TG--AD----------ER-E	D	-----A---R---LT---ET--S-
	Mycobacterium smegmatis str. M	AFP37579	----TG--AD----------ER-E	D	-----A---R---LT---ET--S-
	Mycobacterium szulgai	WP_085670070	----TG--SV---------VR--D	E	-L-------A---MA---D-L-Q-
	Mycobacterium wolinskyi	WP_067859049	----TG-EAD---------VER-E	D	-----S---G---MAR--ET--S-

Figure S37. Partial sequence alignment of the polyprenyl-diphosphate synthase GrcC protein showing a one amino acid deletion that is specific for members of the "Tuberculosis" clade and is absent from most other *Mycobacteriaceae*.

Group	Species	Accession	220		275
	Mycobacterium tuberculosis	AIH44962	KTAVAENISQSYIQVA		RKMDALTRVLEVEPFEAMIVFVRTKQATEEIAEKLRARGF
	Mycobacterium bovis	WP_024457259	----------------		--
**“Tuberculosis” clade (9/9)**	*Mycobacterium canettii*	WP_044096226	----------------		--
	Mycobacterium caprae	APU25380	----------------		--
	Mycobacterium microti	AMC58828	----------------		--
	Mycobacterium mungi	OAQ19120	----------------		--
	Mycobacterium orygis	EMT36515	----------------		--
	Mycobacterium pinnipedii	PRH94032	----------------		--
	Mycobacterium africanum	WP_031670454	----------------		--
	Mycobacterium alsense	WP_083140631	---T------R-----	GS	--------I---------------------V---------
	Mycobacterium asiaticum	WP_065143248	---T------R-----	GP	------------------------------V---------
	Mycobacterium avium complex	WP_009953174	---T------R-----	GP	------------------------------V---------
	Mycobacterium bohemicum	WP_085178946	---T------R-----	GP	------------------------------V---------
	Mycobacterium colombiense	WP_064952138	---T------R-----	GP	------------------------------V--R------
	Mycobacterium europaeum	WP_090421042	---T------R-----	GP	------------------------------V---------
	Mycobacterium gastri	WP_085105122	---T------R-----	GP	------------------------------V---------
	Mycobacterium gastri 'Wayne'	ETW23825	---T------R-----	GP	------------------------------V---------
	Mycobacterium haemophilum	WP_047314919	---T------R-----	GP	-------------------I----------V---------
	Mycobacterium heidelbergense	WP_083073532	---T------R-----	GP	------------------------------V---------
	Mycobacterium interjectum	WP_066914761	---T------R-----	GP	------------------------------V---------
	Mycobacterium intermedium	WP_069421593	---T------R-----	GP	------------------------------V---------
	Mycobacterium intracellulare	WP_064937731	---T------R-----	GP	------------------------------V---------
	Mycobacterium intracellulare M	ETZ34244	---T------R-----	GP	------------------------------V---------
**Other *Mycobacteriaceae* (0/31)**	*Mycobacterium kansasii*	ORB88944	---T------R-----	GP	------------------------------V---------
	Mycobacterium kyorinense	WP_065013221	---T------R-----	GP	------------------------------V---------
	Mycobacterium lacus	WP_085161798	----R-M---	GQ	-----------------------------DV--R-C----
	Mycobacterium malmoense	WP_065470684	---T------R-----	GP	------------------------------V--R------
	Mycobacterium marinum	WP_094358878	---T------R-----	GP	------------------------------V---------
	Mycobacterium marinum str. Europe	EPQ74475	---T------R-----	GP	------------------------------V---------
	Mycobacterium montefiorense	WP_108920612	---T------R-----	GP	-------------------I----------V---------
	Mycobacterium nebraskense	WP_046182870	---T-D----R-----	GP	------------------------------V---------
	Mycobacterium palustre	WP_085078122	---T------R-----	GP	--------I---------------------V---------
	Mycobacterium paraense	WP_085104253	---T------R-----	GP	------------------------------V---------
	Mycobacterium paraffinicum	WP_073879822	---T------R-----	GP	------------------------------V---------
	Mycobacterium paraseoulense	WP_083174887	---T------R-----	GP	------------------------------V---------
	Mycobacterium parmense	WP_085269015	---T------R-----	GP	------------------------------V---------
	Mycobacterium scrofulaceum	WP_067276273	---T------R-----	GP	------------------------------V---------
	Mycobacterium triplex	WP_036470807	---T------R-----	GP	--------I---------------------V---------
	Mycobacterium ulcerans	WP_096371550	---T------R-----	GP	------------------------------V---------
	Mycobacterium vulneris	WP_085292881	---T------R-----	GP	------------------------------V--R------

Figure S38. Partial sequence alignment of a cold-shock protein showing a two amino acid deletion that is specific for members of the “Tuberculosis” clade.

Group	Species	Accession	94		152
**“Tuberculosis” clade (12/12)**	*Mycobacterium tuberculosis*	AIH91383	TRLSQTRLNELAADYPTLDQNISEAVAAHGG	V	TRPVDQEVGSGLVVAFLRAGDAIACAL
	Mycobacterium africanum MAL010	KBG22509	-------------------------------	-	---------------------------
	Mycobacterium bovis	AMC54715	-------------------------------	-	---------------------------
	Mycobacterium bovis B2 7505	KAO02772	-------------------------------	-	---------------------------
	Mycobacterium bovis BCG str. A	AHM07114	-------------------------------	-	---------------------------
	Mycobacterium bovis BCG str. P	CAL71407	-------------------------------	-	---------------------------
	Mycobacterium canettii	WP_014000723	-------------------------------	-	---------------------------
	Mycobacterium caprae	WP_075744501	-------------------------------	-	---------------------------
	Mycobacterium microti	WP_105799575	-------------------------------	-	---------------------------
	Mycobacterium orygis 112400015	EMT36413	-------------------------------	-	---------------------------
	Mycobacterium pinnipedii	WP_105826236	-------------------------------	-	---------------------------
	Mycobacterium africanum	WP_031670234	-------------------------------	-	---------------------------
**Other *Mycobacteriaceae* (0/68)**	*Mycobacterium alsense*	WP_083139193	---WE-QPDDM-VAMAR---VLTDL----D-		A---E-GE-DSF-I--A--S--VC---
	Mycobacterium angelicum	WP_083114609	---WEAQPD-M--AMQR--TVV-DVI---D-		V---E-GE-DSF----T--S--VSA--
	Mycobacterium aquaticum	WP_095533761	-Q-W---PQDMT-AFAQ--TTLADL-E--R-		V--IE-GE-DSF----N--S--V----
	Mycobacterium asiaticum	OBI79507	---WE-QSEAMQDAVAR--AVLATT----H-		V---E-GE-DSF-I--T--A--VS---
	Mycobacterium avium subsp. avium	EUA25771	---WE--PE-MT-ALVQ-NKTVN--I-S-D-		V--LE-GE-DSF-A--A--S--V----
	Mycobacterium canettii	WP_080603405	-H-W-MCPEDM--AIAH--HTV---ITN---		VQ--K-YE-DSF-A--T--S--A----
	Mycobacterium canettii CIPT 14	CCK58918	-H-W-MCPEDM--AIAH--HTV---ITN---		VQ--K-YE-DSF-A--T--S--A----
	Mycobacterium celatum	PIB74260	---WE-QPE-MT-AIAR-NRVACDVI---D-		V---E-GE-DSF-A--A--S--V-T--
	Mycobacterium chimaera	WP_082408958	-S-W--QPEQMTTAIAQ--RALF-I----A-		V---E-GE-DSF----T--S--V----
	Mycobacterium colombiense	OBJ44878	---WESEPD-MT-AFAR--RTL-DV----H-		V---E-GE-DSF-I--A--S--V----
	Mycobacterium florentinum	WP_085224395	-Q-W-AAPD-M-GAVAR--EVLGRI--E-R-		V--IE-GE-DSF-L--R-----V----
	Mycobacterium fragae	WP_085194143	---WE-QPE-MT-AFAR--HAL-DLL-----		V---E-GE-DSF----A--S--V-S--
	Mycobacterium gastri	WP_036415254	---WE-QPETMT-ALTQ-NRTVD-TI---D-		A--LE-GE-DSF-A--A--S--V----
	Mycobacterium gordonae	OBJ80230	---WESQPQ-MRVAVAR--KAV-------D-		V---E-GE-DSF-I--A--T--V----
	Mycobacterium haemophilum	WP_047313978	---W-LQPEQME-AIAG--RAV---I-----		V-S-E-GE-DSF----D--S--AG---
	Mycobacterium interjectum	ORV86953	---W--KPD-MS-AIAR--RAV-DL--Q-S-		V---E-GE-DSF----A--S--V-F--
	Mycobacterium intermedium	ODQ97972	---WESQPK-MT-AIER--HALTDLL-----		V---E-GE-DSF----A--TE-V----
	Mycobacterium intracellulare	OBG03636	---WE--PE-MT-ALVQ-NKTVN--I-S-D-		V--LE-GE-DSF-A--A--S--V----
	Mycobacterium kansasii	KZS57309	---WE-QPETMT-ALAQ-NRTVD--I---D-		V--LE-GE-DSF-A--A--S--V----
	Mycobacterium kyorinense	WP_065015729	---WE-QPD-M--AIAR---AV---I-T-D-		A---E-GE-DSF-I--G--S--V----
	Mycobacterium lacus	WP_085161890	---WDAHPE-MGGAVKR--EALAGVI-----		V---E-GGHDSV----T--S--V-A--
	Mycobacterium lehmannii	WP_094286928	---WESQPD-MT-AFA---RTLA-V-G--D-		V--IE-GE-DSF-I--G--S--V----
	Mycobacterium lentiflavum	WP_090602358	---W--VPD-M-DAVAR---VLGRI--E-R-		V---E-GE-DSF-L--RH-T--V----
	Mycobacterium llatzerense	KIU15640	---WEAQPD-M--AVAQF-RR--DII-TY--		V---E-GE-DSF-A--S-----V----
	Mycobacterium malmesburyense	WP_090344856	---WE-QPD-MT-AFAN--RVLA-V-G----		V--IE-GE-DSF-I--G--S--V----
	Mycobacterium malmoense	OCB30356	---WE-QSDTMT-ALEQ-N-TVNDL------		V---E-GE-DSF-L--A--S--V----
	Mycobacterium marinum	WP_020729494	---WDNQPDQA--AITQ---ILA-VI---D-		V---E-GE-DSF----A--S--V----
	Mycobacterium montefiorense	GBG38058	---WDLTPG-M--AIGV--RAL-------R-		V---E-GE-DSF----A--S--V----
	Mycobacterium moriokaense	WP_083149086	-P-WE-QPH-MT-AFAN--RVLA-I-PS---		V--IE-GE-DSF----G--S-------
	Mycobacterium mucogenicum	WP_061010361	---WE-QPE-M--AIAR---TV-A-LVT-A-		V---E-GE-DSF-I--GC-S--V---V
	Mycobacterium neumannii	WP_094294587	---WE-QADDMT-AFA---RTLT-VIG--S-		V--IE-GE-DSF-I--G--S--V----
	Mycobacterium noviomagense	WP_083088940	---WE-QPE-MT-AFTR--HAL-DLLTSF--		A---E-GE-DSF----A--S--V----
	Mycobacterium novocastrense	GAT12791	---WE-QPD-MT-AFAN--RTLA-VIG--S-		V--IE-GE-DSF-I--G--S--V----
	Mycobacterium paraense	ORW30701	-Q-WE-QPQ-M-KAVAR--RTLAQI--Q-H-		V---E-GE-DS-----N-GT--L----
	Mycobacterium paraffinicum	WP_073875389	-Q-WNSQPA-MT-AIAN---TLA-V-GI-R-		V---E-GE-DSF-I--N--S--V----
	Mycobacterium paraintracellulare	WP_014384097	-Q-WDSQPR-MT-AIAK---TLARL-G--H-		I---E-GE-DSF-I--S--S--V----
	Mycobacterium parascrofulaceum	WP_085981628	---WE-QPEQMT-ALAR-NKTVND--D--A-		V--LE-GE-DSF-A--S--S--V----
	Mycobacterium paraseoulense	WP_083166763	---WE-QPDKVT-AMAQ-NKTVN---P--D-		V--LE-GE-DSF-A--A--S--VS---
	Mycobacterium persicum	WP_083156214	---WH-QPAQM-DAIKL---AVD---SV---		V---E-GE-DSF-A--G--A--V----
	Mycobacterium porcinum	WP_075923785	---WEAQPEDMT-AFEC--RTLGIL-TE-H-		V--ME-GE-DSF----S--S--V----
	Mycobacterium rhodesiae	ORB56109	---WE-QSESM--ALDQ-NRTVDDL-PT---		V---E-GE-DSF----A--S--V----
	Mycobacterium riyadhense	WP_085250552	---WDIQPDQMSVAVAR---AV-DVT-----		V---E-GE-DSF-I--T--S--V-A--
	Mycobacterium scrofulaceum	OBH77515	---WE-QPEQMT-ALAR-NKTVND--D--A-		V--LE-GE-DSF-A--S--S--V----
	Mycobacterium shimoidei	ODR13073	-Q-WE-QPDDMSDAFTR--HALT-VI---D-		V---E-GE-DSF----P--S-------
	Mycobacterium shinjukuense	WP_083046517	---WE-LPEQM--ALAR-N-TVT-TIE--H-		V--LE-GE-DSF-A--A--S--V----
	Mycobacterium szulgai	WP_085672688	---WE-EPD-MT-AVAR--RTLADLL-----		V---E-GE-DSF----A--TE-V----
	Mycobacterium triplex	WP_084163278	-L-WE-QPE-MT-AVAY--RILV-LL-----		V---E-GE-DSF----A--SQ------
	Mycobacterium tusciae	WP_040538944	---WE-QPD-MT-AVAR--TVVANEIS--N-		V---E-GE-DSF----A--S--V----
	Mycobacterium vulneris	WP_085288066	---WE-QSETMTTALDQ-NRTVNDL--G---		V---E-GE-DSF-A--A--S--V----
	Mycolicibacter arupensis	WP_046189249	---WE-QADQMT-AIER--RILA-VLV--S-		V---E-GE-DSF-A--A--S--V---V
	Mycolicibacter heraklionensis	OBG31410	---WE-QAEQMT-AIAR--HTLA-VL---D-		V---E-GE-DSF-A--A--S--V----
	Mycolicibacter icosiumassiliensis	WP_078058768	-Q-WE-QAERMT-AIAS--RTLA-VL---H-		V--IE-GE-DSF-A--A--S--V----
	Mycolicibacter sinensis	OBG04837	---WEFQPD-MTLAVAR--RTLV-L---YR-		V---E-GE-DSF-L--T--S--VG---
	Mycolicibacter terrae	WP_085261241	---WESQPD-MT-AVAR--RALA-L--TYQ-		V---E-GE-DSF-L--A--S--VG---
	Mycolicibacter virginiensis	WP_105295293	---WE-QAEQMT-AIAR--HTLA-VL---D-		V---E-GE-DSF-A--A--S--V----
	Mycolicibacterium celeriflavum	WP_083006986	---WE-QPE-MRVAIAM--RTLADLTSM-N-		V---E-GE-DSF-L--A--S--V----
	Mycolicibacterium chlorophenolicum	WP_082168977	---W--QPE-M--AIAR--HTVW-VIST-H-		V---E-GE-DSF----A--S--V----
	Mycolicibacterium chubuense	WP_083119086	---WE--PEHMRSAIAL--EVLR-LTEV-S-		V---E-GE-DSF-L--A--S--V----

Figure S39. Partial sequence alignment of a transcriptional regulator protein showing a one amino acid insertion that is specific for members of the “Tuberculosis” clade.

Group	Species	Accession	150		208
"Tuberculosis" clade (9/9)	*Mycobacterium tuberculosis*	AIH45031	AHGGQTVMSGATEDAVLGRLPMRAWLIGL	RPME	GSPEGHNFPQSQRIAQLCHPNLRNTF
	Mycobacterium bovis	WP_024457230	-----------------------------	----	--------------------------
	Mycobacterium canettii	WP_015289796	-----------------------------	-L--	--------------------------
	Mycobacterium caprae	APU25476	-----------------------------	----	--------------------------
	Mycobacterium microti	AMC58949	-----------------------------	----	--------------------------
	Mycobacterium mungi	OAQ17721	-----------------------------	----	--------------------------
	Mycobacterium orygis	EMT36366	-----------------------------	----	--------------------------
	Mycobacterium pinnipedii	PRH93554	-----------------------------	----	--------------------------
	Mycobacterium africanum	CCC26455	-----------------------------	----	--------------------------
Other *Mycobacteriaceae* (0/43)	*Mycobacterium asiaticum*	OBI91067	-------L--T---L-VDA--TD----D-		-RH-LRGVARPE-VV-----DI--D-
	Mycobacterium avium subsp. avium	EUA25771	-------L--V--SL-VD---EDV--AD-		--HPLRDL-RPE-VV-V---D---E-
	Mycobacterium bohemicum DSM 44	CPR13160	-------L--TA--L-VDT--AD---TD-		--H-LRGVARPE-VV-----DI--D-
	Mycobacterium celatum	ORV15424	-------L-----QL-VD---EG---TD-		-THPLRDL-RPE-VV-----D-V-E-
	Mycobacterium celeriflavum	WP_083006986	-------L-----SM--DH--VD---TD-		-THTLRDL-RPE-VV-----DIP-E-
	Mycobacterium chimaera	ASL13824	-------L--V--SL-VD---EDV--AD-		--HPLRDL-RPE-VV-V---D---E-
	Mycobacterium chlorophenolicum	WP_082168977	-------L-AT---L-FD---AD----D-		-AHQLRDL-RLE--------D---D-
	Mycobacterium colombiense	OMC22620	-------L--T---L-VDA--PN---TD-		-RH-LRGVARPE-V------DI--E-
	Mycobacterium florentinum	WP_085219889	G------L--V--SL-VD---DG---AD-		--YPLRDL-RPE-VV-----D---E-
	Mycobacterium fragae	WP_085194143	-------L--TA--L-VDA--AD---TD-		-TY-LRGVARPE-VV-----D---D-
	Mycobacterium franklinii	WP_070936487	G------L------I-VDW--TN---LD-		-THQLRDL-RAE-VV-----D-S-D-
	Mycobacterium gastri	WP_036411119	------LL-AT-S-I-IDE--PD---VD-		--QPLRDL-RPE-V------Q---E-
	Mycobacterium gastri 'Wayne'	ETW25423	------LL-AT-S-I-IDE--PD---VD-		--QPLRDL-RPE-V------Q---E-
	Mycobacterium gordonae	OBJ80230	-------L-----PL-IDQ--PGS--TD-		-THALRDL-RPE-VI-----D---S-
	Mycobacterium intermedium	WP_069420655	-------L-----AM--D---DDV---E-		--YALRGV-RPE-VV-----EP--E-
	Mycobacterium intracellulare	OBG03636	-------L--V--SL-VD---EDV--AD-		--HPLRDL-RPE-VV-V---D---E-
	Mycobacterium intracellulare 1	EUA57763	-------L--V--SL-VD---EDV--AD-		--HPLRDL-RPE-VV-V---D---E-
	Mycobacterium intracellulare A	AFC42261	-------L--V--SL-VD---EDV--AD-		--HPLRDL-RPE-VV-V---D---E-
	Mycobacterium intracellulare M	AFC47404	-------L--V--SL-VD---EDV--AD-		--HPLRDL-RPE-VV-V---D---E-
	Mycobacterium intracellulare s	OCB23789	-------L--V--SL-VD---EDV--AD-		--HPLRDL-RPE-VV-M---D---E-
	Mycobacterium kansasii	OOK67750	------LL-AT-S-I-IDE--AD----D-		-IQPLRDL-RPE-V------Q---E-
	Mycobacterium kansasii 732	EUA10622	-------L-----PL-VD---EGV--AD-		--HPLRDL-RPE-VV-----D---E-
	Mycobacterium kansasii 824	EUA04134	------LL-AT-S-I-IDE--AD----D-		-IQPLRDL-RPE-V------Q---E-
	Mycobacterium kansasii ATCC 12	AGZ52470	------LL-AT-S-I-IDE--AD----D-		-IQPLRDL-RPE-V------Q---E-
	Mycobacterium komaniense	CRL66992	-------LT----SM-VDY--EG---SD-		--HPLRDL-RPE-VV-----DI--D-
	Mycobacterium lacus	WP_085161939	-------L--VV-SL--D---DG----D-		-THPLRDL-RPE-VV-----D--VE-
	Mycobacterium lehmannii	WP_094286928	-------L--T-T-L-SD---VG---AE-		-MYPVRDL-RPE-VV-----DI--E-
	Mycobacterium llatzerense	KIU15640	-------L--T-S-V-ID---TD-F-LD-		-VHALRDL-RPE-VV-----D---D-
	Mycobacterium malmesburyense	WP_090344856	-------L--T-T-L-SD---ID---AE-		-THPVRDL-RPE-VV-----DV--E-
	Mycobacterium moriokaense	WP_083149086	-------L--T-T-L-CD---ED---AE-		-THAVRDL-KPE--V-----DI--E-
	Mycobacterium neumannii	WP_094294587	-------L--T-T-L-SD---VD---AD-		-THTVRDL-RPE-VV-----DI--E-
	Mycobacterium noviomagense	WP_083088940	-------L--T---L-VDA--AD---T--		-TH-LRGVARPE-VV-----D---E-
	Mycobacterium novocastrense	WP_067389187	-------LT----SM-VDY--EG---SD-		--HPLRDL-RPE-VV-----DI--D-
	Mycobacterium paraense	ORW40175	-------L--TA--L-VDT--AD---TD-		--H-LRGVARPE-VV-----DI--D-
	Mycobacterium paraintracellulare	WP_014383957	-------L--V--SL-VD---EDV--AD-		--HPLRDL-RPE-VV-V---D---E-
	Mycobacterium parascrofulaceum	EFG75144	-------L-----SM-VD---DGV--AD-		--YPLRDL-RPE-VV-----D-A-E-
	Mycobacterium paraseoulense	WP_083172274	-------L-----QL-VD---AD---TD-		-LHPLRDL-RPE-VV--N--G---D-
	Mycobacterium persicum	WP_083156214	------LL-AT-S-I-IDE--AD----D-		--QPLRDL-RPE-V------Q---E-
	Mycobacterium scrofulaceum	WP_067304799	-------L-----SM-VD---DGV--AD-		--YPLRDL-RPE-VV-----D-V-E-
	Mycobacterium shimoidei	WP_069396374	-------L-----AL-AD---AD---TE-		--YPLRGV-RPE-VL-----DI--E-
	Mycobacterium shinjukuense	WP_083046517	-------L--V--LL-IDH--DGV--TE-		--YPLRDL-RPE-VV-----A---E-
	Mycobacterium sinense	OBG04837	-------L---A-AL-VDW--TD----E-		-CYPLRGA-RPE-VV-----D---E-
	Mycobacterium tusciae	WP_040538944	-------L--T-S-L-ID---VG-F-TD-		--HPLRDL-RPE-VV-----D---DY

Figure S40. Partial sequence alignment of the hypothetical protein IQ40_04435 showing a four amino acid insertion that is specific for members of the "Tuberculosis" clade.

Group	Species	Accession	296		344
"Tuberculosis" clade (10/10)	*Mycobacterium tuberculosis*	AIH45133	GAIANGGEIDGIRFLSWELVTGLT	R	NRRQVLPDRNLLVPLNFHLGYHGM
	Mycobacterium bovis	WP_049950496	----------------R-------	-	------------------------
	Mycobacterium bovis BCG str. A	AHM07257	----------------R-------	-	------------------------
	Mycobacterium canettii	WP_015288077	----------------R---A---	-	------------------------
	Mycobacterium caprae	CEJ52494	----------------R-------	-	------------------------
	Mycobacterium microti	PRI04882	----------------R-------	-	------------------------
	Mycobacterium mungi	OAQ17797	----------------R-------	-	------------------------
	Mycobacterium orygis	EMT36281	----------------R-------	-	------------------------
	Mycobacterium pinnipedii	PRH90308	----------------R-------	-	------------------------
	Mycobacterium africanum	WP_031701576	----------------R-------	-	------------------------
Other *Mycobacteriaceae* (0/30)	*Mycobacterium alsense*	WP_083140056	-----------T----R---A---		G--SLR-----F---A------SL
	Mycobacterium avium MAV_120709	ETZ54726	--------V-------RD--A---		GE-SLR-----Y---A------SV
	Mycobacterium avium MAV_120809	ETZ36628	--------V-------RD--A---		GE-SLR-----Y---A------SV
	Mycobacterium avium subsp. paratuberculosis	ETB50302	--------V-------RD--A---		GE-SLR-----Y---A------SV
	Mycobacterium colombiense	WP_064885345	--------V-------R---A---		GE-SLR----IY---A------SL
	Mycobacterium europaeum	CQD22792	--------V-------R-M-A---		G--SLR-----FM--A------SL
	Mycobacterium gastri	WP_036412899	-------Q---T----P--IA---		GE-RLR---------A------SL
	Mycobacterium gastri 'Wayne'	ETW24424	-------Q---T----P--IA---		GE-RLR---------A------SL
	Mycobacterium heidelbergense	WP_083075186	-----------T----R---A---		G-PSLR-----F---A------SL
	Mycobacterium interjectum	WP_066910755	-----------TQ---R---A---		G--SLR-----F---A------SL
	Mycobacterium kansasii	WP_075509721	-------Q---T----P--IA---		GE-RLR---------A------SL
	Mycobacterium kansasii 732	EUA12245	-----------T----R---A---		GE-RLR-----F--MA------SF
	Mycobacterium lacus	WP_08516299	-------Q---TQ---P---A---		G--DLR---------A------SV
	Mycobacterium liflandii	WP_041299297	-----------TQ---R---A---		GE-RLQ-----FM-MA------SL
	Mycobacterium liflandii 128FXT	AGC63013	-----------TQ---R---A---		GE-RLQ-----FM-MA------SL
	Mycobacterium malmoense	WP_065444433	--------V-------R-M-A---		G--SLR-----FM--A------SL
	Mycobacterium mantenii	WP_083097534	--------V--T----R---A---		G--SLR-----F---A------SL
	Mycobacterium marinum	WP_012394056	-----------TQ---R---A---		GE-RLQ-----FM-MA------SL
	Mycobacterium marinum MB2	EPQ76079	-----------TQ---R---A---		GE-RLQ-----FM-MA------SL
	Mycobacterium montefiorense	GBG38470	----------------P-I-A--A		G--SLKR---IM---S-------V
	Mycobacterium nebraskense	WP_046183089	--------V-------R-M-A---		G--SLR-----F---A------SL
	Mycobacterium palustre	WP_085080710	-----------A----R---A---		G--SLR-----F---A------SL
	Mycobacterium paraense	WP_085096747	-----------TQ---R---A---		G--SLR-----F---A------SL
	Mycobacterium paraffinicum	WP_073872472	--------V-------R-M-A---		G--SLR-----F---A------SL
	Mycobacterium parascrofulaceum	EFG74457	--------V-------R-M-A---		G--SLR-----F---A------SL
	Mycobacterium paraseoulense	WP_083173529	--------V-------R-M-A---		G--SLR-----F---A------SL
	Mycobacterium pseudoshottsii J	GAQ39333	-----------TQ---R---A---		GE-RLQ-----FM-MA------SL
	Mycobacterium scrofulaceum	WP_067268776	--------V-------R---A---		G--SLR-----FM--A------SL
	Mycobacterium shinjukuense	WP_083051086	-------M---SQ---R---A---		GQ-RIR----------------SV
	Mycobacterium ulcerans str. Harvey	EUA91103	-----------TQ---R---A---		GE-RLQ-----FM-MA------SL

Figure S41. Partial sequence alignment of an esterase protein showing a one amino acid insertion that is specific for members of the "Tuberculosis" clade.

			165		213
“Tuberculosis” clade (21/22)	*Mycobacterium tuberculosis*	AMC72874	GGTLLPMADVIRMTSHAHHY	SPASGRYP	QAIFDHGTPLALYHTKRLASP
	Mycobacterium africanum GM0411	CCC25169	--------------------	--------	---------------------
	Mycobacterium africanum K85	KBF47630	--------------------	--------	---------------------
	Mycobacterium africanum MAL010	KBG08006	--------------------	--------	---------------------
	Mycobacterium bovis	WP_080663112	--------------------	--------	---------------------
	Mycobacterium bovis AF2122/97	YP_009360856	--------------------	--------	---------------------
	Mycobacterium bovis AN5	ESK75391	--------------------	--------	---------------------
	Mycobacterium bovis B2 7505	KAN87758	--------------------	--------	-------K-------------
	Mycobacterium bovis BCG	AAB96960	--------------------	--------	---------------------
	Mycobacterium bovis BCG str. A	AHM09251	--------------------	--------	---------------NG----
	Mycobacterium bovis BCG str. P	CAL71612	--------------------	--------	---------------------
	Mycobacterium bovis Bz 31150	KAN92248	--------------------	--------	---------------------
	Mycobacterium bovis MAL010093	KBG51344	--------------------	--------	---------------------
	Mycobacterium bovis MAL010093	KBG56464	--------------------	--------	---------------------
	Mycobacterium canettii	WP_080602950	--S----------A------		L--------------------
	Mycobacterium caprae	WP_083647839	--------------------	--------	---------------------
	Mycobacterium microti	AMC57538	--------------------	--------	---------------------
	Mycobacterium mungi	OAQ17315	--------------------	--------	---------------------
	Mycobacterium orygis	WP_081608392	--------------------	--------	---------------------
	Mycobacterium orygis 112400015	EMT33889	--------------------	--------	---------------------
	Mycobacterium pinnipedii	PRH91488	--------------------	--------	---------------------
	Mycobacterium africanum	WP_080699388	--------------------	--------	---------------------
Other *Mycobacteriaceae* (1/49)	*Mycobacterium gordonae*	WP_065044389	-------S--L-LATS----	A-------	-T---QDKT-----G----T-
	Mycobacterium alsense	WP_083141168	--S----T-----AG-----		L-----AK------------R
	Mycobacterium angelicum	ORA12304	---I---P----LGRQ-N--		L----Q-K-------------
	Mycobacterium arosiense	WP_083067482	-------S----LAR--R--		L------KA-----AR-----
	Mycobacterium asiaticum	OBI87458	--S------L--LAAQ----		L----DAK-----------N-
	Mycobacterium avium	WP_062895105	-------S----LAR-----		L-V--N-K-------------
	Mycobacterium bohemicum	WP_085181641	----V--S----WAG-----		L------K-------------
	Mycobacterium chimaera	WP_082408902	---I---SE---LAR-----		L----K-KA------------
	Mycobacterium colombiense	OBJ73102	---I---S----LAR-----		L----K-KA------------
	Mycobacterium conspicuum	WP_085233296	---I---S----LAR-----		L----K-KA------------
	Mycobacterium europaeum	WP_085240419	---I---S----LAR-----		L-V--K-K-V----G------
	Mycobacterium gastri	WP_036415427	--SR---S-L---A------		L-L--R-KA------------
	Mycobacterium heckeshornense	WP_048890622	--S----SE---LAQ-----		L----N-RA--------I---
	Mycobacterium heidelbergense	ORA65721	--S---LS-----A------		L-L--NAK---------F---
	Mycobacterium interjectum	WP_085202023	----V--S----WAG-----		L----K-KA---H--------
	Mycobacterium intracellulare	WP_064893654	--S-V--S-L---A------		L----R-RA-S-H--------
	Mycobacterium kansasii	KZS64453	--SQ---S-L--LA------		L-L--D-KA-----S------
	Mycobacterium kubicae	WP_085072951	-------S----LSR-----		L----R-NA------------
	Mycobacterium lacus	WP_085155832	--SRV--S-L---A-S----		L-L--G-K------A------
	Mycobacterium malmoense	WP_065442061	----V--S----WAG-----		L----RAR-------------
	Mycobacterium mantenii	WP_083099146	---VV--S----WA------		L------KA---H--------
	Mycobacterium marseillense	ORA94115	---I---S----LDR--N--		L----K-NA------------
	Mycobacterium nebraskense	WP_046186637	----V--S----WAG-----		L----R-RS------------
	Mycobacterium palustre	WP_085079703	----V--S----WA---Y--		L----R-KA---H--------
	Mycobacterium paraense	WP_085094522	---I---S----QAR---Q-		L----K-QAI-----------
	Mycobacterium paraintracellulare	AFC54248	---I---SE---LAR-----		L----K-KA------------
	Mycobacterium parascrofulaceum	WP_007171972	----V--S----WAG-----		L----QAR-------------
	Mycobacterium paraseoulense	WP_083172732	---I---S----LAR-----		L-V--R-E------G------
	Mycobacterium parmense	WP_085269507	---I---S----LAG--N--		L----K-KA------------
	Mycobacterium scrofulaceum	ORB62829	----V--S----WAG-----		L-----AR-------------
	Mycobacterium shimoidei	WP_085178757	-------SE---LAA-----		LV--E--KAIG-F--------
	Mycobacterium shinjukuense	WP_083050485	--S----SE---LAA-----		LR-----RE------------
	Mycobacterium szulgai	WP_085671879	---I---S----LGRQ-N--		L----E-K-------------
	Mycobacterium vulneris	WP_085291349	---I---S----LAR-----		L----R-KA------------
	Mycobacterium xenopi	WP_085194972	--S----SE---LAQ-----		L----K-RA--------I---
	Mycobacterium xenopi	WP_003918915	--S----SE---LAQ-----		L----K-RA--------I---
	Mycobacterium xenopi 3993	EUA24629	--S----S--A-LA------		L----K-RA------------
	Mycobacterium xenopi 4042	EUA78644	--S----S--A-LA------		L----K-RA------------
	Mycobacterium xenopi RIVM70036	EID12217	--S----S--A-LA------		L----K-RA------------
	Mycolicibacillus trivialis	ODR01013	-------S----LAR-----		L----R-KTIG-F--------

Figure S42. Partial sequence alignment of the hypothetical protein RN11_1864 showing an eight amino acid insertion that is specific for most members of the “Tuberculosis” clade and is absent from most other *Mycobacteriaceae*.

			579	(boxed)	624
**"Tuberculosis" clade (11/11)**	*Mycobacterium tuberculosis*	AIH39014	DPRPLVLTEHRWAGRISSADYSG		TTPVGSMTLPKRVEHRQPRVRRD
	Mycobacterium africanum MAL010	KBG20366	-----------------------		-----------------------
	Mycobacterium bovis	WP_099217231	-----------------------		-----------------------
	Mycobacterium bovis AF2122/97	YP_009359459	-----------------------		-----------------------
	Mycobacterium canettii	WP_014001127	-----------------------		-----------------------
	Mycobacterium caprae	APU26116	-----------------------		-----------------------
	Mycobacterium microti	AMC59747	-----------------------		-----------------------
	Mycobacterium mungi	OAQ16566	-----------------------		-----------------------
	Mycobacterium orygis	EMT35678	-----------------------		-----------------------
	Mycobacterium pinnipedii	PRH89975	-----------------------		-----------------------
	Mycobacterium africanum	WP_061846055	-----------------------		-----------------------
**Other *Mycobacteriaceae* (0/>100)**	*Mycobacterium alsense*	WP_083137868	---------N-----------T-	A	SG-----S---------------
	Mycobacterium angelicum	WP_083115490	-----------------------	A	-P---------------------
	Mycobacterium arosiense	WP_083066950	---------N-------------	V	SA-----P---------------
	Mycobacterium asiaticum	WP_065034176	---------------------T-	A	-P---------------------
	Mycobacterium avium	WP_076221066	---------N-------------	N	SA-----P---------------
	Mycobacterium avium 09-5983	ETB25337	---------N-------------	N	SA-----P---------------
	Mycobacterium avium MAV_120709	ETZ42095	---------N-------------	N	SA-----P---------------
	Mycobacterium avium XTB13-223	KBR69579	---------N-------------	N	SA-----P---------------
	Mycobacterium avium subsp. avium	EUA36540	---------N-------------	N	SA-----P---------------
	Mycobacterium avium subsp. hominissuis	ETB41611	---------N-------------	N	SA-----P---------------
	Mycobacterium avium subsp. paratuberculosis	OUZ02606	---------N-------------	N	SA-----P---------------
	Mycobacterium bohemicum	WP_085180955	-----------------------	A	SE-----S----I----------
	Mycobacterium bohemicum DSM 44	CPR04576	-----------------------	A	SE-----S----I----------
	Mycobacterium branderi	WP_083131920	-----------------------	A	S----------------------
	Mycobacterium celatum	WP_062541814	-----------------------	A	A----------------------
	Mycobacterium chimaera	WP_054585322	---------N-------------	M	SA-----P---------------
	Mycobacterium colombiense	WP_064881452	---------N-------T-----	A	SA-----S---------------
	Mycobacterium conspicuum	WP_085232924	-----------------------	A	AA---------------------
	Mycobacterium florentinum	WP_085220593	-----------------------	A	SA-L---P---------------
	Mycobacterium fragae	WP_085199881	-----------------------	A	S--M---S---------------
	Mycobacterium gastri	WP_036410319	---------N-------------	G	VP------------Y--------
	Mycobacterium genavense	WP_025735453	----------L------------	A	VA-L---S---------------
	Mycobacterium gordonae	WP_055579722	-----------------------	A	-P---------------------
	Mycobacterium haemophilum	WP_047316462	-----------------------	A	AA---------------------
	Mycobacterium hassiacum	WP_005628004	E-------------------F--	A	SAKL-R-R--------N--A---
	Mycobacterium heckeshornense	WP_048892127	-------------------N-P-	A	SA---------------------
	Mycobacterium heidelbergense	WP_083072431	-----------------------	A	SA-----S---------------
	Mycobacterium insubricum	WP_083029933	----------K------T-----	P	SAKI---P--------V------
	Mycobacterium intermedium	WP_069420335	---------------------P-	A	NP---------------------
	Mycobacterium intracellulare	WP_064938952	---------N-------------	M	SA-----P---------------
	Mycobacterium iranicum	WP_064285271	-------S---------------	A	-G---K----------N--A---
	Mycobacterium kansasii	ORB88267	---------N-------------	G	VP------------Y--------
	Mycobacterium komaniense	WP_090273992	-----------------------	T	SA-L------------N--A---
	Mycobacterium kubicae	WP_085075427	-----------------------	V	-P---------------------
	Mycobacterium kyorinense	WP_065013412	-----------------------	A	A------S---------------
	Mycobacterium lacus	WP_085159039	-----------------------	A	QE---------------------
	Mycobacterium lentiflavum	WP_090609143	-----------------------	A	-A-L---S---------------
	Mycobacterium leprae	WP_010908278	N--------------------M-	A	AA---------------Q-----
	Mycobacterium lepromatosis	WP_045843071	------------------S--V-	A	AA--------------------E
	Mycobacterium liflandii	WP_041300286	---------N-----------T-	E	SP---------------------
	Mycobacterium liflandii 128FXT	AGC62519	---------N-----------T-	E	SP---------------------
	Mycobacterium llatzerense	WP_082067985	-----------------A---A-	T	SQRL---S----------K----
	Mycobacterium malmesburyense	WP_090346536	-----------------------	T	SA-L---P--------N--A---
	Mycobacterium malmoense	WP_065443144	---------N-------T-----	A	PA-----P---------------
	Mycobacterium marinum	WP_094360196	---------N-----------T-	E	SP---------------------
	Mycobacterium marinum str. Europe	EPQ80767	---------N-----------T-	E	SP---------------------
	Mycobacterium marseillense	WP_083018822	---------N-------------	A	SA-----P---------------
	Mycobacterium microti OV254	PLV50158	-----------------------	A	AA--A--S----------K----
	Mycobacterium montefiorense	GBG39307	-----------------------	A	AE--AT-P---------------
	Mycobacterium mucogenicum	WP_061001588	-----------------A---A-	T	SQRL---S----------K----
	Mycobacterium nebraskense	WP_085165685	---------N-------T-----	A	SA-----P---------------
	Mycobacterium neoaurum	WP_030135490	-----------------------	G	SA-L---H---------------
	Mycobacterium neumannii	WP_094293543	---------------------A-	T	SA-L-T-P--------N--A---
	Mycobacterium noviomagense	WP_083087419	---------N-------------	A	SA---------------------
	Mycobacterium novocastrense	WP_067389524	-----------------------	T	SA-M-T-P--------N--A---
	Mycobacterium palustre	WP_085076295	-----------------------	A	SE-----S---------------
	Mycobacterium paraense	WP_085101710	---------N-------------	A	-A-----S---------------
	Mycobacterium paraffinicum	WP_073873503	---------N-------T-----	A	-A-----P---------------
	Mycobacterium parafortuitum	WP_083144453	-------S-------------A-	A	AG-L-R-S---H--L-N--A---
	Mycobacterium paraseoulense	WP_083172756	---------N-------T-----	A	SA-----P---------------
	Mycobacterium parmense	WP_085268696	---------N-------------	V	SP-----P--R------------
	Mycobacterium persicum	WP_083155888	---------N-------------	G	VP------------Y--------

Other *Mycobacteriaceae* (0/>100)

Mycobacterium pseudoshottsii J	BBA88652	---------N-----------T- E SP---------------------
Mycobacterium rhodesiae	WP_005144386	----------H----V----FT- A AA-L---S---------------
Mycobacterium riyadhense	WP_085250890	----------------------- A -P---------------------
Mycobacterium rufum	KGI68633	---------S------------- A VG-M-R----------N--A---
Mycobacterium rutilum	WP_083408472	---------------------N- G -P-L------------N--A---
Mycobacterium saskatchewanense	WP_085254100	----------------------- A SA-----A--------------
Mycobacterium scrofulaceum	WP_067269749	---------N-------T----- A SA-----P---------------
Mycobacterium sherrisii	WP_069400468	----------------------- A AA--A--S---------------
Mycobacterium shigaense	WP_096440722	----------------------- A -E-----S---------------
Mycobacterium shimoidei	WP_069398024	--------------------F-- A A----------------------
Mycobacterium shinjukuense	WP_083049941	----------------------- A LP-----A---------------
Mycobacterium simiae	WP_061559683	----------------------- A AA--A--S----------K----
Mycobacterium smegmatis	CKI05606	----------K------------ A SA-L---S-------------K-
Mycobacterium smegmatis str. M	AFP40253	----------K------------ A SA-L---S-------------K-
Mycobacterium szulgai	WP_068033385	----------------------- V -P---------------------
Mycobacterium talmoniae	WP_071027792	-----------------A---T- A AA---------------------
Mycobacterium triplex	WP_036469688	----------------------- A AA-L---S---------------
Mycobacterium tusciae	WP_006245285	----------------------- A SA-L------------N--A---
Mycobacterium ulcerans	WP_071497583	---------N-----------T- E SP---------------------
Mycobacterium ulcerans str. Harvey	EUA89887	---------N-----------T- E SP---------------------
Mycobacterium ulcerans subsp. shinshuense	BAV41678	---------N-----------T- E SP---------------------
Mycobacterium vaccae	WP_064635138	-------S-------------A- G -G---R-S---H----N--A---
Mycobacterium vaccae ATCC 2595	EJZ07264	-------S-------------A- G -G---R-S---H----N--A---
Mycobacterium wolinskyi	WP_067852873	----------------------- A SARL-A-----------------
Mycobacterium xenopi	ORX16804	-------------------N-P- A AA---------------------
Mycobacteroides abscessus	WP_070410339	----------------------- G AE-L--IV----------K----
Mycobacteroides abscessus 1948	EUA60730	----------------------- G AE-L--IA----------K----
Mycobacteroides abscessus 3A-0	EIV38099	----------------------- G AE-L--IA----------K----
Mycobacteroides abscessus 4S-0	EIV12419	----------------------- G AE-L--IA----------K----
*Mycobacteroides abscessus MAB_*	ESV60011	----------------------- G AE-L--IV----------K----
*Mycobacteroides abscessus MAB_*	ETZ89174	----------------------- G AE-L--IA----------K----
Mycobacteroides abscessus subsp. abscessus	SIC19648	----------------------- G AE-L--IA----------K----
Mycobacteroides chelonae	WP_070918033	----------------------- T AE-L--IS----------K----
Mycobacteroides franklinii	WP_078336530	----------------------- S AE-L--I-----------K----
Mycobacteroides immunogenum	WP_064632605	----------------------- G AE-L--L-----------K----
Mycobacteroides salmoniphilum	WP_078329102	---------------------TA G AE-L--IA----------K----
Mycobacteroides saopaulense	WP_070910844	----------------------- G AE-L--IA----------K----
Mycolicibacter algericus	WP_083038288	E----------------A----- A EG-I---N---------------
Mycolicibacter heraklionensis	WP_064888463	E---------------------- A VP-I-T-S---------------
Mycolicibacter icosiumassiliensis	WP_067973496	E---------------------- A VP-I-T-S---------------
Mycolicibacter kumamotonensis	WP_083079355	E---------------------- A VP-I-T-N---------------
Mycolicibacter longobardus	WP_085264166	E---------------------- A -P-I-T-S---------------
Mycolicibacter senuensis	WP_085084579	E---------------------- A EG-I-T-S---------------
Mycolicibacter sinensis	WP_064855674	E---------------------- A VP-I-T-N---------------
Mycolicibacter terrae	WP_085259320	E---------------------- A VP-I-T-N---------------
Mycolicibacterium agri	WP_097941217	---------N-----------T- A SAK--T-H---------------
Mycolicibacterium aurum	WP_087023298	-----I----H------------ A SA-L-A-S---------------
Mycolicibacterium bacteremicum	WP_083054808	---------------------P- G SA-L---H---------------
Mycolicibacterium brumae	WP_090589784	-------S--A------T----- P AGAI-T-S---------------
Mycolicibacterium canariasense	WP_062657124	-----I----------------- A SA-L---S---------------
Mycolicibacterium celeriflavum	WP_083000590	----------------------- T SA-L---P--------N--A---
Mycolicibacterium chlorophenolicum	WP_048470893	---------T------------- A VA-M-R----------N--A---
Mycolicibacterium chubuense	WP_014816298	-------S--------------- S AP-M-K----------N--A---
Mycolicibacterium confluentis	WP_085151131	-------S------------C-- A VA-L-T-S---------------
Mycolicibacterium cosmeticum	WP_036399895	-----I----------------- A SA-L---S---------------
Mycolicibacterium diernhoferi	WP_073855042	-----I----H------------ A SA-L---A------Y--------
Mycolicibacterium doricum	WP_085191572	----------------------- A -A---T----------N-G----
Mycolicibacterium duvalii	WP_098002573	-------S--------------- A AP---R----------N------
Mycolicibacterium flavescens	WP_069413399	----------------------- S -P-L---P--------N--A---
Mycolicibacterium fortuitum	WP_025088118	----------------------- G AE-L--IA----------K----

Figure S43. Partial sequence alignment of a DEAD/DEAH box helicase protein showing a one amino acid deletion that is specific for members of the “Tuberculosis” clade.

Group	Species	Accession	Sequence (1–48)
“Tuberculosis” clade (9/9)	*Mycobacterium tuberculosis*	AIH58176	MTVILLRHARSTSNTAGVLAGRS [ ] GVDLDEKGREQATGLIDRIGDLPIR
	Mycobacterium bovis	WP_053905046	----------------------- [ ] -------------------------
	Mycobacterium canettii	WP_014001147	----------------------- [ ] -------------------------
	Mycobacterium caprae	APU26153	----------------------- [ ] -------------------------
	Mycobacterium microti	AMC59794	----------------------- [ ] -------------------------
	Mycobacterium mungi	OAQ19717	----------------------- [ ] -------------------------
	Mycobacterium orygis	EMT35584	----------------------- [ ] -------------------------
	Mycobacterium pinnipedii	PRH89939	----------------------- [ ] -------------------------
	Mycobacterium africanum	AMC64373	----------------------- [ ] -------------------------
Other *Mycobacteriaceae* (0/43)	*Mycobacterium alsense*	WP_083140456	--------G--A----------- E -----D------A------------
	Mycobacterium angelicum	WP_083114571	--------G--S-----I----- E -----D------A-------T----
	Mycobacterium arosiense	WP_083064981	--------G-------------- E -----D------D-----------Q
	Mycobacterium asiaticum	WP_036359527	---L----G-------------- E -----D------A---------S--
	Mycobacterium avium	WP_003872206	--------G-------------- D -----D------A------------
	Mycobacterium bohemicum	WP_085178821	--------G-------------A E ------R------------------
	Mycobacterium colombiense	WP_044485933	--------G--S----------- E -----D------D-----------L
	Mycobacterium conspicuum	WP_085232944	--------G-------------- E -----D------AA------E----
	Mycobacterium europaeum	WP_085239801	--------G-------------- E -----D---D--A------------
	Mycobacterium florentinum	WP_085220425	--------G--------I----- E -----D------A------------
	Mycobacterium gastri	WP_036416327	--------G--------I----- E -----D------D-----L----V-
	Mycobacterium genavense	WP_025735422	--------G-------------- E -----D------A------------
	Mycobacterium gordonae	WP_055579749	---L----G--------T----- E -----D------A------------
	Mycobacterium heidelbergense	WP_083072407	--------G-------------- E -----D------A------------
	Mycobacterium interjectum	WP_066913315	--------G-------------- E -----D------A------------
	Mycobacterium intracellulare	WP_064940122	--------G--S-----I----- E -----D------AA-----------
	Mycobacterium kansasii	ORB82848	--------G--------I----- E --N--D--Q---A-----F----V-
	Mycobacterium lentiflavum	WP_090603266	--------G--------I----- E -----D------V------------
	Mycobacterium lacus	WP_085161913	--------G-------AI----- E -----D---A--A-----V---RL-
	Mycobacterium leprae	AAA17180	--------G-------------A D -----DR-----V------AE----
	Mycobacterium lepraemurium	ATA28629	--------G-------------- E -----D------A------------
	Mycobacterium liflandii	WP_015356111	--------G--------I----- E -----D---Q--S----------V-
	Mycobacterium malmoense	WP_071509904	--------G--------I----- E -----D------A---------S--
	Mycobacterium mantenii	WP_083097745	--------G--S----------- E -----D--S--SA------------
	Mycobacterium marinum	WP_012394796	--------G--------I----- E -----D---Q--S----------V-
	Mycobacterium montefiorense	WP_108921772	--------G-------------- E -----D-----SV------------
	Mycobacterium nebraskense	WP_046186214	--------G-------------- E -----D---D--A------A-----
	Mycobacterium paraense	WP_085244008	--------G-------------- E -----D------A-----L------
	Mycobacterium paraffinicum	WP_073873080	--------G--S-----I----- E -----D---D--A------------
	Mycobacterium paraseoulense	WP_083172728	--------G-------------- E -----D---D--A------------
	Mycobacterium parmense	WP_085269493	---L----G-------------- E -----DR-----A-----V------
	Mycobacterium persicum	WP_083154173	--------G--------I----- E -----D------AA----L----V-
	Mycobacterium saskatchewanense	WP_085254056	--------G--------T----- E -----D------A------------
	Mycobacterium scrofulaceum	WP_067274418	--------G--------I----- E -----D---D--A------------
	Mycobacterium sherrisii	WP_069400417	--------G--------T----- E -----D------A-----L------
	Mycobacterium shigaense	WP_096440787	--------G-------------- E -----D------V---------R--
	Mycobacterium shinjukuense	WP_083046898	----------------------C A -----DN-----A---E---E----
	Mycobacterium simiae	WP_061555988	--------G--------T----- E -----D------A-----L------
	Mycobacterium szulgai	WP_085670465	--------G--S-----I----- E -----D------A-------T----
	Mycobacterium triplex	WP_036469759	--------G-------------- E -----D------A------------
	Mycobacterium ulcerans	WP_011740345	--------G--------I----- E -----D---Q--S----------V-
	Mycobacterium ulcerans str. Harvey	EUA89778	--------G--------I----- E -----D---Q--S----------V-
	Mycobacterium vulneris	WP_085292132	--------G--S----------- E -----D------E------------

Figure S44. Partial sequence alignment of a phosphoglycerate mutase protein showing a one amino acid deletion that is specific for members of the “Tuberculosis” clade.

Group	Species	Accession	10		54
**“Tuberculosis” clade (10/10)**	*Mycobacterium tuberculosis*	AUS51262	MTVTADRHLA	DK	REEFAVEDISTGIFASGYGQVGDGRSFSFHIEH
	Mycobacterium bovis	YP_009359501	----------	--	---------------------------------
	Mycobacterium canettii	WP_080605283	----------	--	---------------------------------
	Mycobacterium canettii CIPT 14	CCK60240	----------	--	---------------------------------
	Mycobacterium caprae	CEJ53301	----------	--	---------------------------------
	Mycobacterium microti	AMC59796	----------	--	---------------------------------
	Mycobacterium mungi	WP_003411107	----------	--	---------------------------------
	Mycobacterium orygis	EMT35586	----------	--	---------------------------------
	Mycobacterium pinnipedii	PRH89938	----------	--	---------------------------------
	Mycobacterium africanum	CCC27219	----------	--	---------------------------------
**Other *Mycobacteriaceae* (0/78)**	*Mycobacterium alsense*	WP_083140458	---I----T-		QN-------------------------------
	Mycobacterium aquaticum	ORA32459	--LA---EPG		YQ---I------VH---F-------T------R
	Mycobacterium arosiense	WP_083064970	-A------MT		QN------M----S------------------N
	Mycobacterium asiaticum	WP_036359521	--------T-		DH------MTA--Y----------------V--
	Mycobacterium avium	WP_099241892	-A------M-		QK------M-----------------------N
	Mycobacterium bohemicum	WP_085178822	---IV---TV		PD------L------------------------
	Mycobacterium colombiense	WP_007770783	-A--------		QN------M----S------------------N
	Mycobacterium conspicuum	WP_085232945	----V---V-		DTG-----MIA--------------L-------
	Mycobacterium europaeum	WP_085239800	-A-IV---S-		EK------M------------------------
	Mycobacterium florentinum	WP_085220426	--------T-		DK-------------------------------
	Mycobacterium gastri	WP_036416330	--------V-		NKQ--I--M--------------------R---
	Mycobacterium genavense	WP_025735421	---I----T-		DK---------------------------R---
	Mycobacterium gordonae	WP_055579751	----D--PT-		DQ------MTP--------H-----T-------
	Mycobacterium grossiae	WP_070351468	-S-V-N-DVD		HDQ-VI------LH---F-S----------V-
	Mycobacterium haemophilum	WP_047316458	--------A-		DN-----E-------------------------
	Mycobacterium hassiacum	WP_005628160	--LA-QPD-G		SR--VI---W--PH---F------------V-R
	Mycobacterium heidelbergense	WP_083072406	---IV---I-		EK-------------------------------
	Mycobacterium holsaticum	WP_069406411	--LA-EQD-G		HQ---I------VH---F-R---------R--R
	Mycobacterium houstonense	WP_066897918	--LA---E-C		SR---M------VH---F--------------R
	Mycobacterium interjectum	WP_066913317	---IV---A-		EK------L------------------------
	Mycobacterium intermedium	WP_069422089	--------I-		SN---I--ML---------V-------------
	Mycobacterium intracellulare	WP_041787055	-A------RT		Q-------M-----------------------N
	Mycobacterium kansasii	ARG71939	--------V-		NKH--I--M----------------T-------
	Mycobacterium komaniense	WP_090274058	--LA---AQG		AR--VI--MT--LH---F------------V-R
	Mycobacterium lacus	WP_085161912	--------V-		DH------MIA----------------------
	Mycobacterium lentiflavum	WP_090603269	---I----T-		HK-------------------------------
	Mycobacterium leprae	AAA17192	----V---V-		GN--T--E-------------------------
	Mycobacterium lepraemurium	ATA28630	-A--D---MT		QK------M-----------------------N
	Mycobacterium lepromatosis	WP_045843043	----V--NMV		KN--T--E-------------------------
	Mycobacterium mageritense DSM	CDO25465	--LS---E-G		SR---I------VH---F-------T------R
	Mycobacterium malmesburyense	WP_090344974	-SLA---A-G		ARD-VI--MT--LH---F------------V-R
	Mycobacterium malmoense	WP_065441981	-A-I----I-		ER------F--------------------R---
	Mycobacterium mantenii	WP_083097743	-A------T-		QK------M----S------------------N
	Mycobacterium nebraskense	WP_046186215	-A--V---V-		EK------V-----G------------------
	Mycobacterium neworleansense	WP_090510208	--LA---E-G		NR---M------VH---F--------------R
	Mycobacterium noviomagense	WP_083088902	---AE--QFS		SL--TI--MW---------H----------V-R
	Mycobacterium novocastrense	WP_067389197	--LA---A-G		AR--VI--MT--LY---F------------V-R
	Mycobacterium palustre	WP_085081191	--------T-		AT------LT-----------------------
	Mycobacterium paraense	WP_085101636	---IV---T-		EK-------------------------------
	Mycobacterium paraffinicum	WP_073873077	-A-IV--QT-		EK------M------------------------
	Mycobacterium paraseoulense	WP_083172729	-A-IV--Q--		EK------M------------------A-----
	Mycobacterium parmense	WP_085269494	---IV---IS		-K-----------S-------------------
	Mycobacterium peregrinum	WP_055114951	--LA---E-G		NR---M------VH---F--------------R
	Mycobacterium phlei	WP_061482905	--L---CE-G		AG-------I--VH---F--------------R
	Mycobacterium shigaense	BAX93152	----V---TS		DG-------------------------------
	Mycobacterium shinjukuense	WP_083046896	--------I-		DN---I--MW-----------------------
	Mycobacterium smegmatis	WP_003895582	---A--VA-S		HS---I---T--V----F------KT------R
	Mycobacterium talmoniae	WP_083342039	-------PV-		SR--VI--M--------F--------------R
	Mycobacterium triplex	WP_036469765	--------T-		DK-------------------------------
	Mycobacterium tusciae	ORB68495	--LA---E-G		SQ---I------VH---F--I---------V-R
	Mycobacterium ulcerans	WP_071498094	---A----T-		DKG--I--ML--------------H----R---
	Mycobacterium wolinskyi	WP_067858873	--LA-----G		DQK--I------VH---F-------T------R
	Mycolicibacillus trivialis	ODR11518	--LA---PQI		GQ--VI--M---V-G--F-A-------A-RVD-
	Mycolicibacter heraklionensis	WP_047321341	---VD-WQ--		CS--RI-E-W--A----F-E----------T-Q
	Mycolicibacter longobardus	WP_085264068	---AE-WQ--		CS--FL-E-W--A----F-E-------------
	Mycolicibacter minnesotensis	WP_083025105	---VE-WQ-V		CS--RI-E-W--A----F-E-----------QQ
	Mycolicibacter senuensis	WP_085081598	---AE-WQ--		CSD-L--E-W--A----F-----------R--Q
	Mycolicibacterium boenickei	WP_077741090	--LA---E-G		NR---M------VH---F--------------R
	Mycolicibacterium brisbanense	WP_029368313	--LA---EPG		YQ---I------VH---F-------T------R
	Mycolicibacterium celeriflavum	WP_083000633	--LA---E-G		SQ---I---T--VR---F------------V-R
	Mycolicibacterium chlorophenolicum	KMO75095	--LAT--VVT		P-G------T--VH---F--------------
	Mycolicibacterium flavescens	WP_069416415	--LA---E-G		AGQ-VL---T--VH---F-----------QV-R
	Mycolicibacterium fortuitum	WP_054602567	--LA---E-G		NR---M------VH---F-R------------R
	Mycolicibacterium goodii	WP_100519464	--LA---S-N		HS---I---T--VH---F------KT------R

Figure S45. Partial sequence alignment of a hypothetical protein CAB90_02390 showing a two amino acid insertion that is specific for members of the “Tuberculosis” clade.

			333		380
“Tuberculosis” clade (12/12)	*Mycobacterium tuberculosis*	AIH60817	TAVGTKDVIGAYAGLRPLIDTG	GAGV	QGRTADVSRDHAVFESPSGVIS
	Mycobacterium bovis	KFW16045	----------------------	----	----------------------
	Mycobacterium bovis AF2122/97	YP_009359617	----------------------	----	----------------------
	Mycobacterium bovis BCG	CUI12183	----------------------	----	----------------------
	Mycobacterium canettii	WP_080627841	----------------------	----	-D--------------------
	Mycobacterium canettii CIPT 14	CCK52218	----------------------	----	----------------------
	Mycobacterium caprae	CEJ53622	----------------------	----	----------------------
	Mycobacterium microti	WP_105799795	----------------------	----	----------------------
	Mycobacterium mungi	OAQ16979	----------------------	----	----------------------
	Mycobacterium orygis	EMT35517	----------------------	----	----------------------
	Mycobacterium pinnipedii	PRH92366	----------------------	----	----------------------
	Mycobacterium africanum	CCC27330	----------------------	----	----------------------
Other *Mycobacteriaceae* (2/>100)	*Mycobacterium alsense*	WP_083137520	--L-AG----------------	---F	E--------E---V-A------
	Mycobacterium saskatchewanense	WP_085254603	VSL-VG----------------	A--S	E-----L------V--NT----
	Mycobacterium angelicum	WP_083113552	-T-T-S----------------		E-----------I---------
	Mycobacterium arosiense	WP_083067335	--LTAT----------------		E--------E---V--A-----
	Mycobacterium asiaticum	WP_065036036	---S-S----S-----------		T----------------T----
	Mycobacterium avium	WP_084024130	--LAAT----------------		E--------E---V--A-----
	Mycobacterium branderi	WP_083133306	ITLDDS----------------		A-----------IV--D-----
	Mycobacterium celatum	WP_085168288	ITLDDS----T-----------		E-----------IV--D---V-
	Mycobacterium chimaera	WP_089151667	-GLTAT----------------		E--------E---V--A-----
	Mycobacterium colombiense	WP_064880506	-GLTAT----------------		E--------E---V--A-----
	Mycobacterium conceptionense	CQD21301	-RL-VS--R-------------		E-N------E---V--AT----
	Mycobacterium conspicuum	WP_085231261	S-L-PE----S-----------		E------------------AL-
	Mycobacterium europaeum	WP_085242268	V-L-PA----------------		E--------E---V--S--I--
	Mycobacterium florentinum	WP_085224170	--LRAT----------------		E--------E---V--------
	Mycobacterium fragae	WP_085199737	IRLD-G----------------		A------------V--------
	Mycobacterium gastri	WP_036411467	-KLQ-T----------------		E------------I----R---
	Mycobacterium genavense	WP_025735050	--LLAT----------------		E--------E---V--------
	Mycobacterium gilvum	WP_011893654	--LRHE--V-S-----------		T--------E---T--------
	Mycobacterium gordonae	WP_065043658	-T-SSS----S-----------		A-----------I----T----
	Mycobacterium haemophilum	WP_047314644	ATLR-T----------------		E------------V---C----
	Mycobacterium hassiacum	WP_005628371	VTLSES-----F------A---		E-----L--E-V-RV--A----
	Mycobacterium heckeshornense	WP_048890592	--L-PP----S-------V---		A------------V--------
	Mycobacterium heidelbergense	WP_083074413	A-L-AA----------------		E--------E---V-G------
	Mycobacterium holsaticum	WP_069404900	VS--PS----------------		A--------E---V-----L--
	Mycobacterium houstonense	WP_066897781	-DLT-A--R-S-----------		A-S------E---V--AG----
	Mycobacterium interjectum	WP_085202787	-SLSAA----------------		E--------E---L-A------
	Mycobacterium intermedium	WP_069421244	-T-TSS----S-----------		G---------------------
	Mycobacterium intracellulare	WP_064937169	-GLSAT----------------		E--------E---V--A-----
	Mycobacterium intracellulare s	AFS14106	-GLTAT----------------		E--------E---V--A-----
	Mycobacterium kansasii	WP_063471996	IKLQ-T----------------		E------------I--------
	Mycobacterium komaniense	WP_090277821	V-LRPS----------------		A--------E---V--------
	Mycobacterium kubicae	WP_085074571	--LQAT----------------		A---------------------
	Mycobacterium kyorinense	WP_065014644	-TLDAS----------------		A-H---I------V--TT----
	Mycobacterium lacus	WP_085161818	-T-TAT----------------		E---------------------
	Mycobacterium lehmannii	WP_094288395	V-LSPS----------------		A--------E---A--------
	Mycobacterium lentiflavum	WP_090603532	--LQPA----S-----------		E--------E---V---A----
	Mycobacterium leprae	WP_049769812	A-LRIT----S-V--------C		E--S------Y--V-L-FD---
	Mycobacterium lepraemurium	ATA28424	--LAAT----------------		E--------E---V--A-----
	Mycobacterium liflandii 128FXT	AGC63267	--LQ-S----------------		E------------I--------
	Mycobacterium llatzerense	WP_071289505	-SL-PD--L-------------		E-N----------V--GD--L-
	Mycobacterium malmesburyense	WP_090341494	V-L-PS----------------		A--------E---V--------
	Mycobacterium malmoense	WP_065444293	V-L-PA----------------		E--------E---V--A-----
	Mycobacterium mantenii	WP_083099795	-GLVAT----------------		E--------E---V--A-----
	Mycobacterium marinum	WP_012394991	--LQ-S----------------		E------------I--------
	Mycobacterium marseillense	WP_083020436	-GLTAT----------------		A--------E---V--S-----
	Mycobacterium microti	WP_101528625	SS-TVA----------------		A--------E---VA-ET----
	Mycobacterium montefiorense	WP_108921470	--LR-T--V-------------		A--------E---V--Q-----
	Mycobacterium moriokaense	WP_083157569	V-L-PD--------------A-		E--------E-R-V-----LL-
	Mycobacterium mucogenicum	WP_064861032	-SLRPD--R-------------		E-N----------V--GN--L-
	Mycobacterium nebraskense	WP_046184104	V-L-PA----------------		E--------E---V--A--IV-
	Mycobacterium neumannii	WP_094294127	V-LSPS----------------		A--------E---V--------
	Mycobacterium neworleansense	WP_090509969	-DLT-A--R-T-----------		E-N------E---V--AG----
	Mycobacterium noviomagense	WP_083089022	--LRPS----S-------V---		A-----I--E---VD-------
	Mycobacterium novocastrense	WP_067389036	V-LSPS----------------		A--------E---V--------
	Mycobacterium obuense	WP_046363408	-SLRPD--V-S-----------		A--------E---T---N--L-
	Mycobacterium palustre	WP_085078183	SRL--A--------------S-		E--------E---V--S--L--
	Mycobacterium paraense	WP_085101773	--L--G--L-------------		E--------E---I--S-----
	Mycobacterium paraffinicum	WP_073875480	V-L-PA----------------		E--------E---V--A--I--
	Mycobacterium parafortuitum	WP_083146628	--LRPE--R-S-----------		T--------E---T--------
	Mycobacterium paraintracellulare	WP_014384524	-VLTAT----------------		E--------E---V--A-----
	Mycobacterium paraseoulense	WP_083172793	A-L-PA----------------		E--------E---V--A--I--

**Other *Mycobacteriaceae* (2/>100)**	*Mycobacterium parmense*	WP_085271387	--LKAA--------------S-	---------E---V--A-----
	Mycobacterium peregrinum	WP_064878018	-DLT-E--R-------------	--N------E---V--A-----
	Mycobacterium persicum	ORB37743	-TLQ-T----------------	E------------I--------
	Mycobacterium phlei	WP_061482149	--LTEA--H-------------	E-----I--E---T--E-----
	Mycobacterium phlei DSM 43239	KXW64819	--LTEA--H-------------	E-----I--E---T--E-----
	Mycobacterium phlei RIVM601174	EID18354	--LTEA--H-------------	E-----I--E---T--E-----
	Mycobacterium porcinum	WP_069424759	-DLTNS--R-------------	E-N------E---V--A-----
	Mycobacterium rhodesiae	WP_014212674	V-LQPD--------------A-	G--------E-R-D-----L--
	Mycobacterium riyadhense	WP_085250016	--LQ-A----------------	E------------V--A-----
	Mycobacterium rufum	KGI68841	--LTPS--L-------------	G--------E---T---T--L-
	Mycobacterium rutilum	SEH74743	--LTPA--V---S-----V-A-	A------------TV-------
	Mycobacterium scrofulaceum	WP_067269534	V-L-PA----------------	E--------E---V--A-----
	Mycobacterium senegalense	WP_019346324	-EL-VS--R-------------	E-N------E---V--AT----
	Mycobacterium septicum	WP_044523462	-ELT-A--R-------------	E-K------E---V--A-----
	Mycobacterium setense	WP_064875022	-DLT-A--Q-------------	E-S---I--E---V--A--A--
	Mycobacterium sherrisii	WP_069402783	RT-T-A----------------	T--------E---V--ET----
	Mycobacterium shigaense	WP_096441043	-SLTAAE-----------V---	E--------E---V--------
	Mycobacterium shimoidei	WP_069396233	ITLEPS----------------	G-H----------V--E-----
	Mycobacterium shinjukuense	WP_083046532	SP-AAS--V-------------	E------------V--------
	Mycobacterium simiae	WP_061556113	-S-TVA----------------	A--------E---VA-ET--L-
	Mycobacterium smegmatis	WP_011729740	-TLTPA--R-T---------A-	E-----L--E---V---D--L-
	Mycobacterium sphagni	WP_094483305	--LT-A--T-------------	E---S-L--E---I--D---F-
	Mycobacterium szulgai	WP_068026911	--LQAT----------------	A---------------------
	Mycobacterium talmoniae	PQM48062	--L-AA----S-----------	---------E---V--E---L-
	Mycobacterium thermoresistibile	WP_003924647	--L-PD---------------P	G-----I------V--DT-LF-
	Mycobacterium triplex	WP_036469954	--LRAT----------------	E--------E---V--------
	Mycobacterium tusciae	WP_006240995	V-L--D--------------A-	E--------E-R-V------L-
	Mycobacterium ulcerans	WP_096370475	--LQ-S----------------	E------------I--------
	Mycobacterium ulcerans str. Harvey	EUA91412	--LQ-S----------------	E------------I--------
	Mycobacterium vulneris	WP_065462195	-DLTNS--R-------------	E-N------E---V--A-----
	Mycobacterium wolinskyi	WP_085149065	--LTPA--R-------------	A--------E---V---N---G
	Mycobacterium xenopi	WP_085196116	S-LEPSE---S-------V---	G--------E--IV--------
	Mycobacterium xenopi 3993	EUA30952	S-LEPSE---S-------V---	G--------E--IV--------
	Mycobacterium xenopi 4042	EUA65815	S-LEPSE---S-------V---	G--------E--IV--------
	Mycobacteroides abscessus subsp. abscessus	SHW55405	-SL-PD--R-------------	E-N----------V--GN-LL-
	Mycolicibacillus trivialis	WP_085110169	-TLSRP----S-------V-A-	E--S--I------I-AD-----
	Mycolicibacter algericus	WP_083036216	--L-AG----R-----------	A-H------N---V--GT--L-
	Mycolicibacter arupensis	WP_109370688	--LS-A--R-------------	E--------N---V--E---F-
	Mycolicibacter engbaekii	WP_085128625	--LDAG--R-------------	S-H------N---V--G---F-
	Mycolicibacter heraklionensis	OBI03140	--LS-A--R-------------	E--------N---V--ET--L-
	Mycolicibacter hiberniae	WP_085135919	--LDAS--R-------------	S-H------N---V--G---F-
	Mycolicibacter icosiumassiliensis	WP_067974189	I-LS-A--R-------------	E--------N---V--E---L-
	Mycolicibacter kumamotonensis	WP_065288790	A--DVG----S-----------	---------N---V--E---L-
	Mycolicibacter longobardus	WP_109561305	--LS-E--R-------------	E--------N---V--E---L-
	Mycolicibacter minnesotensis	ORB04565	--LS-A--R-------------	---------N---V--E---F-
	Mycolicibacter nonchromogenicum	WP_085136990	--LS-A--R-------------	E--------N---V--ET--L-
	Mycolicibacter senuensis	WP_085082827	--L-AG----C---------A-	A-H------N---V--GT--L-
	Mycolicibacter sinensis	WP_064855186	--L--G----S---------A-	A--------N---V--E---F-
	Mycolicibacter terrae	WP_085261089	-TL-GG----S-----------	---------N---V--A---L-
	Mycolicibacterium aromaticivorans	WP_036344571	--LT-A--K-------------	E---S-L--E---I--SN--F-
	Mycolicibacterium boenickei	WP_097926343	-DLTPD--R-------------	E-N------E---V--A--L--
	Mycolicibacterium brumae	WP_090591906	-KLS-D--L-------------	G------------I--E--LL-
	Mycolicibacterium canariasense	WP_062658004	-EL-HG--L------------D	T--------E---T------L-
	Mycolicibacterium celeriflavum	WP_083001160	V-LRPS----------------	A--------E---V--------
	Mycolicibacterium chlorophenolicum	WP_048471771	--L-PS--L-------------	G--------E---T---T--L-
	Mycolicibacterium chubuense	WP_048419178	--L-PS--L-------------	G--------E---T---T--L-
	Mycolicibacterium chubuense NB	AFM18026	--L-RD--V-------------	A--------E---V--------
	Mycolicibacterium conceptionense	OBB07156	-EL-VS--R-------------	E-N------E---V--AT----
	Mycolicibacterium confluentis	WP_085150680	V--T-D----S---------A-	E-----I------I-A---AVN
	Mycolicibacterium cosmeticum	WP_036396790	-QLHPG--R-------------	T--------E--IT------L-
	Mycolicibacterium duvalii	WP_098004525	-S--PA----------------	S--------E---T--------
	Mycolicibacterium elephantis	WP_083043692	VS--PA----------------	A--------E---S--------
	Mycolicibacterium farcinogenes	WP_036394263	-EL-VS--R-------------	E-N------E---V--AT----
	Mycolicibacterium flavescens	WP_069412841	--LTSA--V---------V-A-	A------------TV-------
	Mycolicibacterium fortuitum	WP_061264861	-GLTPA--R-------------	E-H------E---V--A-----
	Mycolicibacterium fortuitum subsp. fortuitum	AMD56546	-GLTPA--R-------------	E-H------E---V--A-----
	Mycolicibacterium goodii	WP_100515925	-TLTEA--R-S-------V-A-	E-----L--E---V---N-LL-

Figure S46. Partial sequence alignment of the glycerol-3-phosphate dehydrogenase protein showing a four amino acid insertion that is specific for members of the "Tuberculosis" clade and is absent from most other *Mycobacteriaceae*.

Group	Species	Accession	298		355
"Tuberculosis" clade (9/9)	*Mycobacterium tuberculosis*	AIH99286	GVGEVGYLITGVKDVRQSKVGDTVTS	LSR	ARGAAAEALTGYREPKPMVYSGLYPVDGS
	Mycobacterium bovis	YP_009359775	--------------------------	---	-----------------------------
	Mycobacterium canettii	WP_015293669	--------------------------	---	-----------------------------
	Mycobacterium caprae	APU26388	--------------------------	---	-----------------------------
	Mycobacterium microti	AMC60094	--------------------------	---	-----------------------------
	Mycobacterium mungi	WP_064319597	-----------------------L--	---	-----------------------------
	Mycobacterium orygis	WP_003412343	--------------------------	---	-----------------------------
	Mycobacterium pinnipedii	PRH92206	--------------------------	---	-----------------------------
	Mycobacterium africanum	WP_031666933	--------------------------	---	-----------------------------
Other *Mycobacteriaceae* (0/42)	*Mycobacterium asiaticum*	OBK18153	-------------------------T		--NG-K-----------------------
	Mycobacterium conceptionense	CQD21896	-------------------------T		--KG-T-----------------------
	Mycobacterium europaeum	CQD11833	-------------------------T		--HG-T-----------------------
	Mycobacterium florentinum	WP_085221212	-------------------------T		--HG-K-----------------------
	Mycobacterium gastri	WP_036419196	-------------------------T		V-NG-K-----------------------
	Mycobacterium gordonae	WP_065133107	-------------------------T		--HG-K-----------------------
	Mycobacterium haemophilum	WP_054880917	-------------------------T		--HG-T-----------------------
	Mycobacterium hassiacum	WP_085977508	-------------------------T		--NG---P------------A--------
	Mycobacterium hassiacum DSM 44	EKF23112	-------------------------T		--NG---P------------A--------
	Mycobacterium insubricum	WP_083028919	-------------------------T		--HG-T---------R-------------
	Mycobacterium intermedium	WP_069417428	-------------------------T		--NG-K-----------------------
	Mycobacterium kansasii	KZS77658	-------------------------T		V-NG-K-----------------------
	Mycobacterium kansasii 732	EUA13120	-------------------------T		V-NG-KA----------------------
	Mycobacterium kansasii 824	EUA01621	-------------------------T		V-NG-K-----------------------
	Mycobacterium lacus	WP_085162908	-------------------------T		--HG-K-----------------------
	Mycobacterium litorale	WP_078021910	-------------------------T		--HG-TD----------------------
	Mycobacterium nebraskense	KKC03420	-------------------------T		--HG-T-----------------------
	Mycobacterium palustre	ORW19959	-------------------------T		--HG-T-----------------------
	Mycobacterium paraense	WP_085244185	-------------------------T		--HG-T-----------------------
	Mycobacterium persicum	WP_089025285	-------------------------T		V-HG-K-----------------------
	Mycobacterium phlei	WP_003890450	-------------------------T		--NG-TDP---------------------
	Mycobacterium rhodesiae	WP_050950587	-------------------------T		--HG-T-----------------------
	Mycobacterium rhodesiae JS60	EHB53837	-------------------------T		--HG-T-----------------------
	Mycobacterium saskatchewanense	ORW65887	-------------------------T		--NG-K-----------------------
	Mycobacterium shinjukuense	WP_083046349	-------------------------T		--NG-T-----------------------
	Mycobacterium sphagni	WP_094481366	-------------------------A		--HG-T-----------------------
	Mycobacterium szulgai	ORX13727	-------------------------T		--HG-K-----------------------
	Mycolicibacillus trivialis	WP_085108973	-------------------------T		--HG-------------------------
	Mycolicibacter algericus	WP_083036319	-------------------------T		---G-------------------------
	Mycolicibacter arupensis	KKC01354	-------------------------T		---G-T-----------------------
	Mycolicibacter engbaekii	ORV42823	-------------------------T		---G-T-----------------------
	Mycolicibacter heraklionensis	KL027872	-------------------------T		---G-T-----------------------
	Mycolicibacter hiberniae	WP_085134854	-------------------------T		---G-T-----------------------
	Mycolicibacter kumamotonensis	OBY31474	-------------------------T		---G-T-----------------------
	Mycolicibacter minnesotensis	WP_083022314	-------------------------T		---G-TQ----------------------
	Mycolicibacter nonchromogenicus	ORW16620	-------------------------T		---G-T-----------------------
	Mycolicibacter senuensis	WP_085083582	-------------------------T		---G-T-----------------------
	Mycolicibacter sinensis	AEF35508	-------------------------T		---G-------------------------
	Mycolicibacterium aromaticivorans	WP_051660447	--------------------------		--HG-T-----------------------
	Mycolicibacterium aurum	WP_048633168	--------------------------		--NG-T-----------------------
	Mycolicibacterium brumae	WP_090593481	--------------------------		--KG-T-----------------------
	Mycolicibacterium confluentis	WP_109788561	-------------------------T		--RG-T-----------------------

Figure S47. Partial sequence alignment of the GTP-binding protein LepA showing a three amino acid insertion that is specific for members of the "Tuberculosis" clade.

Group	Species	Accession	10		58
“Tuberculosis” clade (9/9)	*Mycobacterium tuberculosis*	AUS51933	VRLGDHLDFSNGHTS	GHTS	PASEPGGRYPVYGANGVIGYSAQHNARGPL
	Mycobacterium bovis	WP_105537259	---------------	----	------------------------------
	Mycobacterium canettii	WP_014001408	---------------	----	------------------------------
	Mycobacterium caprae	APU26688	---------------	----	------------------------------
	Mycobacterium microti	AMC60473	---------------	----	------------------------------
	Mycobacterium mungi	OAQ19450	---------------	----	------------------------------
	Mycobacterium orygis	EMT35024	---------------	----	------------------------------
	Mycobacterium pinnipedii	PRH92079	---------------	----	------------------------------
	Mycobacterium africanum	WP_031668551	---------------	----	------------------------------
Other *Mycobacteriaceae* (0/46)	*Mycobacterium alsense*	WP_083138319	-T-----E-R--TSP		-RRA-D--FR------A---A-----T---
	Mycobacterium angelicum	WP_083111509	----------A-GAP		-PRI-HE-F-----------TSEC------
	Mycobacterium asiaticum	WP_065156310	-----Q-E-RS-RSA		-ERNTH-PFA---S------TT---TA--V
	Mycobacterium colombiense	WP_044484994	--E-----T-SSA		-PRDSD-SV-------A---A-A---T---
	Mycobacterium colombiense CECT	EJO87374	--E-----T-SGA		-PRDSD-SV-------A---A-A---T---
	Mycobacterium conspicuum	ORV34068	M-----------S-L		RMR-SQ----I-----A---APE-------
	Mycobacterium europaeum	WP_085242021	-----V----SSA		-RRA-R--FR------A---CNEP--A---
	Mycobacterium florentinum	WP_085227122	--EY---RT- -		--RV-D--F-------A---A--S--S---
	Mycobacterium gastri	WP_036412061	-------E--A-DVL		QVRLAA----------T---TT-C-TC---
	Mycobacterium gordonae	WP_065131755	ME----V--AA-S-P		-IRATE-CH-I-----P--FTT-R------
	Mycobacterium haemophilum	WP_047315388	-----C------R--		--R-S--------------A-----S---
	Mycobacterium heidelbergense	WP_083072716	---EY----S-TSP		-HRAS---FR------A---A-ER--S---
	Mycobacterium interjectum	WP_066909800	-------E----TPP		-PRA-D-CFR------A---A-----A---
	Mycobacterium intermedium	WP_069420349	-K---Q-ELRS-KSP		-RRAKT-P--I---------A-E---N---
	Mycobacterium kansasii	WP_103802145	-K-------AH-RAL		-TRLSD-GF--C----I---AD-P------
	Mycobacterium kansasii 732	EUA09250	M--------AH-RAL		-TRLSDAGF--C----I---AD-P------
	Mycobacterium kansasii 824	EUA02751	MK-------AH-RAL		-TRLSD-GF--C----I---AD-P------
	Mycobacterium kubicae	WP_085073226	-T--EC---RT-SSA		--RAAT--FA----------APDC--S---
	Mycobacterium lacus	WP_085158237	--------AS-NV-		--RGSD--------------A-E-------
	Mycobacterium lentiflavum	CQD11302	--EY---RT-SGA		-VRV-D--F-------A---A--S--S---
	Mycobacterium liflandii	WP_015355299	----E--E-RS-SC-		-PRRAR--V-------P--FA-E--VS---
	Mycobacterium malmoense	WP_083010034	-K-------R--SGL		-GRVSD--FR------P---A-----S---
	Mycobacterium mantenii	WP_083096444	--E-----T-SRA		PRASD--F-------A---A-E---S---
	Mycobacterium marinum	WP_020724791	----E--E-RS-SC-		-PRRAR--V-------P--FA-E--VS---
	Mycobacterium marinum E11	CDM76031	M---E--E-RS-SC-		-PRRAR--F-------P--FA-E--VS---
	Mycobacterium marinum M	ACC40410	M---E--E-RS-SC-		-PRRAR--V-------P--FA-E--VS---
	Mycobacterium marseillense	WP_095577480	--E-----S-SGA		-VRA-N--F-------A--SA-R---G---
	Mycobacterium microti OV254	PLV54014	M-EY----T- A		-VRVSD--F----------FT-RP------
	Mycobacterium montefiorense	WP_108920030	--EY----T-SR-		--RI-D--F-----------A--P--S---
	Mycobacterium nebraskense	WP_046183725	---Q-V--T-SSA		-RRS----FR------A---A-EP--A---
	Mycobacterium palustre	WP_085081345	--EF---A--GGL		--RVA--AVR---------HA--P--S---
	Mycobacterium paraense	WP_085102799	------E----TSP		-RRV-D-CFR---S--A---A-----A---
	Mycobacterium paraffinicum	WP_073877403	-------GS-SRA		-RGA----IR------A---A-E---A---
	Mycobacterium paraseoulense	WP_083173907	-----L-GS-SSA		-PGV---GFR------A---A-ER--A---
	Mycobacterium pseudoshottsii	WP_086085013	----E--E-RS-SC-		-PRRAR--VS------P--FA-E--VS---
	Mycobacterium saskatchewanense	WP_085257689	-----Q--S-KAL		-RGH-T--FR------P---A--R--S---
	Mycobacterium scrofulaceum	WP_067269028	-------GS-GRA		-RQTQ---FR------S---A-E---S--V
	Mycobacterium sherrisii	WP_069400921	--E----HA-SGA		-VRV-D-PF-------A--FT-RP------
	Mycobacterium shinjukuense	WP_083050178	------------RA-		-VR-S---------------A-E-------
	Mycobacterium simiae	WP_061560180	--EY----T-SGA		-VRVAD--F----------FT-RP------
	Mycobacterium szulgai	WP_085674355	-------G--A-GAP		-PRTGDE-F-------P---ASEC------
	Mycobacterium triplex	CDO87998	--EY---RT-SGA		--RV-D--F-------T---A-RP--S---
	Mycobacterium ulcerans	WP_096371185	----E--E-RS-SC-		-PRRAR--V-------P--FA-E--VS---
	Mycobacterium ulcerans str. Harvey	EUA92149	----E--E-RS-SC-		-PRRAR--V-------P--FA-E--VS---
	Mycobacterium ulcerans subsp. shinshuense	BAV42262	M---E--E-RS-SC-		-PRRAR--V-------P--FA-E--VS---
	Mycobacterium vulneris	WP_085290379	--E----RT-SSA		-PRDS---V-------A---A-V---T---

Figure S48. Partial sequence alignment of the type I restriction/modification system specificity determinant HsdS protein showing a four amino acid insertion that is specific for members of the “Tuberculosis” clade.

Clade	Species	Accession	42		81
"Tuberculosis" clade (8/8)	*Mycobacterium tuberculosis*	AIH52525	AAKRRHLYYVRPL		DGHPVARVDRKTDRAADSLPVAGVLGE
	Mycobacterium bovis	KA003028	-------------		--------------------------
	Mycobacterium canettii	WP_014001409	-------------		--------------------------
	Mycobacterium microti	AMC60474	-------------		--------------------------
	Mycobacterium mungi	OAQ19449	-------------		--------------------------
	Mycobacterium orygis	EMT35025	-------------		--------------------------
	Mycobacterium pinnipedii	PRH92080	-------------		--------------------------
	Mycobacterium africanum	CCC27837	-------------		--------------------------
Other *Mycobacteriaceae* (0/52)	*Mycobacterium alsense*	WP_083138320	-----YG---W--	LM	--EL-----L-A-------R-V-AFA-
	Mycobacterium angelicum	WP_083111510	R--YG---W--	LL	--QL-----L-A----NT-R-V-AF--
	Mycobacterium arosiense	WP_083063798	----QYG---W--	LM	--RL-----L-A----NT-R-L-AF--
	Mycobacterium asiaticum	OBI84659	--Q-QYG---W--	LL	--QL-----L-A-----T-R-V-AF--
	Mycobacterium asiaticum DSM 44	ORA15861	--Q-QYG---W--	LL	---L-----L-A----ET-R-V-AF--
	Mycobacterium avium complex	WP_042912564	----QYG---W--	LM	--RL-----L-A--P--T-R-V-TFA-
	Mycobacterium colombiense	WP_040631446	----QYG---W--	LM	--RL-----L-A-------R-L-AF--
	Mycobacterium colombiense CECT	EJO87376	----QYG---W--	LM	--RL-----L-A-------R-L-AF--
	Mycobacterium conspicuum	WP_085235929	----QYG---W--	LL	--RL-----L-A-----A-R-V-AF--
	Mycobacterium europaeum	CQD21580	--Q--YG---W--	LM	--QL-----L-A---G-T-R-V-AF--
	Mycobacterium florentinum	WP_085226631	-----YG---W--	LV	--QLM----L-A--T-GT-R-V-AF--
	Mycobacterium gastri	WP_036412060	----QYG---W--	LL	--QL-----L-A----K--H-V-AF--
	Mycobacterium genavense	WP_036467565	-----YG---W--	LV	--VLM----L-A-----T-R-V-AF--
	Mycobacterium gordonae	WP_055580482	----QYG---W-F	LL	N-EL-----L-A--GT-T-Q-L-AF--
	Mycobacterium heidelbergense	WP_083072876	----QYG---W--	LL	N-EL-----L-A---S---R-V-AF--
	Mycobacterium interjectum	WP_066909794	--Q--YG---W--	LM	--RL-----L-A---G---R-L-AF--
	Mycobacterium intracellulare	WP_036459319	----QYG---W--	LM	--RL-----L-A--P----R-V-AFA-
	Mycobacterium intracellulare M	ETZ28639	----QYG---W--	LM	--RL-----L-A--P--T-R-V-TFA-
	Mycobacterium intracellulare s	AGP65054	----QYG---W--	LM	--RL-----L-A--P----R-V-AFA-
	Mycobacterium kansasii	ORB86476	-P--HYG---W--	LL	--QL-----L-A-----T-R-V-AF--
	Mycobacterium kansasii 662	EUA21148	-P--QYG---W--	LL	--QL-----L-A-----A-R-V-AFS-
	Mycobacterium kansasii 732	EUA09545	-PQ-QYG---W--	LL	--RL-----L-A-----A-R-V-AF--
	Mycobacterium kubicae	WP_085073228	----QYG---W--	LL	--QL-----L-A-----T-Q-V-AFA-
	Mycobacterium lacus	WP_085158235	-S---YG---W--	LL	--QL-----L-A-----T-R-L-AF--
	Mycobacterium lentiflavum	CQD11296	-RQ--YG---W--	LV	--ELR----L-A-----T-R-V-AF--
	Mycobacterium malmoense	WP_083010067	----QYG---W--	LL	--QL-----L-A---TNT-R-V-AF--
	Mycobacterium mantenii	WP_083096447	--Q--YG---W--	LM	--RL-----L-A---VN--R-L-AF--
	Mycobacterium marseillense	WP_095577482	----QYG---W--	LM	--RL-----L-A--S----R-I-AFA-
	Mycobacterium montefiorense	GBG35765	----QYG---W--	LV	--ELR----L-A-----T-R---AF--
	Mycobacterium nebraskense	WP_046183735	----QYG---W--	LM	--QL-----L-A---G-T-R-M-AF--
	Mycobacterium palustre	WP_085081392	-----YG---W--	LM	--RL-----L-A----N--K-L-AF--
	Mycobacterium paraense	WP_085102795	----QYG---W--	LM	--RL-----L-A---G-A-R-L-AF--
	Mycobacterium paraffinicum	WP_073877488	----QYG---W--	LM	--RL-----L-A-----T-R-M-AF--
	Mycobacterium paraintracellulare	WP_014385399	----QYG---W--	LM	--RL-----L-A--P----R-V-AFA-
	Mycobacterium parascrofulaceum	EFG73799	-D--QYG---W--	LM	--RL-----L-A---G-T-R-V-AF--
	Mycobacterium paraseoulense	WP_083173954	----QYG---W--	LM	---L-----L-A---G-T-R-L-AF--
	Mycobacterium parmense	ORW62516	--R--YG---W--	LL	--QL-----L-A----GT-R-V-AFS-
	Mycobacterium persicum	WP_083153394	----QYG---W--	LL	--QL-----L---------R-L-AF--
	Mycobacterium riyadhense	WP_085248607	-----YG---W--	LL	--QL-----L-A----NT-R-L-AF--
	Mycobacterium saskatchewanense	WP_085257691	-HQ--YG---W--	LL	---L-----L-A--T-NV-Q-V-AF--
	Mycobacterium scrofulaceum	WP_083175080	-H--QYG---W--	LM	--RL-----L-A---G-T-R-L-AF--
	Mycobacterium shigaense	BAX91927	-S--QYG---W--	LM	---L-----L-A--V--T-R-V-AFA-
	Mycobacterium szulgai	WP_085674353	-N---YG---W--	LL	---L-----L-A----NA-R-V-AF--
	Mycobacterium triplex	ORX00985	-----YG---W--	LV	--ELR----L-A-----T-R-V-AF--
	Mycobacterium ulcerans	WP_011740205	--Q-QYG------	LL	--QL-G---L-A--ST-T-L-V-AFA-
	Mycobacterium ulcerans str. Harvey	EUA92184	--Q-QYG------	LL	--QL-G---L-A--ST-T-L-V-AFA-
	Mycobacterium vulneris	WP_085290377	----QYG---W--	LM	--RL-----L-A----N--R-L-AF--
	Mycobacteroides abscessus	WP_005057141	----QYG---W-F	LL	--QL-G---L-A----GT-N-V-AFA-
	Mycobacteroides abscessus subsp. abscessus	SHW95453	----QYG---W-F	LL	--QL-G---L-A----GT-N-V-AFA-
	Mycobacteroides abscessus subsp. massiliense	SKM20159	----QYG---W-F	LL	--QL-G---L-A----GT-N-V-AFA-
	Mycobacteroides chelonae	WP_109549529	-P--QYG---W-F	LL	---L-G---L-A----GT-N-I-AFA-
	Mycobacteroides franklinii	OHU18268	-P---YG---W-F	LL	---L-G---L-A----GT-N-V-AFA-

Figure S49. Partial sequence alignment of the hypothetical protein IQ38_12515 showing a two amino acid deletion that is specific for members of the "Tuberculosis" clade.

			1311		1359
**“Tuberculosis” clade (11/12)**	*Mycobacterium tuberculosis*	AIH25735	FEGELPRLFVVTRQAQIVKPHD	SGE	RANLEQAGLRGLLRVISSEHPMLR
	Mycobacterium africanum MAL010	KBG23920	----------------------	---	------------------------
	Mycobacterium bovis	PRI07666	----------------------	---	------------------------
	Mycobacterium bovis AF2122/97	YP_009360318	----------------------	---	------------------------
	Mycobacterium bovis BCG	AMC52014	----------------------	---	------------------------
	Mycobacterium canettii	WP_014001497	----------------------	---	------------------------
	Mycobacterium caprae	WP_075744572	----------------------	---	------------------------
	Mycobacterium microti	AMC60667	----------------------	---	------------------------
	Mycobacterium mungi	OAQ17709	LP--P--------S-AS-L-S-		L-----------M---D----H-G
	Mycobacterium orygis	WP_003414854	----------------------	---	------------------------
	Mycobacterium pinnipedii	WP_105826475	----------------------	---	------------------------
	Mycobacterium africanum	WP_003910676	----------------------	---	------------------------
**Other *Mycobacteriaceae* (0/81)**	*Mycobacterium angelicum*	WP_083111679	L---------------Q-RSD-		EL--------------G----L--
	Mycobacterium asiaticum	WP_065036022	T---A---Y----D--K-L-D-		VP----G---------GA---H-H
	Mycobacterium bohemicum	WP_085180269	IQ--A---Y----S--T-L-E-		VP----G---------GA---H-H
	Mycobacterium branderi	WP_083134348	AP--S--VYL---N--V-LAG-		QP----------M---GT---H--
	Mycobacterium caprae	WP_105812636	LP--P--------S-AS-L-S-		L-----------M---D----H-G
	Mycobacterium celatum	WP_085168573	AP--S--VYL---N--V-LAG-		QP----------M---GT---HV-
	Mycobacterium conceptionense	CQD23689	IN------Y----N--T-VAG-		V---D--E----F---GT---H-A
	Mycobacterium fragae	WP_085200231	IP--A---Y----N--T-LAG-		-P----------M---GT---H--
	Mycobacterium gastri	ORV65917	IM--A---Y----N--T-LAA-		SP--------------GA---H-H
	Mycobacterium gordonae	OBJ79446	I--QTA--Y-M--N--T-LAD-		C-----G----------A---H-K
	Mycobacterium grossiae	WP_070353719	LP--A---Y----D-AT-R-DE		-----H---------VDA---H-S
	Mycobacterium haemophilum	WP_047313877	IL-DV---Y----S--T-LAD-		C-----G---------GA---H--
	Mycobacterium houstonense	WP_066899918	IR------Y----N--T-LAG-		V------E----I---GT---H-A
	Mycobacterium interjectum	WP_084454465	LSAQP---Y---------L-D-		QP--V-----------GA-N-Q--
	Mycobacterium intermedium	WP_069418873	L---------------HARSD-		QL--D-L-----I---G--V-LMQ
	Mycobacterium kansasii	KZS65402	IM--A---Y----N--T-LAG-		SP--------------GA---H-H
	Mycobacterium kyorinense	WP_045373732	AP--S--VYL---N--V-LAG-		QP----------M---GT---H--
	Mycobacterium lacus	WP_085157743	L---------------V-R-K-		QV-------------------L--
	Mycobacterium leprae	WP_010908211	LP--R--------N-ASI-TG-		L-----V-----I---D----Y-S
	Mycobacterium lepromatosis	WP_082082362	LP--R--------N-ASI-TG-		L-----V-----M---D----Y-S
	Mycobacterium liflandii	WP_015355162	L---------------R-R-E-		EI--------------G----L-Q
	Mycobacterium litorale	WP_078019616	T---A---Y----N--T-LAD-		-P--------------GA-Y-Q--
	Mycobacterium mageritense	WP_085980454	IN------Y----N--T-VAG-		V---D--E----F---GT---H-A
	Mycobacterium malmesburyense	WP_090342510	IS--P--VY----N--C-LAG-		-P----------V---G----H-N
	Mycobacterium malmoense	WP_083012525	LS-QP---Y----R----L-D-		-P--V-----------GA-N-Q-Q
	Mycobacterium mantenii	WP_083097797	IP------Y----N--Q-LAG-		V-----------M---GT---H--
	Mycobacterium marinum	WP_094357877	L---------------R-R-E-		EI--------------G----L-Q
	Mycobacterium monacense	WP_083045080	VP--P-----L-HN--T-LAD-		V-----------M--LAM---A--
	Mycobacterium neumannii	WP_094294952	LS--P---Y----D--A-GTG-		-P---LG-----V---GT---H--
	Mycobacterium neworleansense	WP_090518389	MS------Y----N--T-VAG-		V---D--E----I---GT---H-S
	Mycobacterium noviomagense	WP_083089890	IS--S---Y----N--T-LAG-		-P--------------GT---H--
	Mycobacterium paraseoulense	ORB33916	IP------Y----N--T-VAS-		VP---------F----GT---H--
	Mycobacterium parmense	WP_085269581	IQ--A---Y----G--N-LSE-		V-----G---------GA---H-H
	Mycobacterium peregrinum	OBF40515	MS------Y----N--T-VAG-		V---D--E----I---GT---H-S
	Mycobacterium persicum	WP_083153430	L-----------------RSD-		Q---------------G----L--
	Mycobacterium pseudoshottsii J	GAQ32150	L---------------R-R-E-		EI--------------G----L-Q
	Mycobacterium rhodesiae	WP_083120382	TA--P---H----N--T-LAD-		-P--------------GA-Y-Q--
	Mycobacterium riyadhense	WP_085252257	IQ--A--------D--K-L-D-		VP----G---------GA---H-H
	Mycobacterium saskatchewanense	WP_085253693	IQ--A---V-I--N--T-L-E-		VP--D-G---------GA---H-H
	Mycobacterium setense	OBB21522	MS------Y----N--T-LAG-		V---D--E----I---GT---H-S
	Mycobacterium szulgai	WP_085670451	L---------------Q-LSD-		EL--------------G----L--
	Mycobacterium talmoniae	WP_071028710	LP----A-Y----N--T-V-G-		V-----------I---AA-Y-H--
	Mycobacterium thermoresistibile	SNW18166	ID-DA--------G--A-L-Q-		-P--------------GA---H--
	Mycobacterium tusciae	WP_083125838	IS--P---Y---HN--T-VAG-		V-----------T---GT---H--
	Mycobacterium ulcerans	WP_011740068	L---------------R-R-E-		EI--------------G----L-Q
	Mycobacterium xenopi	WP_085197318	VP--A---Y----N--K-LAG-		VV----------V---GT---H--
	Mycobacterium xenopi 3993	EUA24313	VP--A---Y----N--K-LAG-		VV----------V---GT---H--
	Mycobacterium xenopi 4042	EUA68342	VP--A---Y----N--K-LAG-		VV----------V---GT---H--
	Mycobacteroides abscessus	WP_052624728	LP--P--------N--T-VDG-		IT----------V---GA-Y-H--
	Mycobacteroides chelonae	WP_070918665	LP--P---Y----N--T-VDG-		IT----------V---GA-Y-H--
	Mycobacteroides franklinii	WP_078334536	LP--P---Y-L--N--T-VDG-		VT----------V---GA-Y-H--
	Mycobacteroides immunogenum	WP_064633126	LP--P---Y----N--T-VDG-		IT----------V---GA-Y-H--
	Mycobacteroides salmoniphilum	WP_078326763	LP--P---Y----N--T-A-G-		VT----------V---GA-Y-H--
	Mycobacteroides saopaulense	WP_088414140	LP--P---Y----N--T-VDG-		VT----------V---GA-Y-H--
	Mycolicibacterium agri	WP_097944590	AQSG----Y----G--T-V-S-		----Q---I---M--VGA---H--
	Mycolicibacterium aromaticivorans	WP_036337805	TA--P---H----N--S-LAD-		-P--------------GA-Y-Q--
	Mycolicibacterium celeriflavum	WP_083002234	VA--P---Y----N--T-EAG-		-P---L------V---GT---H--
	Mycolicibacterium conceptionense	OMB77444	IN------Y----N--T-VAG-		V---D--E----F---GT---H-A
	Mycolicibacterium confluentis	WP_109788489	SP--P---Y----S--T-L-D-		-L----------M---AI---H--
	Mycolicibacterium farcinogenes	WP_109531157	IN------Y----N--T-VAG-		V---D--E----F---GT---H-A
	Mycolicibacterium fortuitum	OBA94220	IR-----------N--T-VAG-		V------E----I---GT---H-A
	Mycolicibacterium goodii	WP_083453029	IP-A----Y----N-RT-V-G-		V-------V---M---GT---H--

Figure S50. Partial sequence alignment of the polyketide synthase protein showing a three amino acid insertion that is specific for most members of the “Tuberculosis” clade.

Clade	Species	Accession	170		225
"Tuberculosis" clade (9/9)	*Mycobacterium tuberculosis*	AIH36384	SIDYRLAPEHPAPAAVEDAYAAFVWAHEHAS	D	EFGALPGRVAVGGDSAGGNLSAVV
	Mycobacterium bovis	WP_023349817	-------------------------------	-	------------------------
	Mycobacterium canettii	WP_015294068	-------------------------------	-	------------------------
	Mycobacterium caprae	APU26876	-------------------------------	-	------------------------
	Mycobacterium microti	AMC60705	-------------------------------	-	------------------------
	Mycobacterium mungi	OAQ16364	-------------------------------	-	------------------------
	Mycobacterium orygis	EMT34764	-------------------------------	-	------------------------
	Mycobacterium pinnipedii	PRH92506	-------------------------------	-	------------------------
	Mycobacterium africanum	WP_031670981	-------------------------------	-	------------------------
Other *Mycobacteriaceae* (0/52)	*Mycobacterium alsense*	WP_083139214	--------------GI-------T--C---A		-L--A---------------A---
	Mycobacterium arosiense	WP_083067212	---------------I-------T-----GG		-L--T---------------A---
	Mycobacterium asiaticum	WP_036354539	-V-------------I-------T--S---A		-L--A---------------A---
	Mycobacterium avium	WP_003875039	---------------ID------T------G		-L--I---------------A---
	Mycobacterium avium 09-5983	ETB28124	---------------ID------T------G		-L--I--------------
	Mycobacterium bohemicum	WP_085180740	-V---------------------T--Y---A		-L--A---------------AT--
	Mycobacterium branderi	WP_083129535	----------R-----D------R------G		DL--I---------------A---
	Mycobacterium celatum	WP_085167745	----------------D------R------G		-L--I---------------A---
	Mycobacterium chimaera	WP_087139895	---------------I-------T--YD--G		-L--A---I-----------A---
	Mycobacterium colombiense	WP_064880926	--A------------I-------T--YR--E		-L-GT---------------A---
	Mycobacterium conspicuum	WP_085232163	---------------I-------E--Y--GA		-L--R---------------A---
	Mycobacterium europaeum	WP_085240986	-----------------------R---D--G		-L--A---------------A---
	Mycobacterium fragae	WP_085196545	--G-------------D--H---R--C--VD		DL--I--K------------A---
	Mycobacterium gastri	WP_036410862	---------QH---GID--F---T--Y---G		-L--I---I-----------A---
	Mycobacterium gordonae	WP_065134757	---------------I---FT--Q--C---E		-L--I---------------A---
	Mycobacterium haemophilum	WP_054880358	--------------GI-------E--CQ--A		-L--SS--------------A---
	Mycobacterium heckeshornense	WP_048890785	-V-------------LD------R------A		GL--I--K------------A---
	Mycobacterium heidelbergense	WP_083074582	-----------------------R------G		-L--T---------------A---
	Mycobacterium interjectum	WP_066909362	-------------------H---K------G		-L--T---------------A---
	Mycobacterium intracellulare	WP_095580369	---------------I-------T--Y---G		-L--A---I-----------A---
	Mycobacterium intracellulare s	ARR79295	---------------I-------T--Y---G		-L--A---I-----------A---
	Mycobacterium kansasii	OOK84236	----------H---GID--F---T--Y---G		-L--V---I-----------A---
	Mycobacterium kansasii 732	EUA02599	-V--------H---GID--F---M--YA--D		-L--V---I-----------A---
	Mycobacterium kyorinense	OBI47340	-----------------------Q------G		-L--I---------------A---
	Mycobacterium lacus	WP_085157707	----------------D------K--Y---E		DL--VA-K------------A---
	Mycobacterium lentiflavum	WP_090601124	---------------I-------T---D--G		DL--R---------------A---
	Mycobacterium lepraemurium	ATA29141	---------------ID-----LT------G		-LS-T---------------A---
	Mycobacterium malmoense	WP_065442082	-----------------------R--YD--G		-L--AA--------------A---
	Mycobacterium mantenii	WP_083097387	---------------I-------T--Y---G		-L--T--L------------A---
	Mycobacterium montefiorense	WP_108920644	---------------ID------K--C---G		-L--T---------------A-I-
	Mycobacterium noviomagense	WP_083088124	------------L--ID------R-----GA		-L--M-DKIV----------A---
	Mycobacterium palustre	WP_085077243	---------------ID------K--Y---G		DL--T--Q----------------
	Mycobacterium paraense	WP_085102503	---------N-------------R------A		-L--T---------------A---
	Mycobacterium paraffinicum	WP_073879536	-----------------------R--YD--G		-L--A---------------A---
	Mycobacterium paraseoulense	WP_083174030	-----------------------R--YD--G		-L--A---------------A---
	Mycobacterium parmense	WP_085268077	---------------I---W---T------A		-L--A---I-----------A---
	Mycobacterium persicum	WP_083153413	----------H---GID--F---T--Y---G		-L--V--QI-----------A---
	Mycobacterium riyadhense	WP_085253593	-V--------R------------Q--Y---G		GL--I---------------A---
	Mycobacterium saskatchewanense	WP_085256205	---------------I-------T--Y---G		-L--A---------------A---
	Mycobacterium scrofulaceum	WP_067278368	-V---------------------R--Y---G		-L--A---------------A---
	Mycobacterium sherrisii	WP_069398584	-VG-----------GLD-----YR--C---A		-L--V---------------A---
	Mycobacterium shigaense	BAX91763	---------------I-------T--C---G		----TS--------------A---
	Mycobacterium shimoidei	WP_069394970	----------------D------R--C---A		DL--Q---------------A---
	Mycobacterium shinjukuense	WP_083046807	----------R----ID--V---E---A--A		-L--I--K------------A---
	Mycobacterium triplex	WP_036467966	---------------ID------K--W---G		-L--A---------------A-L-
	Mycobacterium vulneris	WP_085292421	---------------I-------T--YR--G		-L--T---------------A---
	Mycobacterium xenopi	WP_003922089	--------------GLD------R------A		-L--I--N------------A---
	Mycolicibacter algericus	WP_109559550	---------------LD------R--C---A		DL--I---------------A-G-
	Mycolicibacter algericus DSM 4	OQZ95135	---------------LD------R--C---A		DL--I---------------A-G-
	Mycolicibacter senuensis	ORW65371	---------------LD------R--C---H		-L-GI---I-----------A-G-
	Mycolicibacter sinensis	WP_013829602	---------------LD------R--C---A		DL--I---------------A-G-
	Mycolicibacterium brumae	WP_090585634	-V-------------F------YL--V---A		-L--D-A-------------A---

Figure S51. Partial sequence alignment of a lipase protein showing a one amino acid insertion that is specific for members of the "Tuberculosis" clade.

Group	Species	Accession	13		61
"Tuberculosis" clade (9/9)	*Mycobacterium tuberculosis*	CFE39138	TRKSFSIAGTRVVDAIKLSVPFQTL		GKSVADDAGLAANVRDAVGNLAAE
	Mycobacterium bovis BCG	AMC52153	-------------------------		------------------------
	Mycobacterium canettii	WP_015294138	-------------------------		------------------------
	Mycobacterium caprae	APU26950	-------------------------		------------------------
	Mycobacterium microti	AMC60792	-------------------------		------------------------
	Mycobacterium mungi	OAQ16436	-------------------------		------------------------
	Mycobacterium orygis	EMT34649	-------------------------		------------------------
	Mycobacterium pinnipedii	PRH90064	-------------------------		------------------------
	Mycobacterium africanum	CCC28131	-------------------------		------------------------
Other *Mycobacteriaceae* (0/56)	*Mycobacterium alsense*	WP_083137013	-----G---A--LE--------K--	E	-RPP-EH-E-S-T---V--K----
	Mycobacterium angelicum	WP_083112114	-----G---A---G------A-K--	E	--AP-EH-E-S-----V--K-T--
	Mycobacterium arosiense	WP_083064358	-----G---A---E--------K--	A	--AP-EH-E-S--------K----
	Mycobacterium asiaticum	OBK28902	-----G---A--I-E----------	G	-QPP-RS-E-TD----V--K----
	Mycobacterium avium	WP_003874932	-----G---A---ES-------K--	A	--AP-EH-E-S-----V--K----
	Mycobacterium avium 05-4293	ETA94798	-----G---A---ES-------K--	A	--AP-EH-E-S-----V--K----
	Mycobacterium avium 11-0986	ETB50998	-----G---A---ES-------K--	A	--AP-EH-E-S-----V--K----
	Mycobacterium avium subsp. hominissuis	ETB32074	-----G---A---ES-------K--	A	--AP-EH-E-S-----V--K----
	Mycobacterium avium subsp. paratuberculosis	AAS05652	-----G---A---ES-------K--	A	--AP-EH-E-S-----V--K----
	Mycobacterium avium subsp. silvaticum	ETB13699	-----G---A---ES-------K--	A	--AP-EH-E-S-----V--K----
	Mycobacterium branderi	WP_083130440	-----G---P---E-------LKS-	D	--HPGEH-E-R-----V--K----
	Mycobacterium celatum	WP_062540237	-----G---P---E-------LAS-	D	--HPREH-E-T--I--V--K----
	Mycobacterium chimaera	WP_095795726	-----G---A----S-------K--	E	-RPP-EH-E-S-----V--K----
	Mycobacterium colombiense	OBI37709	-----G---A-----------KS-	E	--AP--H-E-S-----V--K----
	Mycobacterium colombiense CECT	EJO87655	-----G---A---E--------K--	E	--AP-EHPE-S-----V--K----
	Mycobacterium conspicuum	WP_085232431	-----G---A---E--------K--	D	--AP-EH-E-SDS---V--K----
	Mycobacterium europaeum	CQD20752	-----G---A----S-------K--	E	--AP-EH-E-SG-------K-V--
	Mycobacterium florentinum	WP_085226078	-----G---A--IEG-------A--	E	--PP-KN-E-V--------K----
	Mycobacterium fragae	WP_085193282	-----A---P---E--------R--	D	--HPREH-E----------K----
	Mycobacterium gastri	ORV66887	-----A---A--I----------S-	A	--TP--SSEMS--L--I--K----
	Mycobacterium gordonae	WP_055577475	-----G---A--S------------	G	-SAP--N-E-SG----V--K----
	Mycobacterium heckeshornense	WP_048890854	-----G---P--IE-------L-S-	Q	--HPR---E-VS----V--K----
	Mycobacterium heidelbergense	WP_083074632	-----G---AA--E-V------K--	E	-RAP-E--E-S-----V--K----
	Mycobacterium intermedium	WP_069421426	-----G---A---ES-------RS-	D	--AP-EHEE-T--------K----
	Mycobacterium intracellulare	WP_014942702	-----G---A----S-------K--	E	-RPP-EH-E-S-----V--K----
	Mycobacterium intracellulare A	AFC44977	-----G---A----S-------K--	E	-RPP-EH-E-S-----V--K----
	Mycobacterium intracellulare s	AGP65349	-----G---A----S-------K--	E	-RAP-EH-E-S-----V--K----
	Mycobacterium kansasii	KZS67459	-----A---A--I----------S-	A	--AP--SSEMS--L--I--K----
	Mycobacterium kansasii 732	EUA00055	-----A---A--I-T-------HS-	A	-QAP--SSEMS--L--I--K----
	Mycobacterium kyorinense	WP_045383517	-----G---P---E-------LKS-	D	--HPSEH-E-T-----V--K----
	Mycobacterium lacus	WP_085160569	-----G---S--I---------L--	D	--AP-ES-E---K-G-V--K----
	Mycobacterium lentiflavum	CQD09804	-----G---A---E--------G--	E	--AP-EH-E-V-S------K----
	Mycobacterium liflandii	WP_015355075	-----G---A--S---------L--	E	-NPP--NSE-T-----IIAK----
	Mycobacterium malmoense	WP_065442505	-----G---A---ES-------K--	E	--AP-EH-E-S-----V-EK-V--
	Mycobacterium mantenii	WP_083092792	-----G---A---ES-------K--	E	--AP-EH-E-SG----V--K----
	Mycobacterium marinum	WP_020724592	-----G---A--S---------L--	E	-NPP--NSE-T-----IIAK----
	Mycobacterium marseillense	WP_083018890	S----G---A----S-------T--	D	-RAP-EH-E-S-----V--K----
	Mycobacterium microti OV254	PLV49948	-----G---A---ES-------GS-	D	--AP-E--E-V------L-K-T--
	Mycobacterium montefiorense	GBG36504	-----G---A---E-V------A--	E	--AP-EH-E-V--------K----
	Mycobacterium nebraskense	WP_046184854	-----G---A---ES-------K--	E	--AP-EH-E-SG-------K-V--
	Mycobacterium noviomagense	WP_083088360	-----G---P---E---------S-	D	--HPREN-E-VS----V--K----
	Mycobacterium paraffinicum	WP_073872329	-----G---A---ES-------K--	E	--AP-EHGE-SG-----L-K----
	Mycobacterium parascrofulaceum	WP_007171235	-----G---A---ES-------K--	E	--AP-EH-E-SE----V--K-V--
	Mycobacterium parmense	WP_085267921	-----G---A---EDL------T--	E	--AP-EH-E-S-----I--K----
	Mycobacterium saskatchewanense	ORW72590	-----G---A---E-L---------	E	--AP-EH-E-TQ----V--K----
	Mycobacterium scrofulaceum	WP_083177832	-----G---A---ES-------K--	E	--AP-EH-E-SE----V-EK-V--
	Mycobacterium sherrisii	WP_069399772	-----G---A---ES-------GS-	E	--AP-EH-E-V------L-K-T--
	Mycobacterium shigaense	WP_096438370	-----G---A---ES-------T--	E	--AP-EH-EVS-S---V--K----
	Mycobacterium shimoidei	WP_069395051	-----A---P---E-------L---	D	--APREH-E-S-----V--K----
	Mycobacterium shinjukuense	WP_083049586	-----G--------G------L---	E	-RPP----E-S-----V--K----
	Mycobacterium simiae	AMP24881	-----G---A-I-ES-------GS-	D	--AP-E--E-V------L-K-T--
	Mycobacterium szulgai	WP_085669650	-----G---A---E--E-----S--	E	--AP-EH-E-S-----V--K----
	Mycobacterium triplex	CDO87707	-----G---A------------A--	E	--AP-EH-E-V--------K----
	Mycobacterium ulcerans	WP_096370280	-----G---A--S---------L--	E	-NPP--NSEVT-----IIAK----
	Mycobacterium vulneris	WP_085290116	-----G---A---E--------K--	G	--AP--H-E-S-----V--K----
	Mycobacterium xenopi	WP_003922188	-----G---P---E-------LRS-	Q	--HPRES-E--S----V--K----

Figure S52. Partial sequence alignment of a secreted protein showing a one amino acid deletion that is specific for members of the "Tuberculosis" clade.

	Species	Accession	208		254
"Tuberculosis" clade (8/9)	*Mycobacterium tuberculosis*	WP_031727138	QLAHTDSGLLMSTFGPRTALWLLL		AKGGGDTEVSAQAWVPRSRSHAV
	Mycobacterium bovis	WP_044798225	------------------------		-----------------------
	Mycobacterium canettii	WP_014001558	------------------------	L	---------R-------------
	Mycobacterium caprae	APU26952	------------------------		-----------------------
	Mycobacterium microti	AMC60794	------------------------		-----------------------
	Mycobacterium mungi	OAQ16438	------------------------		-----------------------
	Mycobacterium orygis	WP_003415985	------------------------		-----------------------
	Mycobacterium pinnipedii	PRH90066	------------------------		-----------------------
	Mycobacterium africanum	WP_031670343	------------------------		-----------------------
Other *Mycobacteriaceae* (1/53)	*Mycobacterium angelicum*	ORA01569	------------------------		---------------------
	Mycobacterium alsense	WP_083137012	E---S-AES-T-E-----G-----	L	------SD---EP--------V-
	Mycobacterium arosiense	WP_083064417	----C-AE--T-------G-----	L	------SH---EP--------V-
	Mycobacterium asiaticum	OBI98292	----S-GE-VT-M-----G-----	L	-----------EP--------VI
	Mycobacterium avium	WP_003874930	----S-AE--TA------G-----	L	------DH----P--------V-
	Mycobacterium avium 104	ABK67995	----S-AE--TA------G-----	L	------DH----P--------V-
	Mycobacterium avium MAV_120709	ETZ56441	----S-AE--TA------G-----	L	------DH----P--------V-
	Mycobacterium avium MAV_120809	ETZ47374	----S-AE--TA------G-----	L	------DH----P--------V-
	Mycobacterium avium subsp. paratuberculosis	ETB07205	----S-AE--TA------G-----	L	------DH----P--------V-
	Mycobacterium avium subsp. silvaticum	ETB13724	----S-AE--TA------G-----	L	------DH----P--------V-
	Mycobacterium celatum	WP_062540236	E---S-AE--T-------G-----	L	-N----DV---EP--------V-
	Mycobacterium colombiense	OBJ32471	----C-AE--TAA-----G-----	L	------SQ---EP--------M-
	Mycobacterium conspicuum	WP_085232266	---RA-AE--T-------G-----	L	-----------AP--------V-
	Mycobacterium europaeum	WP_085241158	E--DS-AE--T-A-----G-----	L	-------G---EP-I------V-
	Mycobacterium florentinum	WP_085227040	E---S-AE--T-------G-----	L	-----------EP--------V-
	Mycobacterium fragae	WP_085193285	------AE--T-------G-----	L	-N----DV---EP--------V-
	Mycobacterium genavense	WP_025735584	E---S-AE--T-------G-----	L	-----------EP--------V-
	Mycobacterium gordonae	WP_055577473	---R--GE--T-------G-----	L	----------S-P----------
	Mycobacterium haemophilum	WP_054881032	----S-GE--T-------G-----	L	-------A----P--------V-
	Mycobacterium heckeshornense	WP_048890856	E--R--AE--T-------G-----	L	-------D---TP--------V-
	Mycobacterium heidelbergense	WP_083074631	E---S-AE--T-A-----G-----	L	------AD---EP-I------V-
	Mycobacterium interjectum	WP_084453959	E---A-AEA-TAA-----G-----	L	-------D---EP--------V-
	Mycobacterium intermedium	WP_069420991	----S-GE--T-A-----G---M-	L	-------N---EP--------V-
	Mycobacterium intracellulare	WP_036389998	----S-AE--TA---A--G-----	L	------SH---EP--------V-
	Mycobacterium intracellulare s	AGP65351	K---S-AE--TA---A--G-----	L	------SH---EP--------V-
	Mycobacterium intracellulare s	ARR79412	K---S-AE--TA---A--G-----	L	------SH---EP--------V-
	Mycobacterium kubicae	WP_085073501	E---A-AE--T-K-----G-----	L	------D----EP--------V-
	Mycobacterium lacus	WP_085160648	---RS-AE--T-------G-----	L	------AG-T--P--------V-
	Mycobacterium lentiflavum	WP_090608615	A--NC-AE--TA------G-----	L	------A-----P--------V-
	Mycobacterium litorale	WP_078022147	E-----AER-TA------G-----	L	------AT---DP-I------V-
	Mycobacterium malmoense	WP_065442523	G---S-AE--T-A-----G-----	L	------AQ---EP-I------V-
	Mycobacterium montefiorense	WP_108920735	E--NS-AEV-T-------G-----	L	------A----KP--------V-
	Mycobacterium nebraskense	WP_046184852	E---S-AE--T-------G-----	L	-----------EP--------V-
	Mycobacterium noviomagense	WP_083088362	---N-HAE--T------IG-----	L	-------D---EP--------V-
	Mycobacterium palustre	WP_085077373	----S-AE--TA------G-----	L	-------Q---EP--------V-
	Mycobacterium paraense	WP_085093877	E---S-AE--T-------G-----	L	-----------EP--------V-
	Mycobacterium paraffinicum	WP_073871988	E---S-AE--T-A-----G-----	L	------AG---EP--------V-
	Mycobacterium paraseoulense	WP_083175160	E---S-AE--T-A-----G-----	L	-------G---EP-I------V-
	Mycobacterium parmense	WP_085267919	E--DS--E--T-------G-----	L	------AR----P-I------V-
	Mycobacterium rhodesiae	WP_005141565	------AE--TA------G-----	L	------AT---EP-I------VM
	Mycobacterium riyadhense	WP_085251974	----G-AE--A-------G-----	L	----------TEP--------V-
	Mycobacterium saskatchewanense	WP_085255005	E--YA-AE--TA------G-----	L	-----------EP--------V-
	Mycobacterium scrofulaceum	WP_067274710	E--NS-AE--T-A-----G-----	L	------AR---EP-I------V-
	Mycobacterium setense	WP_039318196	D--S--ATV-T-----S-G--I--	L	----------SEP--------VI
	Mycobacterium sherrisii	WP_069399724	E--RS-AE--T-------G-----	L	------AQ---EP--------V-
	Mycobacterium shimoidei	WP_069395053	------AE--T-----K-G-----	L	-N----D-I-SEP--------VI
	Mycobacterium shinjukuense	WP_083049594	----S--E--TP------TV----	L	-N----A----EP--------V-
	Mycobacterium simiae	WP_084952928	E--DS-AE--T-------G-----	L	------A----EP--------V-
	Mycobacterium sphagni	WP_094476635	E---A-AE--TA------G-----	L	------AT---EP-I------V-
	Mycobacterium szulgai	WP_068025633	E---A-AE--T-------G-----	L	------D----EP--------V-
	Mycobacterium thermoresistibile	WP_040546521	D-EA--ATV-TT----S-G-----	L	-----------EP--------V-
	Mycobacterium triplex	CDO87705	E---S-AE--T-------G-----	L	------S----EP--------V-
	Mycolicibacterium aromaticivorans	WP_036345786	E--SA-AE--TA------G-----	L	-------T---EP-I------VM

Figure S53. Partial sequence alignment of the DNA polymerase IV protein showing a one amino acid deletion that is specific for most members of the "Tuberculosis" clade and is absent from most other *Mycobacteriaceae*.

Group	Species	Accession	378		426
"Tuberculosis" clade (11/11)	*Mycobacterium tuberculosis*	AIH57612	RAHLIDGVPWSQMAVIVRSVPRAV		RLPRALAAAGVPVAPPAVGGPLSAE
	Mycobacterium bovis	KFW50291	------------------------		-------------------------
	Mycobacterium bovis AF2122/97	YP_009360587	------------------------		-------------------------
	Mycobacterium bovis BCG	AMC52329	------------------------		-------------------------
	Mycobacterium canettii	WP_015288475	------------------------		-------------------------
	Mycobacterium caprae	APU27073	------------------------		-------------------------
	Mycobacterium microti	WP_105799880	------------------------		-------------------------
	Mycobacterium mungi	OAQ16619	------------------------		-------------------------
	Mycobacterium orygis	WP_003416835	------------------------		-------------------------
	Mycobacterium pinnipedii	PRH90933	------------------------		-------------------------
	Mycobacterium africanum	WP_031701650	------------------------		-------------------------
Other *Mycobacteriaceae* (2/>100)	*Mycobacterium litorale*	AQT81202	----V-------L-------SA-A		A------V------T-HL--AI-DQ
	Mycobacterium sphagni	WP_094483781	----V-------L------PSAVA		A------G------ANH-A--VAEQ
	Mycobacterium angelicum	WP_083111080	-----------------------A	A	--------------I--S--S--DD
	Mycobacterium aquaticum	ORA31589	----V------------------G	T	A-A---TS-----ESVGDVP-VAQQ
	Mycobacterium asiaticum	WP_065035318	----V------------------G	A	------S-------A--M--C-AE-
	Mycobacterium avium	WP_062889609	----V------------------G	A	-------------TA--AS---AEQ
	Mycobacterium avium 05-4293	ETA95159	----V------------------G	A	-------------TA--AS---AEQ
	Mycobacterium avium subsp. paratuberculosis	AAS05851	----V------------------G	A	-------------TA--AS---AEQ
	Mycobacterium branderi	WP_083129869	----V--L---------------A	A	G-AH--TR------M-TS--L-WQQ
	Mycobacterium celatum	WP_085167680	----V--LR--------------A	A	G-AH--TR------M-TS--L-WQQ
	Mycobacterium colombiense	WP_075237364	----V------------------G	A	--------V-----A---P----EQ
	Mycobacterium conspicuum	WP_085231390	--------------------A--G	S	-----------------F-A---E-
	Mycobacterium dioxanotrophicus	ART71436	----V------------------G	T	A-A---TS-----ESVGEVP-VAQQ
	Mycobacterium florentinum	WP_085223088	----V-N----------------G	A	--------------AS--S-S-TEG
	Mycobacterium fragae	WP_085195852	----V------------------T	N	G-SH---R-------T--A-S--DQ
	Mycobacterium gastri	WP_085104986	---V-------------------A	A	----T---------M--I--S----
	Mycobacterium gastri 'Wayne'	ETW23146	---V-------------------A	A	----T---------M--I--S----
	Mycobacterium gilvum	WP_011895502	----V-----DR----------TG	A	S-G----------ED-HPDV--ANQ
	Mycobacterium gordonae	WP_065133007	----VE-----------------G	A	--------------I--I--S--E-
	Mycobacterium grossiae	WP_070355596	----V-----ER---L---M-S-G	A	A-G-T--------ERQPL-A--AEY
	Mycobacterium haemophilum	WP_047313270	-------------V---------G	A	----G---------V---R------
	Mycobacterium holsaticum	WP_069403967	----V--L-------V------HG	A	A-A---EG-----EI--A-I--ADQ
	Mycobacterium insubricum	WP_083030742	-------T--------------VG	T	A-S-T-T--------GRTRP--AEN
	Mycobacterium intermedium	WP_069419047	----V--I---------------A	S	--------------I--I--S-AE-
	Mycobacterium iranicum	WP_064283186	----V-----D-----------SG	T	V-A---S------DH-HSDA--ADQ
	Mycobacterium kansasii	WP_023371746	---V-------------------A	A	--------------M-TM--S--Q-
	Mycobacterium kansasii 824	EUA03109	---V-------------------A	A	--------------M-TM--S--Q-
	Mycobacterium komaniense	WP_090281576	----V------R----------VG	A	A-A-T--------DT-TPAT--GDQ
	Mycobacterium kyorinense	WP_065016599	-----------------------G	A	G-SQ--MR------M-SA--S-WQQ
	Mycobacterium lacus	WP_085161119	-----------R-----------A	T	--------------V-PTS----D-
	Mycobacterium lehmannii	WP_094286016	----V----------V---M--LG	A	A-A-T--------DA-SPAT--GDQ
	Mycobacterium lepraemurium	ATA29329	----V------------------G	A	-------------TA--AS---AEQ
	Mycobacterium mageritense	WP_051578840	----V--IA--D----------VG	T	T-A---Q------QAAGS-LR-ADQ
	Mycobacterium mageritense DSM	CDO19903	----V--IA--D----------VG	T	T-A---Q------QAAGS-LR-ADQ
	Mycobacterium malmesburyense	WP_090338985	----V-----------------LG	A	S-A-T--------DT-TPAT--GDQ
	Mycobacterium montefiorense	GBG36760	----V-K----------------G	A	------S-------AA----S-VE-
	Mycobacterium moriokaense	WP_083154172	----V------E----------LG	S	A-A-T--------DL-TPSA--ADQ
	Mycobacterium mucogenicum	WP_064860817	----V----------------S-G	T	A-A---T------QQQ-GTA-PAQH
	Mycobacterium neoaurum	CDQ46097	----V-----------------VG	S	GIG---V------DL--AESA-AQQ
	Mycobacterium neumannii	WP_094294317	----V----------V---M--LG	A	A-A-T--------DTSSPAT--GDQ
	Mycobacterium neworleansense	WP_090513887	----V------E---V---I--VG	A	A-A---SG-----QATGPDLG-AQQ
	Mycobacterium noviomagense	WP_083086271	--------------------S-VA	A	T--H---R------M-SI--S-PEQ
	Mycobacterium novocastrense	WP_067395793	----V-----------------LG	A	A-A-T--------DT-SPAT--GDQ
	Mycobacterium obuense	WP_082133485	-------I--AD----------MG	P	A-A---T------DGLQSDA--I-V
	Mycobacterium paraense	WP_085174271	----V------------------G	A	--------------A-PAS------
	Mycobacterium paraffinicum	WP_073876041	----V-----------------VG	A	--------------A--AR-S--D-
	Mycobacterium parafortuitum	WP_083143065	----V-----A-----------MG	D	S-G---T------DE-HTDV--TNQ
	Mycobacterium parmense	WP_085267695	----V------------------G	A	------S-------C-R-S-S-WE-
	Mycobacterium peregrinum	WP_064888266	----V------E---V------VG	A	A-A---SG-----HATGADLG-AQQ
	Mycobacterium persicum	WP_089025042	---A-------------------A	A	--------------M--I--S----
	Mycobacterium phlei	WP_061481543	----V--LA-------------LG	A	G-A-T--------DT-TPTT--AQQ
	Mycobacterium porcinum	WP_075921504	----V------E---V---I--VG	A	A-A---SG-----QATGPDLG-AQQ
	Mycobacterium rhodesiae	WP_014208804	----V-----------------TG	S	A-A-T-T------EL-MPSA-PADH
	Mycobacterium rhodesiae JS60	EHB58687	----V-------L------PSA-A	A	------G-----GAAQ-A-SIADQ
	Mycobacterium riyadhense	WP_085252027	----V-D----------------G	A	--------------L--I--S-PE-
	Mycobacterium rufum	KGI67392	----V-----------------TG	A	P-S---S------EV-QSDVA-A-I
	Mycobacterium rutilum	WP_083409859	----V--LA-------------LG	A	AVA-T--------ET-TPTA--AQQ
	Mycobacterium septicum	WP_044516790	----V------E---V---I--VG	A	G-A---SG-----QSTGGDLG-AQQ
	Mycobacterium setense	WP_064871859	----V------E---V------VG	A	A-A---SG-----QATGPDLG-AQQ
	Mycobacterium shigaense	WP_096438015	-----------------------G	V	----V---------A--AS---VE-
	Mycobacterium shimoidei	WP_069396555	-----------E----------VA	A	G-TH--TR------V-SG--L-CEQ
	Mycobacterium shinjukuense	WP_083049868	---------------L-------A	A	--------------I-PA--S-AE-

Other *Mycobacteriaceae* (2/>100)

Mycobacterium smegmatis	WP_003893321	----V--I--------------VG	T	A-A---T------QDNGTDV-VGRQ
Mycobacterium szulgai	WP_068029251	----VE-----------------A	A	--------------R--SS---AD-
Mycobacterium thermoresistibile	GAT15382	----------------------TG	A	P-A---T------DLT-A-LA-ADQ
Mycobacterium timonense	WP_083187477	----V------------------G	A	-------------TA--AS---AEQ
Mycobacterium tusciae	WP_083125060	----V--I--------------LG	S	T-A---TT-----EL-NPTA--ADQ
Mycobacterium vaccae	WP_003929197	----V-----------------TG	A	A-G---T------DL-HNDSA-ADQ
Mycobacterium vulneris	WP_065461062	----V------E---V---I--VG	A	G-A---SG-----QATGPDLG-AQQ
Mycobacterium wolinskyi	WP_067854431	----V--L---------------G	A	S-A---T------QER-T-V--AHQ
Mycobacterium xenopi 4042	EUA06820	-----------H---L---LR--P	A	M-AH---R-----TV--G--S-AEQ
Mycobacteroides abscessus	WP_062879200	-S----D----R----------SG	A	A-R---QS-----HAESYD--VASV
Mycobacteroides abscessus 3A-0	EIV49761	-S---------R----------SG	A	A-C---QS-----HAESYD--VASV
*Mycobacteroides abscessus MAB_*	ESV56355	-S---------R----------SG	A	A-R---QS-----HAESYD--VASV
Mycobacteroides abscessus subsp. abscessus	SIL26859	-S---------R----------SG	A	A-C---QS-----HAESYD--VASV
Mycobacteroides chelonae	WP_070919392	-S-------------V------SG	A	A-R---QS-----HAESYD--VA-V
Mycobacteroides franklinii	WP_078336403	-S--------------------SG	A	A-R---QS-----HAESHD--VA-V
Mycobacteroides immunogenum	WP_064627996	-S--------------------SG	A	A-R---QS-----RAESHD--VA-V
Mycobacteroides salmoniphilum	WP_078323787	-S--------------------SG	A	A-R---QS-----HAESYD--VA-V
Mycobacteroides saopaulense	WP_083013600	-S----D-------------R-SG	A	A-R---QS-----HAESYD--VASV
Mycolicibacillus koreensis	WP_085302500	----V-KLA-------------VA	G	A-A---TT------L--TT-A-AEH
Mycolicibacillus trivialis	WP_069393355	----V-KLA-------------VA	G	A-A---TT------L--TT-A-AEH
Mycolicibacter algericus	WP_083035848	----V------------------A	A	----T-T-------L-IA--L-AQQ
Mycolicibacter arupensis	WP_046191174	-----------------------A	A	----T-----I---L--G--L-AEQ
Mycolicibacter engbaekii	WP_085129321	-----------------------A	M	----T--------TV--S-AL-AQQ
Mycolicibacter heraklionensis	WP_064887988	-----------------------A	A	----T-----I--ML--S--L-AEQ
Mycolicibacter hiberniae	WP_085133950	-----------------------A	M	----T-----I--TV--S-AL-AQQ
Mycolicibacter icosiumassiliensis	WP_067970803	-----------------------A	A	--H-T-----I--TL--S--L-AEQ
Mycolicibacter kumamotonensis	WP_065287540	-----S-----------------A	A	----S-----I--TL--P--L-AEQ
Mycolicibacter longobardus	WP_085264333	--------------------L--A	A	----T-----I--TL--A--L-TEQ
Mycolicibacter minnesotensis	WP_083027542	--R---S----------------A	A	----S-----I---L--G--L-AQQ
Mycolicibacter nonchromogenicus	WP_085137937	-----------------------A	A	----T--------TL--G--L-AEQ
Mycolicibacter senuensis	WP_085081738	----V-EL--------------VA	A	----T-----I---L--A--L-AQQ
Mycolicibacter sinensis	WP_064855461	----L------------------A	A	----T-----I--TL--P--L-AEQ
Mycolicibacter terrae	WP_085262250	----V------------------A	A	----T-----I--TL--P--L-AEQ
Mycolicibacterium agri	WP_097944507	------E-------------RHSG	A	A-S---V------LA-PS-V--AEQ
Mycolicibacterium aurum	WP_087020394	----V--L--------------VG	A	A-G-V--------DL-PADPG-AQQ
Mycolicibacterium austroafricanum	WP_105387748	----V-----------------MG	A	A-G---T-----LDL-QPEV--AEQ
Mycolicibacterium bacteremicum	WP_083057305	----V-----------------VG	A	GIG---V------DL--AETG-AQQ
Mycolicibacterium brisbanense	WP_109784466	----V------------------G	T	A-A---TS-----ESVGDVP-VAQQ
Mycolicibacterium canariasense	WP_062654522	----V----------V-------G	A	A-G-T--------DL--AEPV-ARR
Mycolicibacterium chlorophenolicum	WP_048471357	----V-----------------MG	A	P-S---S------EV-QSDVA-ADM
Mycolicibacterium chubuense	WP_014814699	----V-----A----VL-----MG	A	A-G---T------DL-PADAA-ADQ
Mycolicibacterium confluentis	WP_085148670	-T--------------A---GGVG	A	M---V--N------GG-THP--AYN
Mycolicibacterium diernhoferi	WP_073857797	----V-----------------VG	A	A-G---T------DL-PADPG-AQQ
Mycolicibacterium duvalii	WP_098004075	----V-E--------------QMG	A	A-G---T------DVAHNEAA-ADQ
Mycolicibacterium elephantis	WP_083042567	----V--L--------------LG	A	A-A---EG-----EMAGT-VT-ADQ
Mycolicibacterium flavescens	WP_069415958	----V--L--------------LG	A	AVA-T--------ES-TPTA--AQQ
Mycolicibacterium fortuitum	WP_061262700	----V------E---V------VG	A	A-A---SG-----HATGPDLG-AEQ
Mycolicibacterium fortuitum subsp. fortuitum	EJZ04493	----V------E---V------VG	A	A-A---SG-----HATGPDLG-AEQ
Mycolicibacterium goodii	WP_049747779	----V-----------------VG	I	A-A---TV-----QENGSDV-VAQH

Figure S54. Partial sequence alignment of an ATP-dependent DNA helicase protein showing a one amino acid deletion that is specific for members of the “Tuberculosis” clade and is absent from most other *Mycobacteriaceae*.

Group	Species	Accession	207		256
“Tuberculosis” clade (10/10)	*Mycobacterium tuberculosis*	AIH73423	SPTTVRGGCGYEFRLETSCYNPSFGG	M	DRQSRVFINEARWVRGAVPFEGD
	Mycobacterium bovis	WP_080655140	--------------------------	-	-----------------------
	Mycobacterium bovis BCG str. K	AGE69244	--------------------------	-	-----------------------
	Mycobacterium canettii	WP_015294267	--------------------------	-	-----------------------
	Mycobacterium caprae	PRH97799	--------------------------	-	-----------------------
	Mycobacterium microti	PRI02585	--------------------------	-	-----------------------
	Mycobacterium mungi	WP_003901570	--------------------------	-	-----------------------
	Mycobacterium orygis	WP_003416858	--------------------------	-	-----------------------
	Mycobacterium pinnipedii	PRH91052	--------------------------	-	-----------------------
	Mycobacterium africanum	AMQ39892	--------------------------	-	-----------------------
Other *Mycobacteriaceae* (0/55)	*Mycobacterium alsense*	WP_083140590	--V-------------------VY-A		E--A-------------------
	Mycobacterium angelicum	WP_083111086	--M---V------P-----------P		-----------------------
	Mycobacterium arosiense	WP_083065571	--V-------------------V--A		N-EA-------------------
	Mycobacterium asiaticum	WP_036353388	--VS--E---------------A--A		---A---------I---------
	Mycobacterium avium	WP_009978669	--V---E---------------V--A		N-EA-------------------
	Mycobacterium avium 10-5581	ETB00793	--V---E---------------V--A		N-EA-------------------
	Mycobacterium avium subsp. paratuberculosis	EGO38462	--V---E---------------V--A		N-EA-------------------
	Mycobacterium bohemicum	ORU97556	--V---E---------------A--R		---A-------------L-----
	Mycobacterium chimaera	WP_095662930	--V---E---------------V--A		N-EA-------------------
	Mycobacterium colombiense	WP_064951701	--V---E---------------V--A		N-EA---------I---------
	Mycobacterium colombiense CECT	EJO88266	--V---E---------------V--A		N-EA---------I---------
	Mycobacterium conspicuum	ORV46132	--V---E---------------VY-P		---A-------------------
	Mycobacterium europaeum	WP_085241617	--V---E------------------K		---P-------------------
	Mycobacterium gastri	WP_051508106	--L---E------------------A		---A-------------------
	Mycobacterium gordonae	WP_055577351	--VS--E------------------S		-------------I---------
	Mycobacterium haemophilum	WP_047313276	--L---E------------------P		---A---------L---L-----
	Mycobacterium heidelbergense	WP_083073138	--V---E---------------VY-A		---A-------------------
	Mycobacterium interjectum	WP_085201869	--V-------------------VY-P		N-EA-------------------
	Mycobacterium intermedium	WP_069419037	--VS--E------------------A		-------V---------------
	Mycobacterium intracellulare	WP_009955650	--V---E---------------V--A		N-EA-------------------
	Mycobacterium intracellulare M	ETZ31621	--V---E---------------V--A		N-EA-------------------
	Mycobacterium kansasii	ORB85931	--L---E------------------A		---A-------------------
	Mycobacterium kansasii 662	AIR20383	--L----------------------A		---A-------------------
	Mycobacterium kansasii 732	EUA08382	--L---E------------------A		---A-------------------
	Mycobacterium kansasii 824	EUA04801	--L----------------------A		---A-------------------
	Mycobacterium lacus	WP_085161131	--M---E------------------P		---A---V---------------
	Mycobacterium leprae	WP_010907946	--L-I-A------------------P		---A--L------L---L-----
	Mycobacterium lepromatosis	WP_045842708	--L-I-V------------------P		---A--L------L---L-----
	Mycobacterium liflandii	WP_051045779	--V---E------P---------Y-P		---A-------------------
	Mycobacterium liflandii 128FXT	AGC61472	--V---E------P---------Y-P		---A-------------------
	Mycobacterium malmoense	WP_050789988	--V-------------------AY-S		-----------------------
	Mycobacterium marinum	WP_094358529	--V---E------P---------Y-P		---A-------------------
	Mycobacterium marinum MB2	EPQ73299	--V---E------P---------Y-P		---A-------------------
	Mycobacterium microti OV254	PLV48167	--V---E---------------VY-P		---A-------------------
	Mycobacterium nebraskense	WP_046185300	--V---E---------------A--R		-----------------------
	Mycobacterium palustre	WP_085077983	--V---D---------------VY-A		---A-------------------
	Mycobacterium paraense	WP_085103854	--V-------------------VY-P		N-EA-------------------
	Mycobacterium paraffinicum	WP_073876051	--V---E------------------K		-----------------------
	Mycobacterium parascrofulaceum	EFG75972	--V-------------------AY-S		-----------------------
	Mycobacterium paraseoulense	WP_083170104	--V---E------------------R		-----------------------
	Mycobacterium parmense	WP_085267690	--V---E----------------Y-A		---A--M----------------
	Mycobacterium persicum	WP_083153873	--L---E------------------A		---A-------------------
	Mycobacterium pseudoshottsii J	GAQ37189	--A---E------P---------Y-P		---A-------------------
	Mycobacterium riyadhense	WP_085252023	--M---E------------------A		-------L---------------
	Mycobacterium saskatchewanense	ORW74006	--V---E------------------A		-------------I---------
	Mycobacterium scrofulaceum	WP_083176932	--V---E------------------K		-----------------------
	Mycobacterium sherrisii	WP_069402505	--V---E---------------VY-P		---A-------------------
	Mycobacterium shigaense	BAX91426	--V---E---------------V--P		---A----------------Q--
	Mycobacterium shinjukuense	WP_083049890	--M---E------------------A		-----------------------
	Mycobacterium simiae	AMP24692	--V---E---------------VY-P		---A-------------------
	Mycobacterium szulgai	WP_085670779	--M---V------P-----------P		-----------------------
	Mycobacterium triplex	ORX02984	--V---E---------------AY-P		E--A-------------------
	Mycobacterium ulcerans	WP_071497838	--V---E------P---------Y-P		---A-------------------
	Mycobacterium ulcerans Agy99	ABL04871	--V---E------P---------Y-P		---A-------------------
	Mycobacterium ulcerans str. Harvey	EUA89555	--V---E------P---------Y-P		---A-------------------

Figure S55. Partial sequence alignment of a membrane protein showing a one amino acid insertion that is specific for members of the “Tuberculosis” clade.

			215		269
“Tuberculosis” clade (9/9)	*Mycobacterium tuberculosis*	AIH51381	IDATRPLISDPFEAHEVDEHVQDLLAG	D	GKGMRMEVDAGGATVLLRTLPLVVAGR
	Mycobacterium bovis	WP_024458059	---------------------------	-	---------------------------
	Mycobacterium canettii	WP_015303772	---------------------------	-	---------------------------
	Mycobacterium caprae	WP_105812584	---------------------------	-	---------------------------
	Mycobacterium microti	WP_105799540	---------------------------	-	---------------------------
	Mycobacterium mungi	WP_064319905	---------------------------	-	---------------------------
	Mycobacterium orygis	EMT34441	---------------------------	-	---------------------------
	Mycobacterium pinnipedii	PRH90948	---------------------------	-	---------------------------
	Mycobacterium africanum	CCC28299	---------------------------	-	---------------------------
Other *Mycobacteriaceae* (0/60)	*Mycobacterium alsense*	WP_083138615	--V-----------Q--A---R--V--		-ES---------------------H-I
	Mycobacterium angelicum	WP_083111098	-RV-----------Q--A---L-----		-AS--------D------------HKA
	Mycobacterium arosiense	WP_083067362	-EV-----------A--A---S--V--		-RS-------------------M-D-A
	Mycobacterium asiaticum	WP_036353355	-KV-----------Q--A---LN----		-SS---------------------H-Q
	Mycobacterium avium	WP_009978684	-EI-------S---Q-MA---K-----		--S---------------------N-A
	Mycobacterium bohemicum	WP_085182024	-KV-----------Q-LA---L-----		--SV--------------------H-A
	Mycobacterium colombiense	WP_007773060	--V-----------Q--A---R--V--		-RS-------------------M-D-V
	Mycobacterium conspicuum	WP_085231409	VQV-------S---Q-MA---------		--S---------------------H--
	Mycobacterium europaeum	WP_085241765	VKV-----------Q-LA---L-----		--S---------------------D-A
	Mycobacterium florentinum	WP_085220075	-RI-------S---Q-MA---------		--S---------------------H-A
	Mycobacterium gastri	WP_085104984	VEV-----------E--A---RN--T-		-PS-------------------M-H-A
	Mycobacterium gastri 'Wayne'	ETW23051	VEV-----------E--A---RN--T-		-PS-------------------M-H-A
	Mycobacterium genavense	WP_025735746	-RI-----------Q-MA---------		--S---------------------H-A
	Mycobacterium gordonae	WP_055577337	-KV-----------Q--A---LN----		-SS---------------------H-E
	Mycobacterium haemophilum	WP_047313289	VKV----L------Q--AK--------		-PS--V--E---------------H-S
	Mycobacterium heckeshornense	WP_048891229	-EI-------------LA---R-S---		-AS--I------------------H-S
	Mycobacterium heidelbergense	WP_083073151	VEV-------S---Q--A---R-----		--S----F----------------Q-S
	Mycobacterium interjectum	WP_066908329	V-V-----------Q--A---R-----		-RS---------------------H-A
	Mycobacterium intermedium	WP_069419026	-RV-----------Q-LA---L-----		-ASV-------------------RK-E
	Mycobacterium intracellulare	WP_009952806	-EV-----------Q--A---R--V--		-RS---------------------D-A
	Mycobacterium kansasii	OOK84423	VEV-----------E--A---R---T-		-PS-------------------M-H-A
	Mycobacterium lacus	WP_085161149	-KV-----------Q--A---L-----		-SS---------------------H--
	Mycobacterium lentiflavum	WP_090600784	-RI-------S---Q-MA---------		--S---------------------H-A
	Mycobacterium leprae	WP_010907937	VKV----L------Q--AK------D-		-PS--V--N---------------H-S
	Mycobacterium leprae Kyoto-2	BBC16776	VKV----L------Q--AK------D-		-PS--V--N---------------H-S
	Mycobacterium lepraemurium	ATA29349	-EI-------S---Q--A---K-----		--S---------------------N-A
	Mycobacterium lepromatosis	WP_045842700	VKV----L------Q--AK------D-		-PS--V------------------H-S
	Mycobacterium liflandii	WP_015354884	-EV-----------E--A---R---V-		-RS---------------------G-V
	Mycobacterium malmoense	WP_065444988	VKV-----------Q-LA---L-----		-RS-------------------M-D-A
	Mycobacterium mantenii	WP_083096176	-EV-----------Q--A------V--		-RS-------------------M-D-A
	Mycobacterium marinum	WP_020732172	-EV-----------E--A---R---V-		-RS---------------------G-V
	Mycobacterium marseillense	WP_083020100	-EV-----------Q--A---R--V--		-RS---------------------D-A
	Mycobacterium montefiorense	WP_108920951	-RI-------S---Q-MA----A----		--S---------------------H-A
	Mycobacterium nebraskense	WP_046185527	VKV-----------Q-LA---L-----		--S---------------------D-A
	Mycobacterium noviomagense	WP_083086319	-EV-----------Q-LAD--R-S---		-AS--I------------------H-S
	Mycobacterium palustre	WP_085080132	VEV-----------Q--A---R-----		-RS---------------------R-V
	Mycobacterium paraense	WP_085095946	V-V-----------Q--A---R-----		-RS---------------------H-A
	Mycobacterium paraffinicum	WP_073876068	VKV----V------Q-LA---L-----		-RS---------------------D-A
	Mycobacterium parascrofulaceum	EFG76001	VKV-----------Q-LA---L-----		-RS-------------------M-D-A
	Mycobacterium paraseoulense	WP_083169987	VKV-----------Q-LA---L-----		--S---------------------D-A
	Mycobacterium parmense	WP_085267679	V-------------Q--A---E--V--		--S---------------------K-A
	Mycobacterium pseudoshottsii J	GAQ37174	-EV-----------E--A---R---V-		-RS---------------------G-V
	Mycobacterium riyadhense	WP_085252014	-RV-----------Q--A---L-----		-AS--------D------------H--
	Mycobacterium saskatchewanense	WP_085254203	-KV-----------Q-LA---L-----		--S---------------------K-V
	Mycobacterium scrofulaceum	WP_067268423	VKV----V------Q-LA---L-----		-RS---------------------D-A
	Mycobacterium sherrisii	WP_069402497	-RI-------S---Q-MA---------		--S---------------------H-A
	Mycobacterium shigaense	WP_096437983	-RI-------S---Q--A--I------		--S-------------------M-H--
	Mycobacterium shinjukuense	WP_083047337	-KV-----------Q--A---L-----		-AS---------------------H--
	Mycobacterium simiae	WP_061558570	-RI-------S---Q-MA---------		--S---------------------H-A
	Mycobacterium szulgai	WP_085670768	-RV-----------Q--A---L-----		-AS--------D------------HKS
	Mycobacterium triplex	WP_036467349	-RI-------S---Q-MA---------		--S---------------------H-A
	Mycobacterium ulcerans	WP_011740500	-EV-----------E--A---R---V-		-RS---------------------G-V
	Mycobacterium ulcerans str. Harvey	EUA89620	-EV-----------E--A---R---V-		-RS---------------------G-V
	Mycobacterium vulneris	WP_085292530	-EV-----------Q--A---R--V--		-RS-------------------M-D-V
	Mycobacterium xenopi	WP_003920843	-EV-------------LA---R-S---		-AS--I------------------H-S
	Mycobacterium xenopi 3993	EUA19514	-EV-------------LA---R-S---		-AS--I------------------H-S
	Mycobacterium xenopi 4042	EUA06903	-EV-------------LA---R-S---		-AS--I------------------H-S
	Mycolicibacterium agri	WP_097939997	VAV-----------Q-LA---R-S-V-		-SS----------A----------H-E
	Mycolicibacterium chlorophenolicum	WP_048471381	VTV-----------Q-LAN--R-S---		-SS----------A--------A-H-Q
	Mycolicibacterium chubuense	WP_048416667	VTV-----------Q-LAN--R-S---		-SS----------A--------A-H-Q

Figure S56. Partial sequence alignment of an ATPase protein showing a one amino acid insertion that is specific for members of the “Tuberculosis” clade.

Group	Species	Accession	80		132
"Tuberculosis" clade (9/9)	*Mycobacterium tuberculosis*	AIH44654	RVDHRARIILEANQ	QEQA	IRVVGVDLGLLEVIDRHNDGAVVAHLGPDLLADDW
	Mycobacterium bovis	WP_099183256	--------------	----	-----------------------------------
	Mycobacterium canettii	WP_014001671	--------------	----	-----------------------------------
	Mycobacterium caprae	APU27160	--------------	----	-----------------------------------
	Mycobacterium microti	AMC61065	--------------	----	-----------------------------------
	Mycobacterium mungi	OAQ16705	--------------	----	-----------------------------------
	Mycobacterium orygis	WP_003417205	--------------	----	-----------------------------------
	Mycobacterium pinnipedii	PRH91013	--------------	----	-----------------------------------
	Mycobacterium africanum	WP_031669431	--------------	----	-----------------------------------
Other *Mycobacteriaceae* (2/66)	*Mycobacterium talmoniae*	WP_071029309	L---V--L---G-	GRD-	V--L-I---V--LL---R-QDA----------E--
	Mycolicibacterium doricum	WP_085190243	-AGY-I------GE	G---	VQAA-I---V--ILE-EH-MDA---------G---
	Mycobacterium angelicum	WP_083111175	------------AN		--A--I---V--ILE-DH-SDA---------GE--
	Mycobacterium arosiense	WP_083067224	----------KTDT		AQA------V---LE-DR--DA---------GE--
	Mycobacterium asiaticum	WP_036353133	-------M----GD		--A------V--IL--ED-V-A--Y------GP--
	Mycobacterium avium	WP_009978797	--------V--TGS		ATA------V-RIL--DR--EA---------GE--
	Mycobacterium bohemicum	WP_085182099	------------G-		--A------V--IL--DR--DA---------GA--
	Mycobacterium branderi	WP_083129980	------------GG		--A--I---V--IL--EH-QD----------G---
	Mycobacterium celatum	WP_062541444	-----V------SG		V--L-I---V--IL--EH-QD----------G---
	Mycobacterium colombiense	WP_064877555	-----------TDS		V-A------V---LE-ER--DA---------GE--
	Mycobacterium conspicuum	WP_085231513	K------------E		V-A------V---LE-AR--D----------GE--
	Mycobacterium europaeum	WP_085241776	--------V---GS		V-A------V--IL--DR--DA---------GP--
	Mycobacterium florentinum	WP_085219980	-A-----------D		--A--I---V--IL--DH--EA---------GP--
	Mycobacterium fragae	WP_085198670	---T------GD		V-----------IL--AD-LS----------G---
	Mycobacterium genavense	WP_025737732	-I-----------D		--A--I---V--IR--DH--EA---------GP--
	Mycobacterium gordonae	WP_055579341	------------GD		--A--I---V--ILE-EH--V----------GP--
	Mycobacterium heckeshornense	WP_048891862	---V------GE		--AL-I---V--ILN-ND-TS---R------G---
	Mycobacterium heidelbergense	WP_083073234	-------------G		--A------V--IVA-DR--DA--------MGE--
	Mycobacterium houstonense	WP_066903670	--I--V--TAD		S-AT-I---V---L--E--V-A---------GP--
	Mycobacterium interjectum	WP_085200868	--------V---SD		--A------V--ILE-DR--D----------GP--
	Mycobacterium intracellulare	WP_014383209	--------V---GT		V-A------V--IL--DR--EA---------GE--
	Mycobacterium kansasii	ORB85828	------------RD		--A-----AV--LL--D--------------GAE-
	Mycobacterium kyorinense	WP_045381281	-----V------GD		--A--I---V--IL--EH-QD----------GA--
	Mycobacterium lacus	WP_085159389	-------------A		--A--AE--V--IL--DH--D----------GH--
	Mycobacterium lentiflavum	WP_090600690	-T-----------D		--A--I---V--IL--DR--EA---------GP--
	Mycobacterium lepraemurium	ATA29424	--------V--TGV		ATA------V-QIL--DR--DA---------GE--
	Mycobacterium malmoense	WP_065482441	--------V---GG		V-A------V--ILE-DR-AEA---------GE--
	Mycobacterium marinum	WP_012393115	----H--------D		--A------V--LLE-D-E------------GE--
	Mycobacterium marseillense	WP_095577726	--------V--TGT		V-A------V--IL--DR--EA---------GE--
	Mycobacterium montefiorense	WP_108921262	--------V----D		--A------V--IL--DH-QE----------GP--
	Mycobacterium nebraskense	KKB97130	--------V---GA		V-A------V--ILE-DH-ADA---------G---
	Mycobacterium noviomagense	WP_083087097	-T---V----A-GD		-QAM-I---V---L--AH--D----------G---
	Mycobacterium palustre	WP_085080253	-------------E		V-A------V---LE-DR-AEA---------GP--
	Mycobacterium paraseoulense	WP_083169833	--------V---GA		V-A------V--ILE-DR--DA---------GQ--
	Mycobacterium parmense	WP_085270643	---Y----V---GA		V-A------V--IL--DR--DA---------GP--
	Mycobacterium persicum	WP_083153802	------------GN		--A------V--LL--D--------------GE--
	Mycobacterium pseudoshottsii J	GAQ31833	----H--------D		--A------V--LLE-D-E------------GE--
	Mycobacterium riyadhense	WP_085251032	-----V------GT		--A-----SV--ILE-DH--D----------GE--
	Mycobacterium saskatchewanense	WP_085255936	--------V---GA		V-A------V--IL--DR-DEA---------GA--
	Mycobacterium sherrisii	WP_069399852	-------------D		--A--I---V--IL--EH-EGA---------GP--
	Mycobacterium shigaense	WP_096437813	------------GL		--AT-I---V--IL--DH--DA---------GP--
	Mycobacterium shimoidei	WP_069395256	-----V------GP		T--A-----V---LE-DR-TE----------DP--
	Mycobacterium shinjukuense	WP_083048664	----T------GD		--A------V---LR-GD-------------GP--
	Mycobacterium simiae	WP_061558476	-----T-------D		--A--I--AV---L--EH-DQA---------GP--
	Mycobacterium sphagni	WP_094479222	-QA-KI------SE		-QAL-I---V--IL--E--QD----------G---
	Mycobacterium szulgai	WP_068028930	KI-Y----V---GL		--A--Y---V--ILE-DQ-D-A---------GE--
	Mycobacterium triplex	WP_036467164	-I------V----D		--A--I---M--IL--DH--ES---------GP--
	Mycobacterium ulcerans str. Harvey	EUA89410	----H--------D		--A------V--LLE-D-E------------GE--
	Mycobacterium vulneris	WP_085290279	-----------TDS		V-A------V---LE-ER--DA---------GE--
	Mycobacterium xenopi	WP_003919158	-T---V------AE		--A--L---V---LN-N--AN----------G---
	Mycolicibacillus koreensis	WP_085304107	-T---V----S-GD		VHA---------IL--ST-L-A-S-------G---
	Mycolicibacillus trivialis	WP_069391158	-T---V---VS-GD		VHA---------IL--ST-L-A-S-------G---
	Mycolicibacter algericus	WP_083036072	L---V------GP		V-AL-I---V---LE-DR--EA---------GP--
	Mycolicibacter arupensis	WP_046188704	-------V--G-		--AT-I---T---L--RL-T-A---------G---
	Mycolicibacter longobardus	WP_085266737	I---T------ES		V-A--I---T--IL--RREDQA---------GE--
	Mycolicibacter minnesotensis	WP_083023686	-----------S-		--AT-----T---L--RL-SVP---------GE--
	Mycolicibacter nonchromogenicus	WP_085137989	N---------GS		V-AT-----V---L--DR--E----------G---
	Mycolicibacter senuensis	WP_085087606	L---V------GD		V-AL-I---V---LE-DR--EA---------GP--
	Mycolicibacter sinensis	WP_064923326	L---V------GP		V-AL-I---V---LE-DR--EA---------GA--
	Mycolicibacter terrae	WP_085259892	L---V--C---GP		V-AL-I---M---L--NR--EA---------GA--
	Mycolicibacter virginiensis	WP_105295118	----V------GS		V-AT-----V---L---H--DT---------GA--
	Mycolicibacterium confluentis	WP_085149076	--------V--TAD		T-A------V---L--TG-LDA-------------

Figure S57. Partial sequence alignment of a DNA glycosylase protein showing a four amino acid insertion that is specific for members of the "Tuberculosis" clade and is absent from most other *Mycobacteriaceae*.

			78		123
"Tuberculosis" clade (11/12)	*Mycobacterium tuberculosis*	AIH36963	VRVPVLSGGHGPAERITLTVLG	LVA	PEPVPQADPGQPWPGRLPDPS
	Mycobacterium bovis	WP_069523478	----------------------	---	---------------------
	Mycobacterium bovis AF2122/97	YP_009360787	----------------------	---	---------------------
	Mycobacterium bovis BCG	ALA80051	----------------------	---	---------------------
	Mycobacterium bovis BCG str. K	AGE69441	----------------------	---	---------------------
	Mycobacterium canettii	WP_044097375	-------------------P--	---	---------------------
	Mycobacterium caprae	CEJ51783	----------------------	---	---------------------
	Mycobacterium microti OV254	PLV45227	---------R---------P--		D----H----L----Q----A
	Mycobacterium mungi	OAQ18521	----------------------	---	---------------------
	Mycobacterium orygis	WP_003417948	----------------------	---	---------------------
	Mycobacterium pinnipedii	PRH93140	----------------------	---	---------------------
	Mycobacterium africanum	CCC28476	----------------------	---	---------------------
Other *Mycobacteriaceae* (2/>100)	*Mycobacterium angelicum*	ORA01377	----------------------	---	---------------------
	Mycobacterium montefiorense	GBG36812	---------R---------P--	-ID	---G-H----L----Q-----
	Mycobacterium alsense	WP_083137352	---------R---------P--		D---------L-------E--
	Mycobacterium aquaticum	ORA22348	-Q-------R---------P--		D----R---R-----Q--E--
	Mycobacterium arosiense	WP_083065440	---------R--------IP--		D----R---DL-------E--
	Mycobacterium asiaticum	WP_065036120	-Q-------R-------F-P--		D----R---S-----Q--E--
	Mycobacterium avium	WP_062889852	-Q-------R--------IP--		D----RC--EL-------E--
	Mycobacterium bohemicum	WP_085180177	-Q-------R---------P--		D--A-H----L----Q--G--
	Mycobacterium bohemicum DSM 44	CPR13539	-Q-------R---------P--		D--A-H----L----Q--G--
	Mycobacterium branderi	WP_083130042	-Q-------R---------P--		D--------RR----Q--E--
	Mycobacterium celatum	WP_085167998	-Q-------R---------P--		D-----S--R--------E--
	Mycobacterium chimaera	WP_054585436	---------R--------IP--		D----R---DL-------E--
	Mycobacterium colombiense	WP_064883787	---------R--------IP--		D----R---DP-------E--
	Mycobacterium conceptionense	CQD05989	-Q-------R-------F-P--		D----R---R-----Q--E--
	Mycobacterium conspicuum	WP_085233601	-Q-------R---------P--		D--E-R---DR----Q--Q--
	Mycobacterium dioxanotrophicus	WP_087082773	-Q-------R---------P--		D----R---R-----Q--E--
	Mycobacterium europaeum	WP_085242228	---------R---------P--		D-VL-L----L-------E--
	Mycobacterium florentinum	WP_085219672	---------R---------P--		D----H----L----------
	Mycobacterium fragae	WP_085198459	-Q-------R---------P--		D--------NR----Q--E--
	Mycobacterium gastri	WP_036411110	-Q--A----R-------F-P--		D--I-----S-----Q-----
	Mycobacterium genavense	WP_025737451	---------R---------P--		D----H----L----Q-----
	Mycobacterium gilvum	WP_013472639	-K-------R---------PF-		D-L----N-D--------E--
	Mycobacterium gordonae	WP_065043273	-Q-------R-------M-P--		D--------A-----Q-----
	Mycobacterium grossiae	WP_083298132	---------R-------F-P--		D----R---A-----Q--E--
	Mycobacterium hassiacum	WP_051007433	-Q--T----R-------F-P--		D-Q--R---D-----Q--E--
	Mycobacterium hassiacum DSM 44	EKF24782	-Q--T----R-------F-P--		D-Q--R---D-----Q--E--
	Mycobacterium heckeshornense	WP_048893275	-Q-------R---------P--		D---AR---RR----Q--E--
	Mycobacterium heidelbergense	WP_083075126	---------R---------P--		E----R---------Q--E--
	Mycobacterium holsaticum	WP_069407817	-QM-M----R------RF-P--		D----R---R-----Q--E--
	Mycobacterium houstonense	WP_066903927	-Q-------R-------F-P--		D----R---R-----Q--E--
	Mycobacterium insubricum	WP_083031146	-QI--P---R------V--P--		D---VRD--A--------E-A
	Mycobacterium interjectum	WP_085202351	------A--R--------IP--		D----R----P----Q--Q--
	Mycobacterium intermedium	WP_069417465	-Q-------R---------P--		D--------D-----Q--Q--
	Mycobacterium intracellulare	WP_064939389	---------R--------VP--		D----R---AL-------E--
	Mycobacterium iranicum	OAN29688	-K-------R-------FIS--		D-L----N-D--------E--
	Mycobacterium kansasii	WP_063470070	-Q-------R---------P--		D--------N-----Q--E--
	Mycobacterium komaniense	WP_090279652	-Q-------R---------A--		D----R---R-----Q--E--
	Mycobacterium kyorinense	WP_045374611	-Q-------R---------P--		D--------R-----Q--E--
	Mycobacterium lacus	WP_085160800	-Q-------R---------P--		D--A-R----R----Q--G-A
	Mycobacterium lentiflavum	WP_090600608	---------R---------P--		D----D----L----------
	Mycobacterium liflandii	WP_041300072	-Q-------R--T------A--		D--------------Q--A--
	Mycobacterium llatzerense	WP_071289272	-Q-------R------M--P--		D-V--RS--TH-------E-A
	Mycobacterium mageritense	WP_036428873	-Q-------R-------F-A--		D----R---A-----Q--E--
	Mycobacterium malmesburyense	WP_090345890	-Q-------R---------P--		D----R---R-----Q--E--
	Mycobacterium malmoense	WP_065441526	---------R---------P--		D-LL-R----L-------E--
	Mycobacterium mantenii	WP_083100207	---------R---G----IP--		D----R---EL-------E--
	Mycobacterium marinum	WP_094358828	-Q-------R--T------A--		D--------------Q--A--
	Mycobacterium marseillense	WP_095577754	---------R--------IP--		D----R---AL-------E--
	Mycobacterium moriokaense	WP_083154034	-Q-------R-------F-P--		D-Q--R---S-----Q--E--
	Mycobacterium mucogenicum	WP_064980713	-Q-------R---------P--		D-V--LN--T--------E-A
	Mycobacterium nebraskense	WP_085165031	---------R---------P--		D----R----L-------E--
	Mycobacterium neoaurum	CDQ46385	---------R---------A--		D----RF--E-----Q--E--
	Mycobacterium neoaurum VKM Ac-	AHC24455	---------R---------A--		D----RF--E-----Q--E--
	Mycobacterium neumannii	WP_094294516	-Q-------R-------F-P--		D-Q--R---C-----Q--E--
	Mycobacterium neworleansense	WP_090514619	-Q-------R-------F-P--		D----R---R-----Q--E--
	Mycobacterium noviomagense	WP_083087846	-Q-------R---------P--		D----R---HR----Q--E--
	Mycobacterium novocastrense	WP_067396338	-Q-------R-------F-P--		D-Q--R---H-----Q--E--
	Mycobacterium obuense	WP_046676498	-K-------R---------P--		D-L--R---------Q--E--
	Mycobacterium palustre	WP_085079337	---------R---------P--		D----R----P----Q--E--
	Mycobacterium paraense	WP_085096309	---------R--------IP--		D----R---EP----Q--Q--
	Mycobacterium paraffinicum	WP_073880252	---------R---------P--		D----R---SL-------E--
	Mycobacterium parafortuitum	WP_083145848	-K-------R-------F-PF-		D-L----N-E--------E--
	Mycobacterium parascrofulaceum	WP_007167954	---------R---------P--		D-LL-R----L-------E--
	Mycobacterium paraseoulense	WP_083169673	---------R---------P--		D-LL-R----L-------E--
	Mycobacterium parmense	WP_085270606	---------R--------IP--		D--------DL-------G--
	Mycobacterium peregrinum	OBB24193	-Q-------R---------P--		D----R---R-----Q--E--
	Mycobacterium phlei	WP_003886509	-Q-------R---------P--		D----R---S-----Q--E--
	Mycobacterium phlei DSM 43070	KXW73927	-Q-------R---------P--		D----R---S-----Q--E--

Other *Mycobacteriaceae* (2/>100)

Mycobacterium porcinum	WP_069425023	-Q-------R-------F-Q--	D----R---R-----Q--E--
Mycobacterium rhodesiae	WP_014209007	-Q--M----R-------F-P--	D-Q--R---A-----Q--E--
Mycobacterium riyadhense	WP_085251239	-Q-------R---------P--	D--------D-----Q--N-A
Mycobacterium rufum	KGI67153	-K-------R---------P--	D----R---AA----Q--E--
Mycobacterium rutilum	WP_083410089	-Q-------R---------PF-	D----R---A-----Q--E--
Mycobacterium saskatchewanense	WP_085255624	-Q-------R---------P--	D----P---DL-------A--
Mycobacterium scrofulaceum	WP_067280621	---------R---------T--	D----R------------E--
Mycobacterium setense	WP_039318398	-Q-------R-------F-P--	D----R---R-----Q--E--
Mycobacterium sherrisii	WP_069399189	---------R---------P--	D----D----L----Q-----
Mycobacterium shigaense	WP_096437648	-Q-------R---------P--	D----H---SL-------E--
Mycobacterium shimoidei	WP_069395409	-Q-------RA-G------P--	D--------SR----Q--E--
Mycobacterium shinjukuense	WP_083045840	---------R---------P--	D--------------Q-----
Mycobacterium simiae	WP_061560071	---------R---------P--	D----H----L----Q-----
Mycobacterium smegmatis	WP_036452851	-Q-------R-------F-A--	D--------R-----Q--E--
Mycobacterium smegmatis MKD8	ELQ90798	-Q-------R-------F-A--	D--------R-----Q--E--
Mycobacterium sphagni	WP_094479075	-QL------R---------P--	D-L--R---YR----Q--E-A
Mycobacterium szulgai	WP_085672483	-Q--T----R---------P--	D--A-GP--------Q-----
Mycobacterium thermoresistibile	EHI10428	-Q-------R--V-----IP--	D----R---D--------E-A
Mycobacterium timonense	WP_083187397	-Q-------R---G----IP--	D--------DL-------E--
Mycobacterium triplex	CDO87210	---------R---------P--	D----H----L----Q-----
Mycobacterium tusciae	WP_006244220	-Q-------R-------F-A--	D--------T-----Q-SE--
Mycobacterium ulcerans	WP_071497494	-Q-------R--T------A--	D--------------Q--A--
Mycobacterium vaccae	WP_040540607	-K-------R-------F-P--	D-L---SN-D--------E--
Mycobacterium vaccae ATCC 2595	EJZ11803	-K-------R-------F-P--	D-L---SN-D--------E--
Mycobacterium vanbaalenii	WP_011778780	-K-------R-------F-P--	D-L---------------E--
Mycobacterium vulneris	WP_065458715	-Q-------R-------F-P--	D----R---R-----Q--E--
Mycobacterium wolinskyi	WP_085146730	-Q-------R-------F-P--	D----R---R-----Q--E--
Mycobacterium xenopi	WP_003919226	-Q-------R---------P--	D---AR---RR----Q--E--
Mycobacteroides abscessus subsp. abscessus	SHT49791	-Q-------R---------P--	D-V--RN--T--------E-A
Mycolicibacillus trivialis	WP_085111407	-Q-------R--G------P--	D--------R--------E--
Mycolicibacter algericus	WP_083037963	---------R---------P--	D----L---R--------E--
Mycolicibacter arupensis	WP_046189094	---------R---------P--	D----L---R--------E--
Mycolicibacter engbaekii	WP_085126546	---------R---------P--	D----L---R--------E--
Mycolicibacter heraklionensis	OBI06007	---------R---------P--	D----L---R--------E--
Mycolicibacter hiberniae	WP_085135167	---------R---------P--	D----L---R--------E--
Mycolicibacter icosiumassiliensis	WP_067970205	---------R---------P--	D----L---R--------E--
Mycolicibacter kumamotonensis	WP_065287282	---------R---------P--	D----L---R--------E--
Mycolicibacter longobardus	WP_085262988	-Q-------R---------P--	D----M---R--------E--
Mycolicibacter minnesotensis	WP_083023556	---------R---------P--	D----L---R--------E--
Mycolicibacter nonchromogenicu	ORW19648	---------R---------P--	D----L---R--------E--
Mycolicibacter senuensis	ORW68292	---------R---------P--	D----L---R--------E--
Mycolicibacter sinensis	WP_049793048	---------R---------P--	D----L---R--------E--
Mycolicibacter terrae	ORW91342	---------R---------P--	D-L--M---R--------E--
Mycolicibacter virginiensis	WP_105295436	---------R---------P--	D----L---R--------E--
Mycolicibacterium agri	WP_097939423	---------R---------P--	D--------K-----Q--E--
Mycolicibacterium aromaticivorans	WP_036340659	-QL------R---------P--	D-L--R---DR-------E-A
Mycolicibacterium aurum	WP_087031613	-Q-------R-------F-A--	D-----S--T-----Q--E--
Mycolicibacterium austroafricans	WP_036369884	-K-------R-------F-P--	D-L---------------E--
Mycolicibacterium bacteremicum	WP_083059271	-Q-------R-------F-A--	D----RF--N-----Q--A--
Mycolicibacterium boenickei	WP_097925768	-Q-------R-------F-Q--	D----R---R-----Q--E--
Mycolicibacterium brisbanense	GAS89810	-Q-------R---------P--	D----R---R-----Q--E--
Mycolicibacterium canariasense	ORV11083	-Q-------R-------F-A--	D----A---DR----Q--E-A
Mycolicibacterium celeriflavum	WP_083001117	-Q-------R---------P--	D----R---R-----Q--E--
Mycolicibacterium chlorophenolicum	WP_048471895	-K-------R---------P--	D-L--C---AA----Q--E--
Mycolicibacterium chubuense	WP_014814416	-K-------R-------F-SF-	D-A-----------AS--E--
Mycolicibacterium conceptionense	WP_064897678	-Q-------R-------F-P--	D----R---R-----Q--E--
Mycolicibacterium confluentis	WP_085149267	-Q-------R--S----F-P--	D-L--R---DR----S--E-A
Mycolicibacterium cosmeticum	CDO09919	-Q-------R-------F-A--	D----A---D-----Q--E-A
Mycolicibacterium diernhoferi	WP_073856482	-Q-------R-------F-A--	D-----S--T-----Q--E--
Mycolicibacterium doricum	WP_085192319	-Q-------R-------F-AF-	D--------H--------E--
Mycolicibacterium duvalii	WP_098004141	---------RS-----AF-A--	D-L------R-----Q--E--
Mycolicibacterium elephantis	WP_046752496	-QM------R-------F-P--	D-Q--R---R-----Q--E--
Mycolicibacterium farcinogenes	CDP84703	-Q-------R-------F-P--	D----R---R-----Q--E--
Mycolicibacterium flavescens	WP_069414586	---------R---------P--	D-L--R---A-----Q--E--
Mycolicibacterium fortuitum	OBB02027	-Q-------R---------P--	D----R---R-----Q--E--
Mycolicibacterium goodii	WP_100516578	-Q-------R-------F-A--	D--------R-----Q--E--

Figure S58. Partial sequence alignment of the hypothetical protein IQ47_16905 showing a three amino acid insertion that is specific for most members of the "Tuberculosis" clade and is absent from most other *Mycobacteriaceae*.

			141		186
"Tuberculosis" clade (9/9)	*Mycobacterium tuberculosis*	CFB06816	LSGVAAGRAGNFAVVVGINRTG	RAA	QAAQLRRHGADVVVTDLAELL
	Mycobacterium bovis	WP_050895971	----------------------	---	---------------------
	Mycobacterium canettii	WP_014001729	----------------------	---	---------------------
	Mycobacterium caprae	APU27245	----------------------	---	---------------------
	Mycobacterium microti	AMC61178	----------------------	---	---------------------
	Mycobacterium mungi	AMC61178	----------------------	---	---------------------
	Mycobacterium orygis	EMT34339	----------------------	---	---------------------
	Mycobacterium pinnipedii	PRH93134	----------------------	---	---------------------
	Mycobacterium africanum	CCC28483	----------------------	---	---------------------
Other *Mycobacteriaceae* (3/66)	*Mycobacterium haemophilum*	WP_047315611	----Q---S---GF--------	-VG	--EK---S-------------
	Mycobacterium leprae	WP_010907705	----Q--LS-H-GF--------	-VD	--EE---I--N----------
	Mycobacterium lepromatosis	WP_045842446	----Q---S---GF--------	-VG	-TEE---N-------------
	Mycobacterium alsense	OQZ91685	----Q-------GY---VD-V-		--ED---N----------D--
	Mycobacterium alsense	WP_083137355	----Q-------GY---VD-V-		--ED---N----------D--
	Mycobacterium angelicum	ORA22140	------------GF---VD-V-		--ED---N-------------
	Mycobacterium arosiense	WP_083065444	------------GF---VD-V-		--ED-----------------
	Mycobacterium avium	WP_003874438	------------GF---VD-V-		--ED---N------S------
	Mycobacterium bohemicum	WP_085180129	----Q-------GF---VD-V-		--DE--KN-------------
	Mycobacterium branderi	WP_083130045	------------GF---VD-V-		--ED---N----------D--
	Mycobacterium celatum	WP_062540230	------------GF---VD-V-		--ED---N-------------
	Mycobacterium colombiense	WP_040631117	------------GF---VD-V-		--ED---N-------------
	Mycobacterium colombiense CECT	EJO88475	------------GF---VD-V-		--ED---N-------------
	Mycobacterium fragae	WP_085198437	----E-------GF---VD-V-		--ED---N-------------
	Mycobacterium gastri	WP_036411084	----Q-------GF---VD-V-		--ED---N------N------
	Mycobacterium gordonae	KQH77897	I---Q-------GL---VD-V-		--EE--DN-------------
	Mycobacterium heckeshornense	KMV20750	------------GY----D-L-		--ED---N---I---------
	Mycobacterium interjectum	WP_066907845	----Q-------GY---VD-V-		--ED---N----------D--
	Mycobacterium intracellulare	WP_064940658	------------GF-I-VD-V-		--ED---N------S------
	Mycobacterium intracellulare s	AGP65787	------------GF-I-VD-V-		--ED---N------S------
	Mycobacterium kansasii	WP_099223361	----Q-------GF---VD-V-		--ED---N------N------
	Mycobacterium kansasii 824	EUA05477	----Q-------GF---VD-V-		--ED---N------N------
	Mycobacterium kyorinense	WP_057003436	----K-------GF---VD-V-		--ED---N----------D--
	Mycobacterium lacus	WP_085160794	------------GC---VD-V-		--ED---N-------------
	Mycobacterium malmoense	WP_083009534	----Q-------GF---VD-V-		--ED---N-------------
	Mycobacterium mantenii	WP_083093535	----E-------GF---VD-V-		--ED---N-------------
	Mycobacterium marinum	WP_020732019	I---Q-------GY---VD-V-		--QD---N-----I-------
	Mycobacterium montefiorense	GBG36821	------------GY---VD-V-		--ED--KN-------------
	Mycobacterium neworleansense	WP_090514655	-A--------K-GF---VD-V-		--E----D---I--------M
	Mycobacterium noviomagense	ORB14288	----------H-GF---VD-L-		--ED---N----------D--
	Mycobacterium palustre	WP_085079333	----Q-------GY---VD-V-		--DD---N----------D--
	Mycobacterium paraense	WP_085096490	----Q-------GY---VD-V-		--DD---N----------D--
	Mycobacterium paraintracellulare	AFC55855	------------GF-I-VD-V-		--ED---N------S------
	Mycobacterium peregrinum	WP_064878431	-A------T-K-GF---VD-V-		-------D---I--------M
	Mycobacterium porcinum	WP_083671544	-A--------K-GF---VD-V-		--E----D------------M
	Mycobacterium pseudoshottsii J	GAQ35438	I---Q-------GY---VD-V-		--ED---N-----I-------
	Mycobacterium riyadhense	ORW75664	----------S-GF---VD-V-		--EE---S---I-----G---
	Mycobacterium saskatchewanense	WP_085255595	----Q-------GF---VD-V-		--EE---D-------------
	Mycobacterium scrofulaceum	WP_067280488	I---Q-------GY---VD-V-		--DD---N-------------
	Mycobacterium septicum	WP_044516388	-A--------K-GF---VD-V-		--E----D---I--------M
	Mycobacterium sherrisii	WP_069399093	------------GF---VD-V-		--DD---N-------------
	Mycobacterium shigaense	WP_096437636	-------H----GF---VD-V-		--EE---S---I--S------
	Mycobacterium shimoidei	WP_069395353	------------GF---VD-V-		--DE--S--------------
	Mycobacterium shinjukuense	WP_083045845	----Q-AQ----GF---VD-V-		--EA---S-------------
	Mycobacterium simiae	WP_061558416	------------GY---VD-V-		--ED-------I---------
	Mycobacterium smegmatis	WP_058125639	----------K-GY---VD-V-		-GE----D----------Q--
	Mycobacterium smegmatis MKD8	ELQ90785	----------R-GF---VD-V-		--G----D----------Q--
	Mycobacterium smegmatis str. M	AFP38043	----------R-GF---VD-V-		--G----D----------Q--
	Mycobacterium szulgai	ORW92779	------------GF---VD-V-		--EE---D-----I-------
	Mycobacterium talmoniae	WP_071026537	------------GF---VD-V-		--EA-----------------
	Mycobacterium triplex	WP_036466991	------------GY---VD-V-		--ED--NN-------------
	Mycobacterium ulcerans	WP_096371604	I---Q-------GY---VD-VS		--ED---N-----I-------
	Mycobacterium vulneris	WP_065458728	-A--------K-GF---VD-V-		-------D------------M
	Mycobacterium wolinskyi	WP_085146714	------------GF---VD-V-		--Q---QE---I---------
	Mycobacterium xenopi	WP_003919229	------------GY----D-L-		H-ED---N-------------
	Mycobacterium xenopi 4042	EUA06935	------------GY----D-L-		H-ED---N-------------
	Mycolicibacterium agri	WP_097943371	-A---------------VD-L-		--DE---N---I--A------
	Mycolicibacterium aurum	WP_087019763	----------H-GY----D-V-		--E----A---T--S------
	Mycolicibacterium austroafricanum	WP_105387397	----------G-GL---VD---		-SEA---N---I-----G---
	Mycolicibacterium boenickei	WP_077743977	-A--------K-GF---VD-V-		--E----D------------M
	Mycolicibacterium conceptionen	OBB06710	-A--------K-GF---VD-V-		--G----D------------M
	Mycolicibacterium farcinogenes	CDP84801	-A--------K-GF---VD-V-		--G----D------------M
	Mycolicibacterium goodii	WP_049748048	----------K-GF---VD-V-		--E----D----------Q--

Figure S59. Partial sequence alignment of a hydrolase protein showing a three amino acid insertion that is specific for members of the "Tuberculosis" clade and is absent from most other *Mycobacteriaceae*.

			19		68
“Tuberculosis” clade (20/22)	*Mycobacterium tuberculosis*	AMC72874	GGGTLLPMADVIRMTSHAHHY	SPASGRYP	QAIFDHGTPLALYHTKRLASP
	Mycobacterium africanum GM0411	CCC25169	---------------------	--------	---------------------
	Mycobacterium africanum K85	KBF47630	---------------------	--------	---------------------
	Mycobacterium africanum MAL010	KBG08006	---------------------	--------	---------------------
	Mycobacterium bovis	WP_080663112	---------------------	--------	---------------------
	Mycobacterium bovis AF2122/97	YP_009360856	---------------------	--------	---------------------
	Mycobacterium bovis AN5	ESK75391	---------------------	--------	---------------------
	Mycobacterium bovis B2 7505	KAN87758	---------------------	--------	-------K-------------
	Mycobacterium bovis BCG	AAB96960	---------------------	--------	---------------------
	Mycobacterium bovis BCG str. A	AHM09251	---------------------	--------	---------------NG----
	Mycobacterium bovis BCG str. P	CAL71612	---------------------	--------	---------------------
	Mycobacterium bovis Bz 31150	KAN92248	---------------------	--------	---------------------
	Mycobacterium bovis MAL010093	KBG51344	---------------------	--------	---------------------
	Mycobacterium canettii	WP_080602950	---S----------A------		L--------------------
	Mycobacterium canettii CIPT 14	CCK65684	---S----------A------		L--------------------
	Mycobacterium caprae	WP_083647839	---------------------	--------	---------------------
	Mycobacterium microti	AMC57538	---------------------	--------	---------------------
	Mycobacterium mungi	WP_082917960	---------------------	--------	---------------------
	Mycobacterium orygis	WP_081608392	---------------------	--------	---------------------
	Mycobacterium orygis 112400015	EMT33889	---------------------	--------	---------------------
	Mycobacterium pinnipedii	PRH91488	---------------------	--------	---------------------
	Mycobacterium africanum	WP_080699388	---------------------	--------	---------------------
Other *Mycobacteriaceae* (1/52)	*Mycobacterium gordonae*	WP_065044389	A-------S--L-LATS----	A-------	-T---QDKT-----G----T-
	Mycobacterium alsense	WP_083141168	A--S----T-----AG-----		L-----AK------------R
	Mycobacterium angelicum	ORA12304	----I---P----LGRQ-N--		L----Q-K-------------
	Mycobacterium arosiense	WP_083067482	A-------S----LAR--R--		L------KA-----AR-----
	Mycobacterium arosiense ATCC B	ORA09870	-A--I---S----LAR-----		L------K-------------
	Mycobacterium asiaticum	WP_065123542	S--S------L---AAQ----		L-V--DAK------A------
	Mycobacterium avium	WP_062895105	A-------S----LAR-----		L-V--N-K-------------
	Mycobacterium bohemicum	WP_085181641	A----V--S----WAG-----		L------K-------------
	Mycobacterium chimaera	ASL09894	----IV--S----LGR-----		L----E-KA------------
	Mycobacterium colombiense	OBJ73102	----I---S----LAR-----		L----K-KA------------
	Mycobacterium conspicuum	WP_085233296	----I---S----LAR-----		L----K-KA------------
	Mycobacterium europaeum	WP_085240419	----I---S----LAR-----		L-V--K-K-V----G------
	Mycobacterium fragae	WP_085194447	--------S----LA------		L----K-KA-G---S------
	Mycobacterium gastri	WP_036415427	---SR---S-L---A------		L-L--R-KA------------
	Mycobacterium heidelbergense	ORA65721	---S---LS-----A------		L-L--NAK---------F---
	Mycobacterium interjectum	WP_085202023	A----V--S----WAG-----		L----K-KA---H--------
	Mycobacterium intracellulare	WP_064893654	---S-V--S-L---A------		L----R-RA-S-H--------
	Mycobacterium intracellulare 1	EUA59687	---S-V--S-L---A------		L----R-RA-S-H--------
	Mycobacterium intracellulare A	AFC44015	---S-V--S-L---A------		L----R-RA-S-H--------
	Mycobacterium intracellulare M	AFC49172	---S-V--S-L---A------		L----R-RA-S-H--------
	Mycobacterium intracellulare s	ARR85558	---S-V--S-L---A------		L----R-RA---H--------
	Mycobacterium kansasii	KZS64453	---SQ---S-L--LA------		L-L--D-KA-----S------
	Mycobacterium kansasii 732	EUA06392	---S----S----LAR--N--		L----D-KA-----S------
	Mycobacterium kansasii 824	EUA01286	--------S----LAR-----		L-L--N-K-------------
	Mycobacterium kubicae	WP_085072951	--------S----LSR-----		L----R-NA------------
	Mycobacterium lacus	WP_085155832	---SRV--S-L---A-S----		L-L--G-K------A------
	Mycobacterium malmoense	WP_065442061	A----V--S----WAG-----		L----RAR-------------
	Mycobacterium mantenii	WP_083099146	----VV--S----WA------		L------KA---H--------
	Mycobacterium marseillense	ORA94115	----I---S----LDR--N--		L----K-NA------------
	Mycobacterium microti	AMC59312	--------S----LA---N--		LR-----RE------------
	Mycobacterium nebraskense	WP_046186637	A----V--S----WAG-----		L----R-RS------------
	Mycobacterium orygis	WP_003405993	---SRV--S-L---A------		L-L--GAK-------------
	Mycobacterium orygis 112400015	EMT35696	---SRV--S-L---A------		L-L--GAK-------------
	Mycobacterium palustre	WP_085079703	-----V--S----WA---Y--		L----R-KA---H--------
	Mycobacterium paraense	WP_085094522	----I---S----QAR---Q-		L----K-QAI-----------
	Mycobacterium paraintracellulare	AFC54248	----I---SE---LAR-----		L----K-KA------------
	Mycobacterium parascrofulaceum	WP_007171972	A----V--S----WAG-----		L----QAR-------------
	Mycobacterium paraseoulense	WP_083172732	----I---S----LAR-----		L-V--R-E------G------
	Mycobacterium parmense	WP_085272093	---SW---S----LAR-----		L----R-KA----------T-
	Mycobacterium pinnipedii	PRH93658	---SRV--S-L---A-N----		L-L--GAK-------------
	Mycobacterium scrofulaceum	WP_083180051	A----V--S----WAG-----		L-----AR-------------
	Mycobacterium shimoidei	WP_069398270	--------SE---LAA-----		LV--E--KAIG-F--------
	Mycobacterium shinjukuense	WP_083052532	---S----SE---LAA-----		LR-----RE------------
	Mycobacterium szulgai	WP_085671879	----I---S----LGRQ-N--		L----E-K-------------
	Mycobacterium xenopi	WP_085194972	---S----SE---LAQ-----		L----K-RA--------I---
	Mycolicibacillus trivialis	ODR01013	--------S----LAR-----		L----R-KTIG-F--------

Figure S60. Partial sequence alignment of the hypothetical protein RN11_1864 showing an eight amino acid insertion that is specific for most members of the “Tuberculosis” clade and is absent from most other *Mycobacteriaceae*.

Group	Species	Accession	162		211
"Tuberculosis" clade (8/8)	*Mycobacterium tuberculosis*	AMC79382	IDVKGLRLTLTQAAWRVAEDS	LASRECPQ	PADIDVATAGFWAAEAGHRVA
	Mycobacterium bovis BCG	AMC52727	---------------------	--------	---------------------
	Mycobacterium canettii	WP_014001776	---------------------	--------	---------------------
	Mycobacterium microti	AMC61290	---------------------	--------	---------------------
	Mycobacterium mungi	OAQ18758	---------------------	--------	---------------------
	Mycobacterium orygis	EMT34247	---------------------	--------	---------------------
	Mycobacterium pinnipedii	PRH93603	---------------------	--------	---------------------
	Mycobacterium africanum	AMC66029	---------------------	--------	---------------------
Other *Mycobacteriaceae* (0/52)	*Mycobacterium alsense*	WP_083137826	-----------------S-EI		--E----S------D------
	Mycobacterium angelicum	WP_083116368	---------------K---EV		---------A-----------
	Mycobacterium avium subsp. paratuberculosis	ETB36650	-----------------S--L		--EV-----A----D------
	Mycobacterium branderi	WP_083131042	----------------LS--L		---TE---------D------
	Mycobacterium gordonae	WP_065132982	---------------K----I		--E----S-A----D------
	Mycobacterium grossiae	WP_070354218	--L------V-------S-GL		--QLE-DS-A-----------
	Mycobacterium hassiacum	WP_005630437	----A----V------IS-NL		--ETE-N--A-----------
	Mycobacterium holsaticum	WP_069404423	----A-----------L--NI		--EVE-N--A-----------
	Mycobacterium kansasii	WP_103846035	---------------K----L		--EL---S-A----D------
	Mycobacterium kansasii 662	EUA10305	---------------K----L		--EL---S-A----D------
	Mycobacterium kansasii 732	EUA00967	---------------K----L		--EL---S-A----D------
	Mycobacterium komaniense	WP_090279879	----A-----------LS--L		---V--N--A-----------
	Mycobacterium kubicae	WP_085072611	-----------------S--V		-------S-A----D------
	Mycobacterium lacus	WP_085162160	-----------------S--V		-------S-A----D------
	Mycobacterium liflandii	WP_015357359	-----------------S--L		--E----S-------------
	Mycobacterium llatzerense	WP_043984605	----A--------S-----GL		--E---N--A-----------
	Mycobacterium mageritense	WP_036430025	----------------LS--L		------SS-A-----------
	Mycobacterium malmesburyense	WP_090342602	----A-----------LS-GL		---V--N--A----D------
	Mycobacterium marinum	WP_094360547	-----------------S--L		--E----S-------------
	Mycobacterium moriokaense	WP_083156648	----A----V------LS--L		------N--A-----------
	Mycobacterium mucogenicum	WP_053855393	----A--------S-----GL		--E---N--A-----------
	Mycobacterium nebraskense	WP_046184748	-----------------S--L		-------S-A----D------
	Mycobacterium nebraskense	WP_047321910	-----------------S--L		-------S-A----D------
	Mycobacterium neumannii	WP_094294060	----A-----------LS--L		---V--S--A----D------
	Mycobacterium noviomagense	WP_083085418	----------------LS--L		--GTE---SA----D------
	Mycobacterium novocastrense	WP_067393849	----A-----------LS--L		---L--G--A----D------
	Mycobacterium parmense	WP_085268281	-----------------S--L		-------S-A----D------
	Mycobacterium phlei	WP_003890630	----A-----------LS-GL		--EV--N--A-----------
	Mycobacterium pseudoshottsii	WP_086085382	-----------------S--L		--E----S-------------
	Mycobacterium rhodesiae	WP_014210733	----A----V------LS--L		---VE-N--A-----------
	Mycobacterium riyadhense	WP_085252608	-----------------S--V		---------A-----------
	Mycobacterium rutilum	WP_083407103	----A-----------LS-GL		---V--N--A----D------
	Mycobacterium septicum	WP_044520595	----------------LS--L		-------S-S-----------
	Mycobacterium shinjukuense	WP_083048583	---------------K-SQ-I		---------A----D------
	Mycobacterium szulgai	WP_068025895	-----------------S--V		-------S-A----D------
	Mycobacterium thermoresistibile	WP_003926320	--I--------------S-QM		---V--H--A-----------
	Mycobacterium tusciae	WP_006246477	----A----V------LS--L		----E-N--A-----------
	Mycobacterium ulcerans	WP_096369650	-----------------S--L		--E----S-------------
	Mycobacteroides abscessus	WP_100523804	----------W------S--L		-S-V-----K----D------
	Mycobacteroides abscessus subsp. abscessus	SHV81298	----A--------S-----GL		--E---N--A-----------
	Mycobacteroides chelonae	WP_070932666	----------W------S--L		---V-----K---SD------
	Mycobacteroides franklinii	WP_070938247	----------W------S--L		---V-----K---SD------
	Mycobacteroides immunogenum	WP_043076722	----------W------S--L		---V-----K----D------
	Mycobacteroides salmoniphilum	WP_078328081	----------W------S--L		---V-----K---SD------
	Mycobacteroides saopaulense	WP_070909104	----------W------S-EL		---V-----K----D------
	Mycolicibacterium agri	WP_097944241	----A----V------LS--L		--EV--N--A-----------
	Mycolicibacterium celeriflavum	WP_083007098	----A-----------LS-NL		---V--S--A----D------
	Mycolicibacterium chubuense	WP_014817736	--I-A-----------LS-GL		--EV--N--A-----------
	Mycolicibacterium confluentis	WP_085153576	--I-------------LS--L		---V--R--A-----------
	Mycolicibacterium flavescens	WP_069413815	----A-----------LS-GL		---V--N--A----D------
	Mycolicibacterium fortuitum	WP_064867998	----------------LS--L		-------STA-----------

Figure S61. Partial sequence alignment of the acyl-CoA dehydrogenase FadE27 protein showing an eight amino acid insertion that is specific for members of the "Tuberculosis" clade.

Group	Species	Accession	25		65
“Tuberculosis” clade (9/10)	*Mycobacterium tuberculosis*	AIH83748	GAAYYLKTMQPAKTFAIVEAR		YPAIRSDSDLHMFSYEFKPW
	Mycobacterium bovis	WP_107193441	---------------------		-----------TS-------
	Mycobacterium bovis AN5	ESK70297	---------------------		-----------T--------
	Mycobacterium canettii CIPT 14	CCC46093	---------------------		-----------T--------
	Mycobacterium caprae	APU28114	---------------------		-----------T--------
	Mycobacterium microti	AMC61553	---------------------		-----------T--------
	Mycobacterium mungi	OAQ19131	---------------------		-----------T--------
	Mycobacterium orygis	WP_003399792	S--WH-QDRC-T-SY--L-K-	ESMGGTWDLFR	--G------MYTLGFR-R--
	Mycobacterium pinnipedii	PRH91703	---------------------		-----------T--------
	Mycobacterium africanum	CCC28820	---------------------		-----------T--------
Other *Mycobacteriaceae* (0/40)	*Mycobacterium angelicum*	WP_083114354	---H--Q------S-------	DDIGGTWDLFR	--G--------T------A-
	Mycobacterium aquaticum	WP_083163338	------QQEH-GRN---L---	GATGGTWDLFR	--G--------T-G------
	Mycobacterium asiaticum	WP_065144905	------QREH-GRSY--L---	GATGGTWDLFR	--G--------T-G------
	Mycobacterium bohemicum	WP_085181533	------Q----NR--------	GEIGGTWDLFR	--G--------T--------
	Mycobacterium conceptionense	CQD19822	---H--QREH-GRRYM-L---	SAIGGTWDLFR	--G--------TYG------
	Mycobacterium dioxanotrophicus	WP_087075042	------QQEH-GRN---L---	GATGGTWDLFR	--G--------T-G------
	Mycobacterium gastri	WP_036410729	----H---YR-GT----L-G-	ESIGGTWSLFQ	--G------MPS-G-G----
	Mycobacterium gastri 'Wayne'	ETW25669	----H---YR-GT----L-G-	ESIGGTWSLFQ	--G------MPS-G-G----
	Mycobacterium gordonae	OBS03650	------Q----G-S---L---	ADIGGTWDLFR	--G--------T------A-
	Mycobacterium houstonense	WP_066897409	------QREH--RSY------	GATGGTWDLFR	--G--------T-G-Q----
	Mycobacterium intermedium	WP_069418835	------QKDH-GR-Y--L---	GATGGTWDLFR	--G--------T-G------
	Mycobacterium kansasii	ORB84657	------QA---H---------	DDIGGTWDLFR	--G--------T------A-
	Mycobacterium kansasii ATCC 12	AGZ51213	------R--------------	GDIGGTWDLFR	--G--------T------A-
	Mycobacterium lacus	WP_085161105	---------------------	ADIGGTWDLFR	--G--------T------A-
	Mycobacterium liflandii	WP_015357597	------QEH------------	NDIGGTWDLFR	--G---E---NT------A-
	Mycobacterium litorale	WP_078020230	-LGH--A-TL-G----L-DS-	DAIGGTWDLFR	--G--------T-G------
	Mycobacterium llatzerense	WP_043984748	------QQEH-GRSY--L-S-	GATGGTWDLFR	--G--------T-G------
	Mycobacterium mageritense	WP_036432246	----H---FR--T---VL-G-	DSIGGTWNLHQ	--G------MPT-G-G----
	Mycobacterium marinum	WP_012396787	------QEH------------	NDIGGTWDLFR	--G--------T------A-
	Mycobacterium mucogenicum	WP_053854296	------QQEH-GRSYT-L-S-	GATGGTWDLFR	--G--------T-G------
	Mycobacterium neoaurum	WP_030136205	------QREH-GRDYT-L---	QSTGGTWDLFR	--G--------T-G------
	Mycobacterium rhodesiae	WP_005141504	-MGH--V-G--G------DS-	EAIGGTWDLFR	--G--------T-G------
	Mycobacterium riyadhense	WP_085252723	------QKHH-GR-Y--L---	GATGGTWDLFR	--G--------T-G------
	Mycobacterium setense	WP_064874682	----H---YR-GT---VL-G-	DSIGGTWSLHQ	--G------MPT-G-G----
	Mycobacterium shinjukuense	WP_083046741	---H--QK--A----------	ADIGGTWDLFR	--G--------T------A-
	Mycobacterium sphagni	WP_094476643	-LGH--V-Q--G-----ADS-	DAIGGTWDLFR	--G--------T-G------
	Mycobacterium szulgai	WP_085673537	---H--Q--------------	DDIGGTWDLFR	--G--------T------A-
	Mycobacterium tusciae	WP_083127306	------QKDH-DRSY--L---	GASGGTWDLFR	--G--------T-G------
	Mycobacterium ulcerans	WP_011741956	------QEH------------	NDIGGTWDLFR	--G--------T------A-
	Mycobacterium ulcerans str. Harvey	EUA86658	------QEH------------	NDIGGTWDLFR	--G--------T------A-
	Mycobacteroides abscessus subsp. abscessus	SHW22920	------QQEH-GRSY--L-S-	GATGGTWDLFR	--G--------T-G------
	Mycolicibacterium aromaticivorans	WP_051659881	-MGH--A----G-S----DS-	DAIGGTWDLFR	--G--------T-G------
	Mycolicibacterium aurum	WP_087026162	------QRDH-GRSYT-L-S-	GATGGTWDLFR	--G--------T-G------
	Mycolicibacterium brisbanense	WP_062830665	------QQEH-GRN---L---	GATGGTWDLFR	--G--------T-G------
	Mycolicibacterium canariasense	GAS98011	------QREH-GRSY--I---	GATGGTWDLFR	--G--------T-G------
	Mycolicibacterium conceptionense	WP_064894335	------QREH-GRRYM-L---	SAIGGTWDLFR	--G--------TYG------
	Mycolicibacterium cosmeticum	WP_036396920	------QREH-GRSY--I---	GATGGTWDLFR	--G--------T-G------
	Mycolicibacterium diernhoferi	WP_073858792	------QRDH-GRSYT-L-S-	GATGGTWDLFR	--G--------T-G------
	Mycolicibacterium farcinogenes	WP_036387415	------QREH-GRRYM-L---	NAIGGTWDLFR	--G--------TYG------
	Mycolicibacterium fortuitum	ALI28406	------QREH-QRSY--L---	GVTGGTWDLFR	--G--------T-G------

Figure S62. Partial sequence alignment of an oxidoreductase protein showing an eleven amino acid deletion that is specific for most members of the “Tuberculosis” clade.

Group	Species	Accession	113		159
"Tuberculosis" clade (9/10)	*Mycobacterium tuberculosis*	AIH40693	MIAGAAGLCAVATAIGVGA		VVDAPPPAPSAPTTAQHITVSKPAPVIP
	Mycobacterium bovis	WP_044797969	-------------------		----------------------------
	Mycobacterium bovis BCG	AMC53222	-------------------		----------------------------
	Mycobacterium canettii	WP_014002029	-------------------	VA	LI--------------------------
	Mycobacterium caprae	APU24247	-------------------		----------------------------
	Mycobacterium microti	WP_105799879	-------------------		----------------------------
	Mycobacterium mungi	OAQ18867	-------------------		----------------------------
	Mycobacterium orygis	EMT37754	-------------------		----------------------------
	Mycobacterium pinnipedii	PRH91305	-------------------		----------------------------
	Mycobacterium africanum	AMC66536	-------------------		----------------------------
Other *Mycobacteriaceae* (0/39)	*Mycobacterium alsense*	WP_083139489	AV--V--VG--LA-----T	AA	LLN--E-T----V--E-----T-PMT--
	Mycobacterium arosiense	WP_083064680	T--A-------LA---L-T	SA	LLH--E---E--GDV----L-T-PME--
	Mycobacterium asiaticum	WP_065034065	L---I------VA-VA--T	TA	LLHE--STA-T-A--D-----T-V-L--
	Mycobacterium avium	WP_033730589	A--AV---G-ALA-F---T	VA	LLRT-A----T--DIE-----T-PMQ--
	Mycobacterium avium	WP_038430586	A--AV--SG-ALA-----T	VA	LLRT-A----T--DIE-----T-PMQ--
	Mycobacterium avium 09-5983	ETB18351	--G-ALA-----T	VA	LLRT-A----T--DIE-----T-PMQ--
	Mycobacterium bohemicum	WP_085179808	VV--L--V---LA-V---T	LA	LLRS-A---------E-----A-PM--S
	Mycobacterium celatum	WP_085167563	VV-A---VA--GV-VAL-T	AA	L-SE-A-T--SQI--------R-PAA--
	Mycobacterium chimaera	WP_072501544	L--AV-----PLA--RLRP	AR	LIA-VAG---T-GDV------T-PMEV-
	Mycobacterium colombiense	OBJ58540	T--A---V---LA---F-T	IA	LLH--E---DT-GDV------T-PME--
	Mycobacterium europaeum	WP_085239316	-V-AV--VA-ILA-V---T	AA	LIT--E----TQV--E-----T-PM---
	Mycobacterium florentinum	WP_085219527	VA--V--VG--LA---F-T	AA	LLNE-E----T----M-----T-PMA--
	Mycobacterium gastri	WP_036418423	IL--I-----AVA-----T	AA	L-T--A----T----M-----T-P----
	Mycobacterium genavense	WP_025737867	--LA---F-T	AA	LL-E-E---------L-----A-PMA--
	Mycobacterium gordonae	WP_055579943	VV--I--V---LA-V-I-T	MS	LLNE-DSV-G--V--D-----T-V----
	Mycobacterium haemophilum	WP_047315650	V--AVT--G--VA-----T	AA	LI---AST-AE-A--------T-P----
	Mycobacterium heidelbergense	WP_083075756	LAV-V--VG-ALA-----T	AA	L-H--G-T----V--E-----T-P-A--
	Mycobacterium interjectum	WP_066917037	LA--V--VG--LA-----T	AA	LLN--Q----T-A--E-----T-PM---
	Mycobacterium intermedium	WP_069421178	V---LI--A--LA-V-L-T	AA	LLN----E--T-RS--L----TTP-T--
	Mycobacterium intracellulare	WP_014383556	L--AV-----ALA---F-T	VA	LLH--E----T-GDV------T-PMEV-
	Mycobacterium kansasii	OOK75776	IL--I--F--AVA-----T	AA	L-T--S---------M-----T-P----
	Mycobacterium lacus	WP_085163139	VL--I-----AVA---L--	AA	L--G-A-T--G----E-----T-P----
	Mycobacterium liflandii 128FXT	AGC65128	IL--I---I-LIA---L-T	AA	LIT--G-T--G-P--M-----T-P----
	Mycobacterium malmoense	WP_065441537	VL-TVI-VG--LA-----T	AA	L-T--E----TQV--E-----T-PM---
	Mycobacterium mantenii	WP_083099529	T--AV------LA---F-T	VA	LLH--E---DT-GDV------T-PME--
	Mycobacterium marinum	WP_012396974	IL--I---I-LIA---L-T	AA	LIT--G-T--G-P--M-----T-P----
	Mycobacterium microti OV254	PLV47966	IL-----IG--LA-A-F-T	AA	LLHE--S---T---IE-----T-PMA--
	Mycobacterium nebraskense	WP_047322614	VV--V--V---LA-V---T	AA	LIT--G----T-V--E-----T-PM---
	Mycobacterium paraense	WP_085104579	LA--V--VA--LA-----T	AA	LL---E-T--T-A--E-----T-PM---
	Mycobacterium paraffinicum	WP_073880719	V---V--VG-LLA-VD--T	AA	LIN--E----TGV--E-----T-PMA--
	Mycobacterium paraintracellulare	WP_014386141	L--TV-----ALA---F-T	VA	LLH--E----T-GDV------T-PMEV-
	Mycobacterium paraseoulense	WP_083171234	VV-TV--VG--LA-V---T	AA	LIT--E----T-V--E-----T-PMA--
	Mycobacterium parmense	WP_085268192	LV-AVT-S--LLA---F-T	AA	LLR--Q------V--E-----T-PMAV-
	Mycobacterium persicum	WP_083154545	IL--I--F--AVA-----T	AA	L-T--S---TT-A--M-----T-P----
	Mycobacterium riyadhense	WP_085252970	V---V------IA---L-T	RA	LL------------------T---A--
	Mycobacterium scrofulaceum	WP_067279825	VV--V--VG-LLA-V---T	AA	LIN--E----TGV--E-----T-PMA--
	Mycobacterium shinjukuense	WP_085163139	VL--I-----AVA---L--	AA	L--G-A-T--G----E-----T-P----
	Mycobacterium ulcerans	WP_011742518	IL--I---I-LIA---L-T	AA	LIT--G-T-NG-P--M-----T-P----
	Mycobacterium vulneris	WP_085290981	T--A---V---LA---F-T	VA	LLH--E---DT-GDVE-----T-PME--

Figure S63. Partial sequence alignment of the hypothetical protein IQ42_20035 showing a two amino acid deletion that is specific for most members of the "Tuberculosis" clade.
